# Direct histone proteoform profiling of the unannotated, endangered coral *Acropora cervicornis*

**DOI:** 10.1093/nar/gkaf740

**Published:** 2025-07-31

**Authors:** Cassandra N Fuller, Sabrina Mansoor, Kevin Jeanne Dit Fouque, Lilian Valadares Tose, Javier Rodriguez-Casariego, Mariangela Kosmopoulou, Detlev Suckau, Francisca N de Luna Vitorino, Benjamin A Garcia, Jose M Eirin-Lopez, Francisco Fernandez-Lima

**Affiliations:** Department of Chemistry and Biochemistry, Florida International University, Miami, FL 33199, United States; Environmental Epigenetics Laboratory, Institute of Environment, Florida International University, Miami, FL 33199, United States; Department of Chemistry and Biochemistry, Florida International University, Miami, FL 33199, United States; Department of Chemistry and Biochemistry, Florida International University, Miami, FL 33199, United States; Environmental Epigenetics Laboratory, Institute of Environment, Florida International University, Miami, FL 33199, United States; Department of Marine Biology and Ecology, Rosenstiel School, University of Miami, Miami, FL 33124, United States; Fasmatech Science & Technology, TESPA Lefkippos, NCSR Demokritos, Agia Paraskevi, Athens 15341, Greece; Bruker Daltonics GmbH & Co. KG, Bremen 28359, Germany; Department of Biochemistry and Molecular Biophysics, Washington University School of Medicine, St. Louis, MO 63110, United States; Department of Biochemistry and Molecular Biophysics, Washington University School of Medicine, St. Louis, MO 63110, United States; Environmental Epigenetics Laboratory, Institute of Environment, Florida International University, Miami, FL 33199, United States; Department of Chemistry and Biochemistry, Florida International University, Miami, FL 33199, United States; Biomolecular Sciences Institute, Florida International University, Miami, FL 33199, United States

## Abstract

Epigenetic modifications directly regulate the patterns of gene expression by altering DNA accessibility and chromatin structure. A knowledge gap is presented by the need to directly measure these modifications, especially for unannotated organisms with unknown primary histone sequences. In the present work, we developed and applied a novel workflow for identifying and annotating histone proteoforms directly from mass spectrometry-based measurements for the endangered Caribbean coral *Acropora cervicornis*. Combining high-accuracy *de novo* top-down and bottom-up analysis based on tandem liquid chromatography, trapped ion mobility spectrometry, non-ergodic electron-based fragmentation, and high-resolution mass spectrometry, near complete primary sequence (up to 99%) and over 86 post-translational modification annotations were obtained from pull-down histone fractions. In the absence of reliable genome annotations, H2A, H2B, and H4 histone sequences and the annotation of the post-translational modifications of the stressed *A. cervicornis* coral allow for a better understanding of chromatin remodeling and new strategies for targeting intervention and restoration of endangered reef corals.

## Graphical Abstract

**Graphical Abstract:**
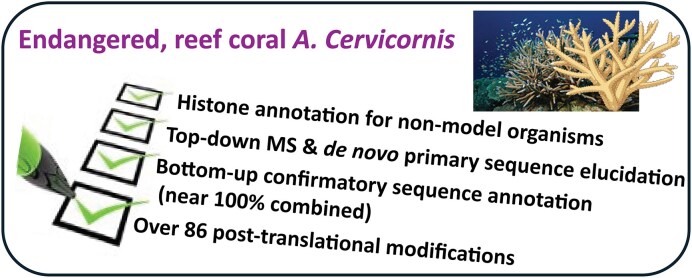

## Introduction

Epigenetic modifications stand out due to their responsiveness to environmental signals and reversibility as potential tools for phenotype manipulation and enhancement in endangered species threatened by climate change. Corals have evolved remarkable phenotypic plasticity [1], involving genetic and non-genetic mechanisms that allow these organisms to acclimate and maximize their survival in rapidly changing environments [2–4]. The endangered staghorn coral (*Acropora cervicornis*) is one of the main builders of the structural base of Caribbean coral reefs, providing extensive habitats for marine organisms and contributing to coastal protection [5]. Coral reefs in general, and *A. cervicornis* in particular, are uniquely susceptible to rapid variations in environmental conditions resulting in a dramatic decline of their populations under current global climate change conditions [6, 7], despite extensive restoration efforts.

Epigenetic reprogramming via DNA methylation has been the main focus of attention as a target for intervention [8], leaving other critical mechanisms (notably those intimately involved in chromatin remodeling) and their interactions understudied [9]. Histone protein variations and their post-translational modifications (PTMs) represent a critical epigenetic mechanism modulating gene function and DNA metabolism through structural changes at the chromatin level [10, 11]. Histones are a diverse family of basic proteins that bind to the DNA, forming the nucleosome, a highly dynamic chromatin fundamental subunit [12]. The affinity of histones for DNA is critically influenced by chemical PTMs in the amino-terminal tails (e.g. acetylation [ac], ubiquitination [ub], phosphorylation [ph], and methylation [me_1–3_]), facilitating the activation/repression of different genome regions in response to environmental signals [10, 13]. Despite its fundamental role in epigenetic regulation, studies addressing the epigenetics of chromatin structural components (e.g. histone variants and PTMs) in corals are scarce [9, 14]. The purification of high-quality histones and the lack of specific antibodies remain the main challenges hampering coral epigenetic studies. The lack of appropriate reference information on histone variant sequences and occurring chemical modifications in proteins constitutes a strong limiting factor. Consequently, a major knowledge gap related to coral’s chromatin composition and dynamics remains to be addressed.

Mass spectrometry (MS) methods have shown potential for the analysis and identification of histones, especially due to continuous improvements in the mass resolving power and mass accuracy over the last decades [15]. Traditional bottom-up MS proteomics is the most widely used method for histone PTM analysis [16–18], which has benefitted largely from the commercial integration of non-ergodic electron-based dissociation methods (e.g. electron transfer dissociation, ETD; and electron capture dissociation, ECD) [19], and more recently, from the mobility separation of positional isomers before tandem MS/MS [20–22]. A major trade-off from histone bottom-up analysis is the need for derivatization methods to produce longer and more informative proteolytic peptides [23–26], in addition to the possibility of losing information about the protein during digestion. Middle-down MS proteomics approaches using enzymes that only partially digest the histones have shown promise in the characterization of histone tails with varying PTMs [27]; nevertheless, the high isomeric content requires the use of complementary pre-separation and electron/UV-based fragmentation for more informative sequencing and PTM localization [28–32]. Top-down MS proteomics has been effective for the annotation of histone proteoforms [33–44]; however, it requires pre-fractionation steps and ultra-high resolution to account for histone chemical diversity and complexity [45–47]. Regioisomers ubiquitously co-exist in cells and will be ideally resolved before the MS step due to challenges associated with disentanglement of tandem MS/MS from more than two concurrent variants [48], despite recent efforts using fragment correlation MS [49]. Top “double down” MS includes mobility separation steps to achieve isomeric separation and reduce the spectral complexity leading to better sequence coverage and characterization of histone proteoforms [36]. While sub-stoichiometric histone modifications render epigenetic studies more difficult than general proteome analyses from the sensitivity perspective, an added challenge arises from the characterization of non-annotated species.

In this work, a new workflow capable of direct protein sequence generation and proteoform PTM annotation based on liquid chromatography (online and offline), trapped ion mobility spectrometry (TIMS), ECD fragmentation, and high-resolution MS is described and applied to the unannotated, endangered Caribbean coral *Acropora cervicornis*. The method combines histone pull-down extraction followed by liquid chromatography (online and offline), top-down mobility- and mass-precursor ion isolation before non-ergodic electron-based fragmentation and high-resolution molecular fragment detection. The *de novo* processing of the top-down data, combined with parallel bottom-up sequence confirmation, allows for direct full primary sequence determination and PTM annotation of histone proteoforms. When applied to the endangered staghorn coral *Acropora cervicornis*, a high diversity of PTMs based on mono-, di-, and tri-methylation, oxidation, and acetylation was detected. The *de novo* top-down analysis provided high sequence coverage (∼90%) for all the proteoforms, and when combined with bottom-up confirmation, full sequence coverages were obtained. Data showed that the high diversity of proteoforms (e.g. isomeric species) and depth of the analysis requires the use of complementary separations, not accessible using traditional proteomic analysis.

## Materials and methods

### Endangered coral histone extraction

Three coral colonies (*Acropora cervicornis* genets) were obtained from the Coral Reef Foundation nursery located at Tavernier, Florida Keys (N 24.982715°, W -80.436286°) and were propagated under CRF’s permit #FKNMS-2019-012-A2. Histones were isolated from 12 g of powdered coral fragments, pooled from propagated colonies that were exposed to nutrient stress. Briefly, flash-frozen coral fragments were manually powdered in liquid nitrogen using a mortar and pestle. Coral tissue was separated from the calcium carbonate skeleton by suspending powdered coral (∼250 mg/mL) in pre-chilled 1× Pre-lysis buffer (EpiQuik™ Total Histone Extraction Kit, EpigenTek, Farmingdale, NY) on ice for 10 min [50]. The tissue slurry was transferred to a 2 mL Dounce homogenizer and the EpiQuik™ Total Histone Extraction Kit was used (see details in Supplementary Fig. S1). The supernatant fraction containing the acid-soluble proteins was subjected to acetone precipitation overnight [51]. The coral histone protein concentration and quality were assessed using the Qubit Protein Assay Kit (Invitrogen: Carlsbad, CA) and sodium dodecyl sulfate–polyacrylamide gel electrophoresis (SDS–PAGE), respectively. Samples were resuspended in molecular-grade, sterile water and stored at −80°C.

### Top-down MS coral proteoform analysis

The analysis consisted of liquid chromatography separation (online and offline) coupled with TIMS and tandem ECD-MS/MS. Details on the experimental LC-TIMS-q-ECD-TOF MS/MS platform based on a Bruker Maxis Impact II ToF MS (Bruker Daltonics Inc., Billerica, MA) can be found elsewhere (Supplementary Fig. S2) [22]. Liquid chromatography was carried out on a Dionex Ultimate 3000 LC system equipped with an XBridge peptide BEH C_18_ column (4.6 × 250 mm × 5 μm) maintained at a temperature of 50°C. The mobile phase consisted of (A) 5% acetonitrile (ACN) with 0.2% trifluoroacetic acid (TFA) and (B) 95% ACN with 0.2% TFA with the following gradient: (i) 0–5 min with 5% B, (ii) 5–6 min to 30% B, (iii) 6–25 min to 40% B, (iv) 25–100 min to 55% B, (v) 100–102 min to 100% B, (vi) 102–107 min hold 100% B, (vii) 107–108 min to 5% B, and (viii) 108–113 min hold 5% B. The flow rate was kept at 0.8 mL/min for the duration of the 113 min gradient and each injection consisted of 100 μL of 2 mg/mL total histones. For offline LC analysis, fractions were collected, dried, and resuspended in a 1:1 methanol:water buffer with 0.1% formic acid.

An Apolo II ESI source operated with a 0.4 ml/min flow rate in positive mode was used for online LC analysis. The ESI source nitrogen nebulizer, dry gas, and temperature were 3 bar, 10 L/min, and 225°C with capillary and end plate voltages of 4500 and 500 V, respectively. A custom-built pulled tip nanoESI source, with similar performance to commercial nanoESI sources, was used for direct infusion analysis (offline LC) at 600–1200 V mounted on an XYZ stage. TIMS fundamentals and principles of operation have been previously described [52–54]. Briefly, mobility experiments were carried out using nitrogen (N_2_) as the buffer gas at ambient temperature with a gas velocity defined by the TIMS funnel pressure differences (*P*_1_ = 2.5 mbar and *P*_2_ = 0.68 mbar). Trapped ions were radially confined using a rf voltage of 250 Vpp at 880 kHz. Deflector and base voltages of 135 and 60 V and a mobility ramp of −150 to −15 V over 100 ms were used. A custom-built 19 mm long electro-magnetostatic cell (EMS from e-MSion Inc., Corvallis, OR) was used [22, 29]. A 2.5 A EMS filament current was used and the collision cell was operated with high-purity argon (oxygen-free). While the experiments described were performed on a custom-built tims-qTOF MS platform, alternative capabilities are available using the integration of front-end IMS analyzers and ExD capabilities using third-party MS companies. Additional information regarding MS instrument operation can be found in Supplementary Fig. S3 in the supporting information.

### Histone proteoform derivatization for bottom-up MS

Bottom-up MS histone samples were derivatized using propionic anhydride, as previously described (Supplementary Fig. S4) [21, 24, 25, 55, 56]. Briefly, dried extracted histones were reconstituted in 100 mM NH_4_CO_3_ (pH 8) to 1 μg/μL. Propionylation reagent (1:3 v/v propionic anhydride in ACN) was added to the samples (1:4 v/v), followed quickly by NH_4_OH to maintain a pH of 8. The reaction was incubated at room temperature for 15 min, then repeated before drying the samples in a vacuum centrifuge. Derivatized samples were reconstituted in 100 mM NH_4_CO_3_ to a final concentration of 1 μg/μL and enzymatically digested using trypsin (1:10 wt/wt) [56] at room temperature overnight. The enzymatic activity was ceased by freezing the samples at −80°C for at least 1 h. Proteolytic digests were thawed and dried using a vacuum centrifuge, then resuspended to 1 μg/μL using 100 mM NH_4_CO_3_. The derivatization procedure was repeated to label the newly generated peptide N-terminals. Samples were desalted using homemade C_18_ stage-tips [26]. The stage-tips were quenched using ACN, followed by equilibration with 0.1% TFA. The pH of the samples was adjusted using glacial acetic acid (pH < 4), samples were loaded onto C_18_ stage-tips, washed with 0.1% TFA, and eluted using 0.5% acetic acid in 75% ACN. Samples were dried using a vacuum centrifuge, resuspended in the mobile phase A, and spiked with custom histone-like QC peptides to monitor the analytical reproducibility of the bottom-up MS analysis.

### Bottom-up MS histone analysis

A nanoLC system (nanoElute 2) fitted with a C_18_ column (15 cm × 150 μm i.d., 1.5 μm, Bruker PepSeq column) kept at 50°C was connected to a commercial timsTOF Pro2 mass spectrometer (Bruker Daltonics, MA). A sample injection consisted of 1 μL of 200 ng/μL derivatized histone digest spiked with 25 ng/μL QC peptides. The nanoLC separation consisted of A (0.1% FA in water) and B (0.1% FA in 100% ACN) mobile phases with the following gradient: (i) 0 min 2% B, (ii) 0–60 min to 35%, (iii) 60–69 min to 95% B, and (iv) 70–78 to 2% B. A nanoESI source (Captive Spray, Bruker Daltonics MA) operated at 1200 V with a flow rate of 500 nL/min was used. Tandem CID MS/MS was performed on mobility and *m/z* selected precursor ions (PASEF mode) over the 100–1700 *m/z* and 0.60–1.80 1/K_0_ range; CID collision energy was stepped as a function of the *m/z* and mobility [24, 26]. Additional information regarding MS instrument parameters can be found in Supplementary Fig. S3 in the supporting information.

### Data analysis

Top-down LC-TIMS-MS and TIMS-q-ECD MS/MS data were analyzed using DataAnalysis software (DA, v6.1, Bruker Daltonics) and OmniScape 2025 software (OSc, Bruker Daltonics). A Tuning Mix standard (G24221A, Agilent Technologies, Santa Clara, CA) was used for external mobility and *m/z* calibration (see instrument parameters in Supplementary Fig. S3). Tandem q-ECD MS/MS spectra were processed for *de novo* sequencing with a 15 ppm mass error; the charge, neutral monoisotopic mass, and noise curve offset were determined for each data set. The UniProtKB [57] reference proteomes and Swiss-Prot databases were used with no species bias for the MS-BLAST [58] sequence similarity search. The high abundance of PTMs in the endogenous coral samples relative to unmodified proteins (only search considered in the *de novo* and MS-BLAST algorithms) required added manual annotation and confirmation of the proposed proteoforms, which was performed using a mass error of 10 ppm (Supplementary Figs S5–S49). For example, annotated MS/MS spectra of the more abundant proteoforms can be found in Supplementary Figs S50–S55 in the supporting information; note that all fragment assignments were manually curated.

Bottom-up LC-TIMS-q-CID MS/MS data were analyzed using a script (Supplementary Fig. S56) in DataAnalysis software (DA, v6.1, Bruker Daltonics) for the extraction and quantification of a predetermined peptide target list with varying PTMs (me_1–3_, ac, and ox). The derivatization and MS analysis reproducibility in the bottom-up workflow were evaluated using the internal, custom QC synthetic peptide standard (GVKFRGSTGGKAPRGKAPATSGMVGPHR), which, when subjected to propionylation, digestion, and desalting procedures, as described in Supplementary Fig. S4, results in the targeted screening for QC1 (GSTGGKAPR, 471.75^2+^) and QC2 (GKAPATSGMVGPHR, 739.38^2+^). The hexakis(2,2-difluoroethoxy) phosphazine, hexakis(2,2,3,3-tetrafluoropropoxy) phosphazine, and hexakis(1H, 1H, 7H-dodecafluoroheptoxy) phosphazine standards (Apollo Scientific Ltd, UK) were used for external mobility and *m/z* calibration (see instrument parameters in Supplementary Fig. S3). All reported peptides and PTMs (Supplementary Figs S57–S77) were manually curated using the precursor mass (± 0.01 Da) and isotopic profile, reported peptide mobility profiles (including positional isomers, RSD < 2%) [21], and MS/MS product ion assignment (<10 ppm).

Proteoform sequence confirmation was performed and evaluated based on the sequence coverage (%SC), sequence validation percentage (%SVP) [59], intensity coverage (%IC), and MS score (MS):


(1) \begin{eqnarray*}
\% SC = \ \frac{{number\ of\ amino\ acids\ confirmed}}{{total\ number\ of\ amino\ acids}} \times 100\% ,
\end{eqnarray*}

(2) \begin{equation*}
\% SVP = \ \frac{{continuous\ amino\ acids\ confirmed\ from\ terminals\ \left( {within\ tolerance} \right)}}{{total\ number\ of\ amino\ acids}} \times 100\% \nonumber\\ ,
\end{equation*}

(3) \begin{eqnarray*}
\% IC = \ \frac{{intensity\ of\ matched\ fragment\ peaks}}{{intensity\ of\ all\ peaks}} \times 100\% ,
\end{eqnarray*}

(4) \begin{eqnarray*}
MS\ score = \% SC \times \% IC.
\end{eqnarray*}

The relative abundance of top-down isomeric proteoforms was calculated based on the intensity of reporter ions specific to each isomer and normalized to the area of the integrated MS^1^ spectra using Equation 5:


(5) \begin{equation*}
Relative\ abundance = \ \frac{{intensity\ of\ selected\ isomer\ reporter\ ion\left( s \right)}}{{combined\ intensity\ of\ all\ isomeric\ reporter\ ions}} \times M{{S}^1}\ area\nonumber\\ .
\end{equation*}

## Results

### Coral tissue histone pull-down and extraction

**Figure 1.**
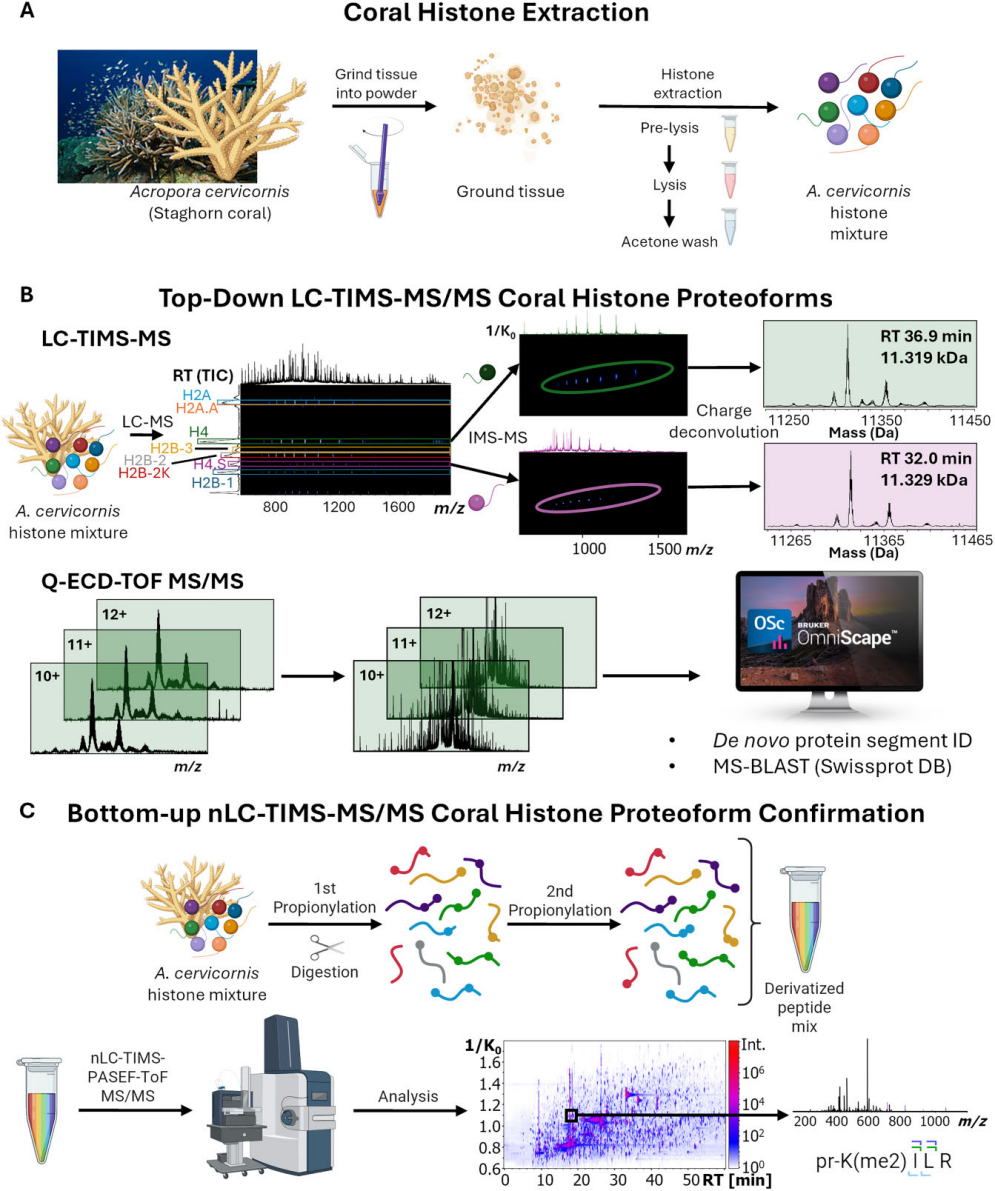
Direct protein sequence generation and proteoform PTM annotation based on liquid chromatography (online and offline), TIMS, ECD fragmentation, and high-resolution MS for *A. cervicornis*. (**A**) Histone extraction from *A. cervicornis* coral. (**B**) Top-down identification of histone variants with subsequent ECD-MS/MS and *de novo* sequencing. (**C**) Bottom-up nLC-TIMS-MS/MS peptide confirmation.

The coral tissue histone extraction (Fig. 1A) resulted in ∼5 μg protein mass per 100 mg of powdered coral fragments (tissue + skeleton). Several bands were observed in the SDS–PAGE analysis; the most abundant bands suggested the presence of histone families. At a lower abundance, other acid-soluble nuclear proteins were also observed (Supplementary Fig. S78A).

### Coral histone primary sequences and PTM annotation

The analysis of the pooled *A. cervicornis* histones using LC-TIMS-MS confirmed several histone families (see Supplementary Fig. S78). Inspection of the LC-derived 2D IMS-MS plots allowed for the separation of the histone variants based on their retention time (Fig. 1B). The endogenous histone variants showed a high degree of PTMs; different from traditional top-down methods, a better MS identification was achieved due to the mobility separation of the charge state distribution (CSD) of interest from potential mass interferences (e.g. co-eluting proteins) and reduced chemical noise. Two proteins with a ∼11.3 kDa mass at retention times (RT) of 32.0 min (11.250–11.432 kDa mass range) and 36.9 min (11.234–11.442 kDa mass range) were flagged for further analysis as potential H4 variants (Fig. 1B); in addition to the mass, the 36.9 min RT LC band is consistent with the observation of the human (HeLa) H4 histone (Supplementary Fig. S78B). Four proteins with a ∼13.3–13.5 kDa mass at RT 30.1 min (13.401–13.470 kDa mass range), 33.8 min (13.355–13.464 kDa mass range), 35.2 min (13.421–13.484 kDa mass range), and 45.9 min (13.408–13.540 kDa mass range) were also flagged as potential H2A/H2B variants (Supplementary Fig. S78C). The CSD and deconvoluted MS showed distinctive mass shifts corresponding to multiple known PTMs (e.g. me_1–3_ with a +14.02, +28.03, and +42.05 Da, ac with +42.01 Da, and ox with +15.99 Da per modified amino acid and/or N-term; Supplementary Fig. S79). Additionally, mass shifts were observed in the 33.8 min and 45.9 min RT bands, which did not correspond to common PTMs (+28.01 and +26.27 Da, respectively).

**Figure 2.**
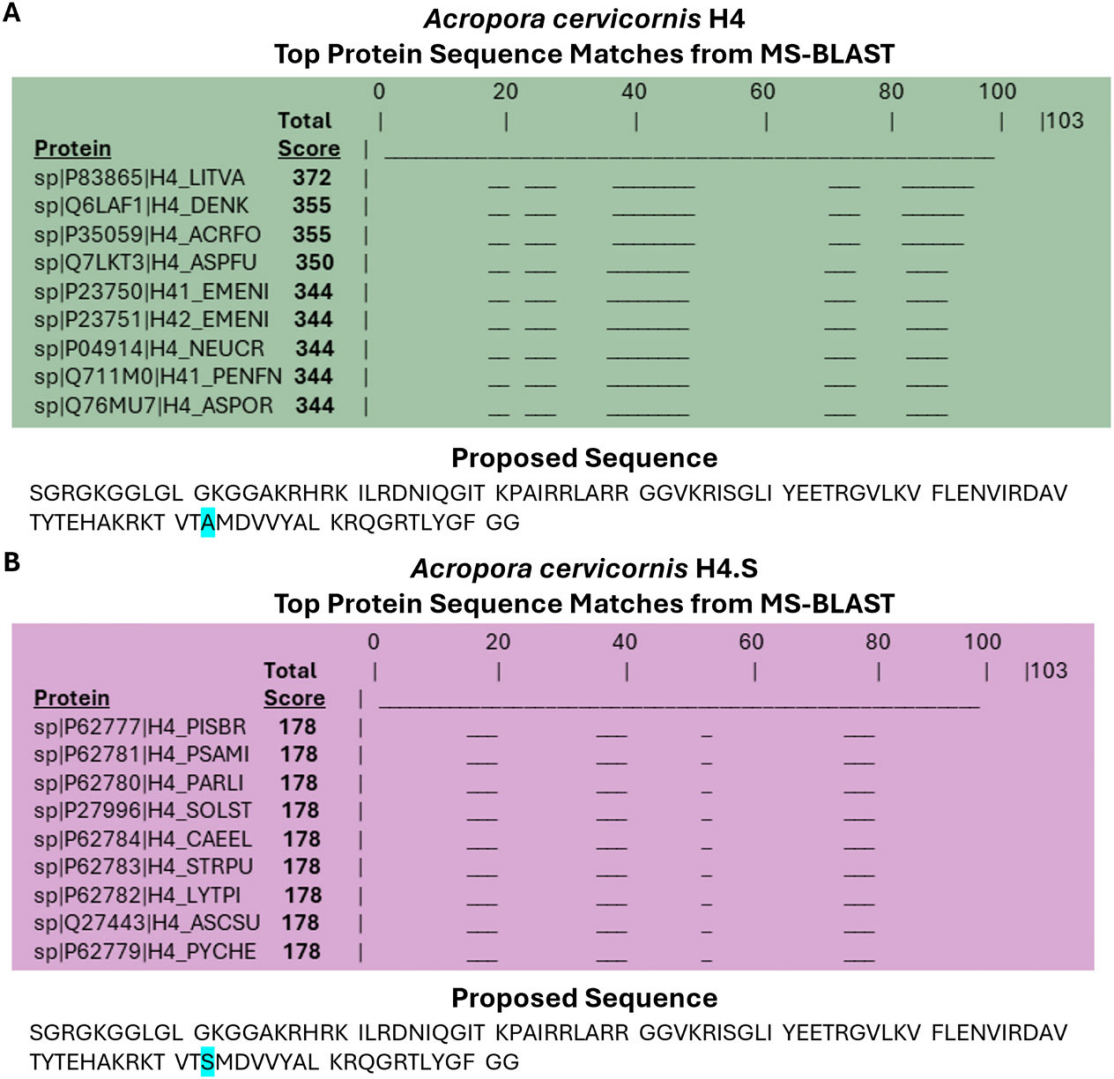
MS-BLAST results for *A. cervicornis* 32.0 and 36.9 min fractions. (**A**) *A. cervicornis* H4 and (**B**) H4.S top 9 matching proteins found in the SwissProt database with total scores and mapped protein segments, and proposed sequences for each fraction. The single amino acid variation A83S is highlighted.

*De novo* sequencing of H4 histone variants. Tandem ECD MS/MS on mobility and mass-isolated precursor ions showed abundant fragmentation for the most abundant charge states: +10 to +12 and +11 to +13 for 32.0 min and 36.9 min RT LC bands, respectively (Fig. 1B). The *de novo* sequencing of the ECD-MS/MS spectra generated a list of AA strings: ∼150 and ∼300 (Supplementary Fig. S80) for the 32.0 and 36.9 min RT LC bands, respectively. The MS-BLAST database [58] search of the AA strings resulted in the identification of high-scoring pairs (HSPs; query sequences producing significant local alignments to a database sequence) matching H4 sequences from various organisms (Fig. 2 and Supplementary Fig. S80). High-scoring pairs are assigned a score by MS-BLAST based on the length and AA matches of the query (unknown) sequence to a sequence from the database being searched (e.g. SwissProt). Ambiguities such as unclear AA ordering or AAs with the same or similar masses (i.e. I/L and K/Q, respectively) in either the query or database sequences may also bias the HSP score for a given AA string. The highest total score (sum of HSP scores as determined by MS-BLAST) was for *Penaeus vannamei* (a.k.a *Litopenaeus vannamei*) or *Whiteleg shrimp* (Protein ID P83865) with 372; the ambiguity of the 92–100 protein segment partially biases the score. The next high-scoring H4 proteins were *Dendronephthya klunzingeri* (Q6LAF1) and *Acropora formosa* (P35059) with 355 and less ambiguity in the segments. Considering that *A. formosa* shares the genus and ancestry with *A. cervicornis*, this H4 sequence was used as the starting point for further analysis.

*De novo* sequencing of H2A and H2B histone variants. Tandem ECD MS/MS was performed on the most abundant charge states for each RT fraction: +14 to +16 for the 30.1, 33.8, and 35.2 min RT bands and + 12 to +14 for the 45.9 min RT band (Supplementary Fig. S81). *De novo* sequencing generated a list of amino acid (AA) strings: ∼500, ∼400, ∼500, and ∼600 for the 30.1, 33.8, 35.2, and 45.9 min RT LC bands, respectively (Supplementary Figs S82A–S85A). The MS-BLAST database [58] search resulted in HSPs from either H2B (30.1, 33.8, and 35.2 min RT band) or H2A (45.9 min RT band) sequences from various organisms (Supplementary Figs S82B–S85B). The highest-scoring protein matched for the H2A fraction was *A. formosa* (Protein ID P35061), a closely related coral from the same genus with a total score of 228 (Supplementary Fig. S85C). The same case was seen for the 33.8 min and 35.2 min H2B fractions (Protein ID P35067), which had total matching scores of 393 and 148, respectively (Supplementary Figs S83C and S84C).

### *Acropora cervicornis* H4 variants

Combining the information at the protein level (e.g. potential PTMs), the *de novo* results, and the manually annotated PTMs, a candidate proteoform annotation list was established for each RT band (see the proposed sequences in Fig. 2). For example, the N-terminal amino acids and PTMs were sequenced manually and suggested that the N-term was observed acetylated for most proteoforms in both H4 LC bands (based on the +42.01 Da mass shifts of the first 3–5 AAs corresponding to the *c*_3_and *c*_3–5_, *b*_3_ fragments in the 32.0 min and 36.9 min spectra, respectively). The most abundant mass signal of the 32.0 and 36.9 min RT LC bands corresponded to N-acSK20me_2_, confirmed by the additional mass shift observed from mass fragments of the first 23 AAs. (e.g. mass shifts of +28.03 observed in *c*_20,21,23_*and c*_20–23_, *a*_21_ fragments, respectively). The difference in the sequences from the two LC bands was derived from discrepancies toward the C-terminal fragment assignment and resulted from a single amino acid variation of A83 to S83 (A83S); this AA position is commonly seen mutated in the H4 histones (see H4 sequences listed in Supplementary Figs S80 and S86). The H4 *A. cervicornis* variant proposed (RT 36.9 min LC band) has a 100% homology with the H4 *A. formosa* sequence and no less than 92% with the other listed H4 sequences. The second H4 variant (RT 32.0 min LC band), now referred to as H4.S *A. cervicornis* has a 99% homology with the H4 *A. formosa* sequence. An all-versus-all comparison of the established H4 *A. cervicornis* sequence and the 9 selected matches from MS-BLAST showed that <12% of the AA positions in H4 are prone to mutations between species/variants.

**Figure 3.**
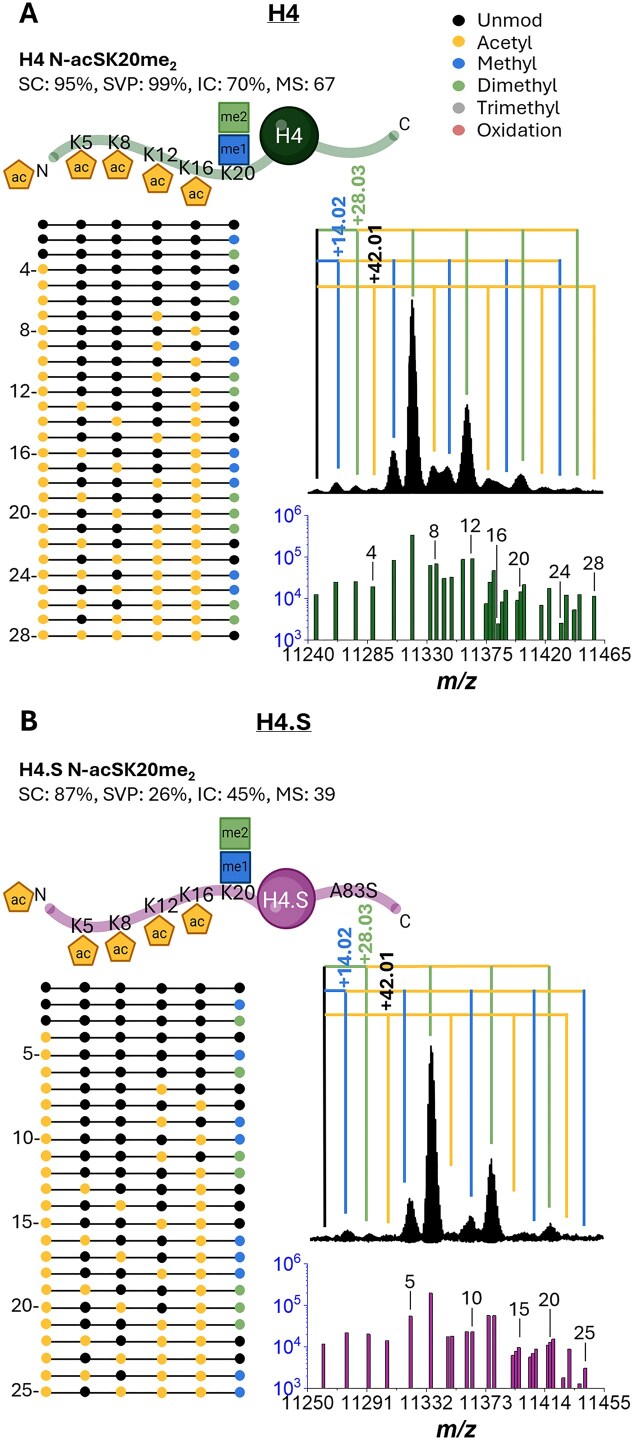
*Acropora cervicornis* histone H4 detected variants and corresponding proteoforms. (**A**) H4 and (**B**) H4.S variants with PTM positions shown with specific PTM combinations below (dotted lines; left). The deconvoluted MS1 spectra, labeled with mass shifts corresponding to the observed PTM(s) (branched lines, colored according to the PTM represented by the observed mass shift from the unmodified mass), and bar plot (right), showing the relative abundances of each of the observed proteoforms with numbered labels corresponding to the PTM combinations shown (dotted lines). Details on sequence coverage at the protein and peptide levels are provided in supplementary data.

The H4 *A. cervicornis* top-down analysis resulted in the annotation of 28 proteoforms with me_0–2_ and ac_0–4_ and a high sequence coverage range (80%–95%), sequence validation percentage (up to 99%), intensity coverage range of 6%–70%, and MS score range of 5.5–66.9 (see relative abundances in Fig. 3A and MS/MS statistics in Supplementary Figs S5–S18 and Supplementary Table S1). The first six mass signals in the MS (RT 36.9 min LC band) correspond to unmodified, K20me_1_, K20me_2_, N-acS, N-acSK20me_1_, and N-acSK20me_2_ proteoforms. The next mass signals correspond to a mass shift of 2ac (N-acSK12ac and N-acSK16ac), 2ac + me_1_ (N-acSK12acK20me_1_ and N-acSK16acK20me_1_), and 2ac + me_2_ (N-acSK12acK20me_2_ and N-acSK16acK20me_2_); the ratio of K12ac to K16ac in these proteoforms was nearly equal. The next mass signals correspond to 3ac (N-acSK5acK16ac, N-acSK8acK16ac, and N-acSK12acK16ac), 3ac + me_1_ (N-acSK5acK16acK20me_1_, N-acSK8acK16acK20me_1_, and N-acSK12acK16acK20me_1_), and 3ac + me_2_ (N-acSK5acK16acK20me_2_, N-acSK8acK16acK20me_2_, and N-acSK12acK16acK20me_2_). The following three mass signals correspond to 4ac (N-acSK5acK12acK16ac and N-acSK8acK12acK16ac), 4ac + me_1_ (N-acSK5acK12acK16acK20me_1_ and N-acSK8acK12acK16acK20me_1_), and 4ac + me_2_ (N-acSK5acK12acK16acK20me_2_ and N-acSK8acK12acK16acK20me_2_). The next mass signal corresponded to 5ac (N-acSK5acK8acK12acK16ac), observed at lower levels than the other acetylated proteoforms.

The H4.S *A. cervicornis* top-down analysis resulted in the annotation of 25 proteoforms with me_0–3_ and ac_0–4_ and a high sequence coverage (70%–87%), sequence validation percentage up to 30%, intensity coverage range of 6%–45%, and MS score range of 4.6–39.2 (see relative abundances in Fig. 3B and MS/MS statistics in Supplementary Figs S19–S31 and Supplementary Table S1). The first six mass signals in the MS (RT 32.0 min LC band) correspond to unmodified, K20me_1_, K20me_2_, N-acS, N-acSK20me_1_, and N-acSK20me_2_. The next mass signals correspond to a mass shift of 2ac (N-acSK12ac and N-acSK16ac), 2ac + me1 (N-acSK12acK20me_1_ and N-acSK16acK20me_1_), and 2ac + me2 (N-acSK12acK20me_2_ and N-acSK16acK20me_2_). The next mass signals correspond to a mass shift of 3ac (N-acSK5acK16ac, N-acSK8acK16ac, and N-acSK12acK16ac), 3ac + me1 (N-acSK5acK16acK20me_1_, N-acSK8acK16acK20me_1_, and N-acSK12acK16acK20me_1_), and 3ac + me2 (N-acSK5acK16acK20me_2_, N-acSK8acK16acK20me_2_, and N-acSK12acK16acK20me_2_). The next mass signals correspond to 4ac (N-acSK5acK12acK16ac and N-acSK8acK12acK16ac) and 4ac + me_1_ (N-acSK5acK12acK16acK20me_1_ and N-acSK8acK12acK16acK20me_1_).

### *Acropora cervicornis* H2A variants

**Figure 4.**
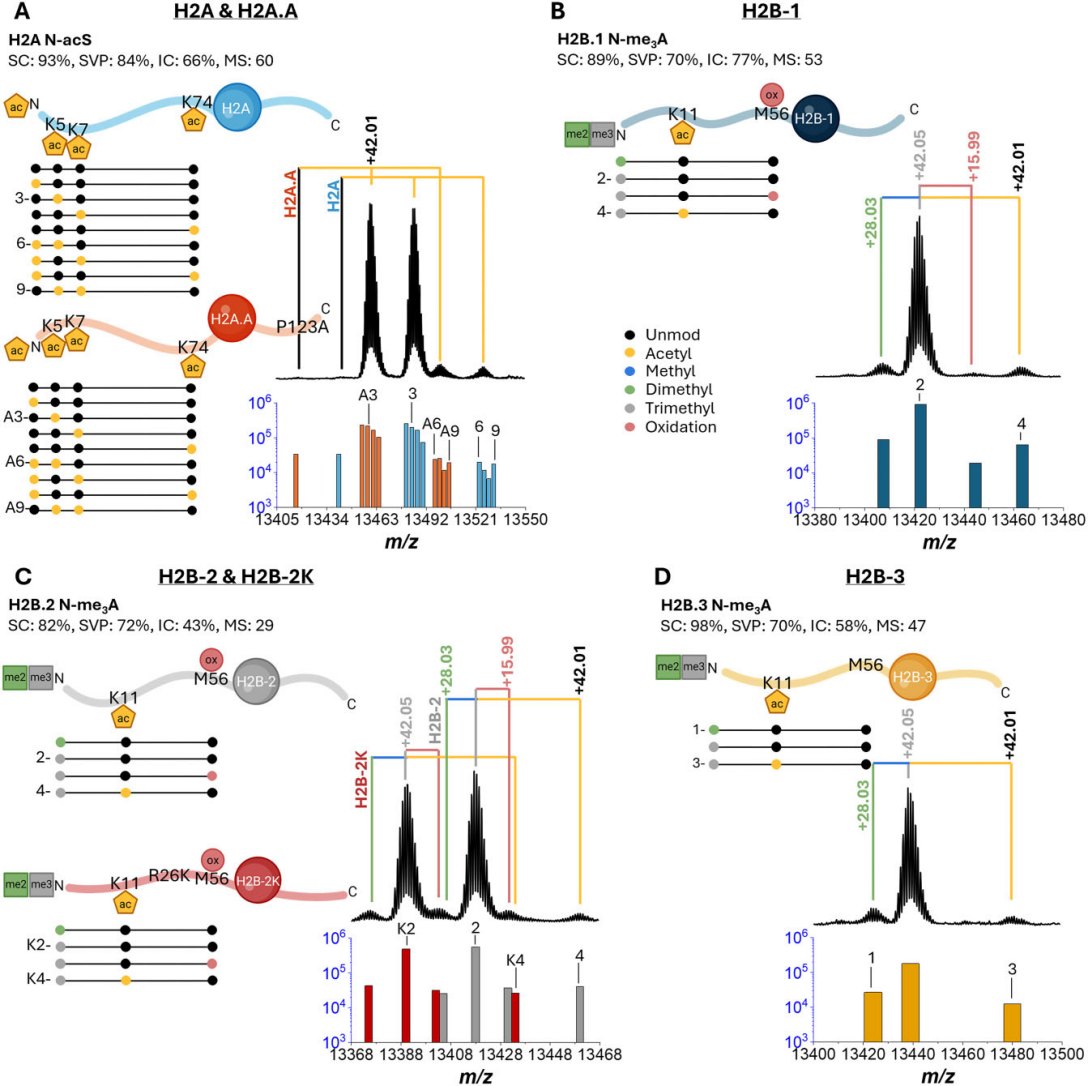
*Acropora cervicornis* histone H2A and H2B detected variants and corresponding proteoforms. (**A**) H2A & H2A.A, (**B**) H2B-1, (**C**) H2B-2 & H2B-2K, and (**D**) H2B-3 variants with PTM positions shown with specific PTM combinations below (dotted lines; left). The deconvoluted MS1 spectra, labeled with mass shifts corresponding to the observed PTM(s) (branched lines, colored according to the PTM represented by the observed mass shift from the unmodified mass), and bar plot (right), showing the relative abundances of each of the observed proteoforms with numbered labels corresponding to the PTM combinations shown (dotted lines). Details on sequence coverage at the protein and peptide levels are provided in supplementary data.

The H2A N-terminal AAs and PTMs were sequenced manually, leading to the suggestion that the N-terminal was primarily observed to be acetylated based on the +42.01 Da mass shift of the first 3–5 AAs corresponding to the *c*_3–5_, *b*_3,5_, and *a*_3,5_ fragment ions (Fig. 4A). In the case of the H2A band (45.9 min RT), a mass shift between the two most abundant MS peaks of +26.27 Da was observed (Supplementary Fig. S79F). This same mass shift was observed between other peaks in the distribution and is not a common mass shift among known PTMs. Upon further inspection, the mass shift was found to be derived from a C-terminal single amino acid variation of P123 to A123 (P123A), indicating the presence of two coeluting H2A variants (referred to as H2A and H2A.A for the variants containing P123 and A123, respectively, from this point forward). In particular, the presence of reporter ions *a*_124_, *c*_123–124_, and *y*_3_ corresponding to the P123 in the H2A N-acS proteoform (92.74% SC, 84.00% SVP, 65.77% IC, 59.94 MS) and ions *a*_124_, *b*_124_, *c*_124_, and *y*_3_ corresponding to the A123 in the H2A.A N-acS proteoform (89.52% SC, 72.80% SVP, 39.92% IC, 34.45 MS) was used to confirm the presence of both variants. The C-terminal of H2A proteins commonly mutates between and within species, as suggested by the matching H2A sequences found by the *de novo* MS-BLAST search (Supplementary Fig. S85D). The most abundant signal from both variants corresponded to N-acS, as previously determined by N-terminal fragments from the first 3–5 AAs and sequence coverages of ∼93% and ∼90% achieved for variants H2A and H2A.A, respectively (Fig. 4A). The proposed H2A *A. cervicornis* variants both demonstrate a 93% homology match to the H2A *A. formosa* sequence (top MS-BLAST result); however, homology ranges from 70% to 90% to the remaining 9 top matching BLAST sequences (Supplementary Fig. S85D). An all-versus-all comparison of the proposed sequences to the top 10 MS-BLAST results suggests that up to 40% of the AA positions in H2A are prone to mutations or insertions/deletions between species and/or variants.

The H2A/H2A.A *A. cervicornis* combined top-down and bottom-up analysis resulted in the annotation of 18 proteoforms (9 per variant) with a high sequence coverage (68%–93% and 67%–90%), sequence validation percentage ranges of 38%–84% and 44%–73%, intensity coverage ranges of 13%–66% and 8%–40%, and MS score ranges of 9.5–60.0 and 4.9–34.4 for H2A and H2A.A, respectively (see relative abundances in Fig. 4A and MS/MS statistics in Supplementary Figs S32–S41 and Supplementary Table S1). The H2A.A and H2A unmodified mass signals are followed by two much higher (two orders of magnitude) signals corresponding to H2A.A and then H2A 1ac (N-acS, K5ac, K7ac, and K74ac) with consistent ratios of each proteoform between variants. The next mass signals correspond to 2ac proteoforms for each variant (N-acSK5ac, N-acSK7ac, N-acSK74ac, and K5acK7ac). The ratios of each 2ac proteoform between variants are mostly consistent, except for the N-acSK7ac proteoform being slightly higher than N-acSK5ac in the H2A.A versus the H2A variant.

### *Acropora cervicornis* H2B variants

Three separate LC peaks were identified as H2B variants (30.1, 33.8, and 35.2 min RT bands). For each fraction, the *de novo* sequence strings were combined with the manual mass shift annotations detected in the CSDs at the protein level to determine proteoform candidate lists. In each case, the N-terminal AAs and PTMs were sequenced manually.

The 30.1 min RT band (referred to as H2B-1 from this point forward) was largely observed to contain trimethylation at the N-terminal based on the +42.05 Da mass shift of the first 3–6 AAs corresponding to *c*_4–6_, *b*_5,6_, and *a*_4–6_ and the presence of the *z*_120_ and *y*_121_ C-terminal ions, which confirm the K3 to be largely unmodified (Fig. 4B). Sequence coverage higher than 89% was achieved for the H2B-1 variant with the most abundant proteoform being N-me_3_A. The H2B-1 *A. cervicornis* histone was the only fraction not found to have high sequence homology to *A. formosa* in the MS-BLAST search of the *de novo* sequence strings (Supplementary Fig. S82). Homology of the protein compared to the top 10 MS-BLAST results ranged from 70% to 83% with the highest match being to *Asterias rubens* (common European starfish, Protein ID P02286). An all-versus-all comparison of the proposed sequence to the top 10 BLAST results suggested ∼36% of AAs in the H2B sequence may be prone to mutations, insertions, or deletions between species/variants (Supplementary Fig. S82D).

The 33.8 min RT band (referred to as H2B-2 from this point forward) was observed to be largely trimethylated at the N-terminal based on a +42.05 Da mass shift of the first 3–6 AAs corresponding to the *c*_4–6_ and *b*_6_ N-terminal ions and the presence of *z*_120_ and *y*_121_ C-terminal ions, indicating that K3 was largely unmodified. The CSD of the H2B-2 fraction showed a distinctive mass shift between the two most abundant peaks of +28.01 Da (Supplementary Fig. S79D). Upon inspection, this mass shift was not a result of dimethylation (+28.03 Da), but rather a single amino acid variation in the N-terminal tail of R26 to K26 (R26K), indicating two coeluting variants (referred to as H2B-2 and H2B-2K for the variants containing R26 and K26, respectively, from this point forward). The N-terminal of H2B proteins is highly variable between and within species, as shown by the matching H2B sequences found by the *de novo* MS-BLAST search (Supplementary Figs S82D–S84D). The most abundant signal from both variants corresponded to N-me_3_A proteoforms, as determined by the previously stated N-terminal fragments and sequence coverages of ∼82% and ∼79%, for H2B-2 and H2B-2K, respectively (Fig. 4C). The signal-to-noise ratio (S/N) of each charge state for this fraction was lower than the other observed H2A/B fractions, reducing the number/quality of fragment ions observed, and leading to the slightly lower sequence coverages obtained. The proposed H2B-2 *A. cervicornis* variants demonstrate 87% and 86% (H2B-2 and H2B-2K, respectively) homology matches to the H2B *A. formosa* sequence (top MS-BLAST result). Shared homology between the other 9 top BLAST matches ranges from 72% to 81% and 71% to 80% for H2B-2 and H2B-2K, respectively (Supplementary Fig. S83D). An all-versus-all comparison of the proposed sequences to the top 10 MS-BLAST results suggested that ∼39% of AAs in the H2B sequence may be prone to mutations, insertions, or deletions between species/variants (Supplementary Fig. S83D).

The 35.2 min RT band (referred to as H2B-3 from this point forward) was also observed to primarily be trimethylated at the N-terminal based on a +42.05 Da mass shift of the first 3–6 AAs corresponding to *c*_3_, *b*_4,6_, and *a*_4,6_ ions, as well as *z*_120_ and *y*_121_ C-terminal ions confirming that the K3 is unmodified (Fig. 4D). The highest sequence coverage (nearly 98%) was achieved for H2B-3 with the most abundant proteoform being N-me_3_A. This protein was observed with the fewest proteoform peaks in the MS spectrum (Supplementary Fig. S79E). The H2B-3 *A. cervicornis* histone variant had an 84% homology match to the H2B *A. formosa* protein sequence and a homology match range of 77%–80% to the remaining top 9 MS-BLAST result sequences. An all-versus-all comparison of the proposed sequence to the top 10 MS-BLAST results suggested ∼30% of AAs in the H2B sequence may be prone to mutations/insertions/deletions between species, with most of these changes observed in the N-terminal sequences (Supplementary Fig. S84D).

A total of 15 H2B variant proteoforms were annotated. The H2B-1 *A. cervicornis* combined top-down and bottom-up analysis resulted in the annotation of 4 proteoforms with a sequence coverage range of 74%–90%, sequence validation percentage range of 20%–70%, intensity coverage range of 12%–77%, and MS score range of 7.1–53.0 (see relative abundances in Fig. 4B and MS/MS statistics in Supplementary Figs S42 and S43, and Supplementary Table S1). The four MS peaks correspond to the unmodified, N-me_2_A, N-me_3_A, N-me_3_AM56ox, and N-me_3_AK1ac proteoforms. The H2B-2/2K *A. cervicornis* combined top-down and bottom-up analysis resulted in the annotation of 8 proteoforms (4 each) with sequence coverage ranges of 63%–82% and 60%–79%, sequence validation percentage ranges of 25%–72% and 26%–44%, intensity coverage ranges of 19%–43% and 8%–3%, and MS score ranges of 8.6–28.9 and 3.8–19.3 for H2B-2 and H2B-2K, respectively (see relative abundances in Fig. 4C and MS/MS statistics in Supplementary Figs S44–S47 and Supplementary Table S1). The eight annotated MS peaks correspond to the H2B-2K N-me_2_A, H2B-2K N-me_3_A, H2B-2K N-me_3_AM56ox, H2B-2 N-me_2_A, H2B-2 N-me_3_A, H2B-2 N-me_3_AM56ox, H2B-2K N-me_3_AK11ac, and H2B-2 N-me_3_AK11ac proteoforms. No unmodified H2B-2/2K was confirmed to be observed. The H2B-3 *A. cervicornis* combined top-down and bottom-up analysis resulted in the annotation of 3 proteoforms with a sequence coverage range of 85%–97%, sequence validation percentage range of 25%–70%, intensity coverage range of 17%–58%, and MS score range of 10.8–47.2 (see relative abundances in Fig. 4D and MS/MS statistics in Supplementary Figs S48 and S49, and Supplementary Table S1). The three MS peaks correspond to the N-me_2_A, N-me_3_A, and N-me_3_AK11ac proteoforms. No unmodified H2B-3 could be confirmed to be observed.

### Bottom-up histone proteoform confirmation

Bottom-up nLC-TIMS-PASEF-ToF MS/MS analysis provided extended confirmation of H4, H2A, and H2B sequences based on LC RT, mobility (1/K_0_), and tandem MS/MS fragmentation (Fig. 1C and Supplementary Table S2).

**Figure 5.**
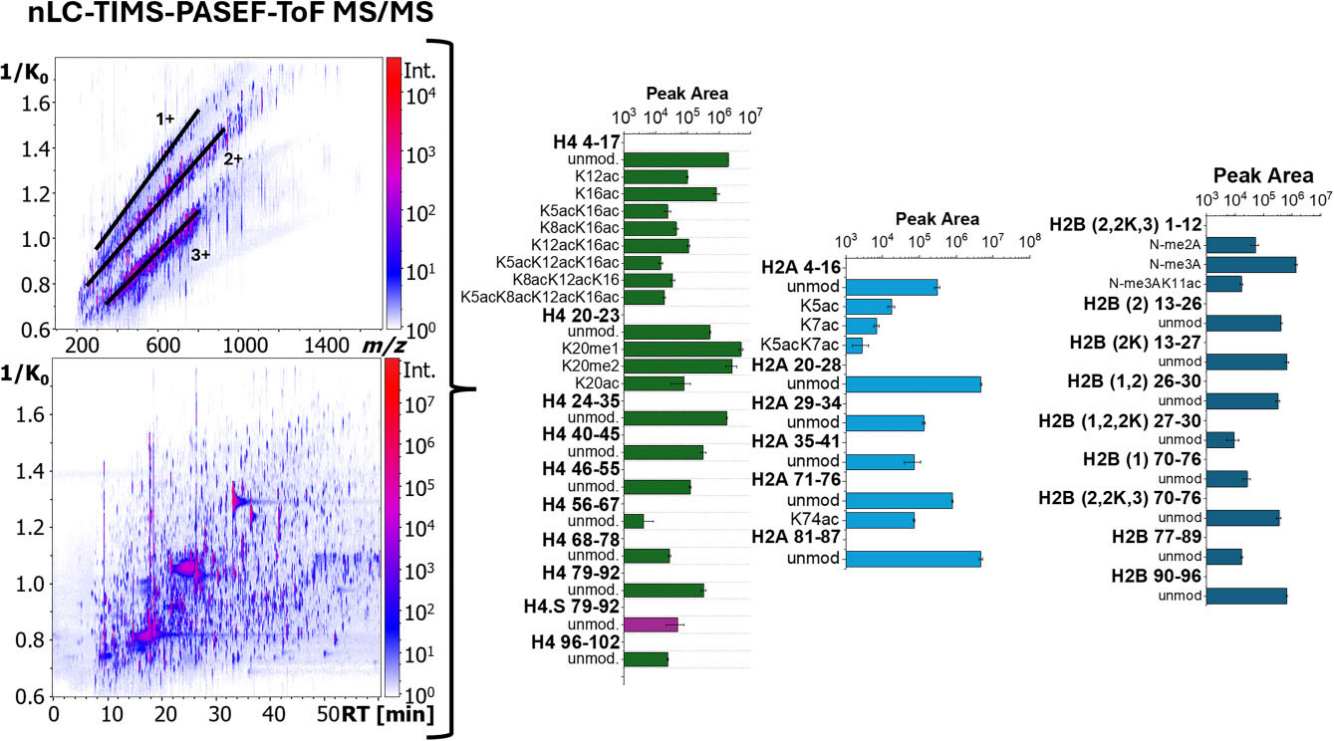
Bottom-up confirmation of histone peptides using nLC-TIMS-PASEF-ToF MS/MS. Detected peptides for H4, H2A, and H2B variants with relative abundances.

H4 *A. cervicornis* bottom-up confirmation. Over 21 peptides were identified and confirmed corresponding to: (i) sequence-confirming H4 24–35, 40–45, 46–55, 56–67, 68–78, and 96–102; (ii) variant-confirming H4 79–92 and H4.S 79–92; and (iii) PTM-confirming H4 4–17 and 20–23 peptides (Supplementary Figs S57–S77). The H4 4–17 peptide was detected with ac_0–4_ and a sequence coverage range of 46%–92%, intensity coverage range of 4%–32%, and MS score range of 2.5–29.5 (see relative abundances in Fig. 5 and Supplementary Fig. S87 and MS/MS statistics in Supplementary Figs S57–S77 and Supplementary Table S2). The H4 4–17 peptide was detected in the unmodified, 1ac (K12ac and K16ac), 2ac (K5acK16ac, K8acK16ac, and K12acK16ac), 3ac (K5acK12acK16ac and K8acK12acK16ac), and 4ac (K5acK8acK12acK16ac) forms. Additionally, H4 20–23 was detected with me_0–2_ and ac_0–1_ and a sequence coverage range of 33%–100%, intensity coverage range up to 51%, and MS score range of 0.6–51.4 (see relative abundances in Fig. 5 and Supplementary Fig. S87, and MS/MS statistics in Supplementary Figs S57–S77 and Supplementary Table S2). The H4 20–23 peptide was detected in the unmodified, K20me_1_, K20me_2_, and K20ac forms.

H2A *A. cervicornis* bottom-up confirmation. A total of 10 H2A peptides were identified and confirmed. The H2A and H2A.A unmodified peptides 4–16, 20–28, 29–34, 35–41, 71–76, and 81–87 were able to confirm shared sequence segments between the two variants with sequence coverage ranging from 20% to 100%, intensity coverage of up to 77%, and MS scores of 0.3–71.3 (see peptide abundances in Fig. 5, and Supplementary Fig. S88 and MS/MS statistics in Supplementary Table S2). The H2A/H2A.A 4–16 peptide was detected with up to two acetylations at positions K5 (average 55% SC, 15% IC, 9.2 MS score), K7 (average 61% SC, 16% IC, 10.1 MS score), or both (no MS/MS available; see relative abundances in Fig. 5 and Supplementary Fig. S88). Additionally, acetylation was observed at position K74 in the H2A/H2A.A 71–76 peptide (average 27% SC, 49% IC, 14.8 MS score). The C-terminal 88–125 peptide was not observed for either variant.

H2B *A. cervicornis* bottom-up confirmation. A total of eleven H2B peptides were identified and confirmed. The common H2B peptides 77–89 and 90–96 were observed in the unmodified forms with averaged sequence coverages of 66% and 50%, intensity coverages of 16% and 67%, and MS scores of 10.9 and 33.8, respectively (see peptide abundances in Fig. 5, and Supplementary Fig. S89 and MS/MS statistics in Supplementary Table S2). The H2B-2/2K/3 1–12 was observed in the N-me_2_A (average 15% SC, 13% IC, 2.8 MS score), N-me_3_A (average 48% SC, 9% IC, 5.9 MS score), and N-me_3_AK11ac (average 33% SC, 54% IC, 15.4 MS score) forms (see relative abundances in Fig. 5 and Supplementary Fig. S89). The H2B-2 13–26 peptide was observed in the unmodified form (average 66% SC, 17% IC, 12.1 MS score), as was the H2B-2K 13–27 peptide (average 69% SC, 20% IC, 14.5 MS score). The H2B-1/2 26–30 (average 41% SC, 2% IC, 0.7 MS score) and H2B-1/2/2K 27–30 (average 33% SC, 4% IC, 1.3 MS score) peptides were observed as unmodified, as well. The H2B-1 70–76 (average 61% SC, 63% IC, 38.9 MS score) and H2B-2/2K/3 70–76 (average 77% SC, 50% IC, 34.2 MS score) peptides were also observed only as unmodified. The N-terminal peptide for H2B-1 was not observed; peptides in the range of 31–69 and the C-terminal 97–122 common peptide were also not observed.

## Discussion

Direct analysis of histone proteoforms based on liquid chromatography (online and offline), mobility separation, and ECD MS/MS resulted in a high-confidence, direct alternative to homology modeling to annotate *A. cervicornis* histone proteoforms. The high confidence of the method relies on improved top-down precursor ion isolation (i.e. LC, IMS, and MS) and non-ergodic fragmentation (ECD MS/MS) leading to high primary sequence coverage (over 89%) based on *de novo* sequence prediction and database screening; when complemented with bottom-up approaches, near full sequence coverage was obtained. This resulted in a method capable of effective characterization of histone protein variants by generating the primary sequence for unknown histones, with the added benefit of detecting the PTMs at the proteoform level. When applied to the non-model organism, *A. cervicornis*, with little to no genomic reference information, this is the first report of the H4, H2A, and H2B core histone sequences and PTMs observed under stressed conditions. The method was effectively applied to the most abundant MS1 signals corresponding to H4, H2A, and H2B histones. Histones H1 and H3 were observed at the MS1 level at lower intensities and may require sample enrichment alternatives, which will be described in a follow-up publication.

The *A. cervicornis* histone proteoforms showed a high diversity of PTMs, including acetylation, oxidation, mono-, di-, and tri-methylation, as well as positional isomers, not distinguishable using traditional bottom-up MS proteomics workflows. Two H4 (53 proteoforms) were identified with up to ∼95% sequence coverage, two H2A variants (18 proteoforms) were identified and sequenced with up to ∼93% sequence coverage, and four different H2B *A. cervicornis* variants (15 proteoforms) were sequenced with up to nearly 98% coverage with little to no genomic reference material (see full table of proteoforms in Supplementary Table S1). The sequence coverages were reported after manual curation, and an average fragment false discovery rate of ∼16% was calculated by comparing the number of total fragments identified by OSc to the number of final curated fragments. Additionally, sequence validation percentage is biased toward the N- and C-terminals, occasionally resulting in an SVP cutoff when terminal ions are absent or there is a break in sequence coverage, despite a high total sequence coverage and an average fragmentation efficiency of ∼47%. Inspection of the H4 PTM patterns agrees with previously reported acetylation patterns (“zip model” [60]), where the H4 4–17 peptide acetylation decreases in abundance from K16 to K5. Unique to this approach is the direct identification of histone sequence segments and their validation based on MS and MS/MS annotation and sequence homology. A current caveat on proteoform discovery is the lack of PTMs in the existing data processing algorithms, which is accentuated in the case of endogenous samples where the most abundant mass signals deviate from the unmodified protein, which in some cases was not present at all. Results showed that in cases where the unmodified protein was observed, it was typically two orders of magnitude lower than the most abundant mass signals. The top-down MS proteoform annotation showed good agreement with the bottom-up MS results—all the H4 and H4.S sequence-confirming and variant-confirming peptides were detected, and several H2A and H2B sequence and PTM-confirming peptides were observed (see Fig. 5 and Supplementary Figs S87–S89). Moreover, complete proteoform annotation was achieved using top-down MS proteomics, partially due to the reduced chemical noise provided by the complementary mobility separation. The signal-to-noise in IMS-MS experiments was ∼3× higher than that of the LC-MS (see Supplementary Fig. S90 for LC-MS and IMS-MS S/N comparisons). While histones are presumed to be partially conserved (typically <12% AA variation for H4 and up to 40% for H2A/H2B), the histone variability across species can benefit from these methods to account for AA mutations, insertions/deletions, and PTMs. The described method can be expanded beyond histones. Moreover, when applied to corals, inspection of the histone annotations and diversity demonstrates high chemical diversity in proteins that are traditionally considered conserved. The effect of amino acid variations and PTMs on the resistance and adaptability of corals to the environment can be addressed at a more detailed and systematic epigenetic level. Even within a single species, a population of environmentally stressed organisms can exhibit several variants and proteoforms of the same protein as mutations, genetic splicing, and epigenetic mechanisms act in favor of survival. Special to the method is the ability to predict and annotate any proteoform, particularly when applied to histone families highly prone to PTMs. When applied to epigenetic studies of non-annotated, endangered species (e.g. corals), in the context of climate change and phenotypic responses, this method can support intervention strategies to increase species resilience for successful conservation and restoration efforts [3].

## Supplementary Material

gkaf740_Supplemental_Files

## Data Availability

The data underlying this article are available in the ProteomeXchange consortium and can be accessed with the unique identifier PXD059506. The protein sequence data reported in this paper will appear in the UniProt Knowledgebase under the accession numbers C0HMF0 (H4 & H4.S), C0HMF1 (H2A & H2A.A), C0HMF2 (H2B.1), C0HMF3 (H2B.2 & H2B.2K), and C0HMF4 (H2B.3).
