# Supplementary material for: Direct histone proteoform profiling of the unannotated, endangered coral Acropora cervicornis
Source: Nucleic Acids Res. 2025 Jul 31;53(14):gkaf740. doi: 10.1093/nar/gkaf740 (PMC12311784; doi:10.1093/nar/gkaf740)
Supplement: gkaf740_Supplemental_Files [file gkaf740_supplemental_files.zip › Supplemental_Information_revised.pdf]

Supplementary Information

Direct histone proteoform profiling of the unannotated, endangered coral *Acropora cervicornis*

Cassandra N. Fuller[1], Sabrina Mansoor[2], Kevin Jeanne Dit Fouque[1], Lilian Valadares Tose[1], Javier Rodriguez-Casariego[2,3], Mariangela Kosmopoulou[4], Detlev Suckau[5], Francisca N. L. Vitorino[6], Benjamin A. Garcia[6], Jose M. Eirin-Lopez[2], Francisco Fernandez-Lima[1,7]*

[1]Department of Chemistry and Biochemistry, Florida International University, Miami, FL 33199, USA.

[2]Environmental Epigenetics Laboratory, Institute of Environment, Florida International University, Miami, FL 33199, USA.

[3]Department of Marine Biology and Ecology, Rosenstiel School, University of Miami, Miami, FL 33124, USA.

[4]Fasmatech Science & Technology, TESPA Lefkippos, NCSR Demokritos, Agia Paraskevi, 15341 Athens, Greece.

[5]Bruker Daltonics GmbH & Co. KG, Bremen, Germany.

[6]Department of Biochemistry and Molecular Biophysics, Washington University School of Medicine, St. Louis, Missouri 63110, United States

[7]Biomolecular Sciences Institute, Florida International University, Miami, FL 33199, USA.

Contents

Figure S1. EpiQuik™ Total Histone Extraction Kit Protocol step 1-3 used............................ 8

Figure S2. Custom-built LC-TIMS-ECD-ToF MS/MS instrument schematic. 9

Figure S3. Top-down and bottom-up MS instrument parameters....................................11

Figure S4. Propionylated digest procedure. 12

Figure S5. Top-down proteoform confirmation of *A. cervicornis* H4 variant PTM patterns with ±10 ppm error: Unmodified and K20me$_1$...13

Figure S6. Top-down proteoform confirmation of *A. cervicornis* H4 variant PTM patterns with ±10 ppm error: K20me$_2$ and N-acS...14

Figure S7. Top-down proteoform confirmation of *A. cervicornis* H4 variant PTM patterns with ±10 ppm error: N-acSK20me$_1$ and N-acSK20me$_2$. 15

Figure S8. Top-down proteoform confirmation of *A. cervicornis* H4 variant PTM patterns with ±10 ppm error: N-acSK12ac and N-acSK16ac. 16

Figure S9. Top-down proteoform confirmation of *A. cervicornis* H4 variant PTM patterns with ±10 ppm error: N-acSK12acK20me$_1$ and N-acSK16acK20me$_1$...................................17

Figure S10. Top-down proteoform confirmation of *A. cervicornis* H4 variant PTM patterns with ±10 ppm error: N-acSK12acK20me$_2$ and N-acSK16acK20me$_2$.................................18

Figure S11. Top-down proteoform confirmation of *A. cervicornis* H4 variant PTM patterns with ±10 ppm error: N-acSK5acK16ac and N-acSK8acK16ac. 19

Figure S12. Top-down proteoform confirmation of *A. cervicornis* H4 variant PTM patterns with ±10 ppm error: N-acSK12acK16ac and N-acSK5acK16acK20me$_1$. 20

Figure S13. Top-down proteoform confirmation of *A. cervicornis* H4 variant PTM patterns with ±10 ppm error: N-acSK8acK16acK20me$_1$ and N-acSK12acK16acK20me$_1$..................21

Figure S14. Top-down proteoform confirmation of *A. cervicornis* H4 variant PTM patterns with ±10 ppm error: N-acSK5acK16acK20me$_2$ and N-acSK8acK16acK20me$_2$. 22

Figure S15. Top-down proteoform confirmation of *A. cervicornis* H4 variant PTM patterns with ±10 ppm error: N-acSK12acK16acK20me$_1$ and N-acSK5acK12acK16ac. 23

Figure S16. Top-down proteoform confirmation of *A. cervicornis* H4 variant PTM patterns with ±10 ppm error: N-acSK8acK12acK16ac and N-acSK5acK12acK16acK20me$_1$. 24

Figure S17. Top-down proteoform confirmation of *A. cervicornis* H4 variant PTM patterns with ±10 ppm error: N-acSK8acK12acK16acK20me$_1$ and N-acSK5acK12acK16acK20me$_2$.25

Figure S18. Top-down proteoform confirmation of *A. cervicornis* H4 variant PTM patterns with ±10 ppm error: N-acSK8acK12acK16acK20me$_2$ and N-acSK5acK8acK12acK16ac. ...26

Figure S19. Top-down proteoform confirmation of *A. cervicornis* H4.S variant PTM patterns with ±10 ppm error: Unmodified and K20me$_1$. ...27

Figure S20. Top-down proteoform confirmation of *A. cervicornis* H4.S variant PTM patterns with ±10 ppm error: K20me$_2$ and N-acS. ...28

Figure S21. Top-down proteoform confirmation of *A. cervicornis* H4.S variant PTM patterns with ±10 ppm error: N-acSK20me$_1$ and N-acSK20me$_2$. ...29

Figure S22. Top-down proteoform confirmation of *A. cervicornis* H4.S variant PTM patterns with ±10 ppm error: N-acSK12ac and N-acSK16ac. ...30

Figure S23. Top-down proteoform confirmation of *A. cervicornis* H4.S variant PTM patterns with ±10 ppm error: N-acSK12acK20me$_1$ and N-acSK16acK20me$_1$. ...31

Figure S24. Top-down proteoform confirmation of *A. cervicornis* H4.S variant PTM patterns with ±10 ppm error: N-acSK12acK20me$_2$ and N-acSK16acK20me$_2$. ...32

Figure S25. Top-down proteoform confirmation of *A. cervicornis* H4.S variant PTM patterns with ±10 ppm error: N-acSK5acK16ac and N-acSK8acK16ac. ...33

Figure S26. Top-down proteoform confirmation of *A. cervicornis* H4.S variant PTM patterns with ±10 ppm error: N-acSK12acK16ac and N-acSK5acK16acK20me$_1$. ...34

Figure S27 .Top-down proteoform confirmation of *A. cervicornis* H4.S variant PTM patterns with ±10 ppm error: N-acSK8acK16acK20me$_1$ and N-acSK12acK16acK20me$_1$. ...35

Figure S28. Top-down proteoform confirmation of *A. cervicornis* H4.S variant PTM patterns with ±10 ppm error: N-acSK5acK16acK20me$_2$ and N-acSK8acK16acK20me$_2$. ...36

Figure S29. Top-down proteoform confirmation of *A. cervicornis* H4.S variant PTM patterns with ±10 ppm error: N-acSK12acK16acK20me$_2$ and N-acSK5acK12acK16ac. ...37

Figure S30. Top-down proteoform confirmation of *A. cervicornis* H4.S variant PTM patterns with ±10 ppm error: N-acSK8acK12acK16ac and N-acSK5acK12acK16acK20me$_1$. ...38

Figure S31. Top-down proteoform confirmation of *A. cervicornis* H4.S variant PTM patterns with ±10 ppm error: N-acSK8acK12acK16acK20me$_1$. ...39

Figure S32. Top-down proteoform confirmation of *A. cervicornis* H2A variant PTM patterns with ±10 ppm error: Unmodified and N-acS. ...40

Figure S33. Top-down proteoform confirmation of *A. cervicornis* H2A variant PTM patterns with ±10 ppm error: K5ac and K7ac. ...41

Figure S34. Top-down proteoform confirmation of *A. cervicornis* H2A variant PTM patterns with ±10 ppm error: K74ac and N-acSK5ac. 42

Figure S35. Top-down proteoform confirmation of *A. cervicornis* H2A variant PTM patterns with ±10 ppm error: N-acSK7ac and N-acSK74ac..43

Figure S36. Top-down proteoform confirmation of *A. cervicornis* H2A variant PTM patterns with ±10 ppm error: K5acK7ac. 44

Figure S37. Top-down proteoform confirmation of *A. cervicornis* H2A.A variant PTM patterns with ±10 ppm error: Unmodified and N-acS...45

Figure S38. Top-down proteoform confirmation of *A. cervicornis* H2A.A variant PTM patterns with ±10 ppm error: K5ac and K7ac..46

Figure S39. Top-down proteoform confirmation of *A. cervicornis* H2A.A variant PTM patterns with ±10 ppm error: K74ac and N-acSK5ac. 47

Figure S40. Top-down proteoform confirmation of *A. cervicornis* H2A.A variant PTM patterns with ±10 ppm error: N-acSK7ac and N-acSK74ac. 48

Figure S41. Top-down proteoform confirmation of *A. cervicornis* H2A.A variant PTM patterns with ±10 ppm error: K5acK7ac. 49

Figure S42. Top-down proteoform confirmation of *A. cervicornis* H2B-1 variant PTM patterns with ±10 ppm error: N-me$_2$A and N-me$_3$A. 50

Figure S43. Top-down proteoform confirmation of *A. cervicornis* H2B-1 variant PTM patterns with ±10 ppm error: N-me$_3$AM56ox and N-me$_3$AK11ac. 51

Figure S44. Top-down proteoform confirmation of *A. cervicornis* H2B-2 variant PTM patterns with ±10 ppm error: N-me$_2$A and N-me$_3$A. 52

Figure S45. Top-down proteoform confirmation of *A. cervicornis* H2B-2 variant PTM patterns with ±10 ppm error: N-me$_3$AM56ox and N-me$_3$AK11ac. 53

Figure S46. Top-down proteoform confirmation of *A. cervicornis* H2B-2K variant PTM patterns with ±10 ppm error: N-me$_2$A and N-me$_3$A. 54

Figure S47. Top-down proteoform confirmation of *A. cervicornis* H2B-2K variant PTM patterns with ±10 ppm error: N-me$_3$AM56ox and N-me$_3$AK11ac. 55

Figure S48. Top-down proteoform confirmation of *A. cervicornis* H2B-3 variant PTM patterns with ±10 ppm error: N-me$_2$A and N-me$_3$A. 56

Figure S49. Top-down proteoform confirmation of *A. cervicornis* H2B-3 variant PTM patterns with ±10 ppm error: N-me$_3$AK11ac. 57

Figure S50. Ion annotation of MS/MS spectrum for H4 N-acSK20me2 $[M+12H]^{12+}$. 58

Figure S51. Ion annotation of MS/MS spectrum for H4.S N-acSK20me2 $[M+13H]^{13+}$. 64
Figure S52. Ion annotation of MS/MS spectrum for H2A N-acS $[M+14H]^{14+}$.........................69
Figure S53. Ion annotation of MS/MS spectrum for H2B-1 N-me3A $[M+16H]^{16+}$..................74
Figure S54. Ion annotation of MS/MS spectrum for H2B-2 N-me3A $[M+15H]^{15+}$. 78
Figure S55. Ion annotation of MS/MS spectrum for H2B-3 N-me3A $[M+14H]^{14+}$. 82
Figure S56. Bottom-up DataAnalysis script and example script input..............................87
Figure S57. Replicate 1 bottom-up fragmentation of *A. cervicornis* H4 4-17 and 20-23 PTM patterns with ±10 ppm error: 4-17 unmodified and 4-17 K12ac..89
Figure S58. Replicate 1 bottom-up fragmentation of *A. cervicornis* H4 4-17 and 20-23 PTM patterns with ±10 ppm error: 4-17 K16ac and 4-17 K5acK16ac..90
Figure S59. Replicate 1 bottom-up fragmentation of *A. cervicornis* H4 4-17 and 20-23 PTM patterns with ±10 ppm error: 4-17 K8acK16ac and 4-17 K12acK16ac. 91
Figure S60. Replicate 1 bottom-up fragmentation of *A. cervicornis* H4 4-17 and 20-23 PTM patterns with ±10 ppm error: 4-17 K5acK12acK16ac and 4-17 K8acK12acK16ac. 92
Figure S61. Replicate 1 bottom-up fragmentation of *A. cervicornis* H4 4-17 and 20-23 PTM patterns with ±10 ppm error: 4-17 K5acK8acK12acK16ac and 20-23 unmodified.............93
Figure S62. Replicate 1 bottom-up fragmentation of *A. cervicornis* H4 4-17 and 20-23 PTM patterns with ±10 ppm error: 20-23 K20me$_1$ and 20-23 K20me$_2$.......................................94
Figure S63. Replicate 1 bottom-up fragmentation of *A. cervicornis* H4 4-17 and 20-23 PTM patterns with ±10 ppm error: 20-23 K20ac. 95
Figure S64. Replicate 2 bottom-up fragmentation of *A. cervicornis* H4 4-17 and 20-23 PTM patterns with ±10 ppm error: 4-17 unmodified and 4-17 K12ac..96
Figure S65. Replicate 2 bottom-up fragmentation of *A. cervicornis* H4 4-17 and 20-23 PTM patterns with ±10 ppm error: 4-17 K16ac and 4-17 K5acK16ac..97
Figure S66. Replicate 2 bottom-up fragmentation of *A. cervicornis* H4 4-17 and 20-23 PTM patterns with ±10 ppm error: 4-17 K8acK16ac and 4-17 K12acK16ac. 98
Figure S67. Replicate 2 bottom-up fragmentation of *A. cervicornis* H4 4-17 and 20-23 PTM patterns with ±10 ppm error: 4-17 K5acK12acK16ac and 4-17 K8acK12acK16ac. 99
Figure S68. Replicate 2 bottom-up fragmentation of *A. cervicornis* H4 4-17 and 20-23 PTM patterns with ±10 ppm error: 4-17 K5acK8acK12acK16ac and 20-23 unmodified...........100
Figure S69. Replicate 2 bottom-up fragmentation of *A. cervicornis* H4 4-17 and 20-23 PTM patterns with ±10 ppm error: 20-23 K20me$_1$ and 20-23 K20me$_2$.....................................101

Figure S70. Replicate 2 bottom-up fragmentation of *A. cervicornis* H4 4-17 and 20-23 PTM patterns with ±10 ppm error: 20-23 K20ac. 102
Figure S71. Replicate 3 bottom-up fragmentation of *A. cervicornis* H4 4-17 and 20-23 PTM patterns with ±10 ppm error: 4-17 unmodified and 4-17 K12ac....................103
Figure S72. Replicate 3 bottom-up fragmentation of *A. cervicornis* H4 4-17 and 20-23 PTM patterns with ±10 ppm error: 4-17 K16ac and 4-17 K5acK16ac. 104
Figure S73. Replicate 3 bottom-up fragmentation of *A. cervicornis* H4 4-17 and 20-23 PTM patterns with ±10 ppm error: 4-17 K8acK16ac and 4-17 K12acK16ac. 105
Figure S74. Replicate 3 bottom-up fragmentation of *A. cervicornis* H4 4-17 and 20-23 PTM patterns with ±10 ppm error: 4-17 K5acK12acK16ac and 4-17 K8acK12acK16ac. 106
Figure S75. Replicate 3 bottom-up fragmentation of *A. cervicornis* H4 4-17 and 20-23 PTM patterns with ±10 ppm error: 4-17 K5acK8acK12acK16ac and 20-23 unmodified. 107
Figure S76. Replicate 3 bottom-up fragmentation of *A. cervicornis* H4 4-17 and 20-23 PTM patterns with ±10 ppm error: 20-23 K20me$_1$ and 20-23 K20me$_2$. 108
Figure S77. Replicate 3 bottom-up fragmentation of *A. cervicornis* H4 4-17 and 20-23 PTM patterns with ±10 ppm error: 20-23 K20ac. 109
Figure S78. A. cervicornis histone extraction and online LC-MS. 110
Figure S79. Observed mass shifts in H4 and H2A/H2B A. cervicornis histone fractions. ..111
Figure S80. MS-BLAST results for A. cervicornis 32.0 and 36.9 min (H4 & H4.S) fractions.112
Figure S81. Highest intensity charge state ECD-MS/MS and highest intensity proteoforms sequenced for H2A/H2B fractions. 113
Figure S82. MS-BLAST results for A. cervicornis 30.1 min (H2B-1) fraction. 114
Figure S83. MS-BLAST results for A. cervicornis 33.8 min (H2B-2/H2B-2K) fraction. 115
Figure S84. MS-BLAST results for A. cervicornis 35.2 min (H2B-3) fraction. 116
Figure S85. MS-BLAST results for A. cervicornis 45.9 min (H2A/H2A.A) fraction. 117
Figure S86. A. cervicornis H4 variant proposed sequences compared to highest similarity sequences from UniProt. 118
Figure S87. Bottom-up H4 target peptides with relative abundance of observed peptides. 119
Figure S88. Bottom-up H2A target peptides with relative abundance of observed peptides. 120

Figure S89. Bottom-up H2B target peptides with relative abundance of observed peptides. 121
Figure S90. Comparison of S/N from IMS-MS and LC-MS. 122

PROTOCOL

1. For Tissues (Treated and Untreated)
    a. Add the Diluted 1X Pre-Lysis Buffer at 1 ml per 200 mg of tissue and disaggregate tissue pieces by 50-60 strokes.
    b. Transfer homogenized mixture to a 2 ml vial and centrifuge at 10,000 rpm for 1 min at 4°C.
    c. Remove supernatant.

Note: After treatment with Pre-Lysis Buffer, the supernatant will contain the cytoplasmic fraction, while the intact nuclei will pellet.

2. Re-suspend cell/tissue pellet in 3 volumes (approximately 200 µl/107 cells or 100 mg of tissue) of Lysis Buffer and incubate on ice for 30 min.
3. Centrifuge at 12,000 rpm for 5 min at 4°C and transfer the supernatant fraction (containing acid-soluble proteins) into a new vial.

Figure S1. EpiQuik™ Total Histone Extraction Kit Protocol step 1-3 used.

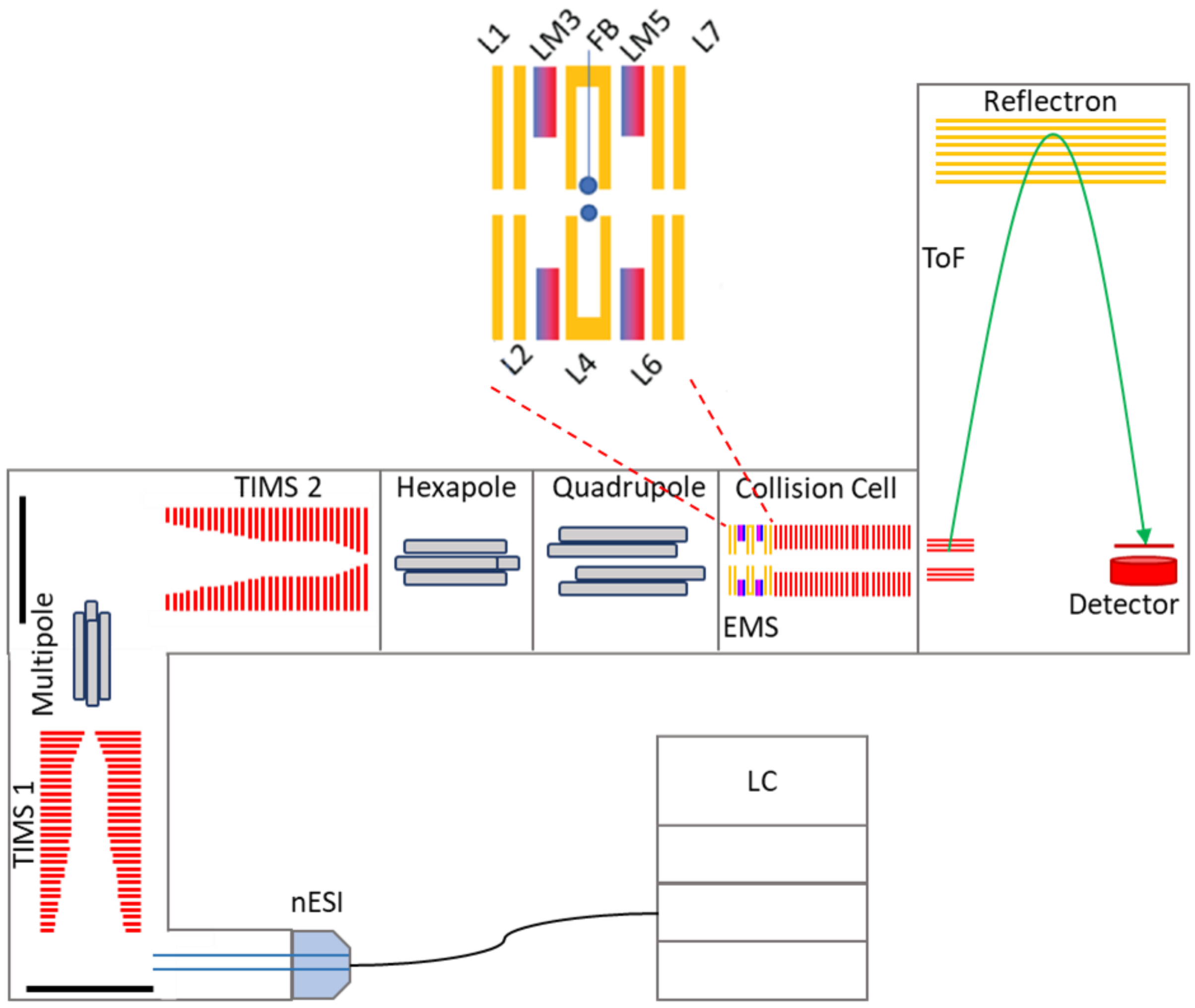

Figure S2. Custom-built LC-TIMS-ECD-ToF MS/MS instrument schematic.

Top-Down MS parameters

PARAMETERS	SETTINGS IN TRANSMISSION MODE*	SETTINGS IN ECD MODE
L1	34.5 V	29.5 V
L2	-43.8 V	38.6 V
LM3	33.2 V	46.5 V
L4	32.0 V	48.3 V
FB	31.1 V	35.5 V
LM5	30.0 V	38.1 V
L6	29.1 V	37.9 V
L7	28.3 V	31.0 V
FB CURRENT	0 A	2.5 A
COLLISION ENERGY	10 eV	6 eV

*Note: TIMS1 was operated in transmission mode

Bottom-up PASEF MS parameters

ACQUISITION PARAMETER	VALUE
PASEF CHARGE RANGE MINIMUM	0
PASEF CURRENT INTENS/PREVIOUS INTENS	4.00
PASEF DUPLICATE DETECTION MOBILITY TOLERANCE	10 pts
PASEF SCHEDULING MEASURING TIME	2.75 ms
PASEF INTENSITY THRESHOLD FOR SCHEDULING	1000
PASEF USE INTENSITY / REPETITION TABLE	On
PASEF EXCLUSION WINDOW V·S/CM² WIDTH	0.015 V·s/cm²
PASEF MAXIMUM NUMBER OF MOBILOGRAM PEAKS	3
PASEF MASSES	200.00 m/z
PASEF INTENSITY REPETITION TABLE REPETITION ROW	10; 10; 9; 8; 7; 6; 5; 4; 3; 2; 1
PASEF TRIGGERS	0
PASEF ACTIVE	1
PASEF CHARGE RANGE MAXIMUM	3

PASEF CYCLE OVERLAP	4
PASEF ACTIVE EXCLUSION SWITCH	On
PASEF RECONSIDER PRECURSOR SWITCH	On
PASEF EXCLUSION RELEASE TIME	0.40 min
PASEF EXCLUSION WINDOW MASS WIDTH	0.015 m/z
PASEF INTENSITY REPETITION TABLE INTENSITY ROW	0; 6324; 6666; 7071; 7559; 8164; 8944; 10000; 11547; 14142; 20000
PASEF MOBILOGRAM GRID MASS OVERLAP	0.50 Da
PASEF MOBILOGRAM GRID MASS WIDTH	2.50 Da
PASEF MOBILOGRAM SUMMATION WIDTH	25 pts.
PASEF MOBILOGRAM THRESHOLD	50
PASEF MS REPETITIONS	1 x
PASEF DUPLICATE DETECTION MASS TOLERANCE	0.025 m/z
PASEF NUMBER OF MS/MS SCANS	10
PASEF NUMBER OF VALID PRECURSORS	1
PASEF RESOLUTIONS	0.00 m/z
PASEF SCHEDULING SWITCHING TIME	1.65 ms
PASEF SCHEDULING TARGET INTENSITY	10000
PASEF MOBILOGRAM TOF RESOLUTION	45000
PASEF TOTAL CYCLE TIME	1.17 s

Figure S3. Top-down and bottom-up MS instrument parameters.

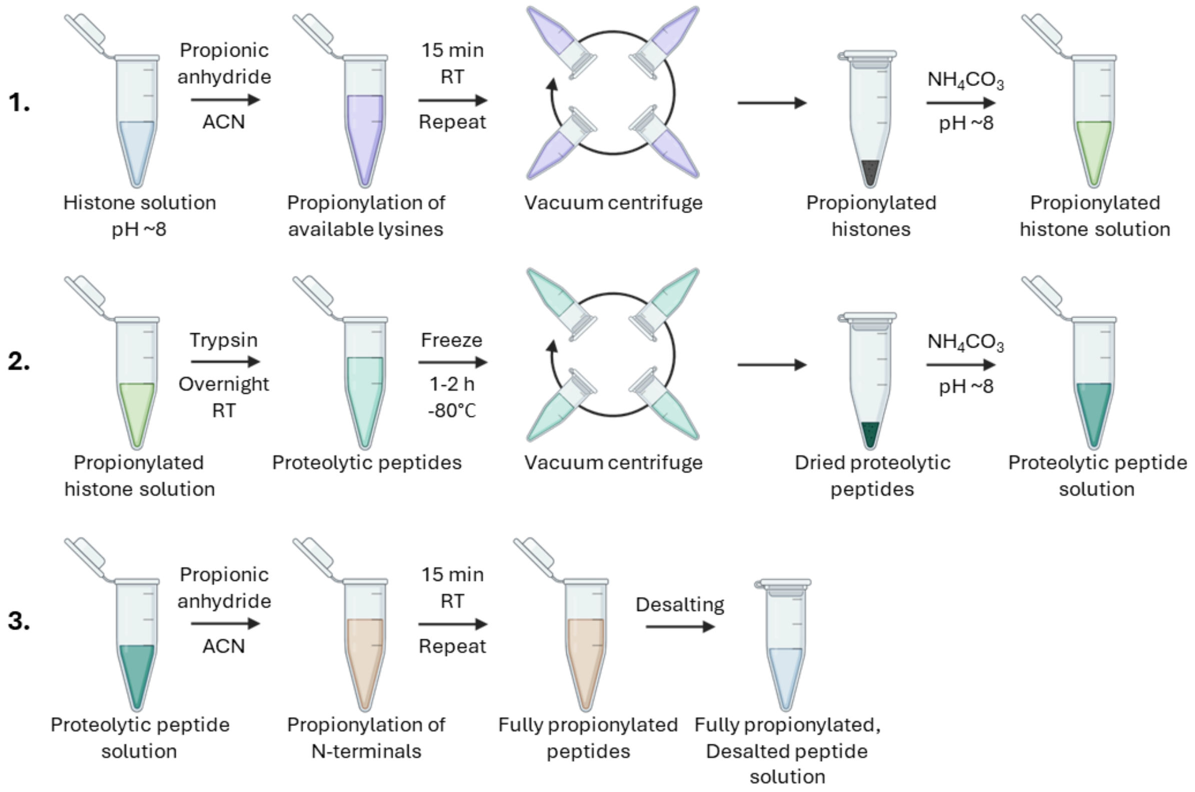

Figure S4. Propionylated digest procedure.

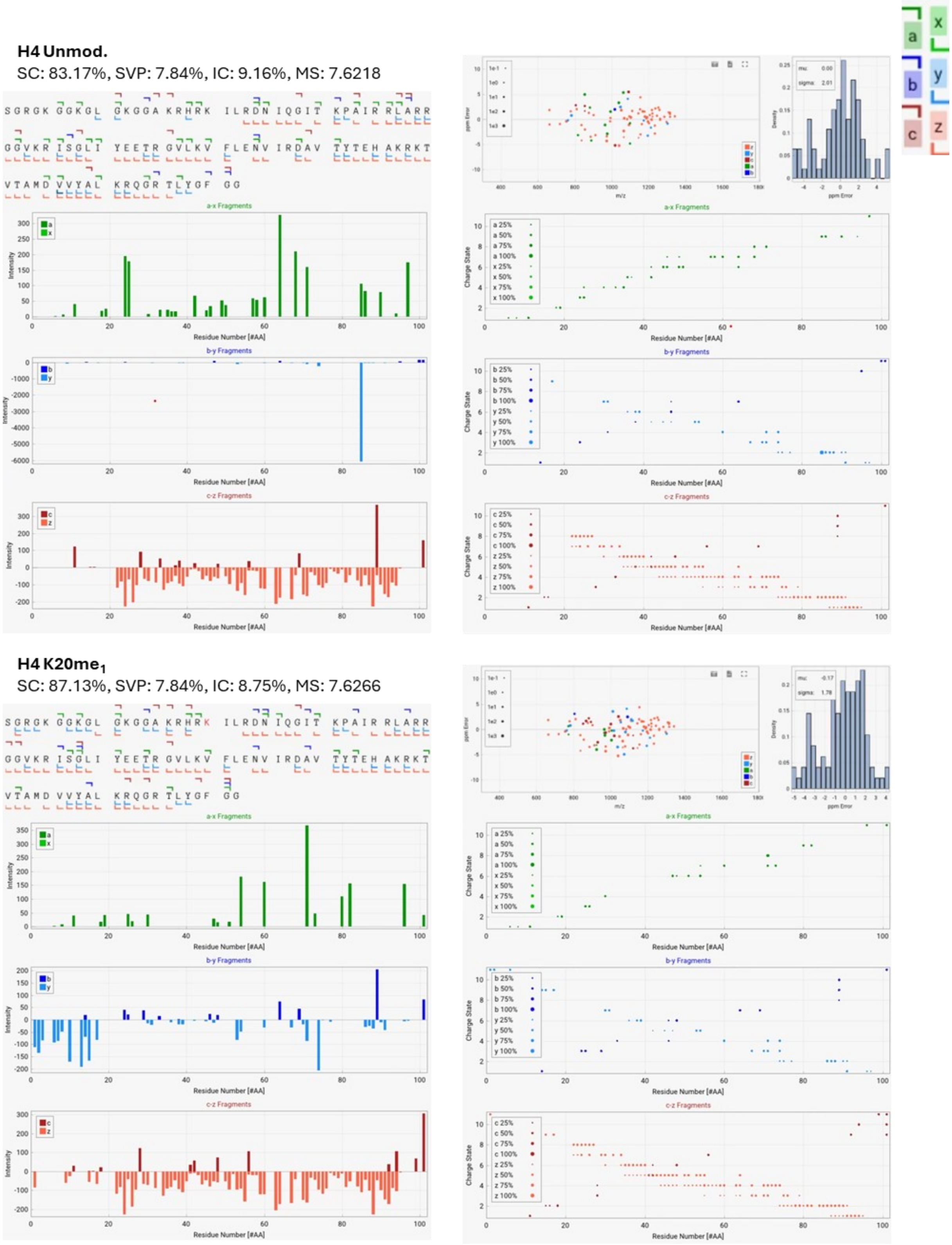

Figure S5. Top-down proteoform confirmation of *A. cervicornis* H4 variant PTM patterns with ±10 ppm error: Unmodified and K20me$_1$.

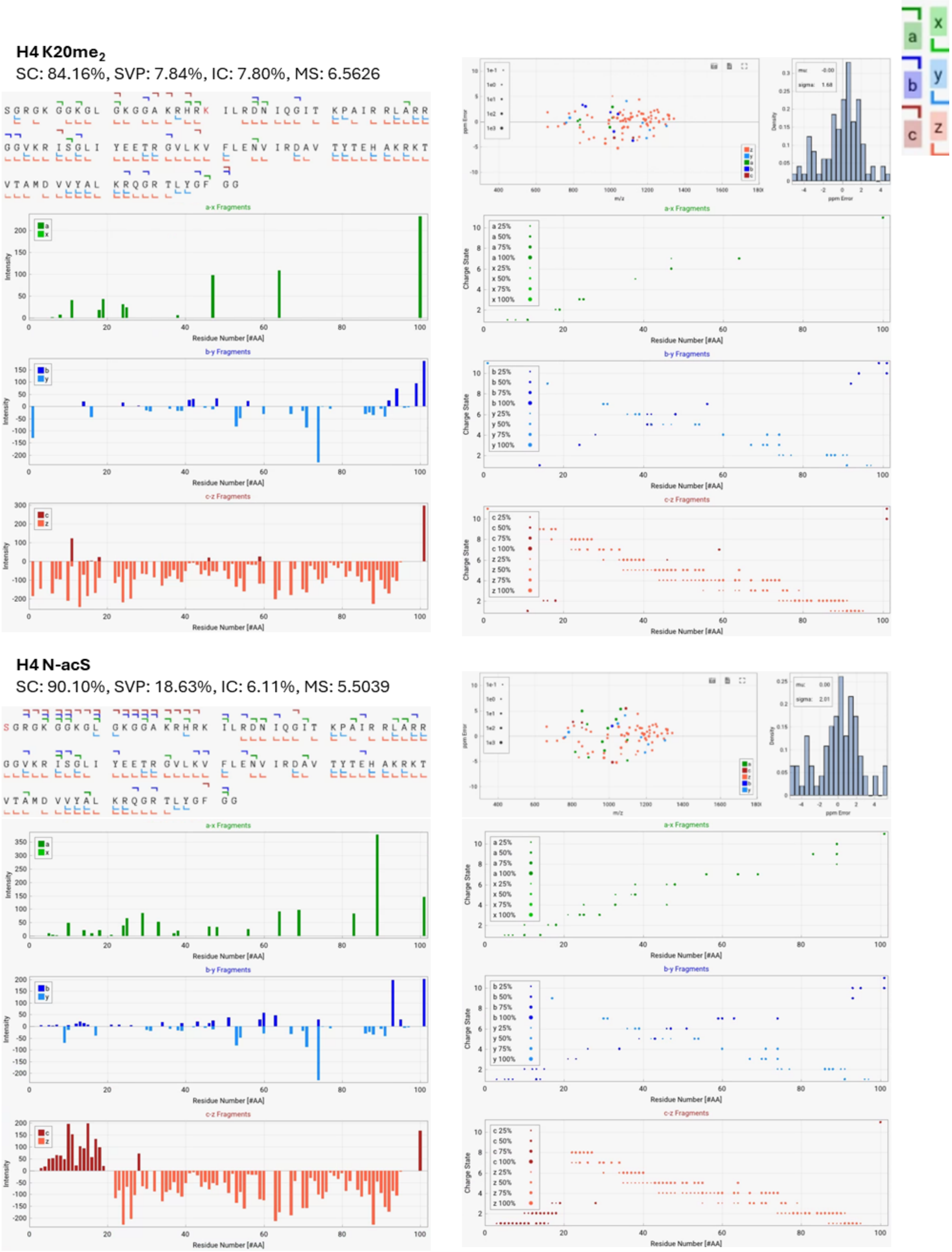

Figure S6. Top-down proteoform confirmation of *A. cervicornis* H4 variant PTM patterns with ±10 ppm error: K20me$_2$ and N-acS.

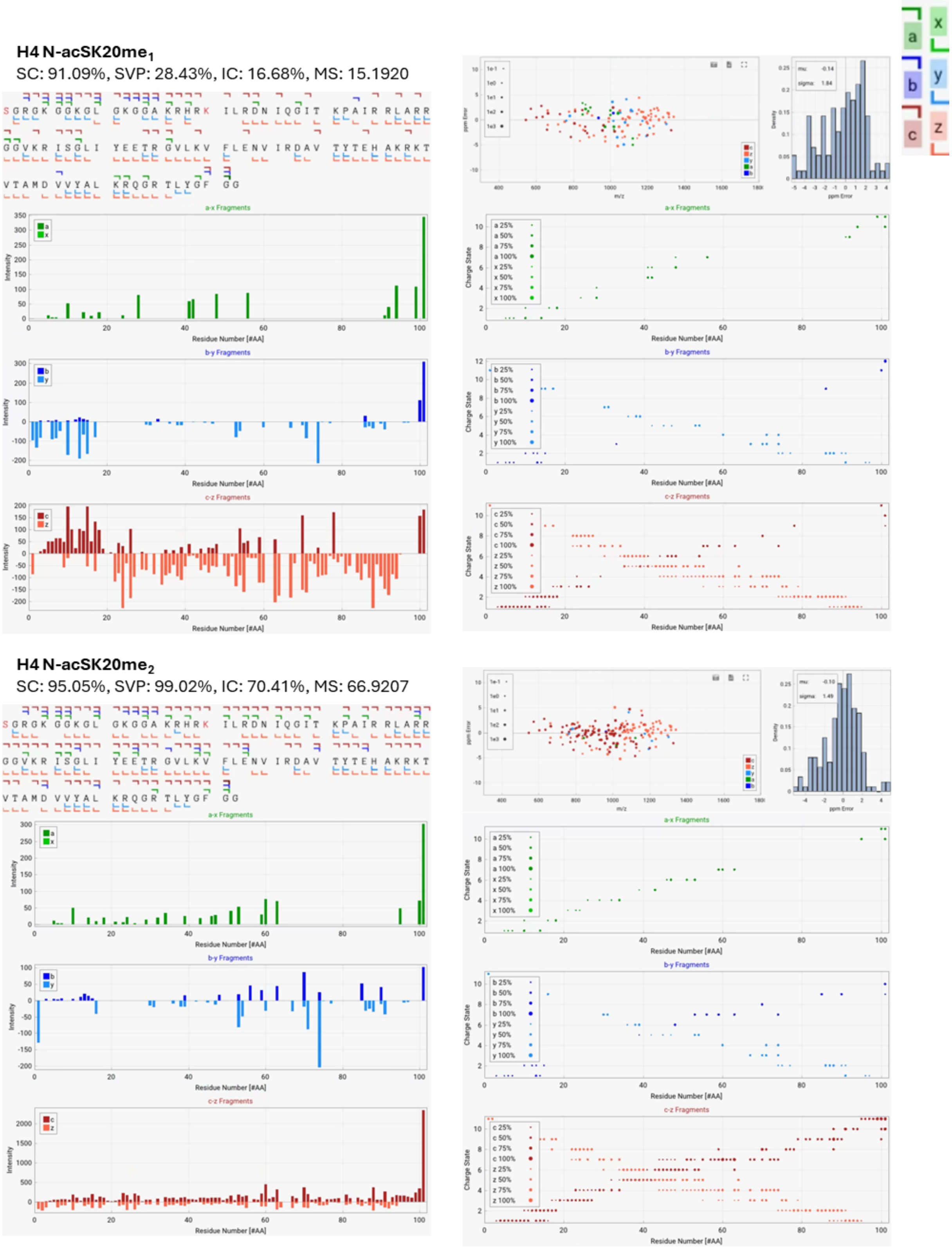

Figure S7. Top-down proteoform confirmation of *A. cervicornis* H4 variant PTM patterns with ±10 ppm error: N-acSK20me$_1$ and N-acSK20me$_2$.

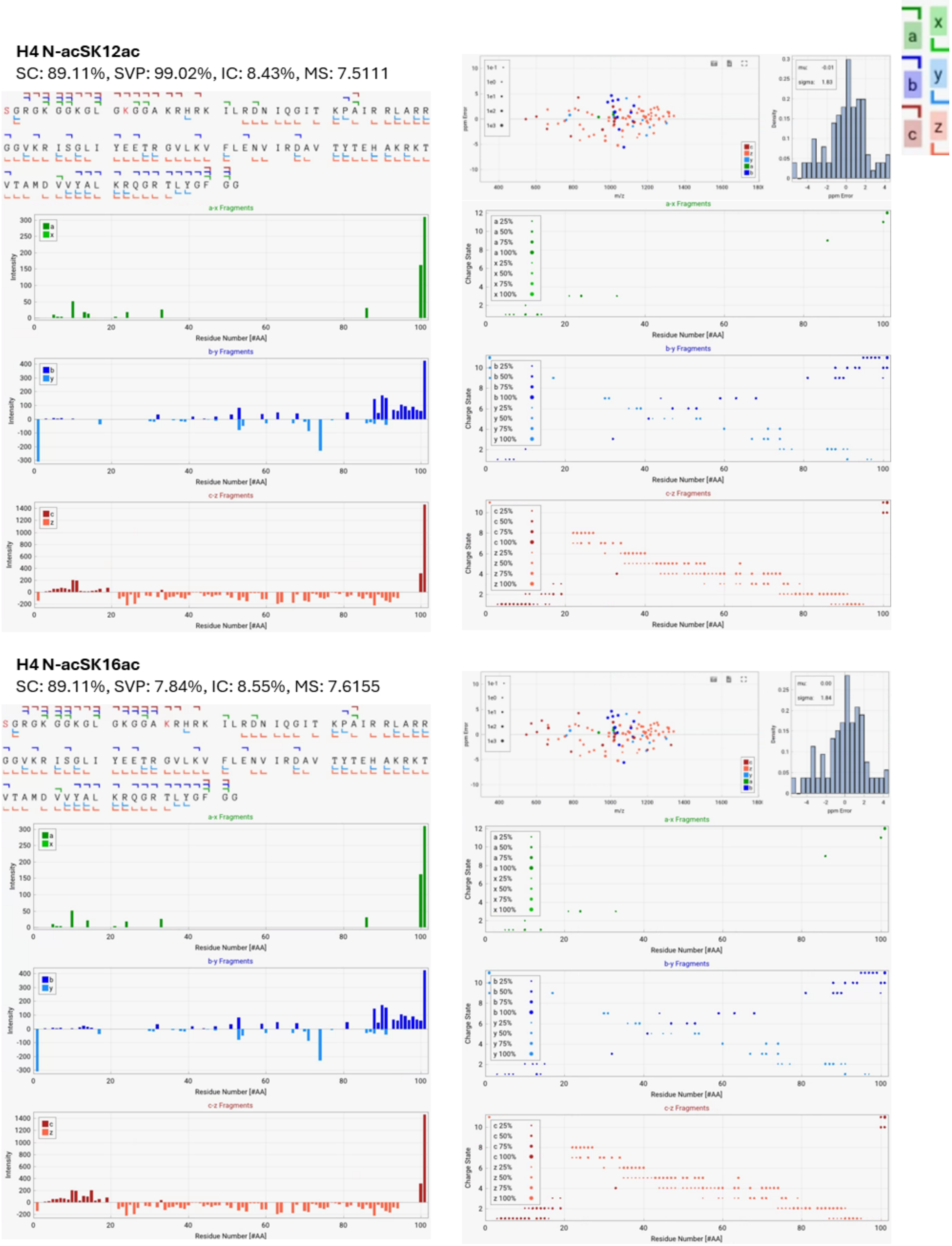

Figure S8. Top-down proteoform confirmation of *A. cervicornis* H4 variant PTM patterns with ±10 ppm error: N-acSK12ac and N-acSK16ac.

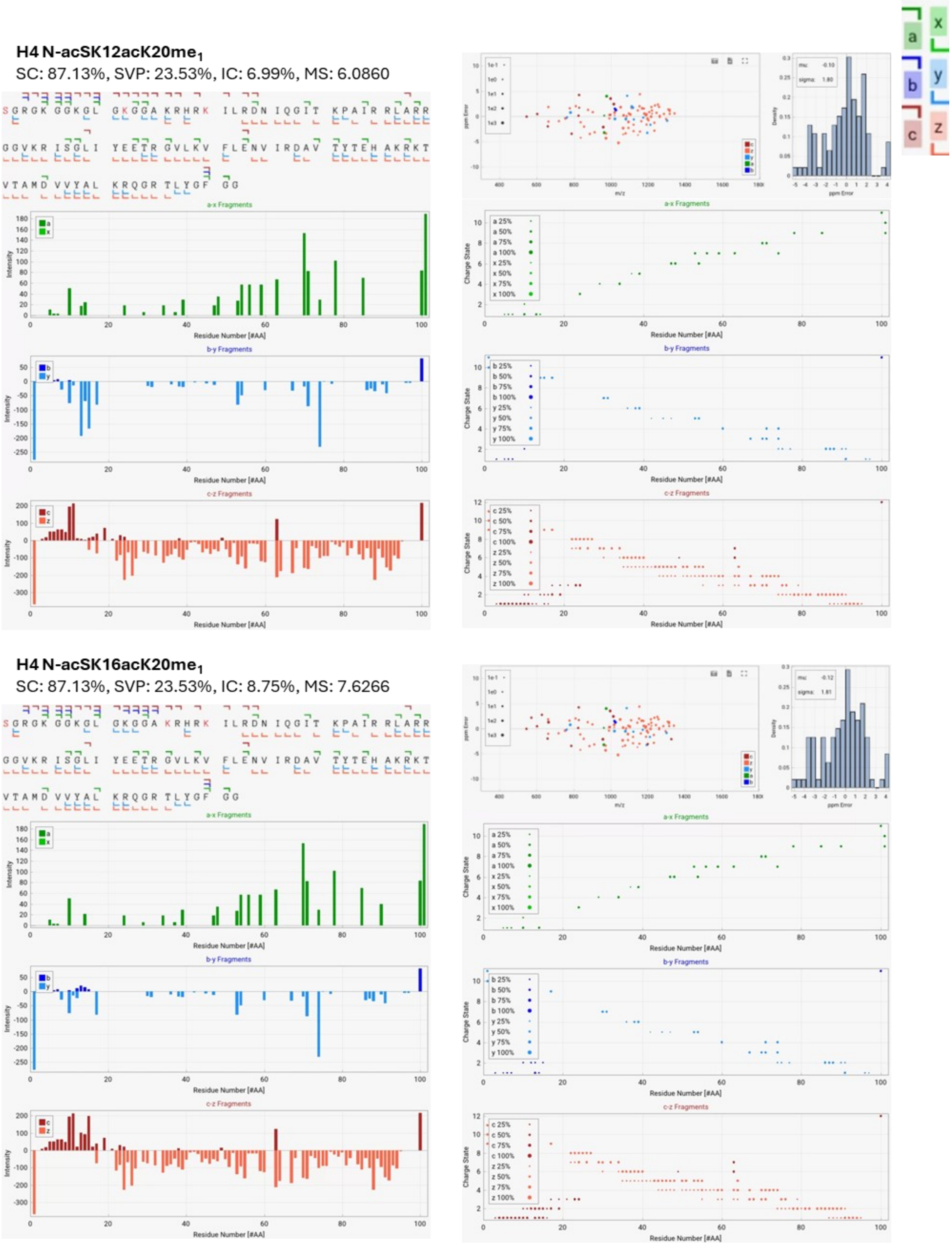

Figure S9. Top-down proteoform confirmation of *A. cervicornis* H4 variant PTM patterns with ±10 ppm error: N-acSK12acK20me$_1$ and N-acSK16acK20me$_1$.

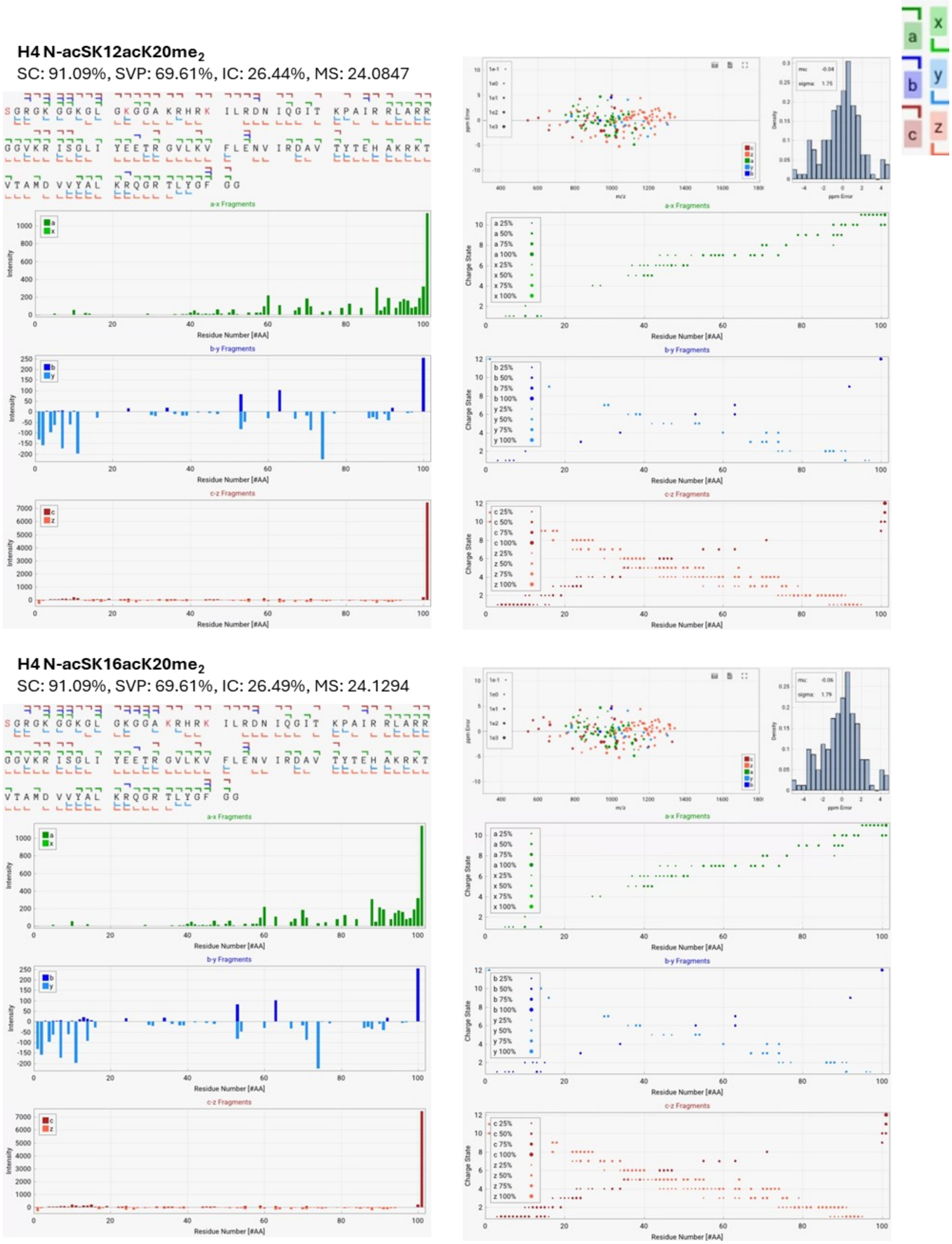

Figure S10. Top-down proteoform confirmation of *A. cervicornis* H4 variant PTM patterns with ±10 ppm error: N-acSK12acK20me$_2$ and N-acSK16acK20me$_2$.

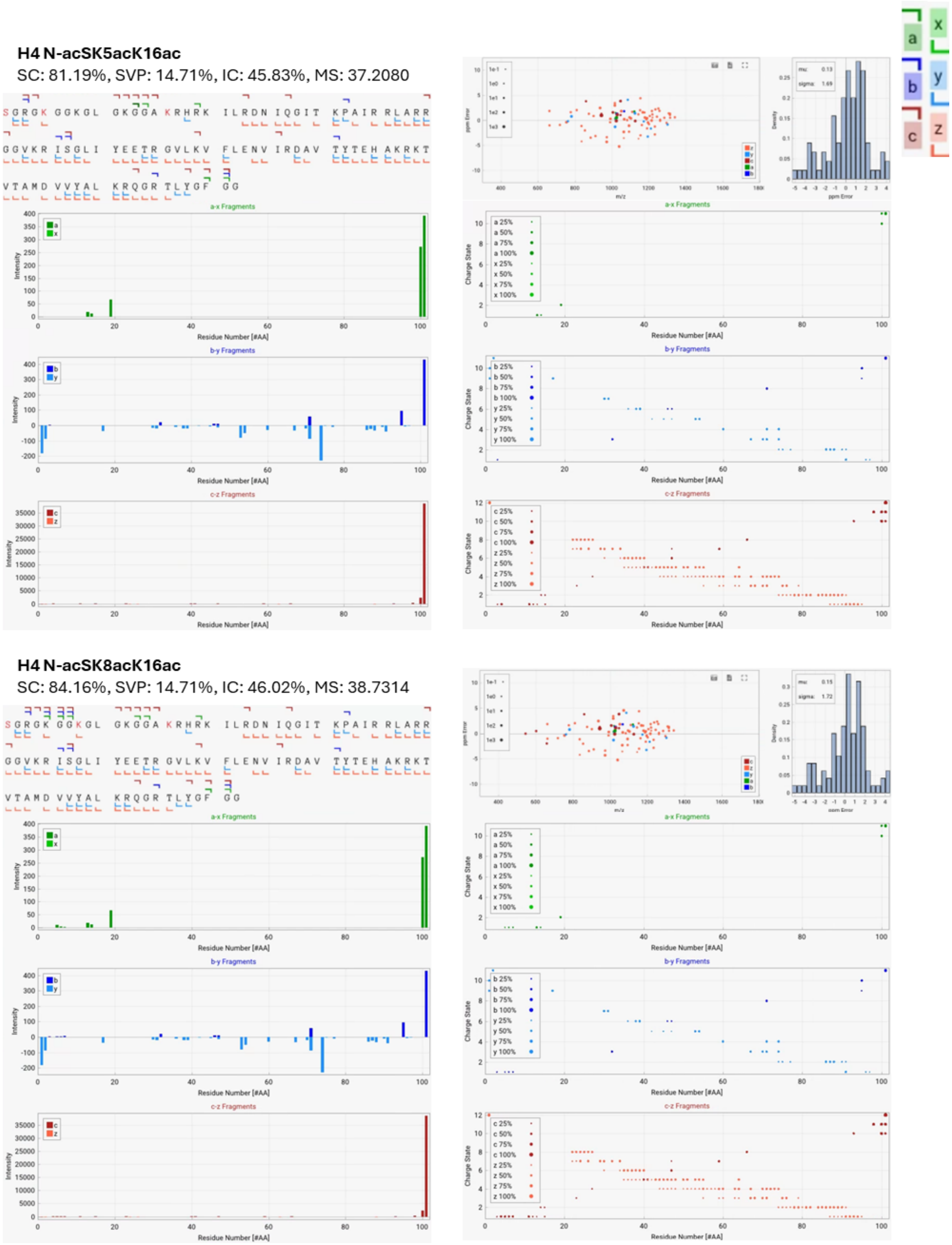

Figure S11. Top-down proteoform confirmation of *A. cervicornis* H4 variant PTM patterns with ±10 ppm error: N-acSK5acK16ac and N-acSK8acK16ac.

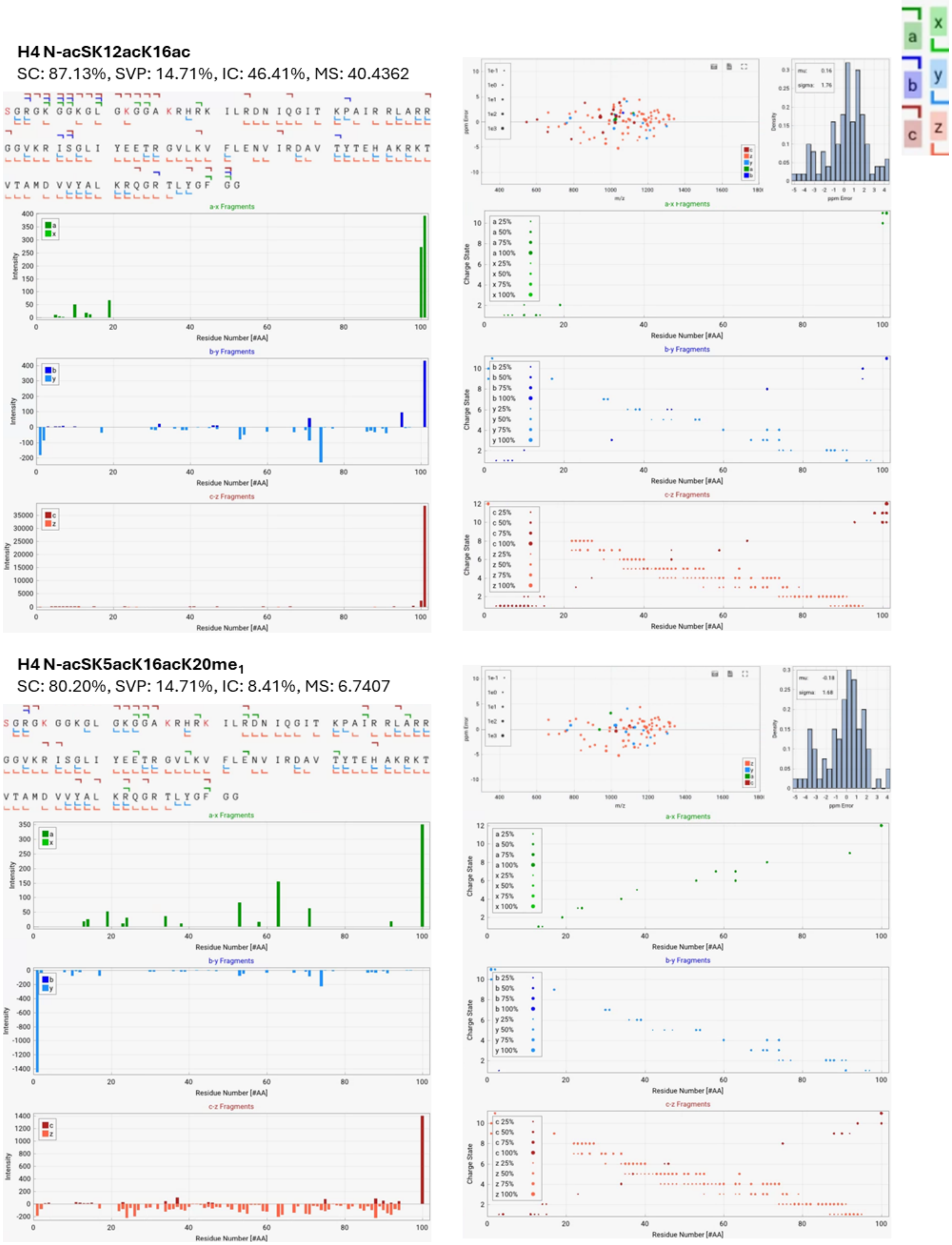

Figure S12. Top-down proteoform confirmation of *A. cervicornis* H4 variant PTM patterns with ±10 ppm error: N-acSK12acK16ac and N-acSK5acK16acK20me$_1$.

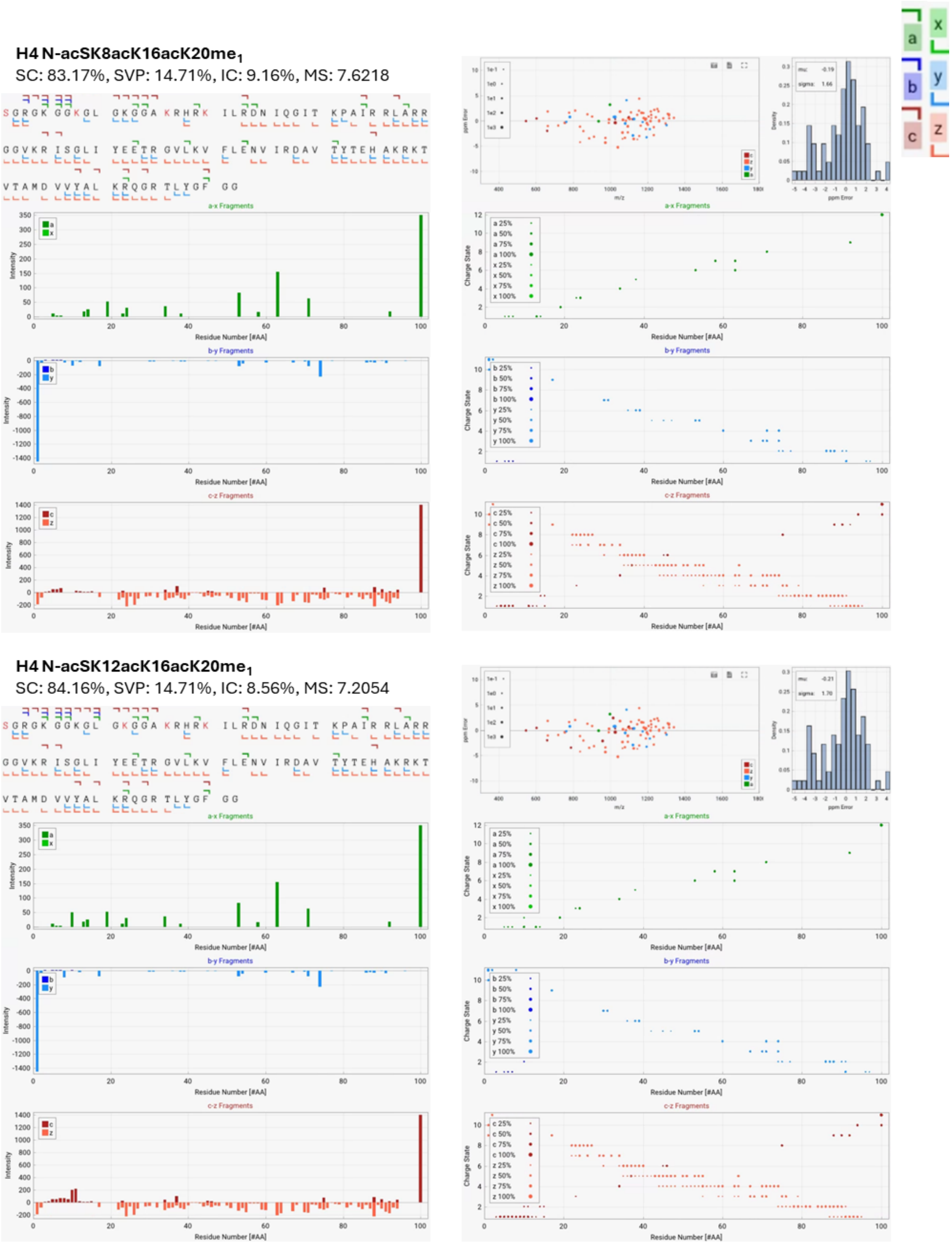

Figure S13. Top-down proteoform confirmation of *A. cervicornis* H4 variant PTM patterns with ±10 ppm error: N-acSK8acK16acK20me$_1$ and N-acSK12acK16acK20me$_1$.

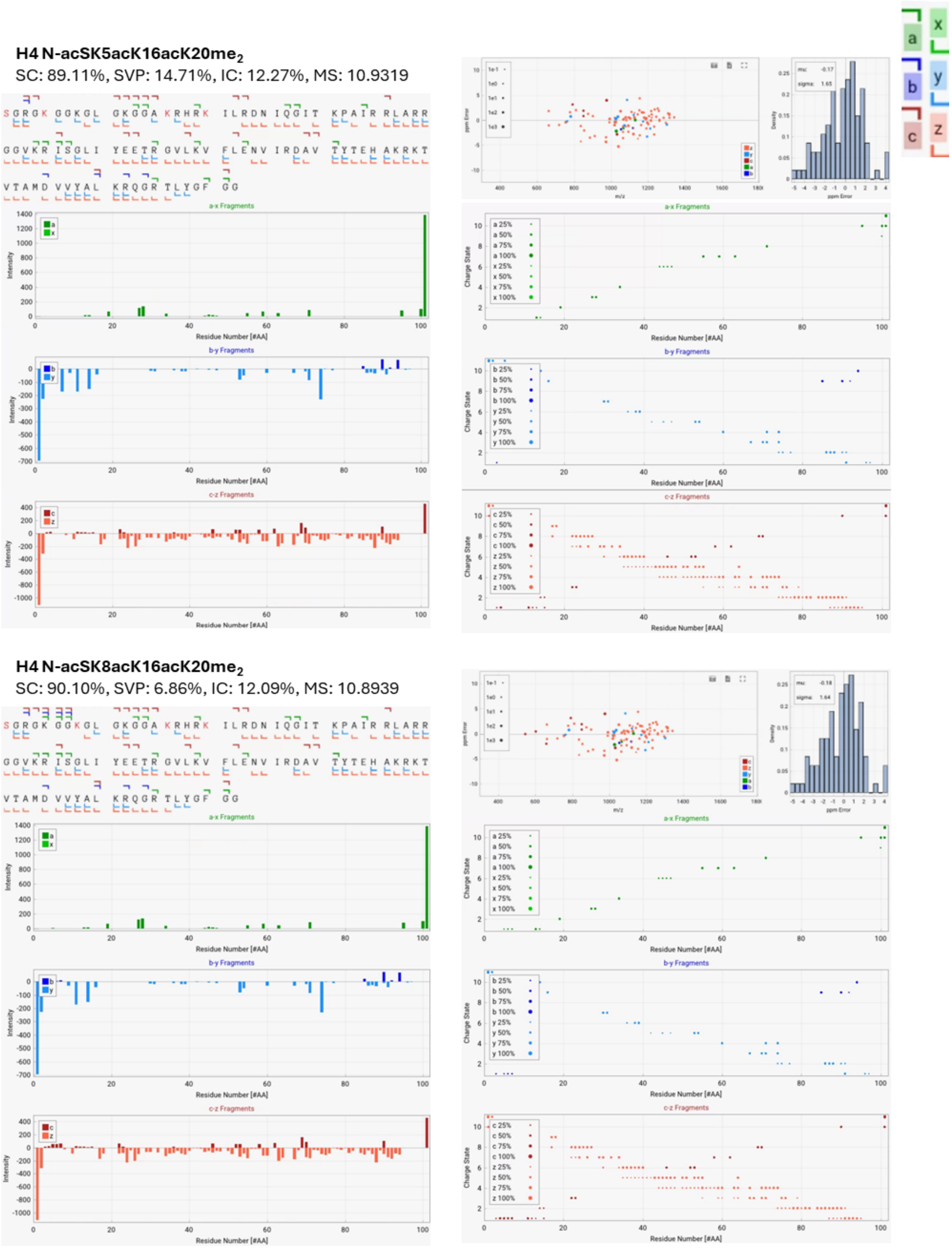

Figure S14. Top-down proteoform confirmation of *A. cervicornis* H4 variant PTM patterns with ±10 ppm error: N-acSK5acK16acK20me$_2$ and N-acSK8acK16acK20me$_2$.

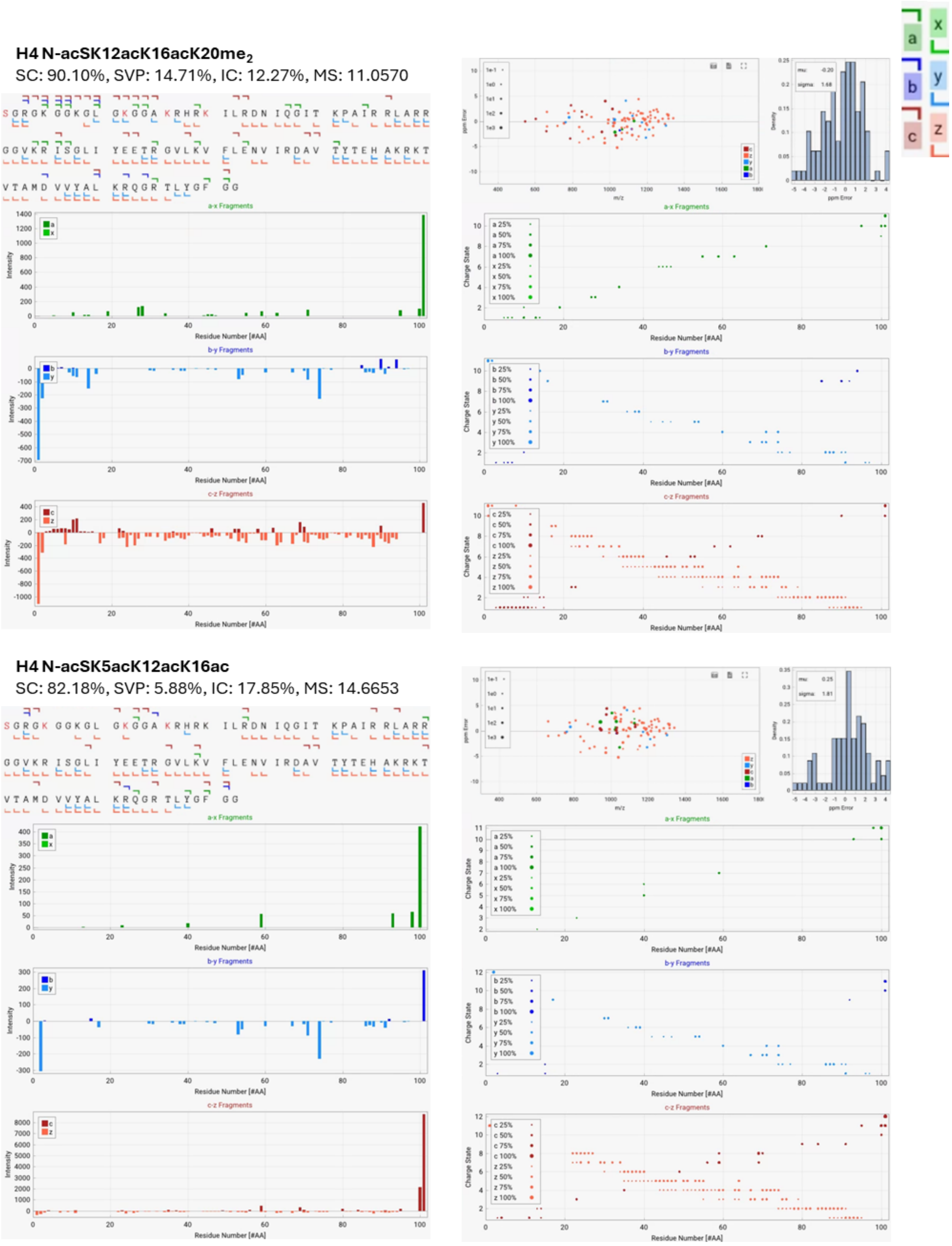

Figure S15. Top-down proteoform confirmation of *A. cervicornis* H4 variant PTM patterns with ±10 ppm error: N-acSK12acK16acK20me$_1$ and N-acSK5acK12acK16ac.

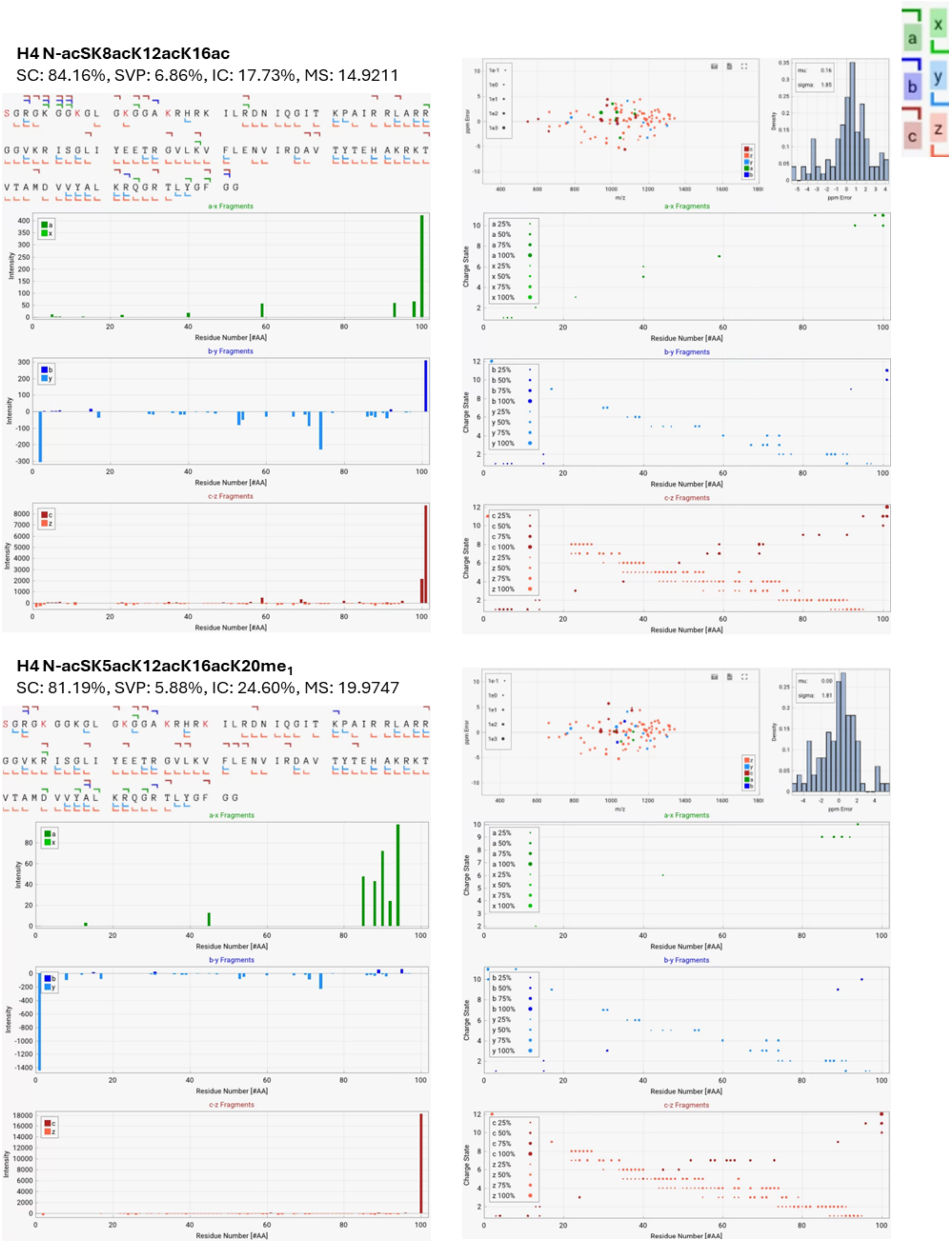

Figure S16. Top-down proteoform confirmation of *A. cervicornis* H4 variant PTM patterns with ±10 ppm error: N-acSK8acK12acK16ac and N-acSK5acK12acK16acK20me$_1$.

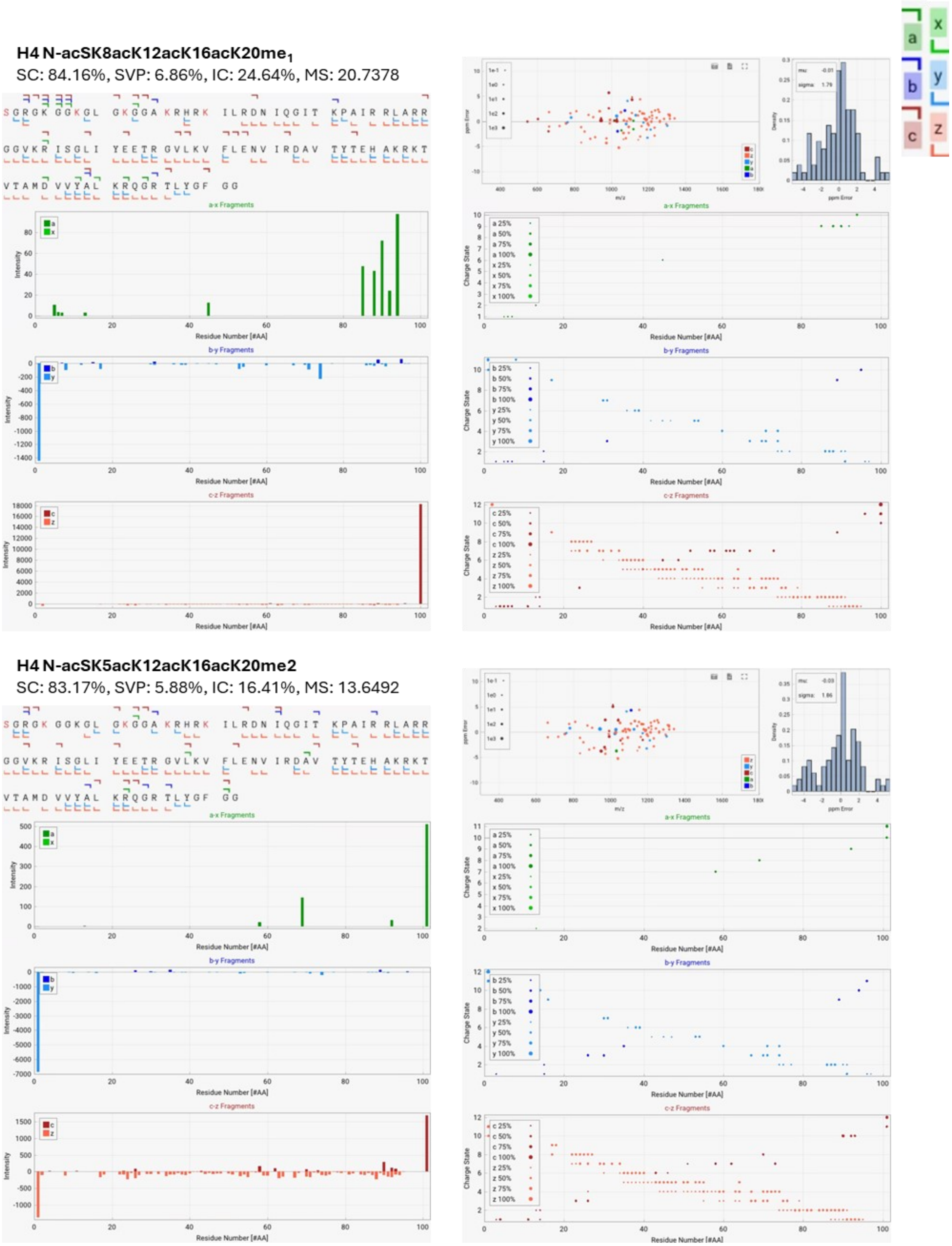

Figure S17. Top-down proteoform confirmation of *A. cervicornis* H4 variant PTM patterns with ±10 ppm error: N-acSK8acK12acK16acK20me$_1$ and N-acSK5acK12acK16acK20me$_2$.

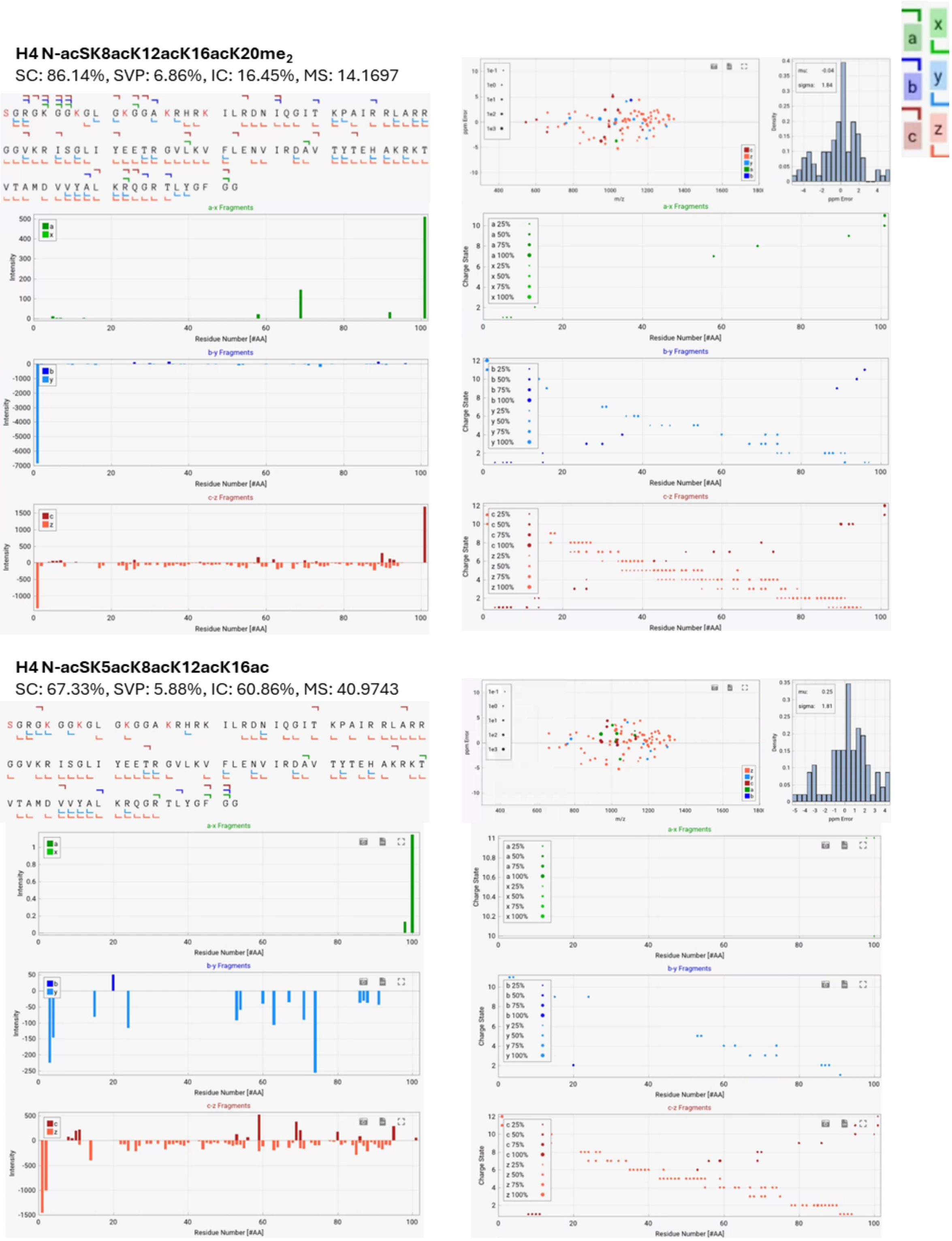

Figure S18. Top-down proteoform confirmation of *A. cervicornis* H4 variant PTM patterns with ±10 ppm error: N-acSK8acK12acK16acK20me$_2$ and N-acSK5acK8acK12acK16ac.

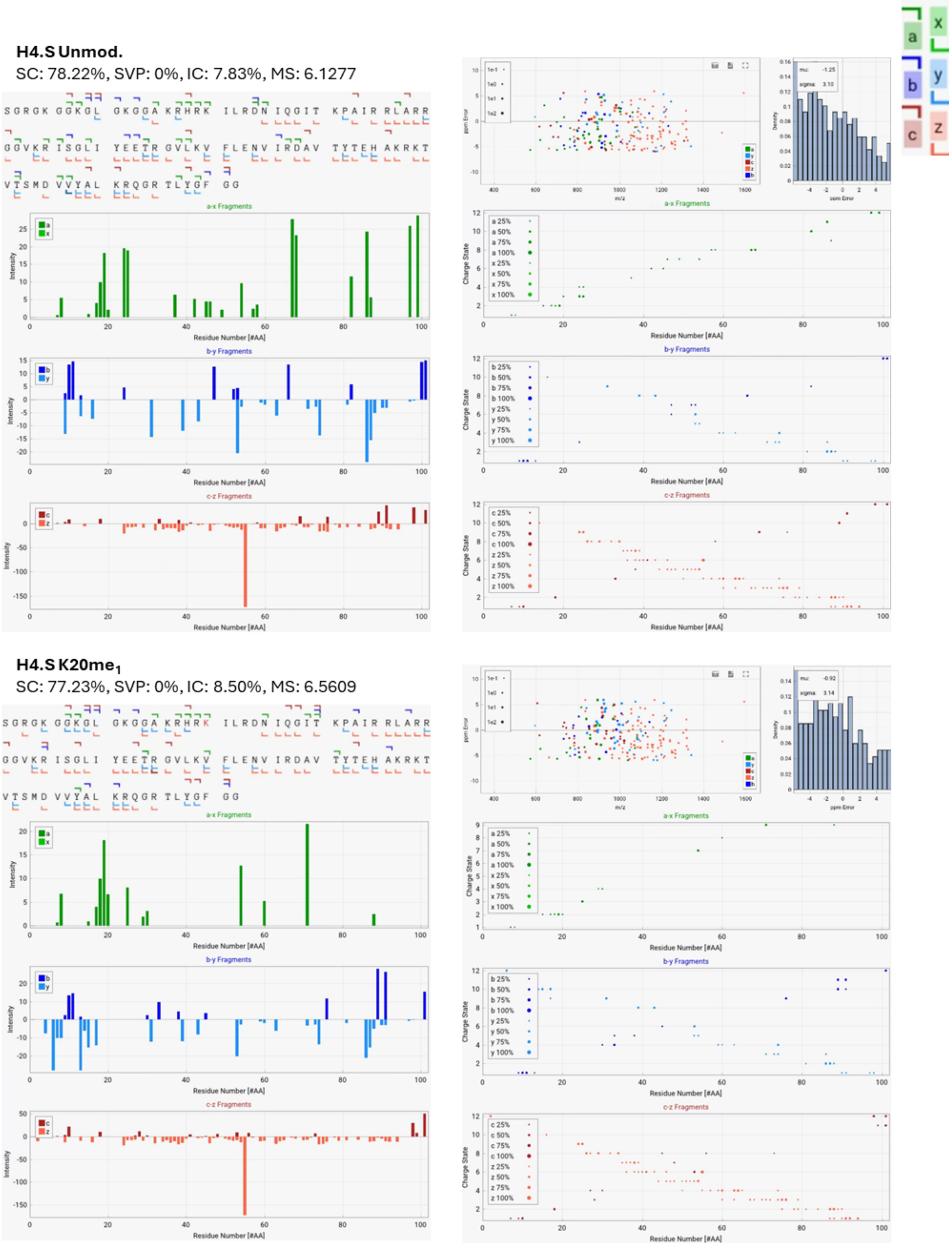

Figure S19. Top-down proteoform confirmation of *A. cervicornis* H4.S variant PTM patterns with ±10 ppm error: Unmodified and K20me$_1$.

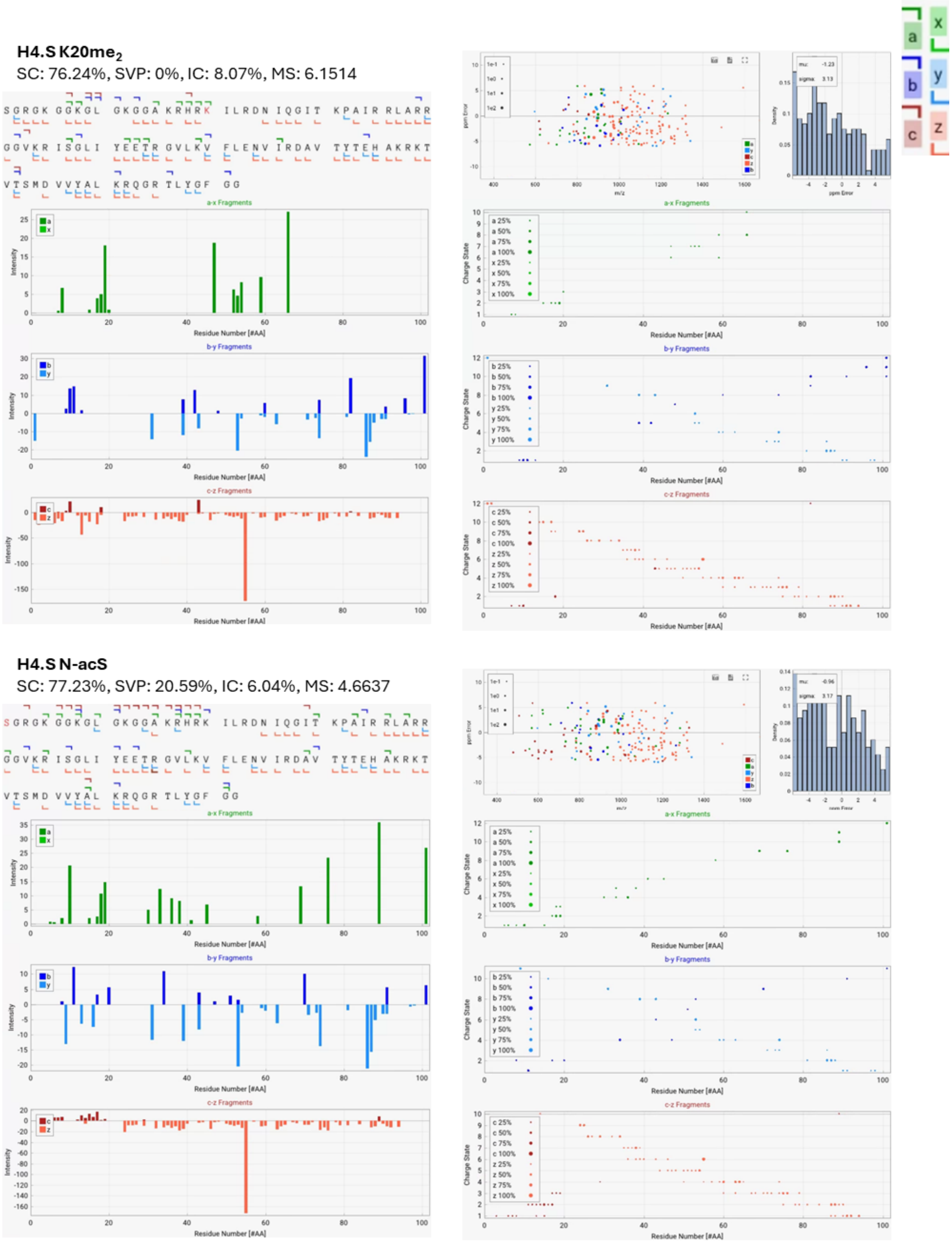

Figure S20. Top-down proteoform confirmation of *A. cervicornis* H4.S variant PTM patterns with ±10 ppm error: K20me$_2$ and N-acS.

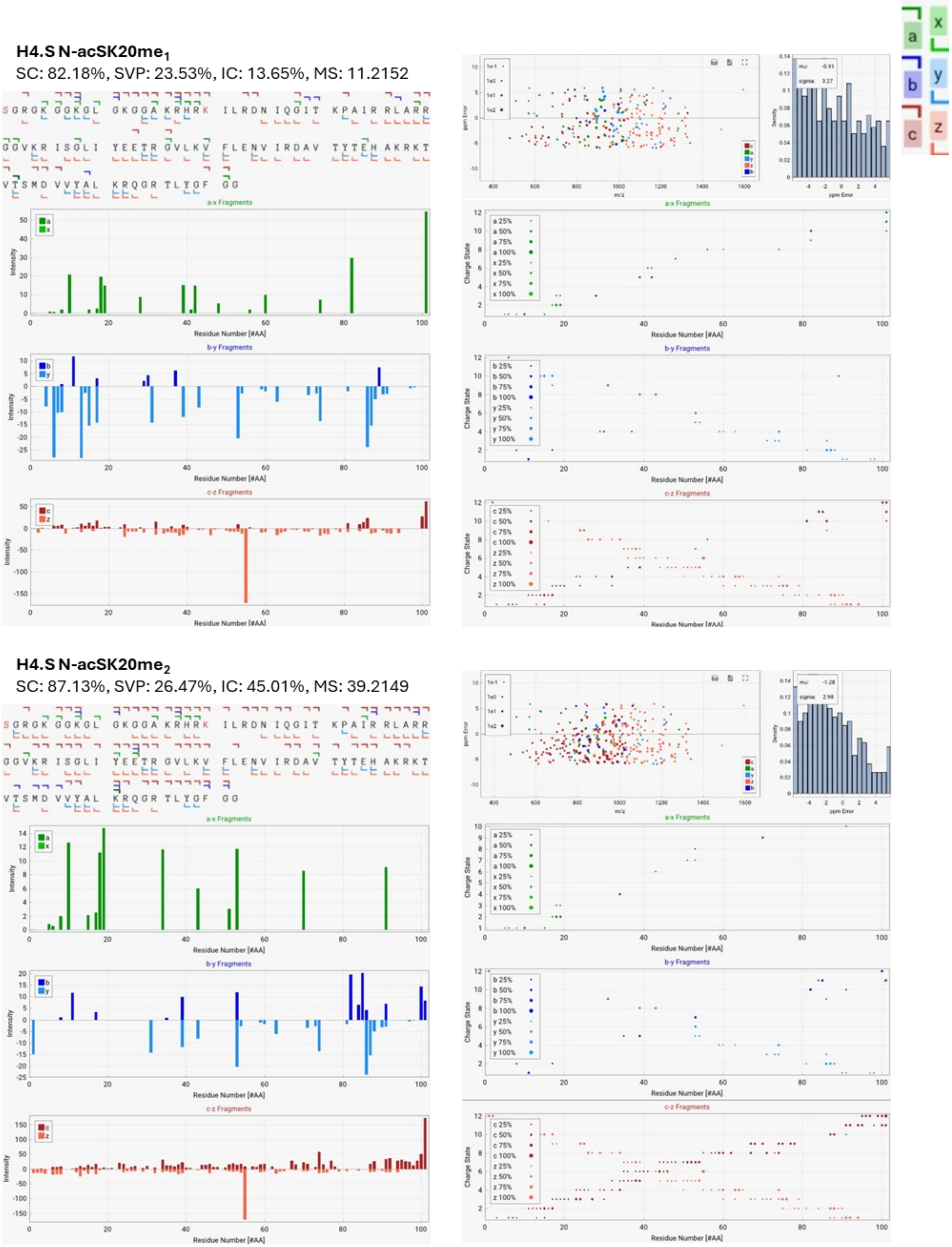

Figure S21. Top-down proteoform confirmation of *A. cervicornis* H4.S variant PTM patterns with ±10 ppm error: N-acSK20me$_1$ and N-acSK20me$_2$.

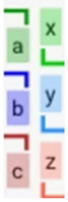

H4.S N-acSK12ac

SC: 75.25%, SVP: 0%, IC: 8.84%, MS: 6.6484

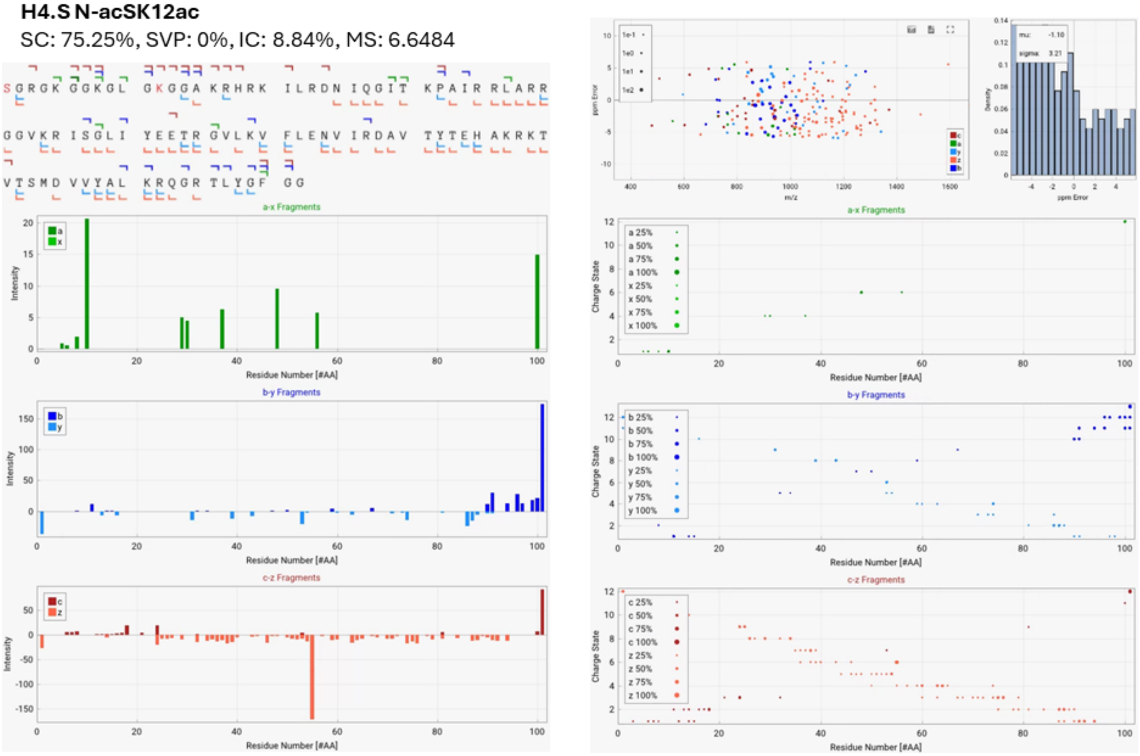

H4.S N-acSK16ac

SC: 75.25%, SVP: 0%, IC: 8.94%, MS: 6.7267

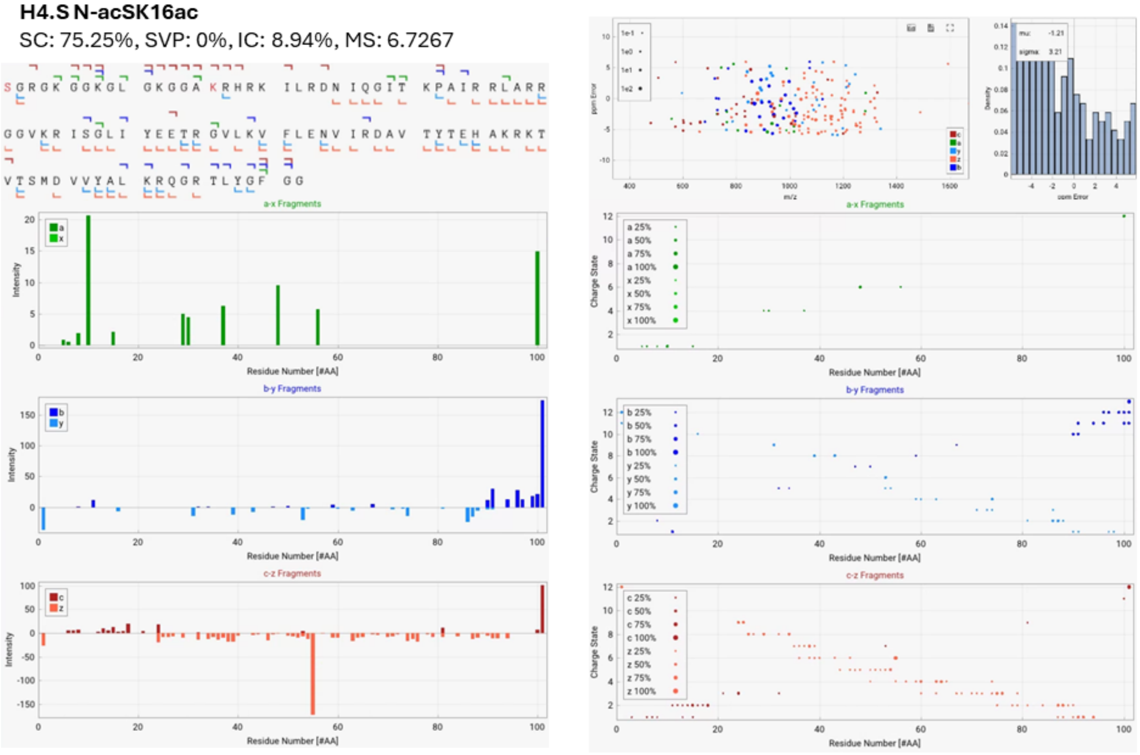

Figure S22. Top-down proteoform confirmation of *A. cervicornis* H4.S variant PTM patterns with ±10 ppm error: N-acSK12ac and N-acSK16ac.

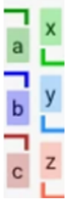

H4.S N-acSK12acK20me$_1$
SC: 74.26%, SVP: 24.51%, IC: 7.63%, MS: 5.6650

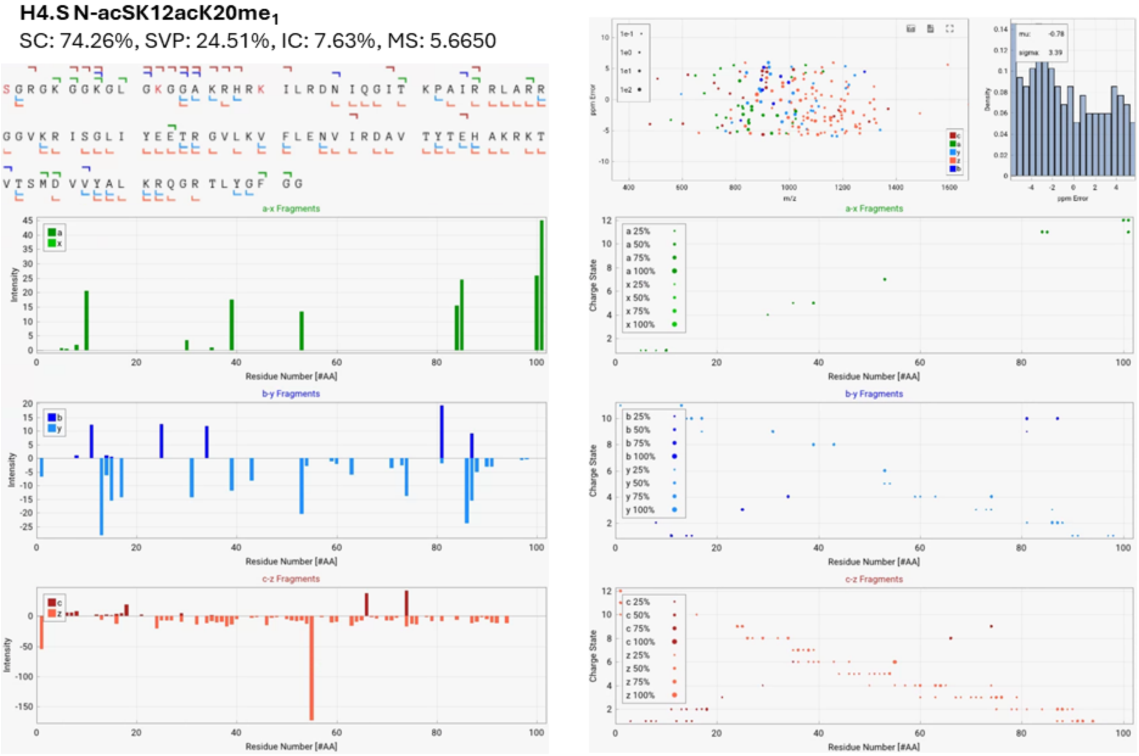

H4.S N-acSK16acK20me$_1$
SC: 74.26%, SVP: 24.51%, IC: 7.33%, MS: 5.4418

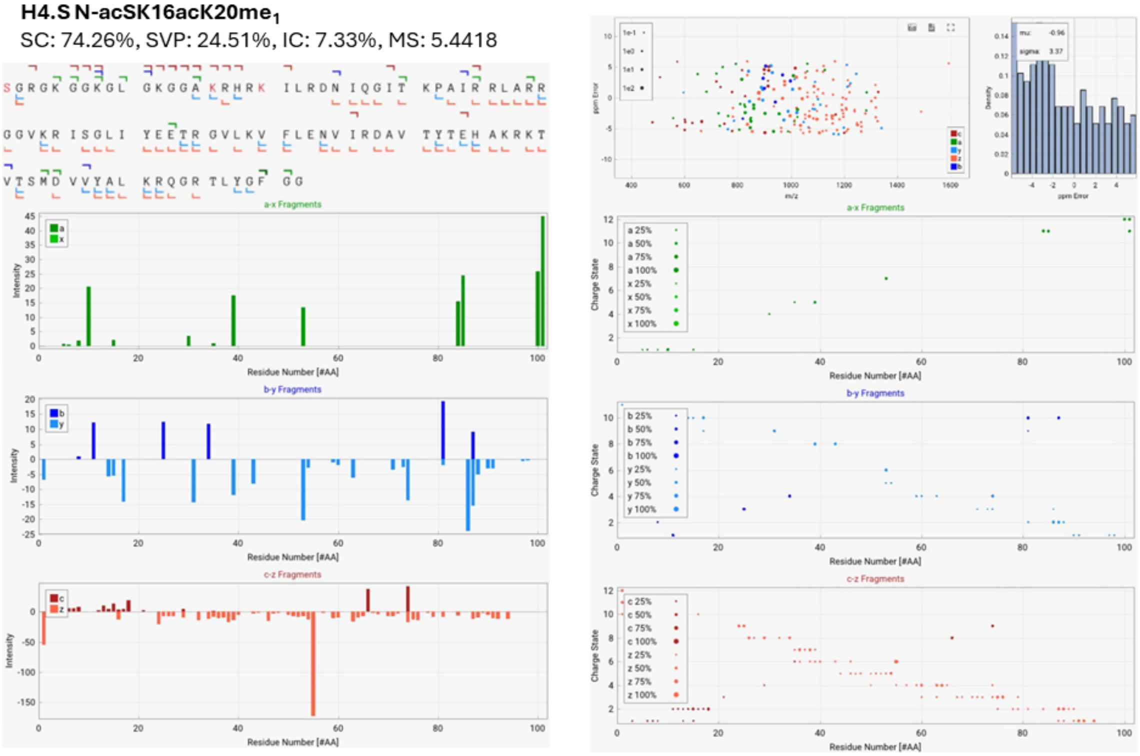

Figure S23. Top-down proteoform confirmation of *A. cervicornis* H4.S variant PTM patterns with ±10 ppm error: N-acSK12acK20me$_1$ and N-acSK16acK20me$_1$.

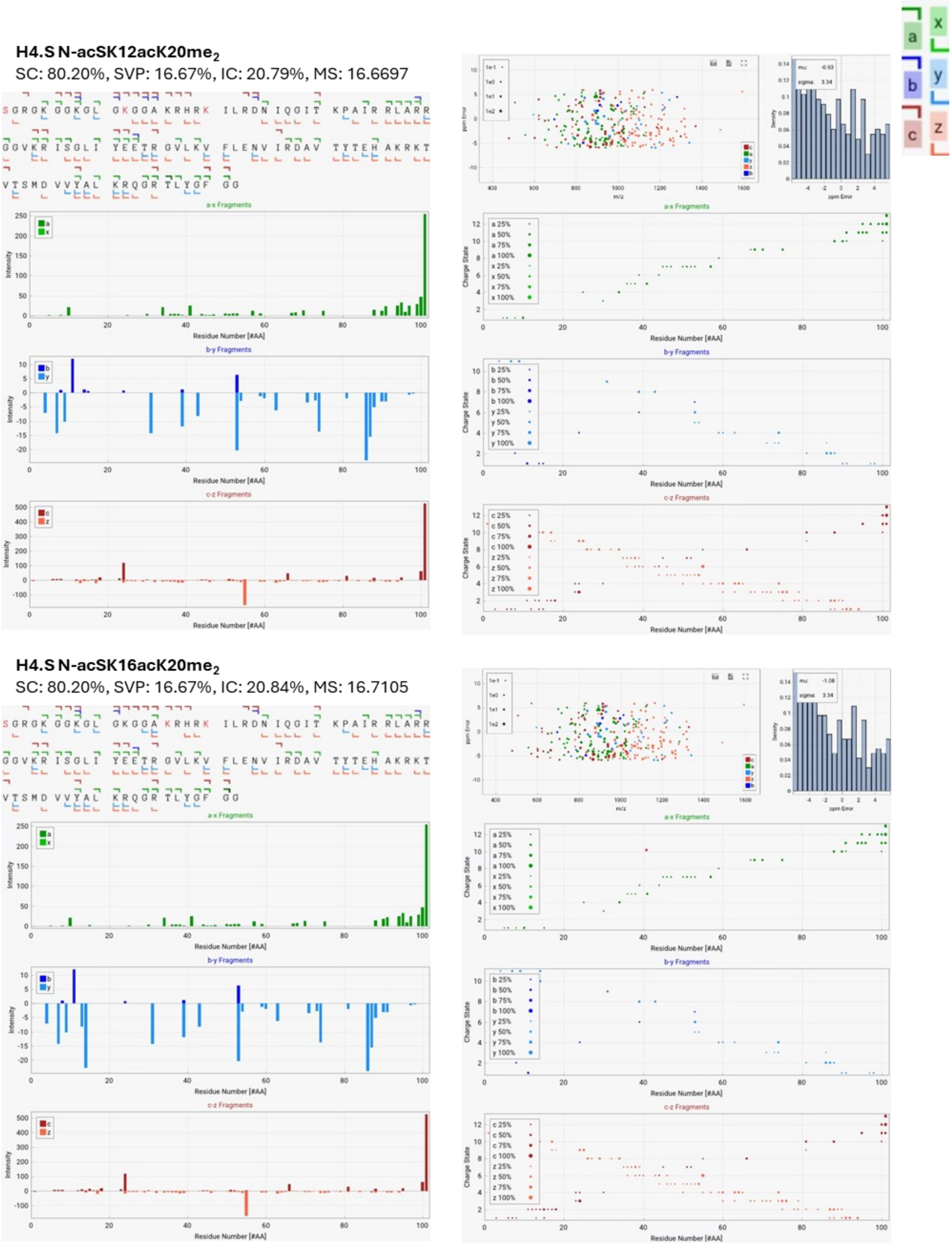

Figure S24. Top-down proteoform confirmation of *A. cervicornis* H4.S variant PTM patterns with ±10 ppm error: N-acSK12acK20me$_2$ and N-acSK16acK20me$_2$.

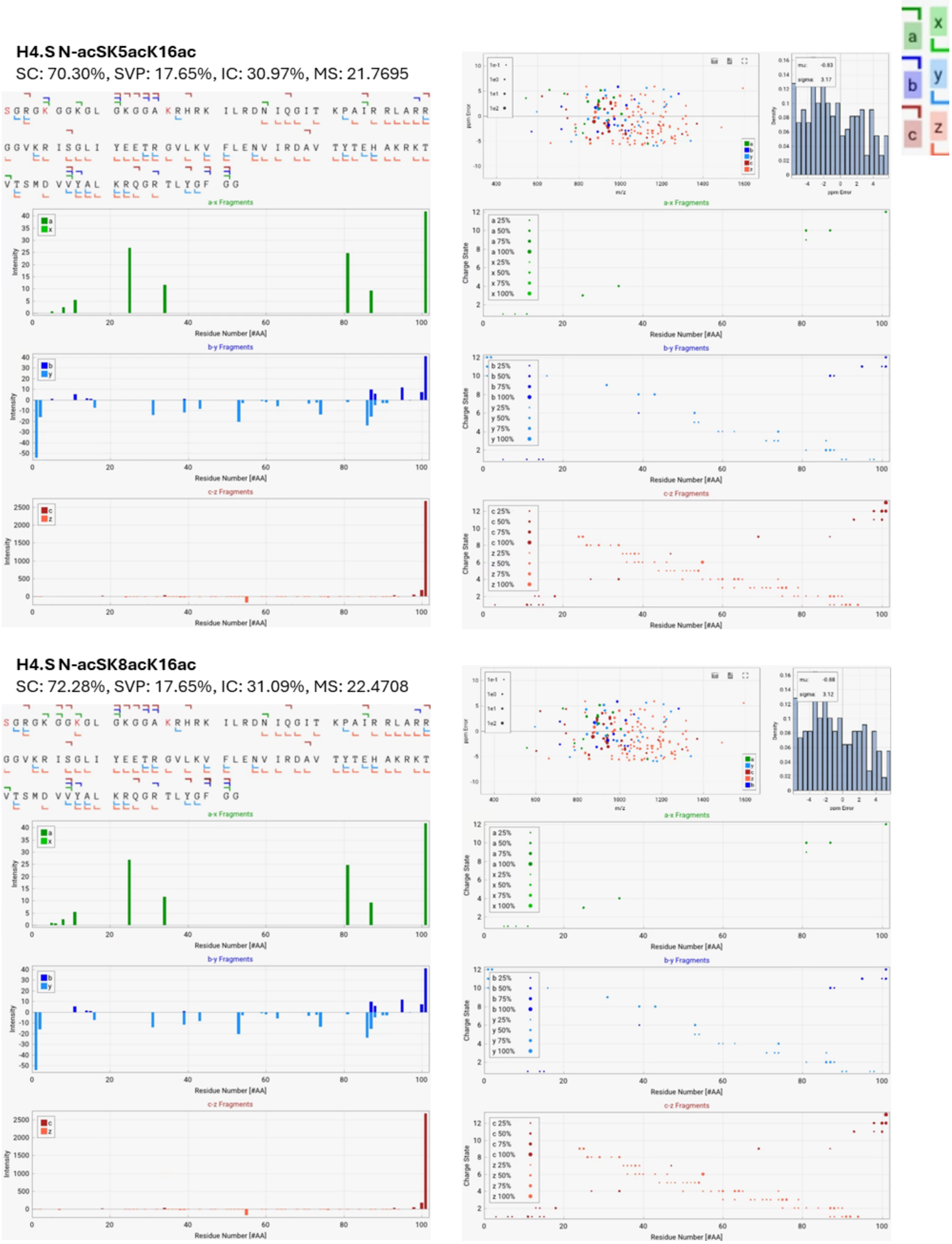

Figure S25. Top-down proteoform confirmation of *A. cervicornis* H4.S variant PTM patterns with ±10 ppm error: N-acSK5acK16ac and N-acSK8acK16ac.

H4.S N-acSK12acK16ac

SC: 74.26%, SVP: 17.65%, IC: 31.31%, MS: 23.2519

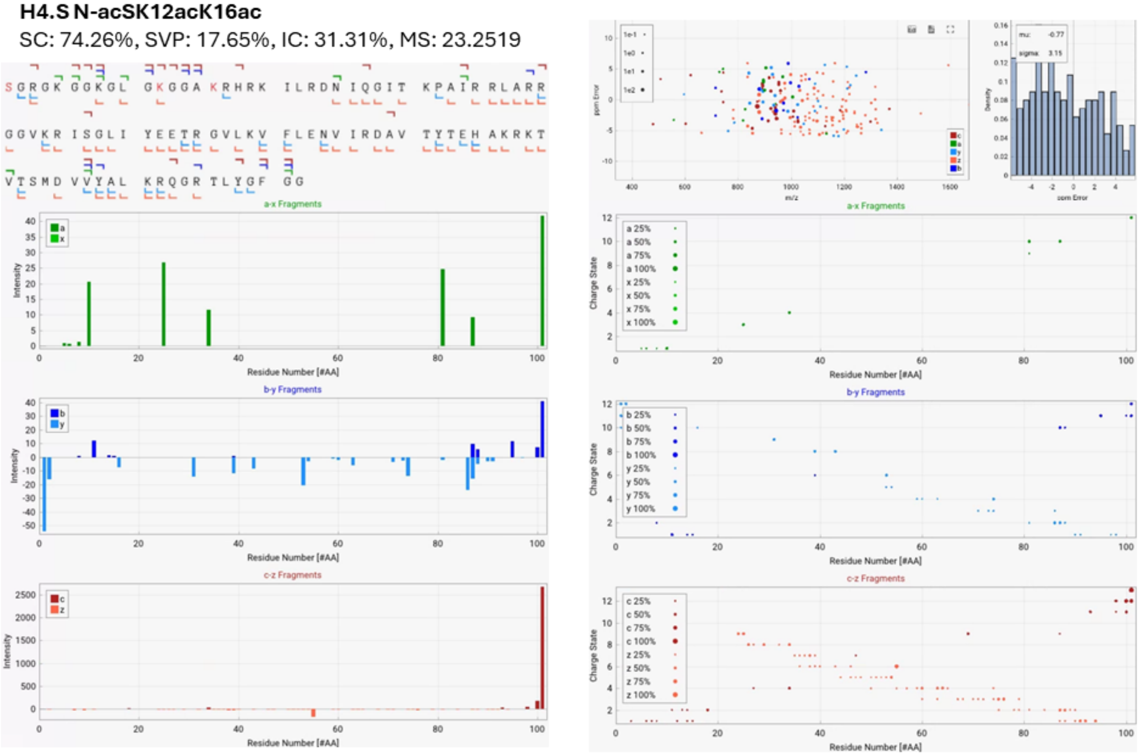

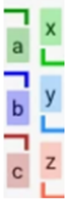

H4.S N-acSK5acK16acK20me$_1$

SC: 71.29%, SVP: 24.51%, IC: 9.43%, MS: 6.7235

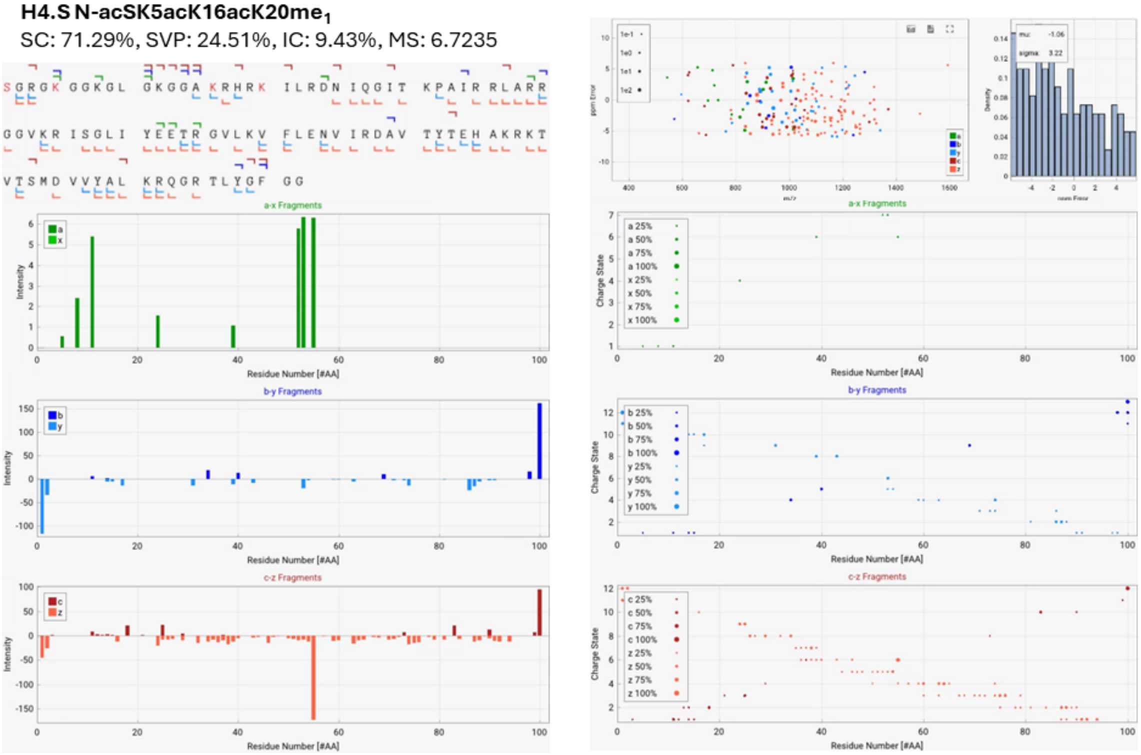

Figure S26. Top-down proteoform confirmation of *A. cervicornis* H4.S variant PTM patterns with ±10 ppm error: N-acSK12acK16ac and N-acSK5acK16acK20me$_1$.

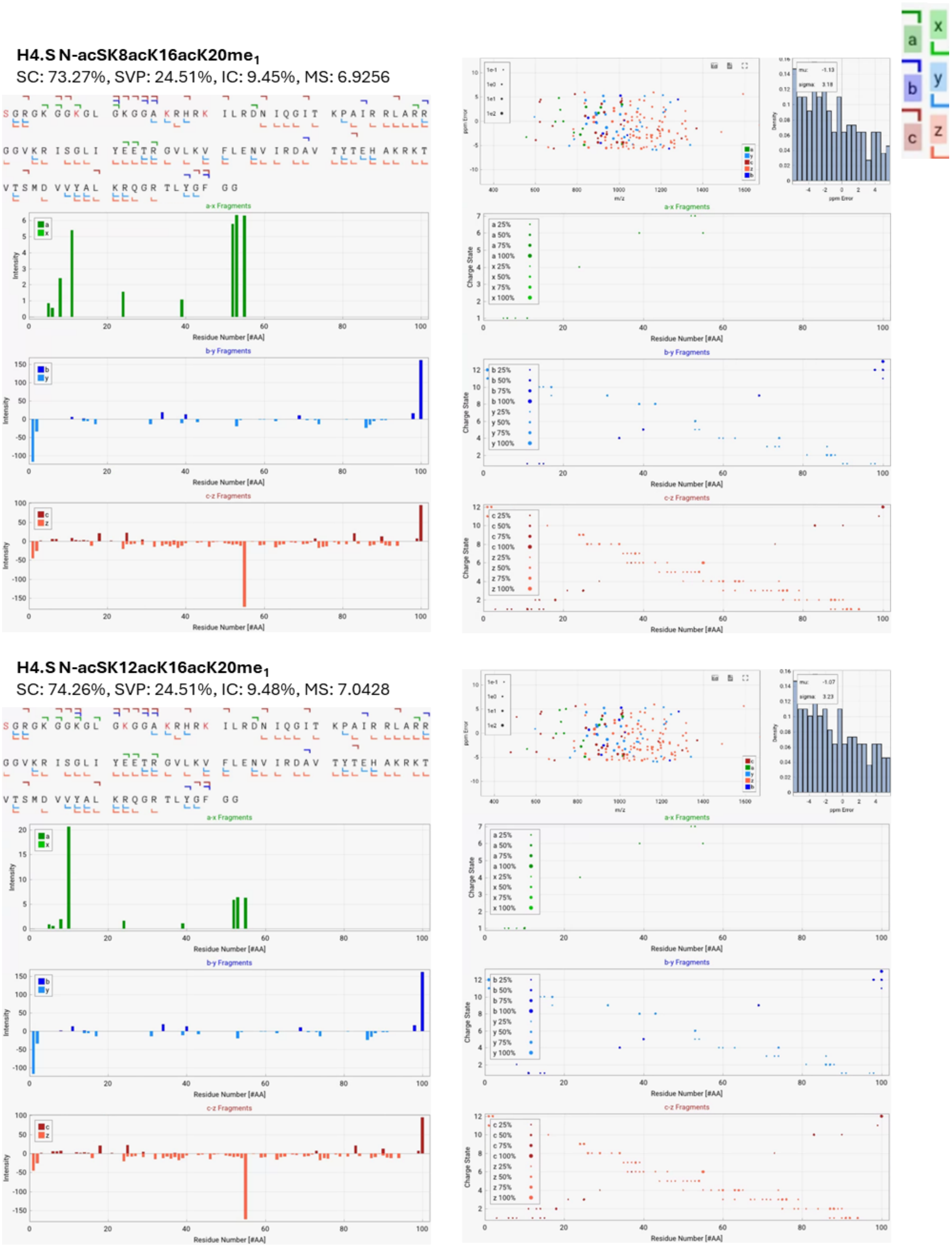

Figure S27 .Top-down proteoform confirmation of *A. cervicornis* H4.S variant PTM patterns with ±10 ppm error: N-acSK8acK16acK20me$_1$ and N-acSK12acK16acK20me$_1$.

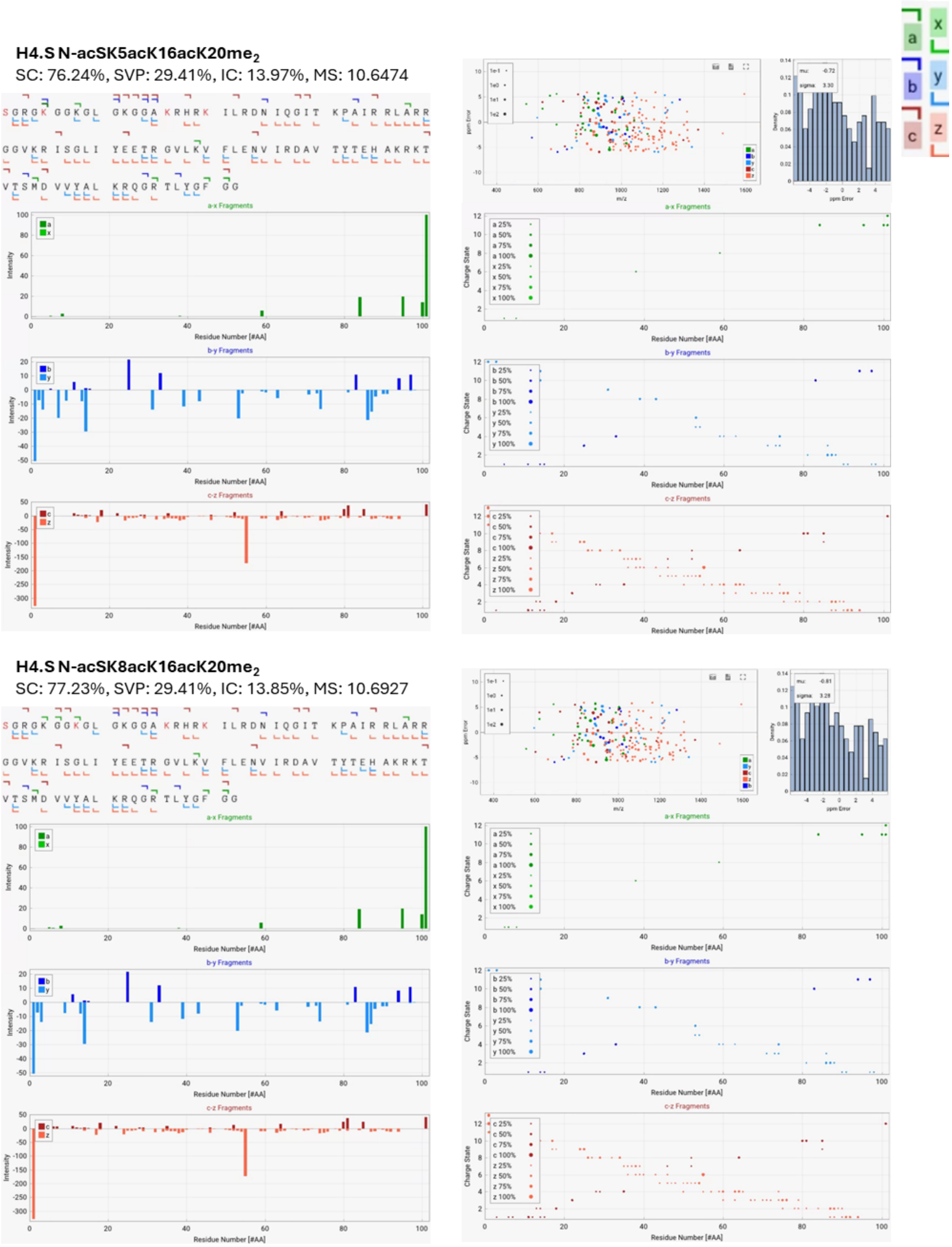

Figure S28. Top-down proteoform confirmation of *A. cervicornis* H4.S variant PTM patterns with ±10 ppm error: N-acSK5acK16acK20me$_2$ and N-acSK8acK16acK20me$_2$.

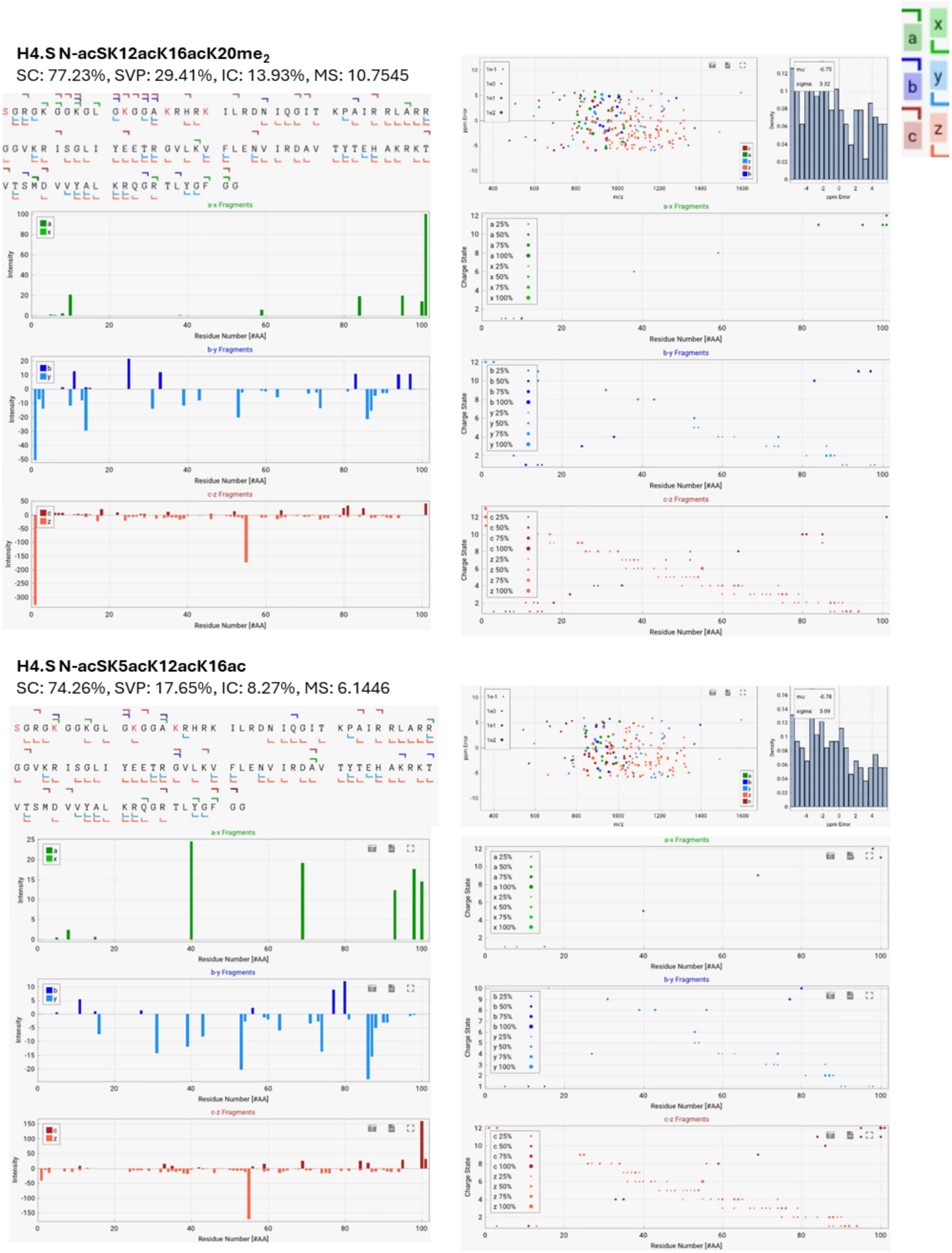

Figure S29. Top-down proteoform confirmation of *A. cervicornis* H4.S variant PTM patterns with ±10 ppm error: N-acSK12acK16acK20me$_2$ and N-acSK5acK12acK16ac.

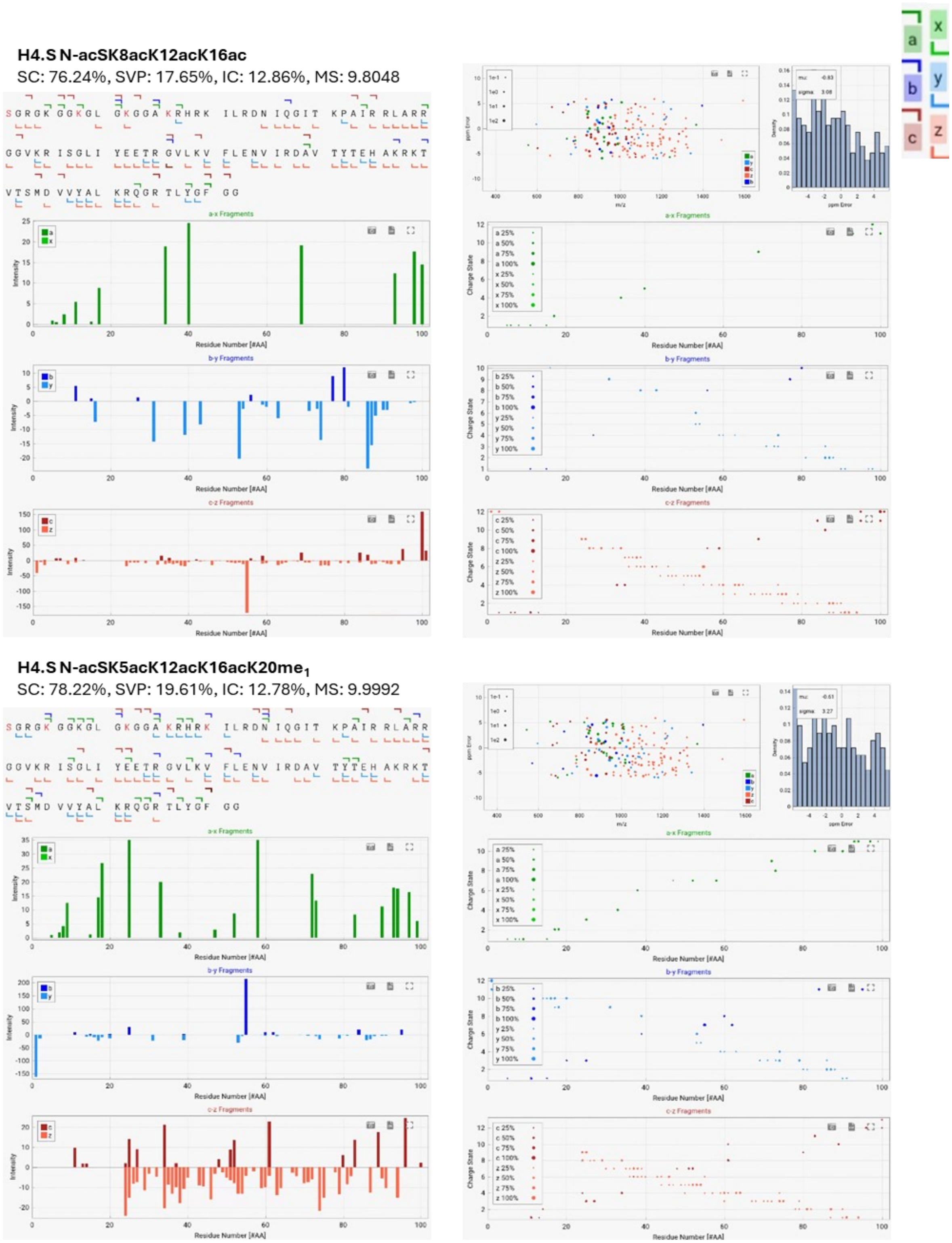

Figure S30. Top-down proteoform confirmation of *A. cervicornis* H4.S variant PTM patterns with ±10 ppm error: N-acSK8acK12acK16ac and N-acSK5acK12acK16acK20me$_1$.

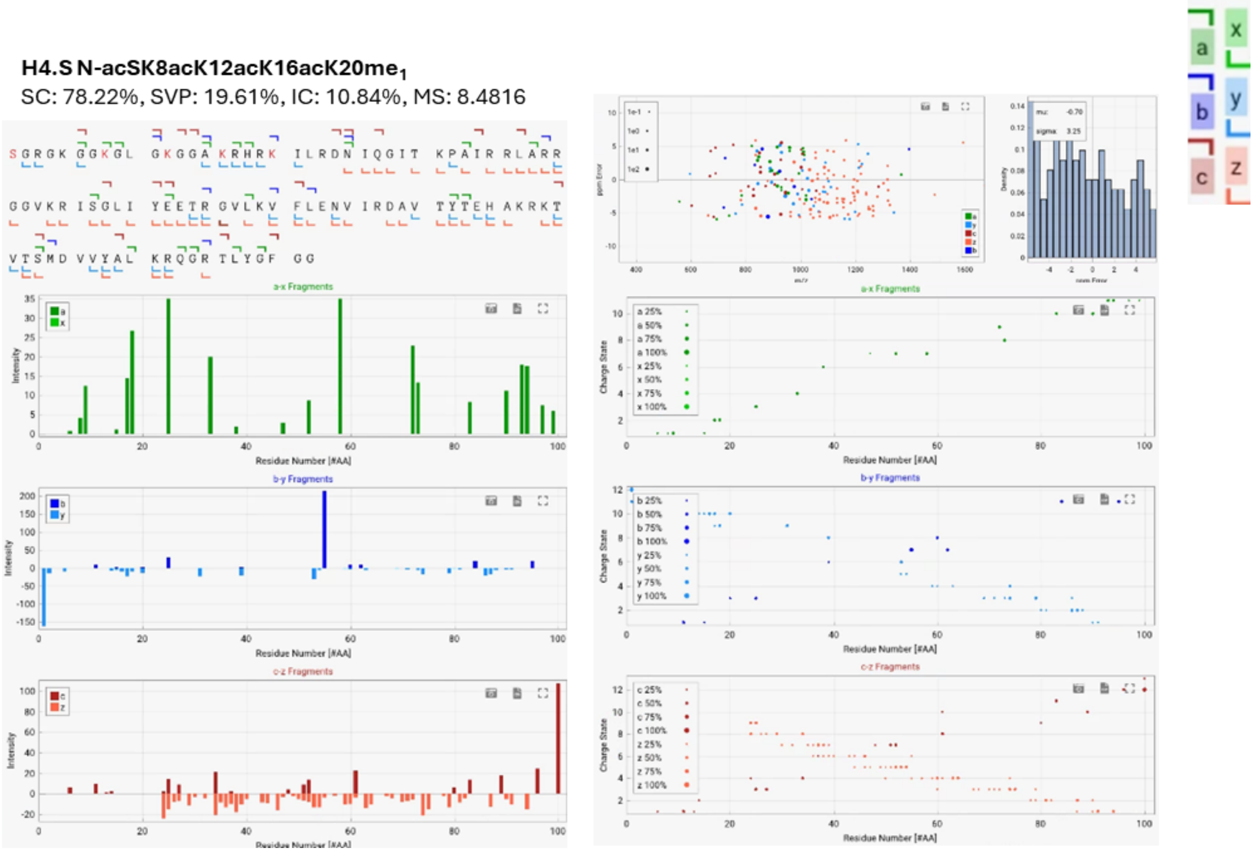

Figure S31. Top-down proteoform confirmation of *A. cervicornis* H4.S variant PTM patterns with ±10 ppm error: N-acSK8acK12acK16acK20me$_1$.

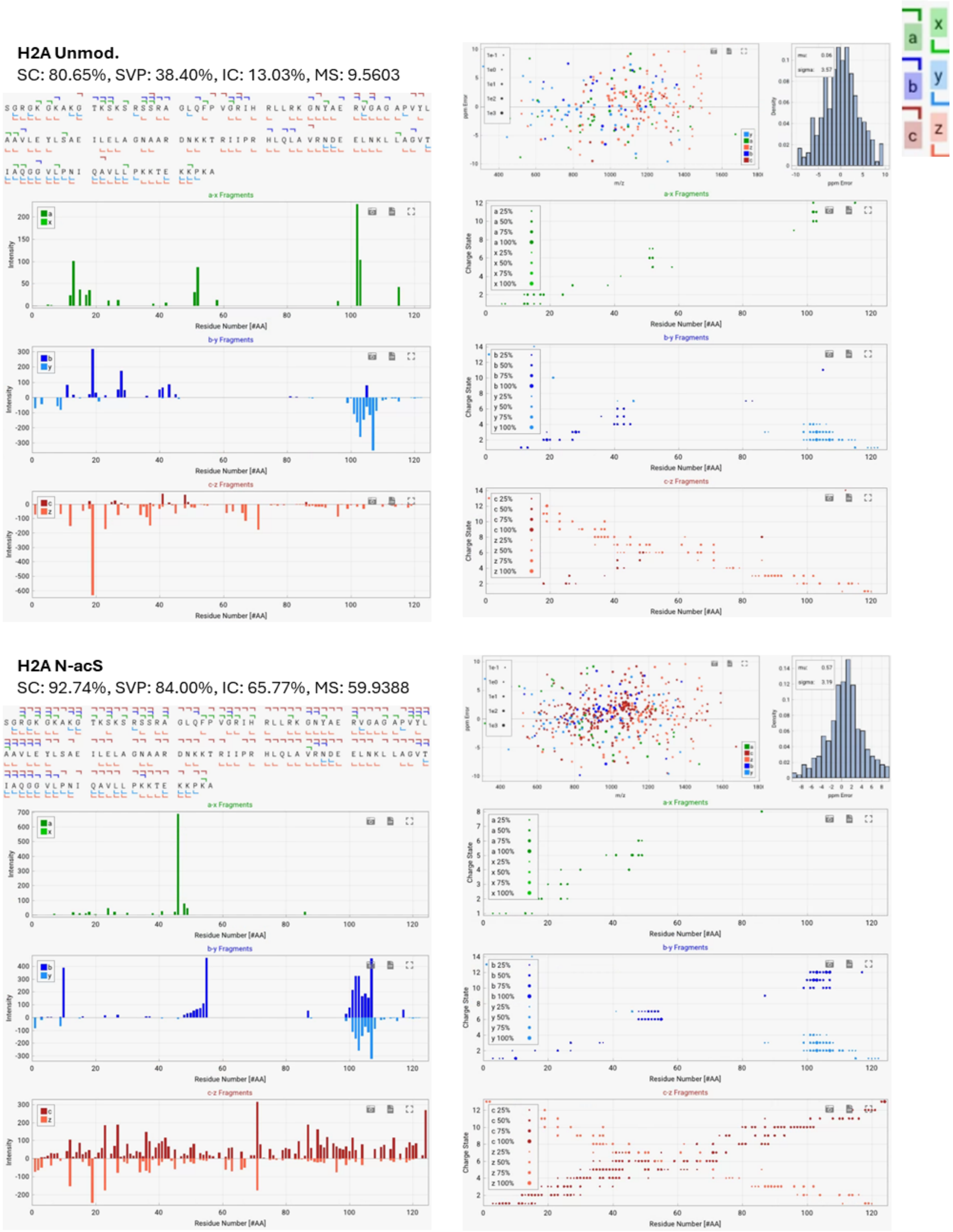

Figure S32. Top-down proteoform confirmation of *A. cervicornis* H2A variant PTM patterns with ±10 ppm error: Unmodified and N-acS.

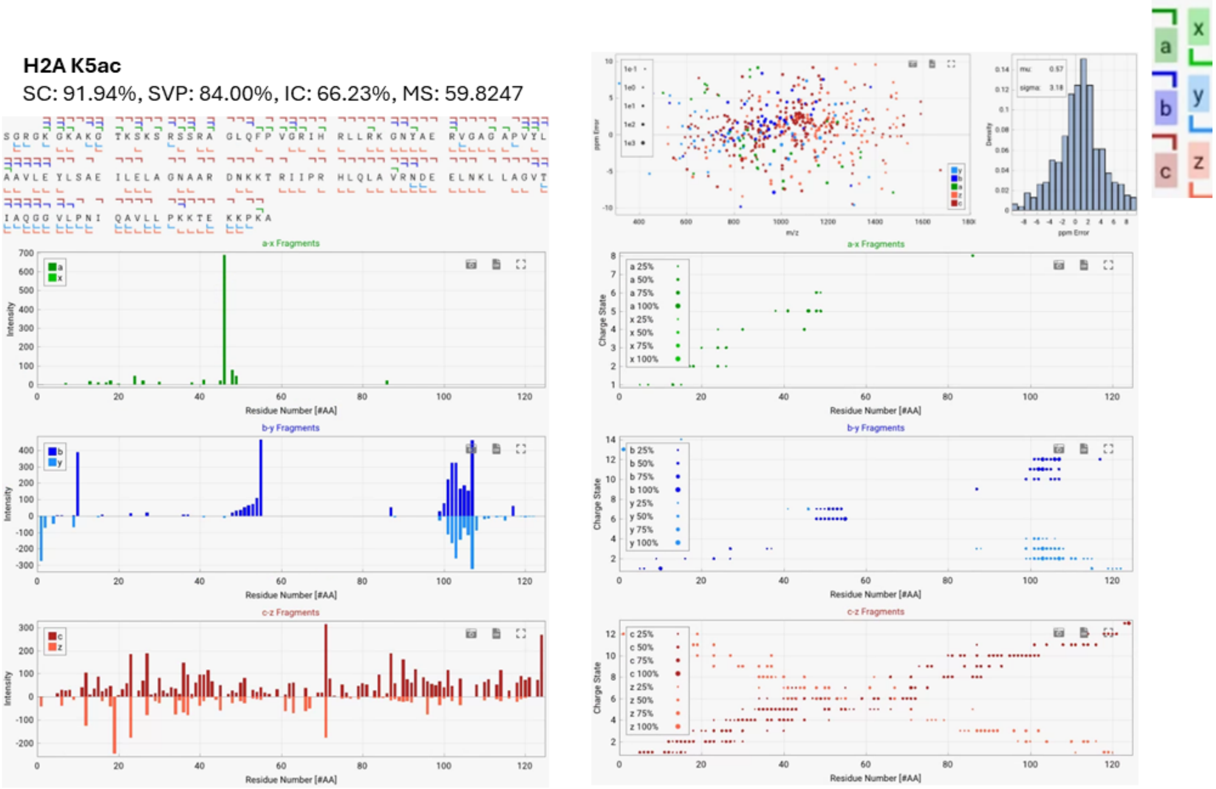

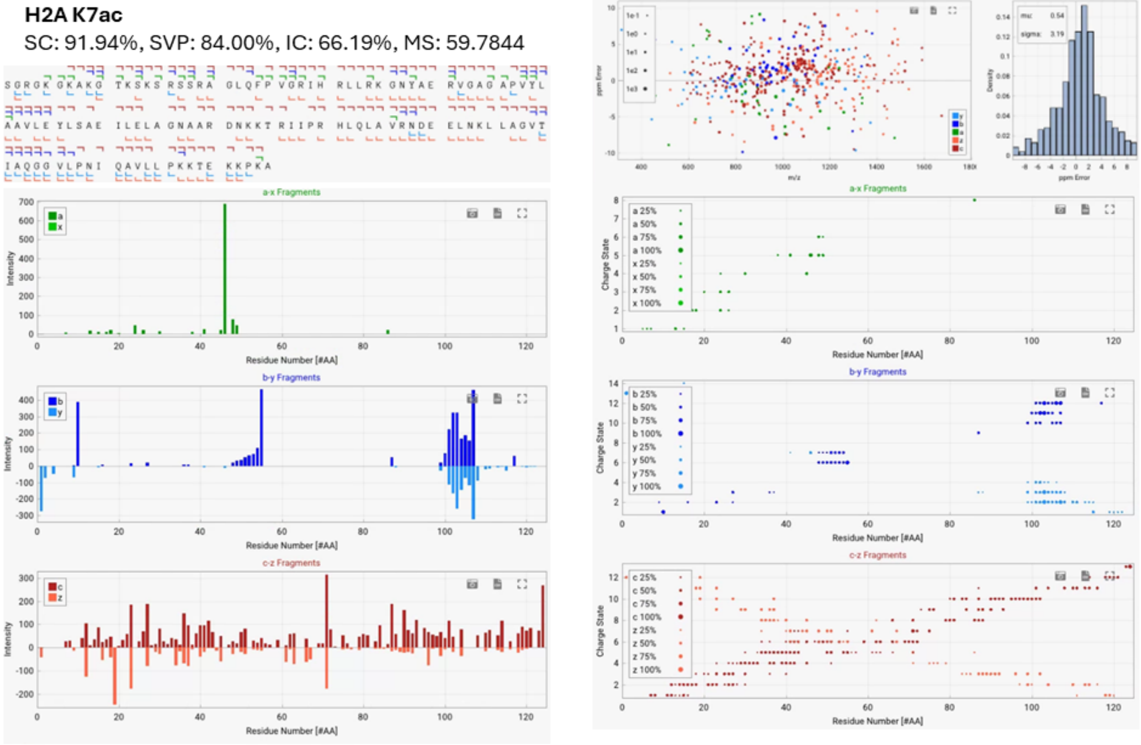

Figure S33. Top-down proteoform confirmation of *A. cervicornis* H2A variant PTM patterns with ±10 ppm error: K5ac and K7ac.

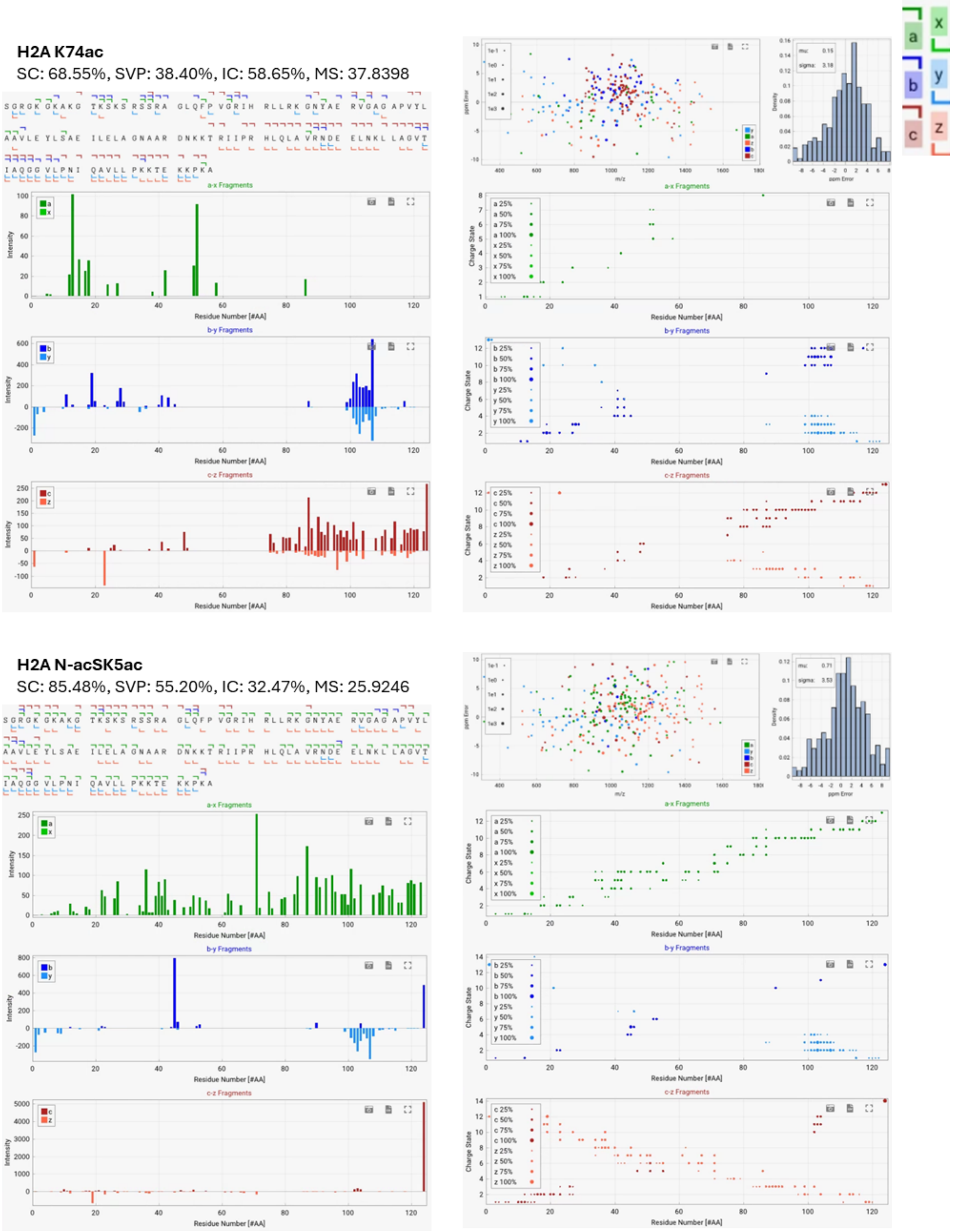

Figure S34. Top-down proteoform confirmation of *A. cervicornis* H2A variant PTM patterns with ±10 ppm error: K74ac and N-acSK5ac.

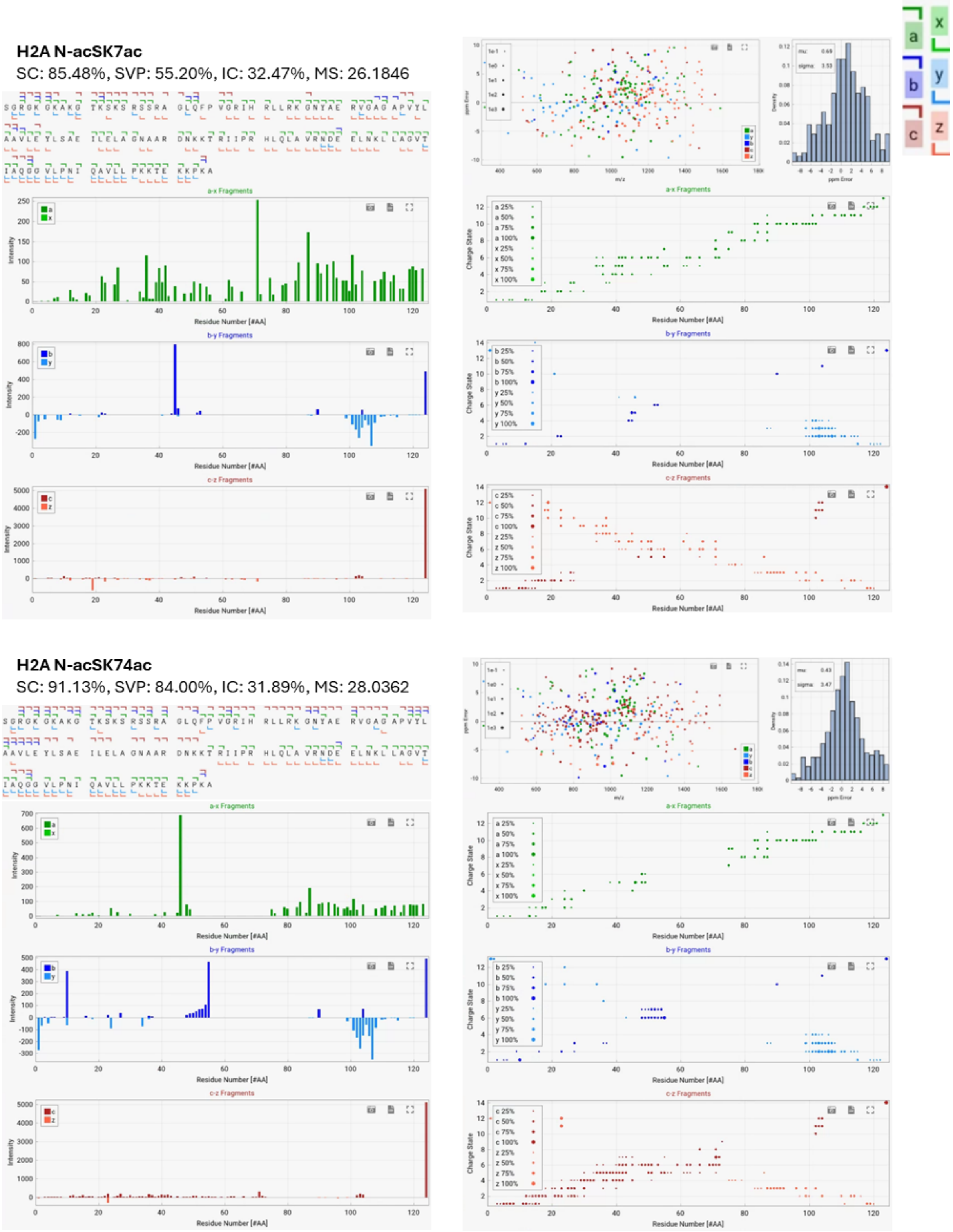

Figure S35. Top-down proteoform confirmation of *A. cervicornis* H2A variant PTM patterns with ±10 ppm error: N-acSK7ac and N-acSK74ac.

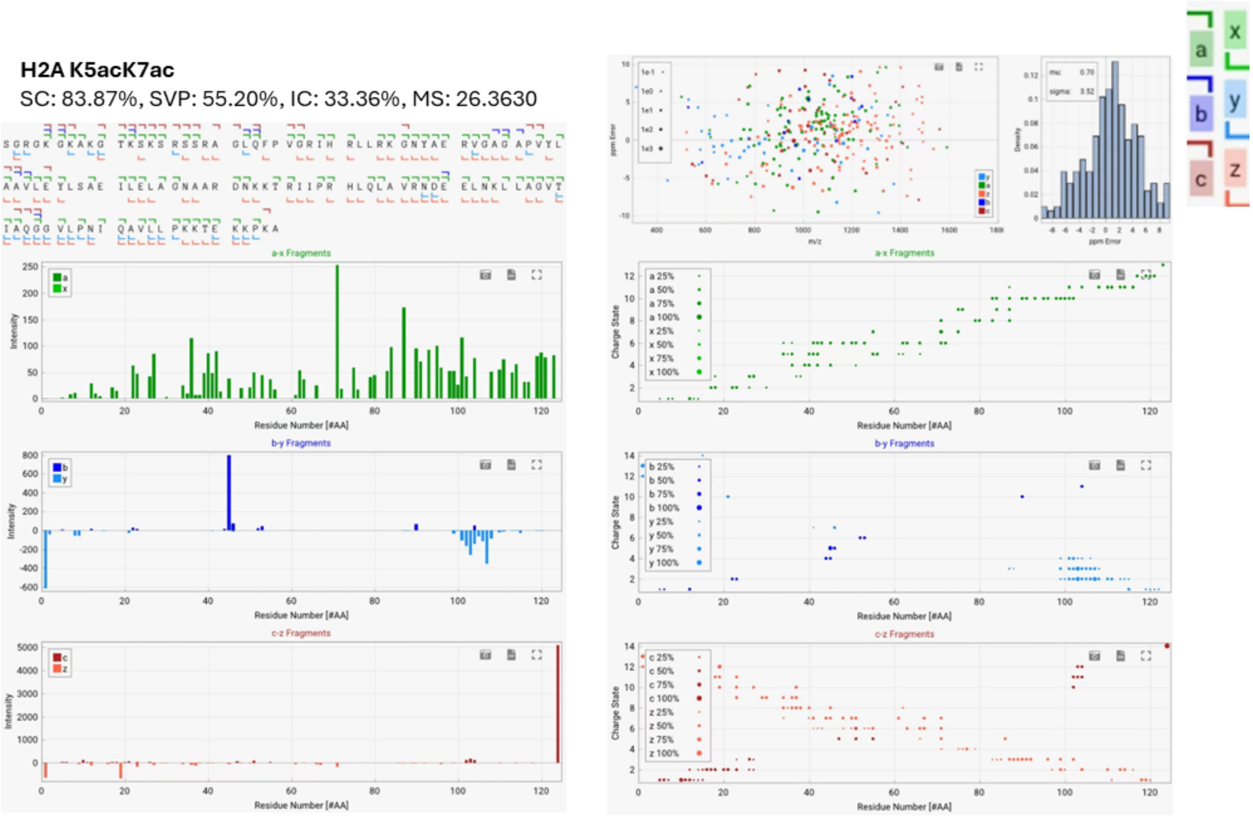

Figure S36. Top-down proteoform confirmation of *A. cervicornis* H2A variant PTM patterns with ±10 ppm error: K5acK7ac.

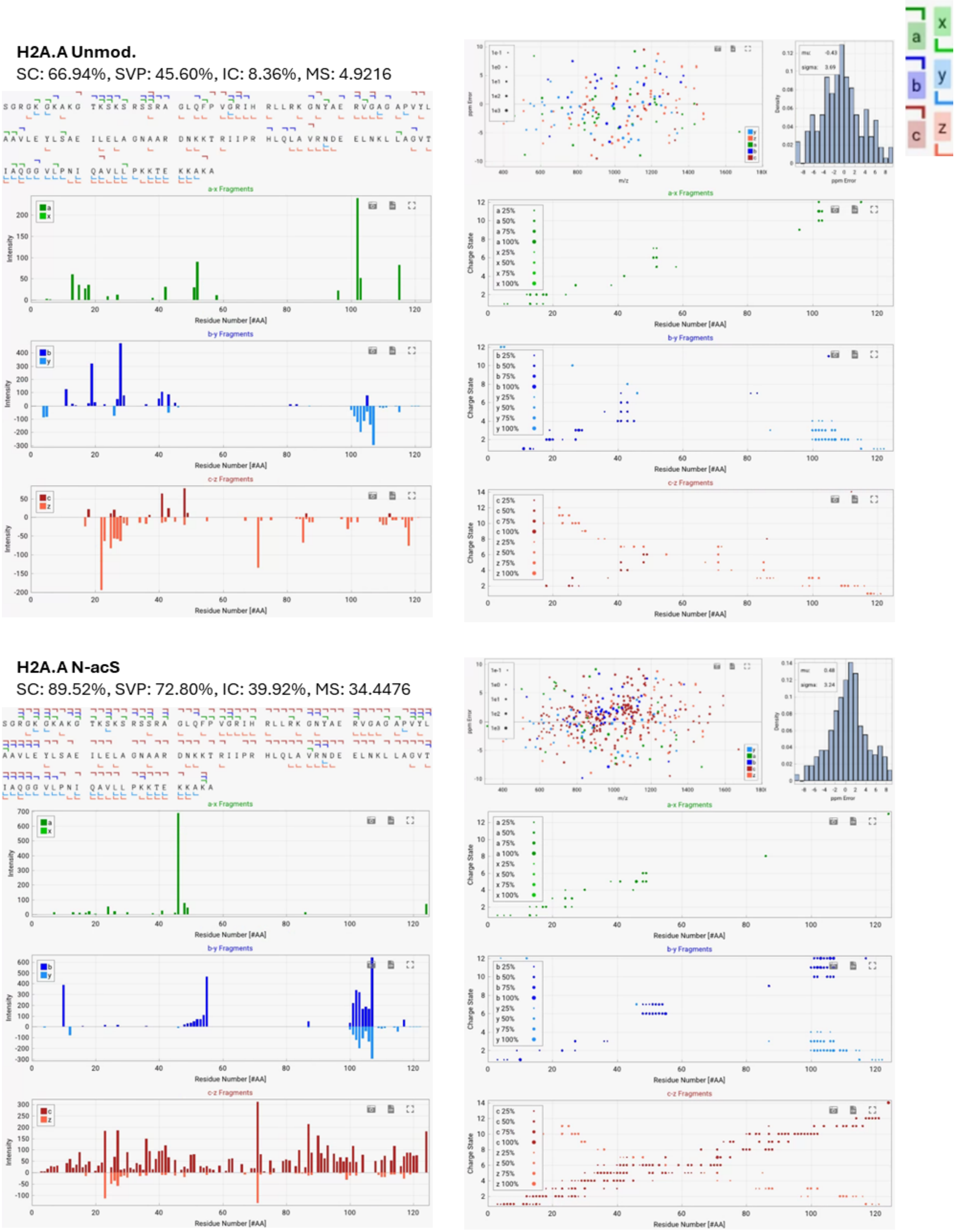

Figure S37. Top-down proteoform confirmation of *A. cervicornis* H2A.A variant PTM patterns with ±10 ppm error: Unmodified and N-acS.

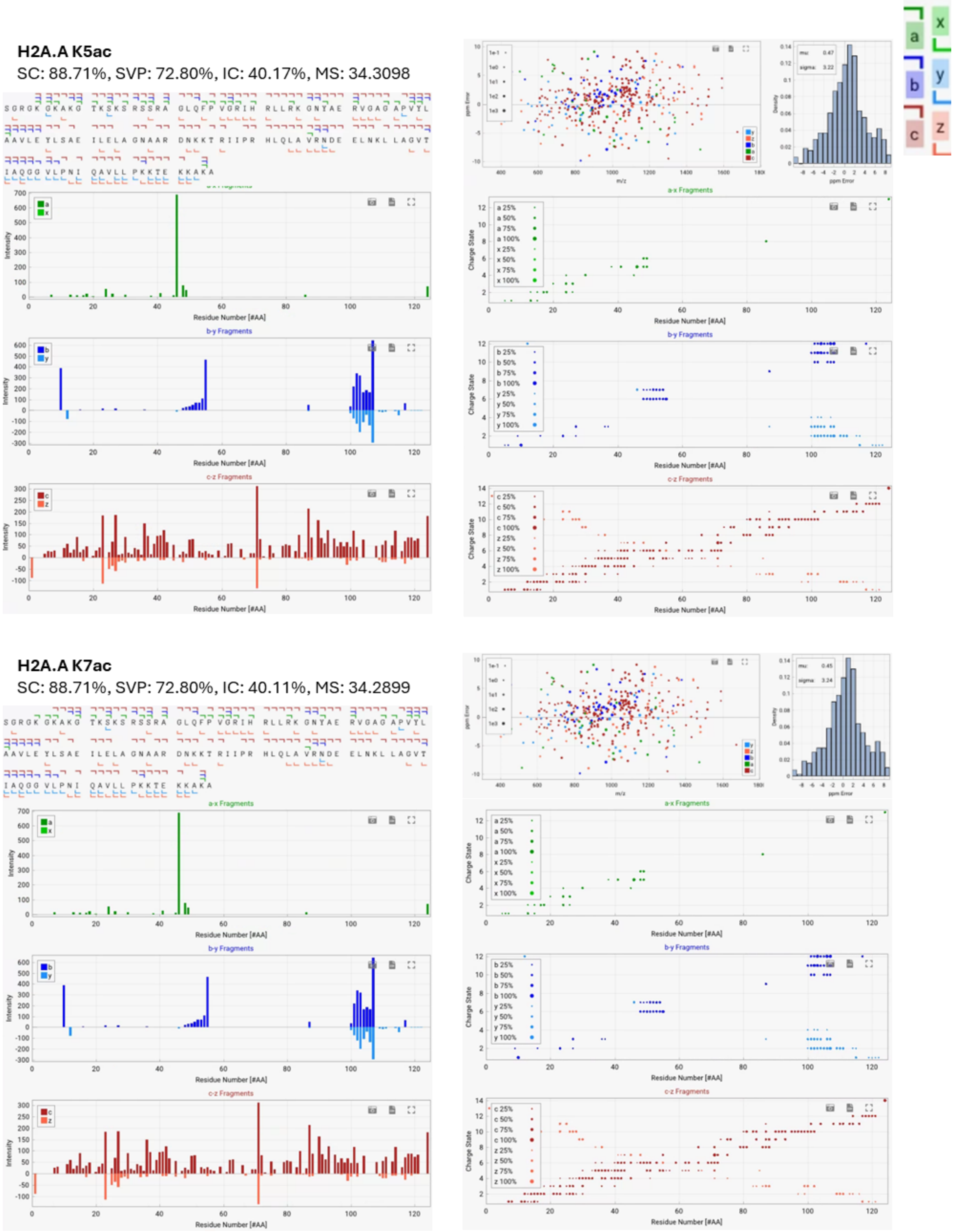

Figure S38. Top-down proteoform confirmation of *A. cervicornis* H2A.A variant PTM patterns with ±10 ppm error: K5ac and K7ac.

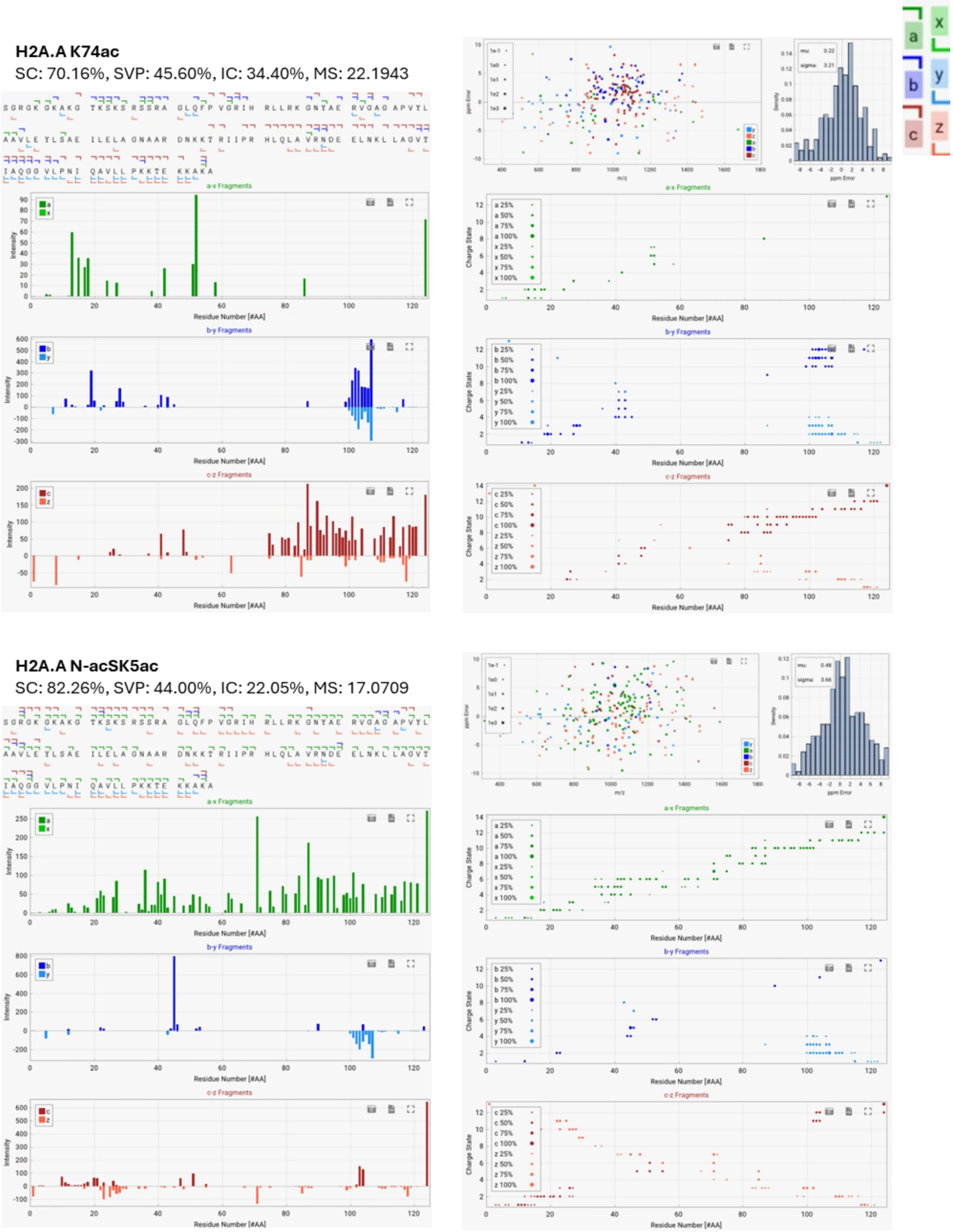

Figure S39. Top-down proteoform confirmation of *A. cervicornis* H2A.A variant PTM patterns with ±10 ppm error: K74ac and N-acSK5ac.

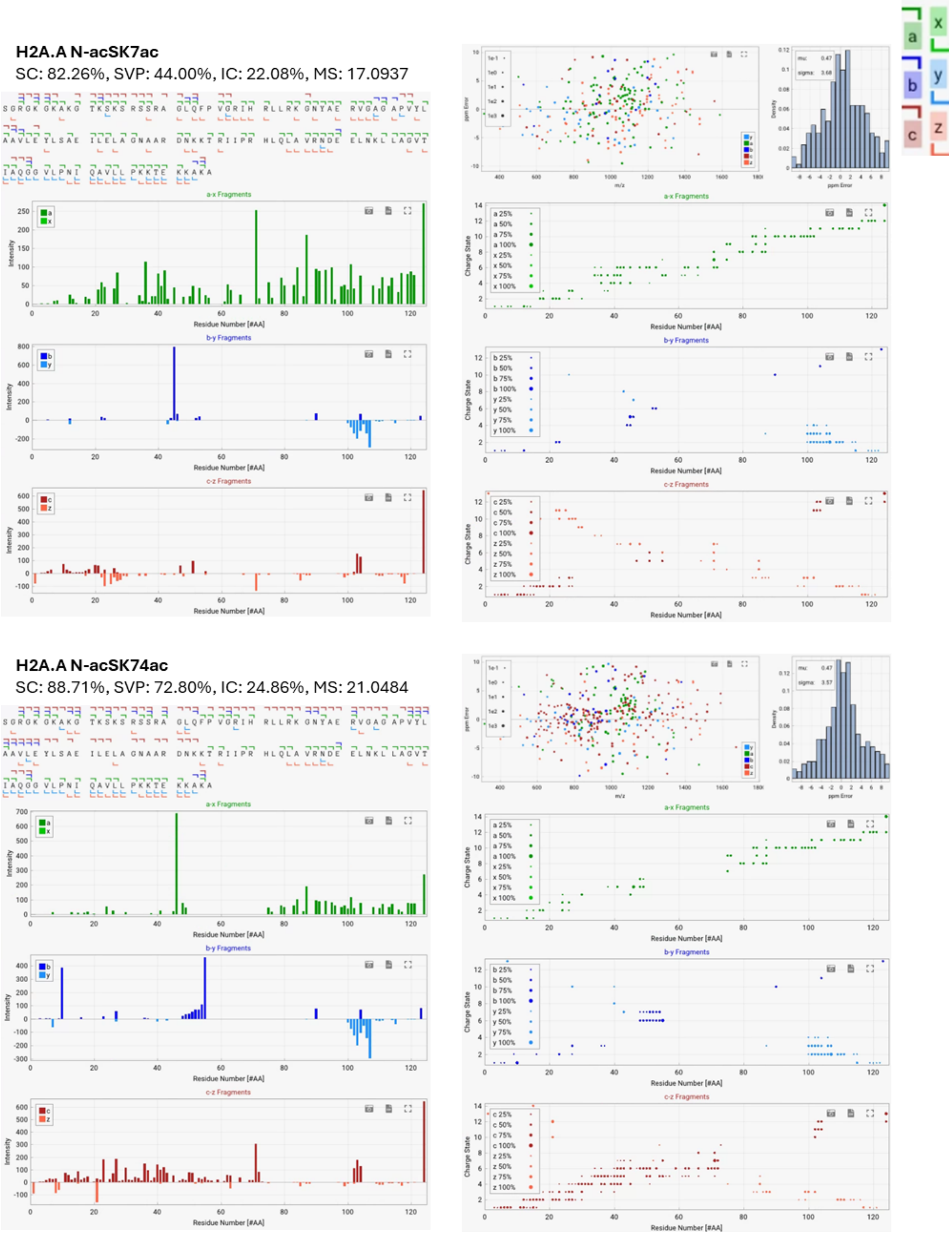

Figure S40. Top-down proteoform confirmation of *A. cervicornis* H2A.A variant PTM patterns with ±10 ppm error: N-acSK7ac and N-acSK74ac.

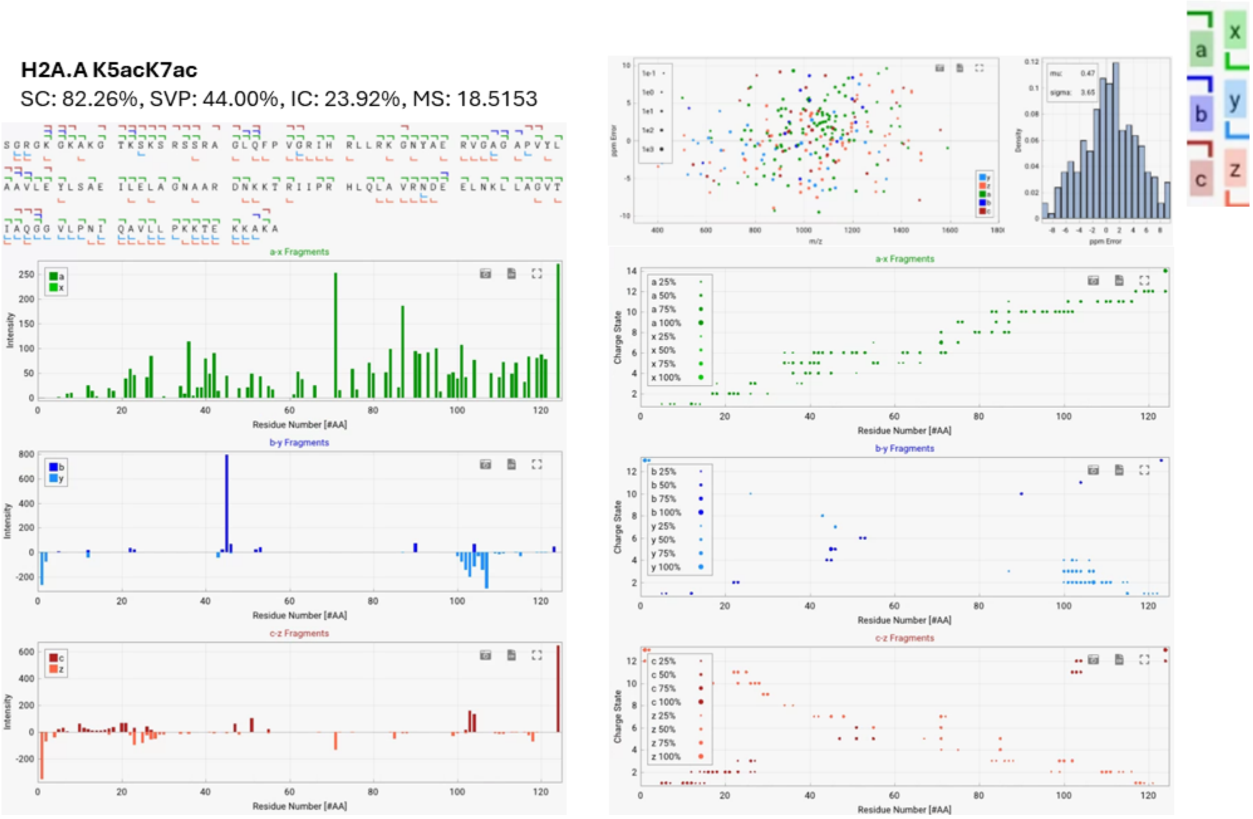

Figure S41. Top-down proteoform confirmation of *A. cervicornis* H2A.A variant PTM patterns with ±10 ppm error: K5acK7ac.

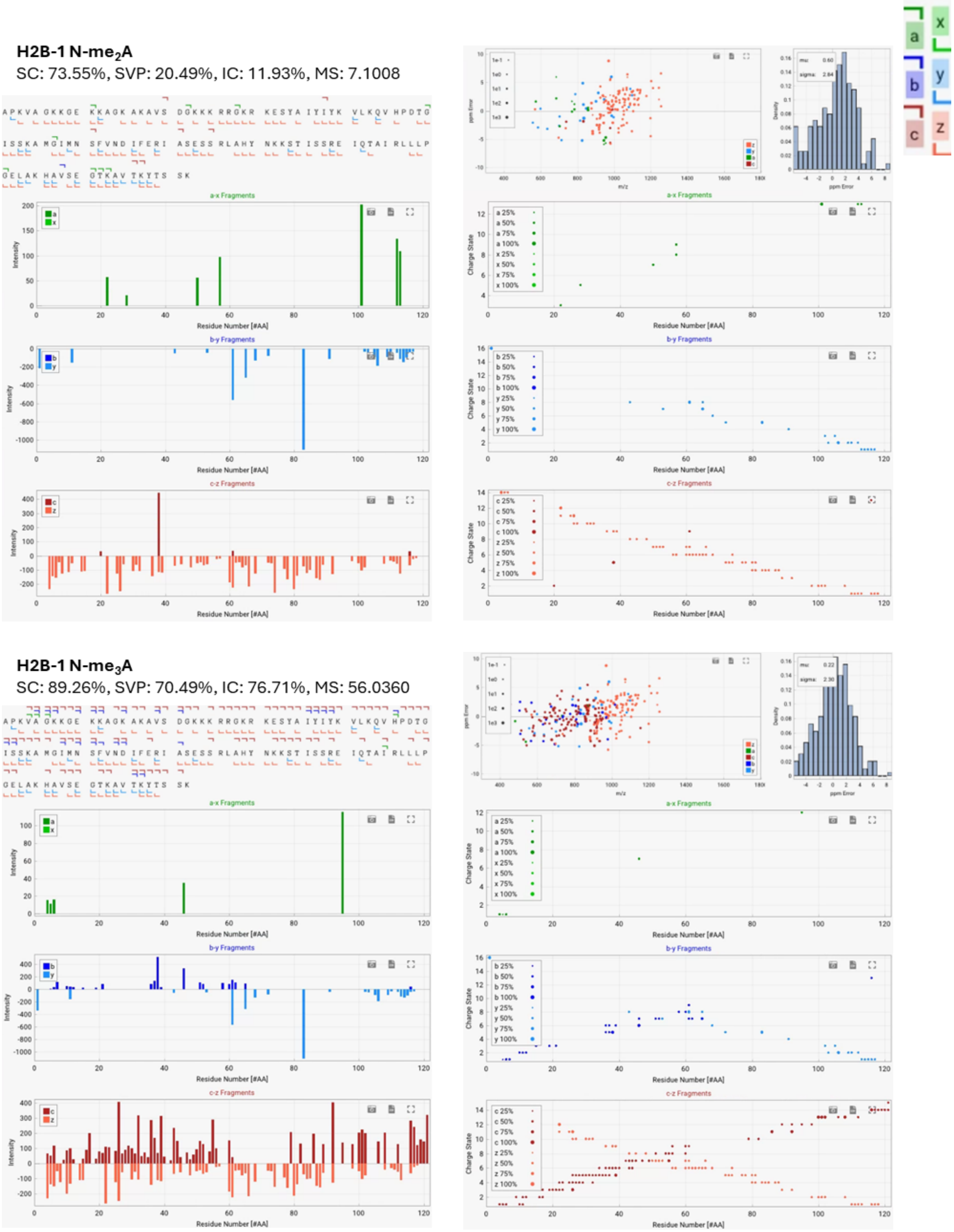

Figure S42. Top-down proteoform confirmation of *A. cervicornis* H2B-1 variant PTM patterns with ±10 ppm error: N-me$_2$A and N-me$_3$A.

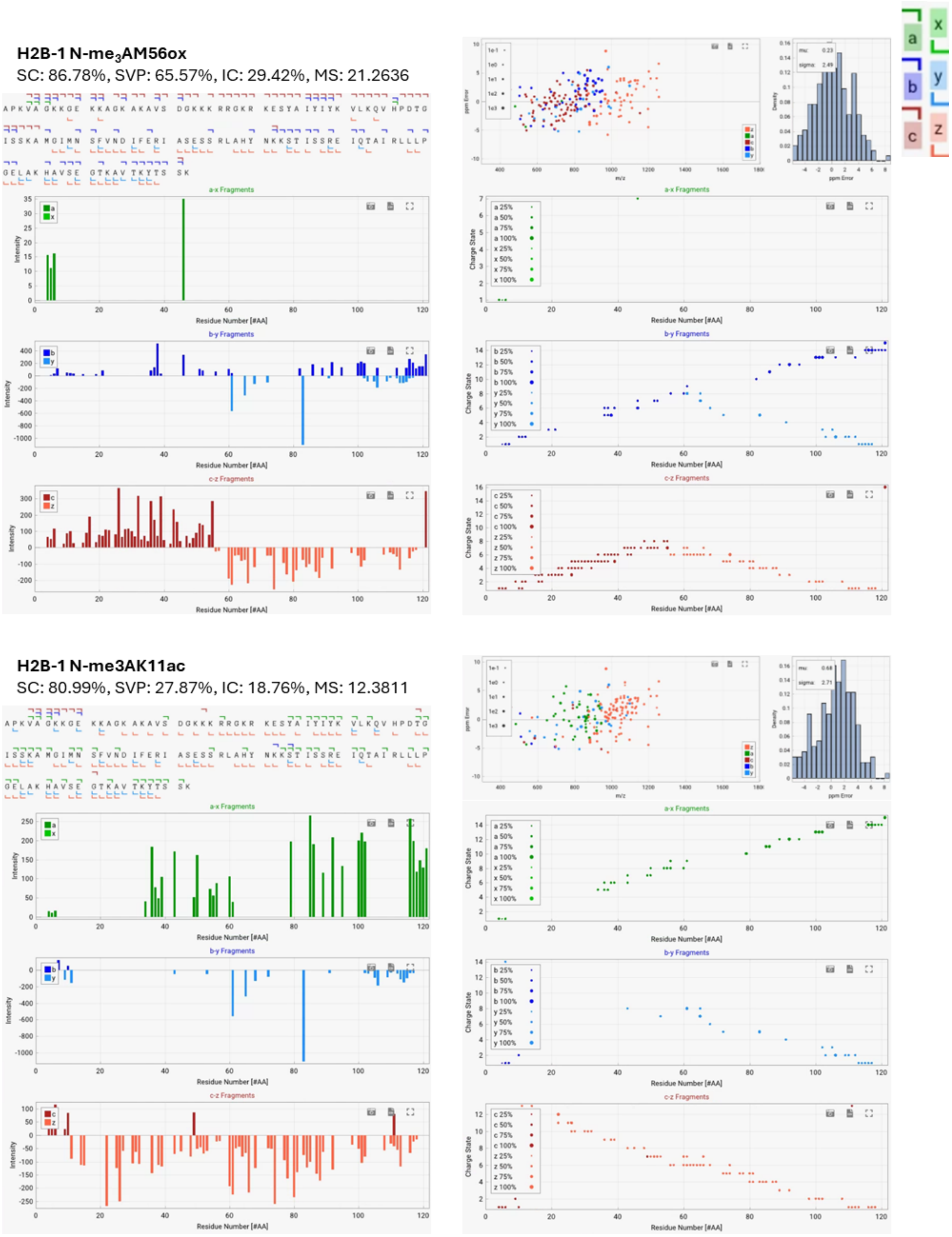

Figure S43. Top-down proteoform confirmation of *A. cervicornis* H2B-1 variant PTM patterns with ±10 ppm error: N-me$_3$AM56ox and N-me$_3$AK11ac.

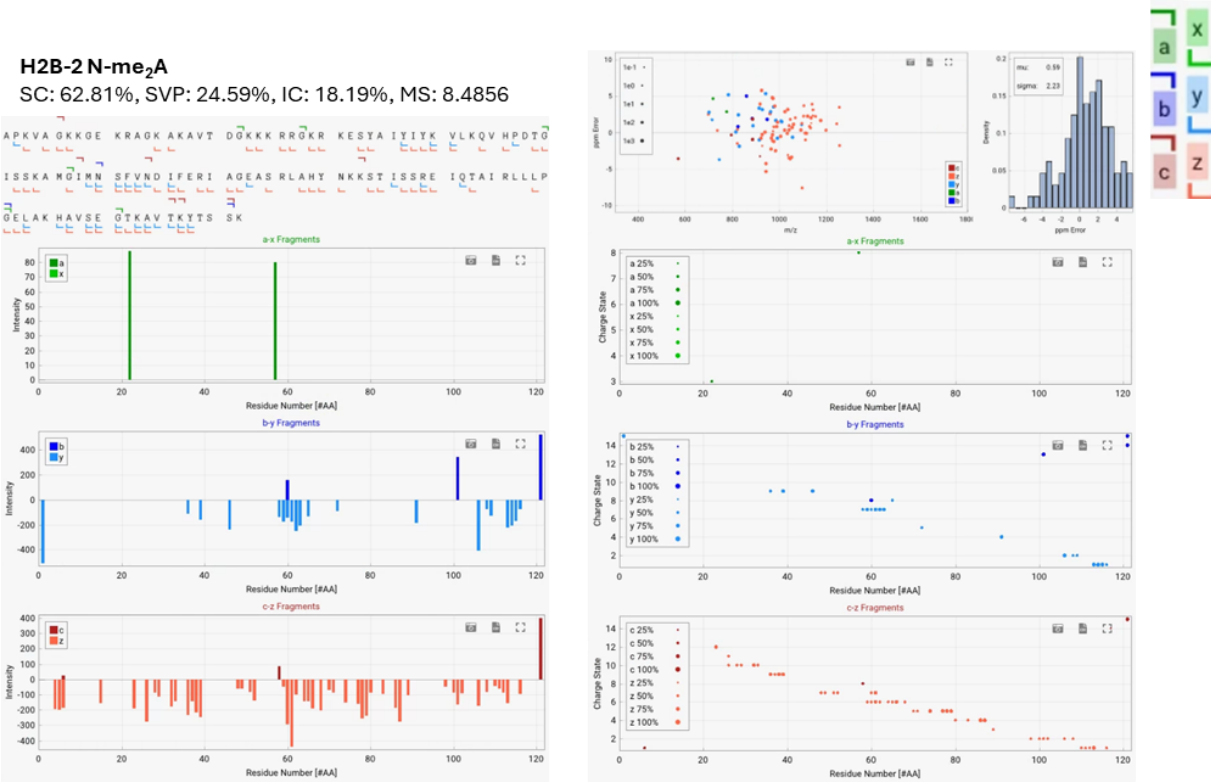

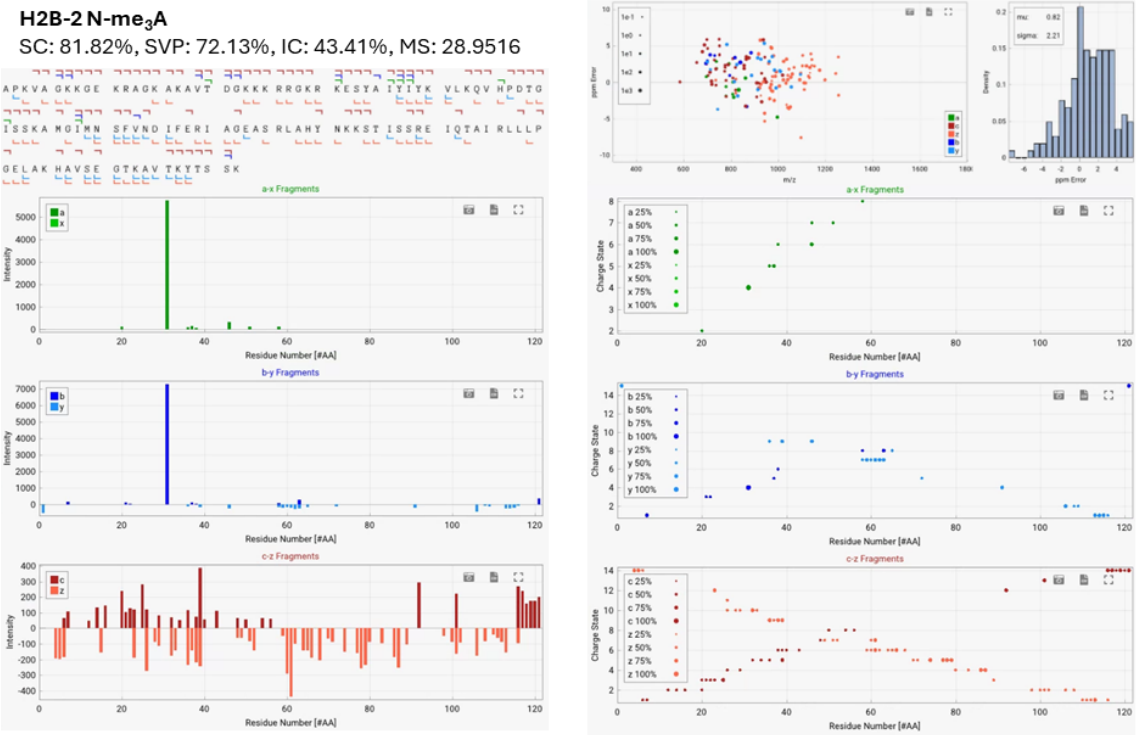

Figure S44. Top-down proteoform confirmation of *A. cervicornis* H2B-2 variant PTM patterns with ±10 ppm error: N-me$_2$A and N-me$_3$A.

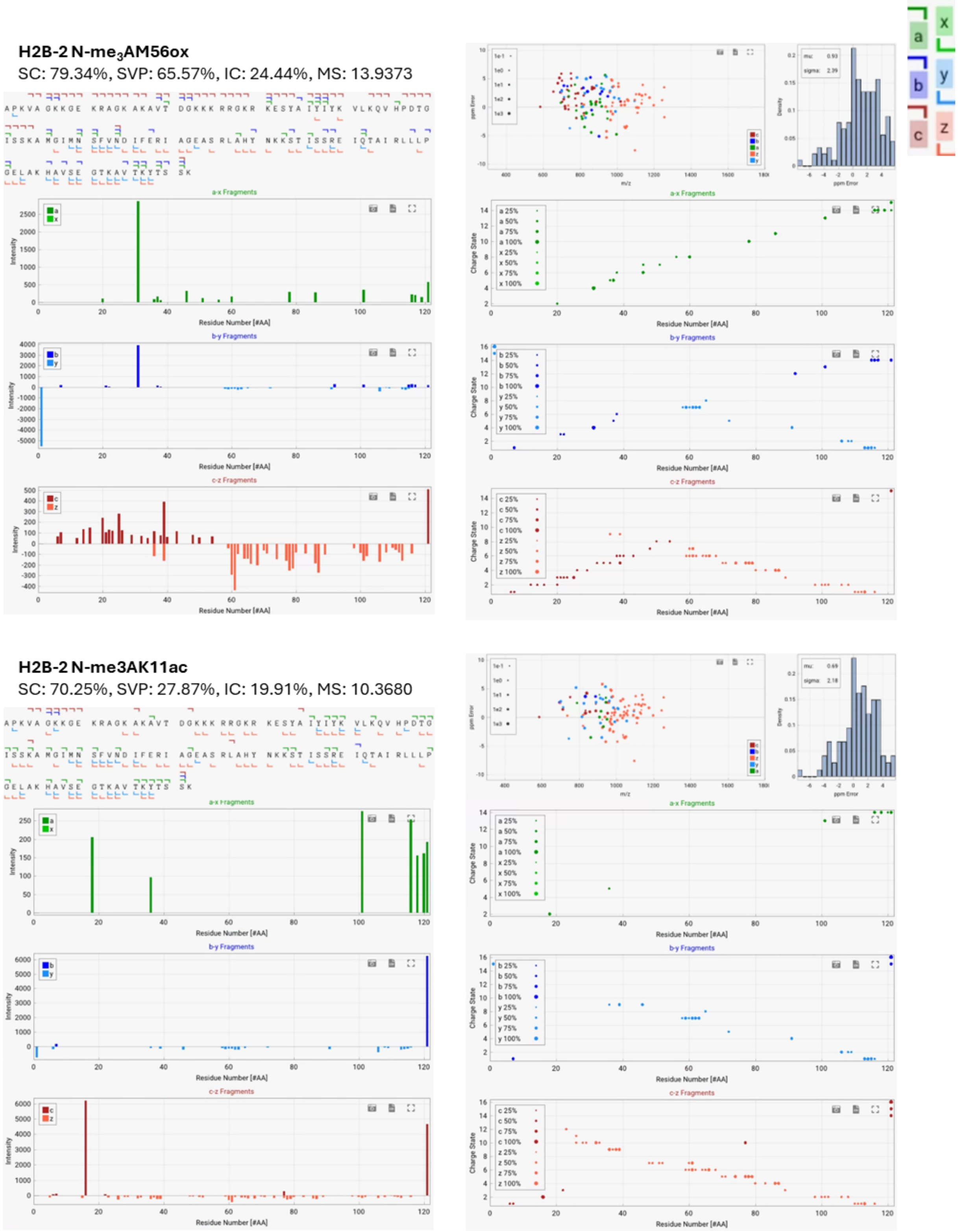

Figure S45. Top-down proteoform confirmation of *A. cervicornis* H2B-2 variant PTM patterns with ±10 ppm error: N-me$_3$AM56ox and N-me$_3$AK11ac.

H2B-2K N-me$_2$A

SC: 59.50%, SVP: 25.59%, IC: 8.21%, MS: 3.8512

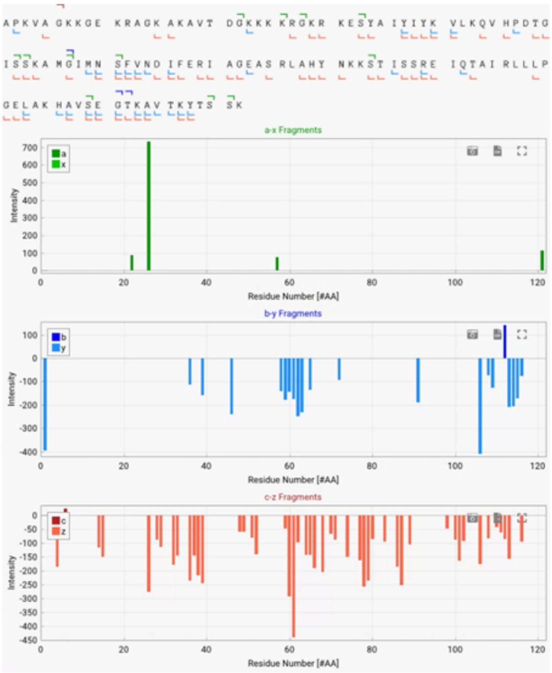

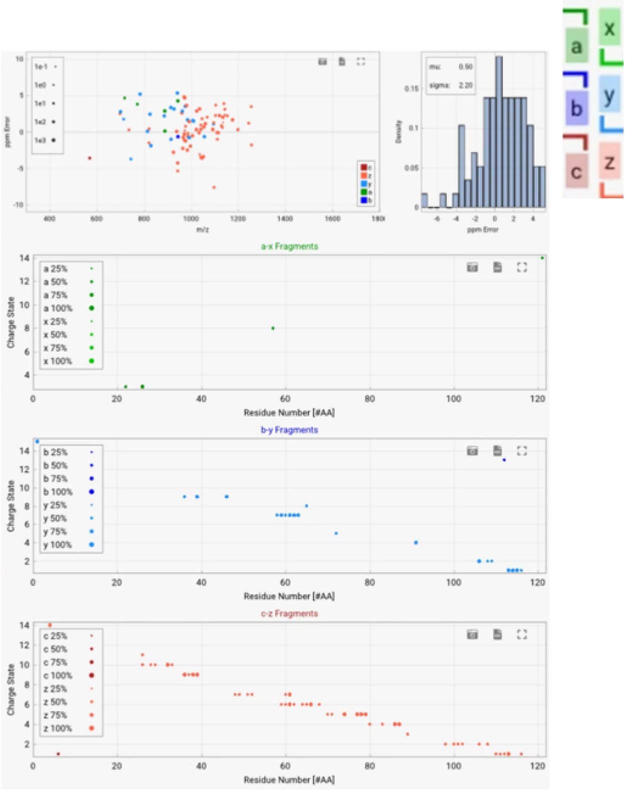

H2B-2K N-me$_3$A

SC: 78.51%, SVP: 43.44%, IC: 31.19%, MS: 19.3305

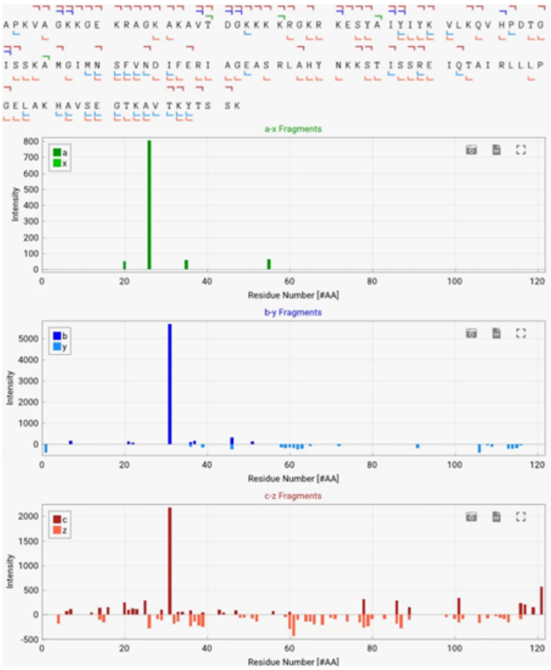

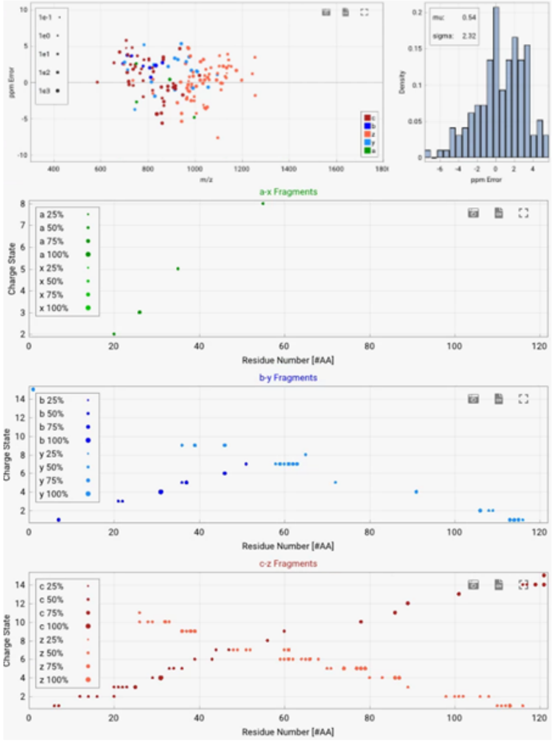

Figure S46. Top-down proteoform confirmation of *A. cervicornis* H2B-2K variant PTM patterns with ±10 ppm error: N-me$_2$A and N-me$_3$A.

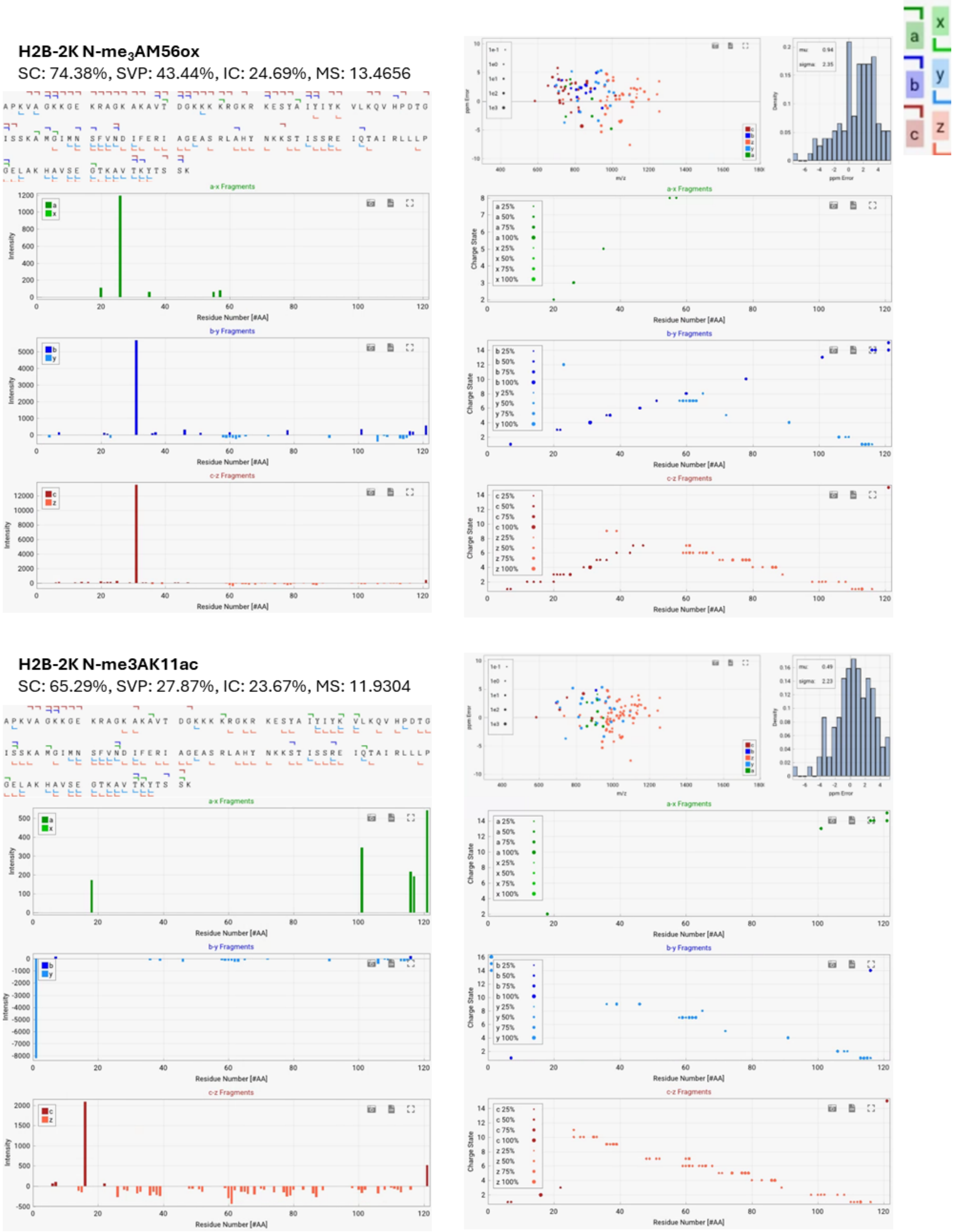

Figure S47. Top-down proteoform confirmation of *A. cervicornis* H2B-2K variant PTM patterns with ±10 ppm error: N-me$_3$AM56ox and N-me$_3$AK11ac.

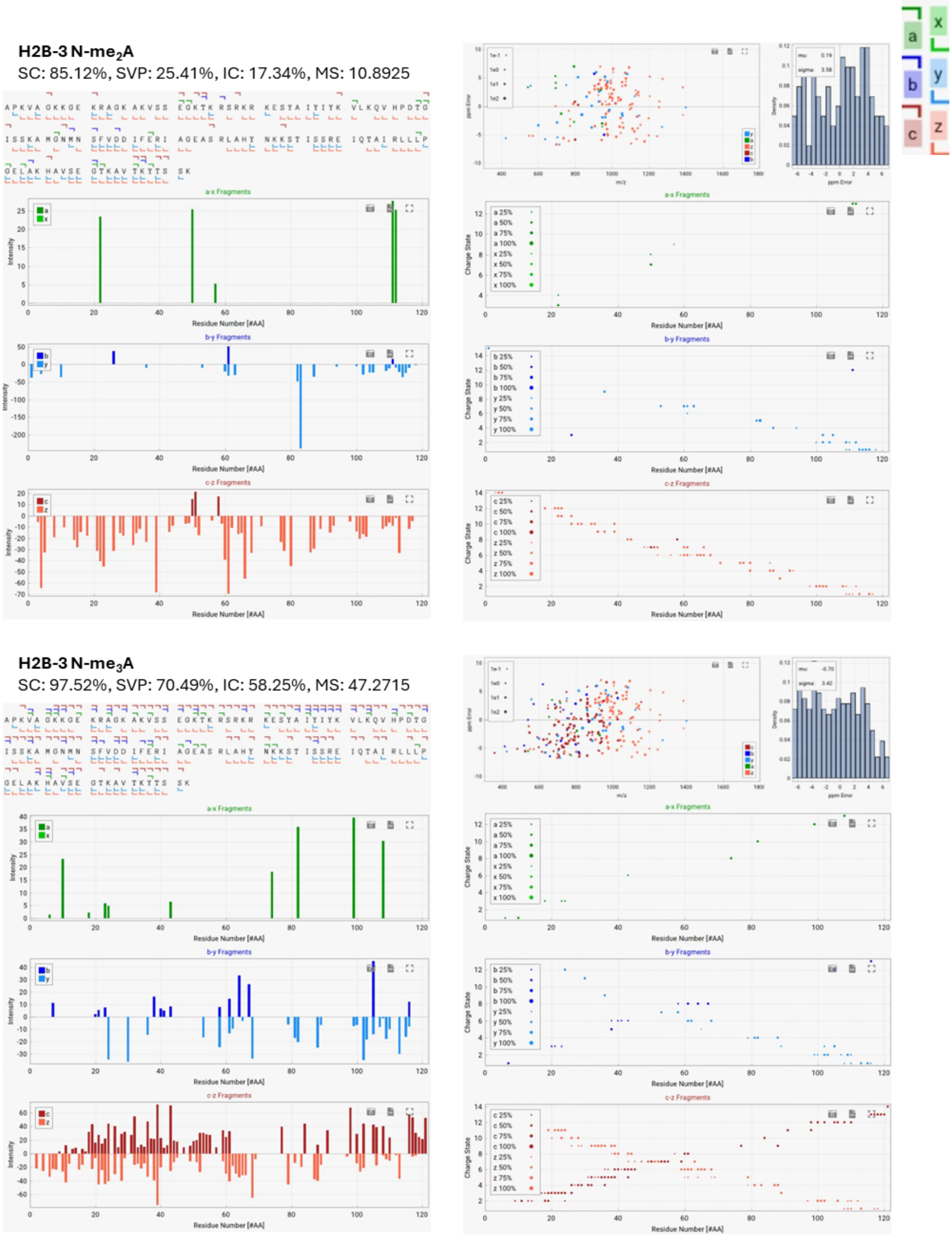

Figure S48. Top-down proteoform confirmation of *A. cervicornis* H2B-3 variant PTM patterns with ±10 ppm error: N-me$_2$A and N-me$_3$A.

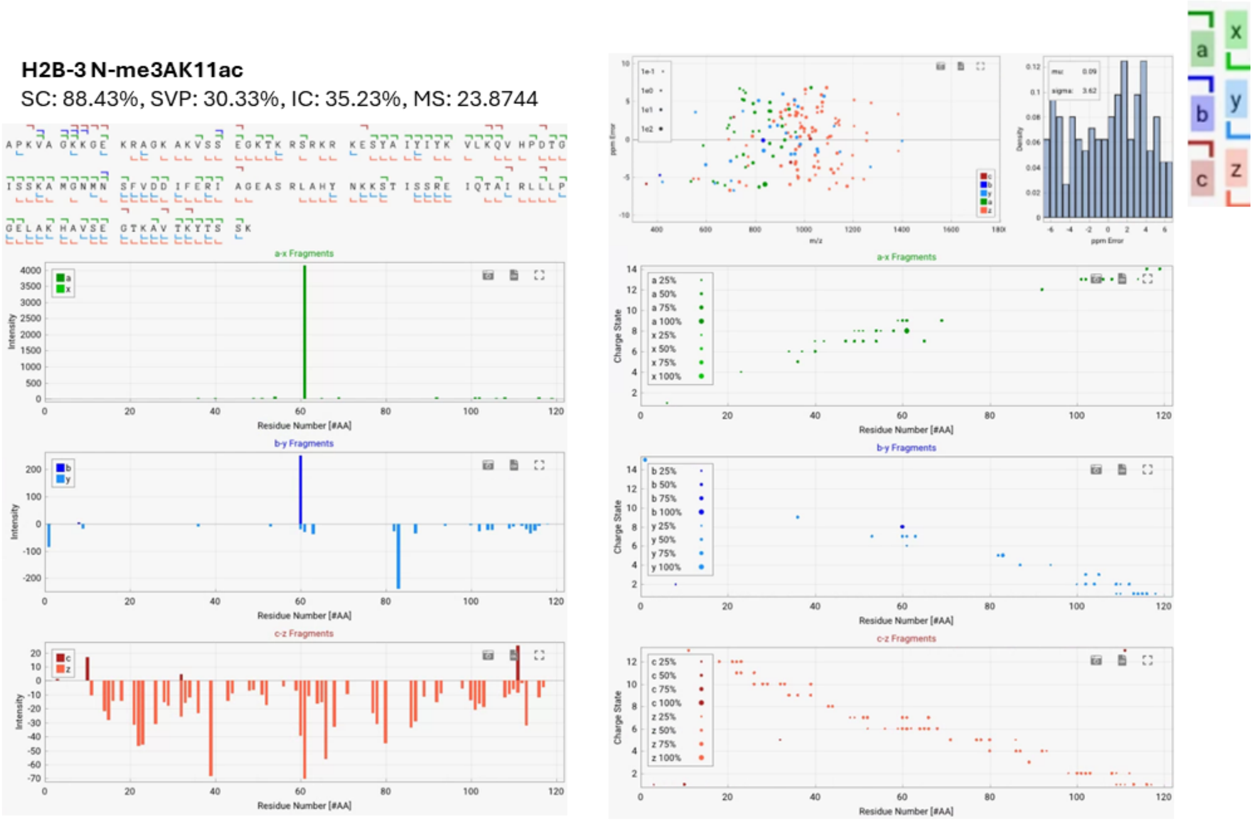

Figure S49. Top-down proteoform confirmation of *A. cervicornis* H2B-3 variant PTM patterns with ±10 ppm error: N-me$_3$AK11ac.

Figure S50. Ion annotation of MS/MS spectrum for H4 N-acSK20me2 $[M+12H]^{12+}$.

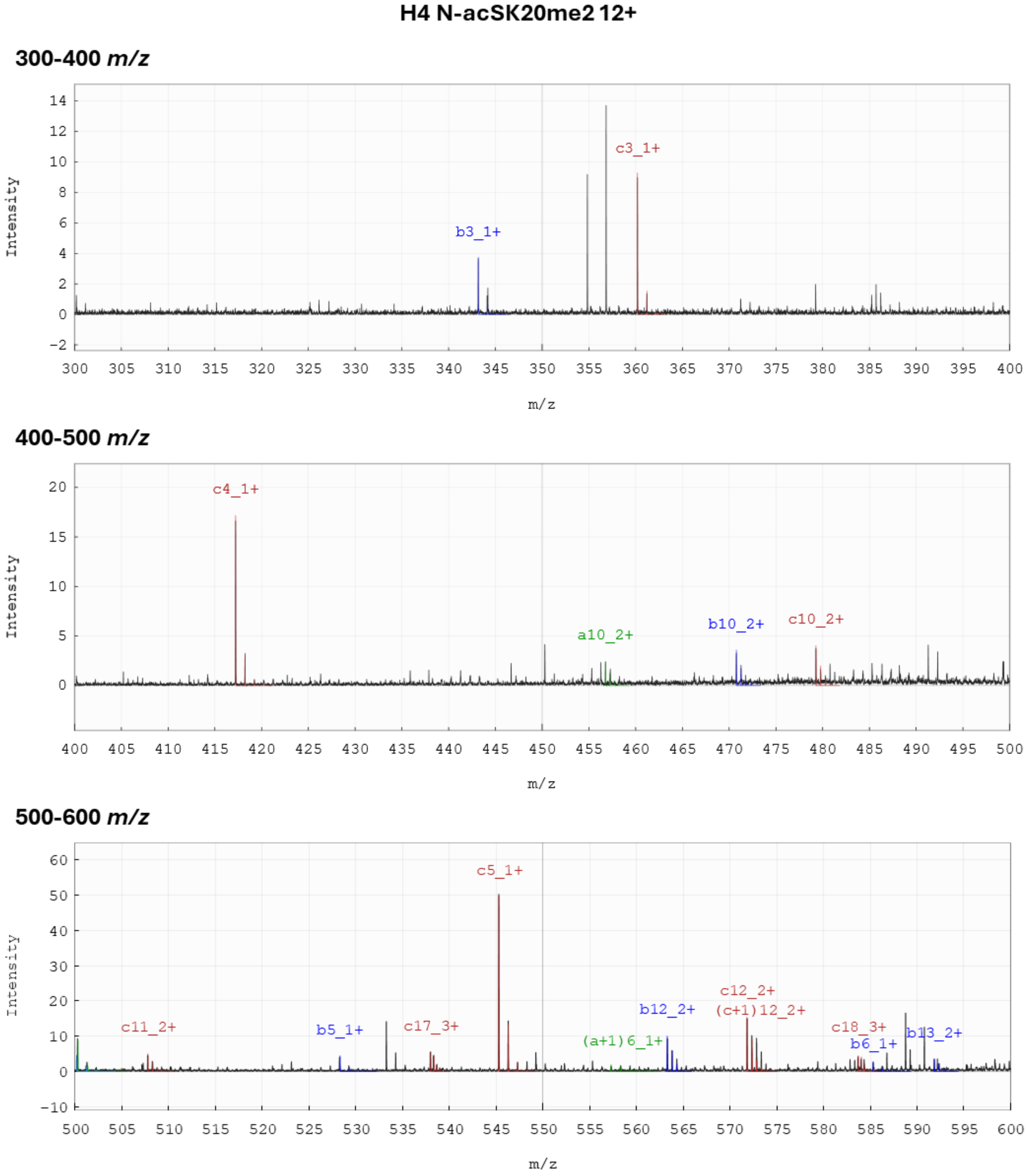

H4 N-acSK20me2 12+

600-700 *m/z*

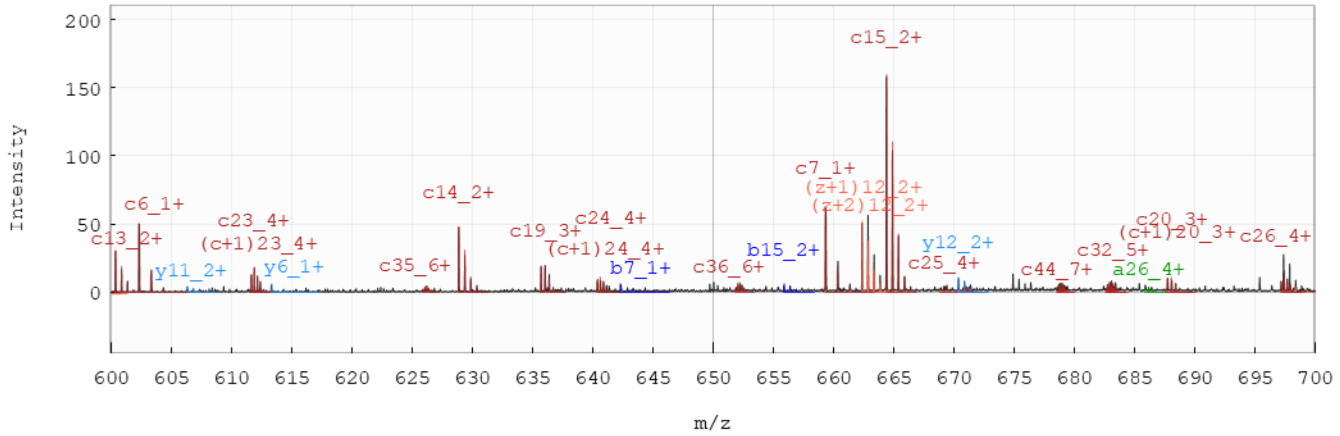

700-800 *m/z*

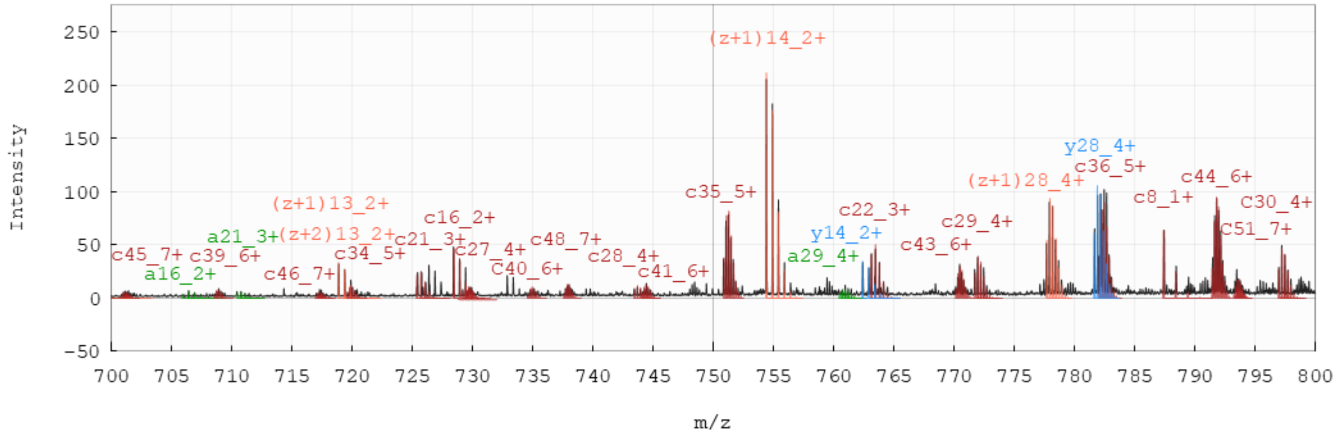

800-900 *m/z*

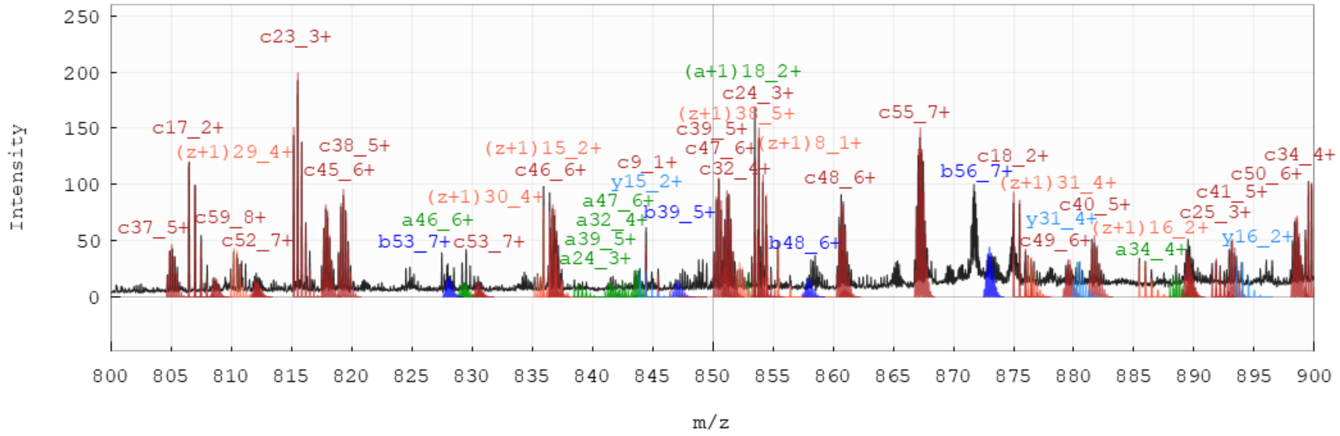

H4 N-acSK20me2 12+

900-950 *m/z*

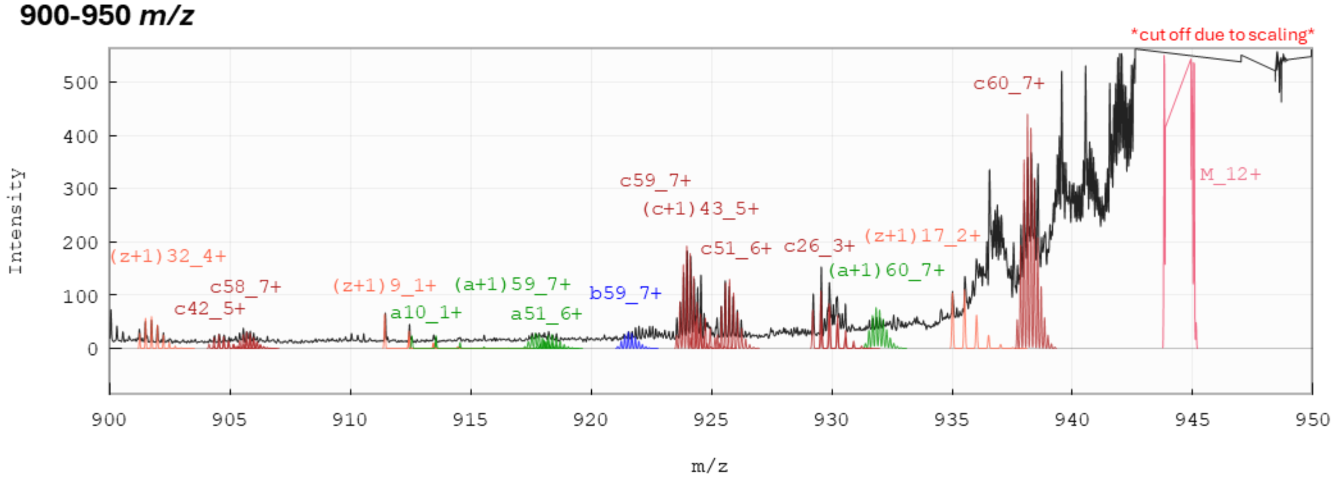

950-1000 *m/z*

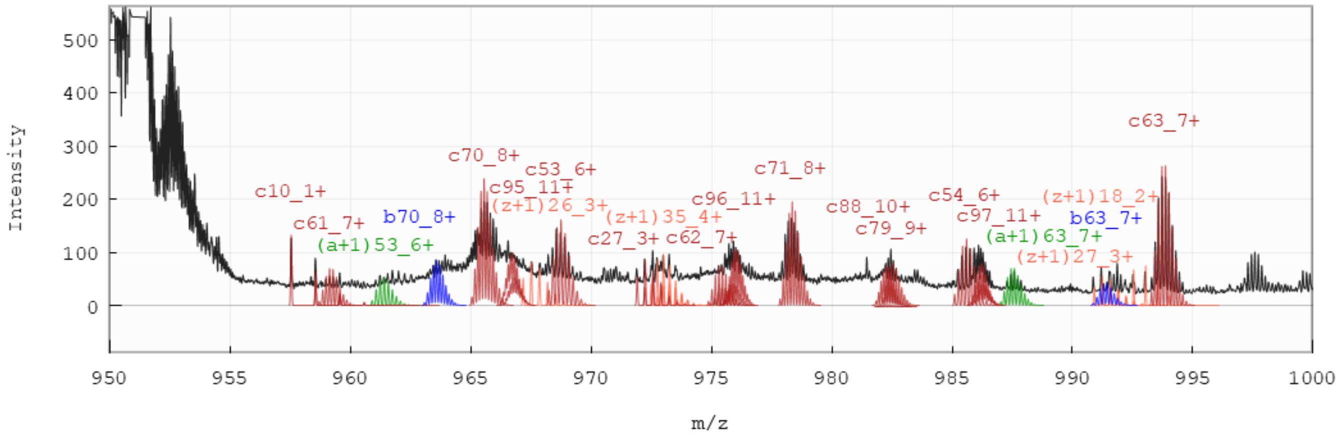

1000-1050 *m/z*

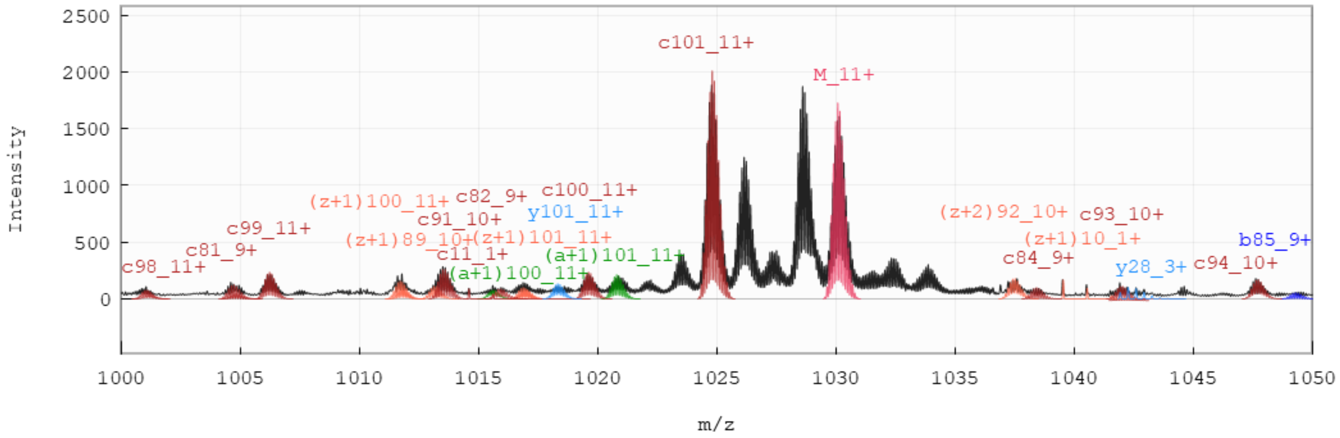

H4 N-acSK20me2 12+

1050-1100 *m/z*

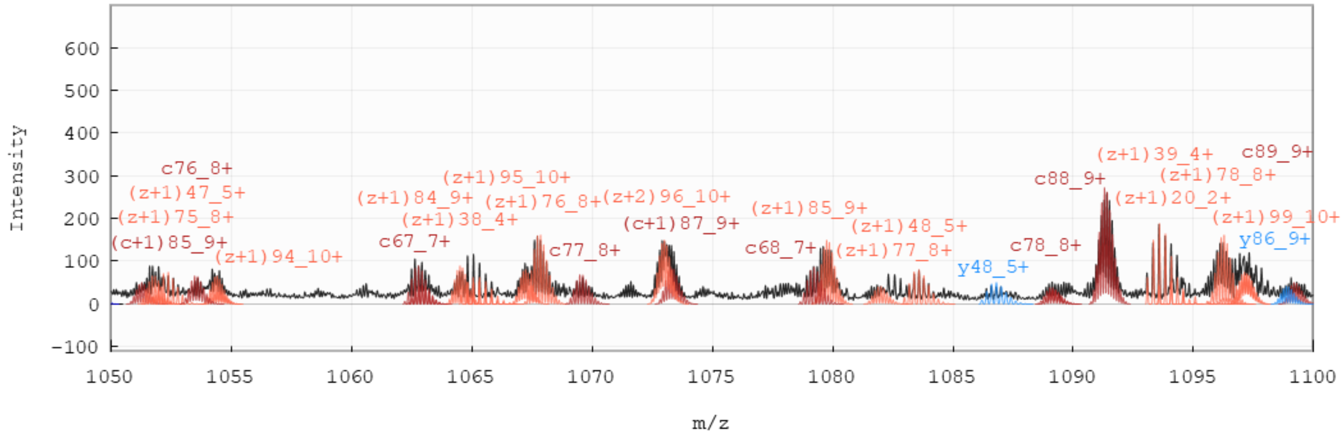

1100-1200 *m/z*

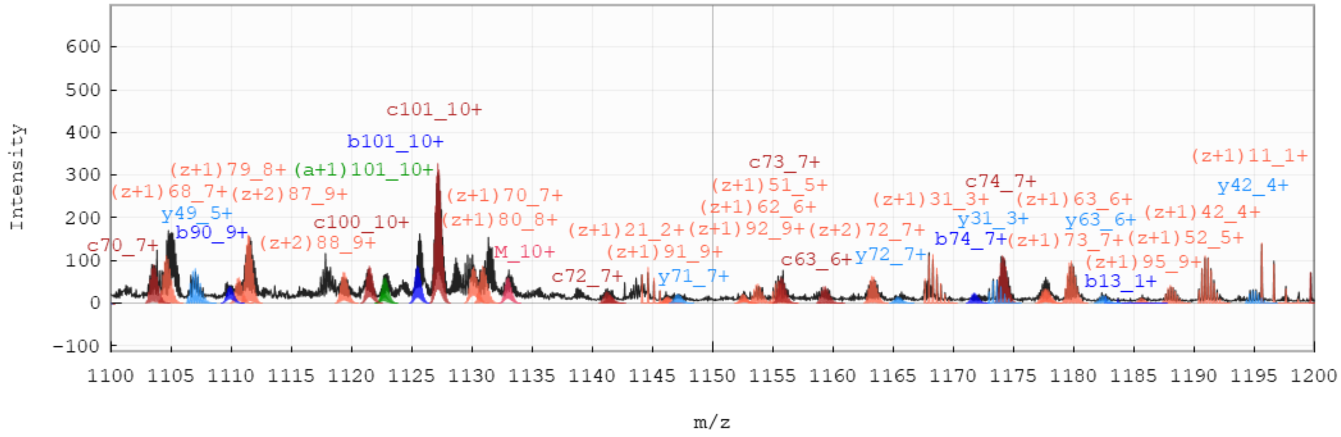

1200-1300 *m/z*

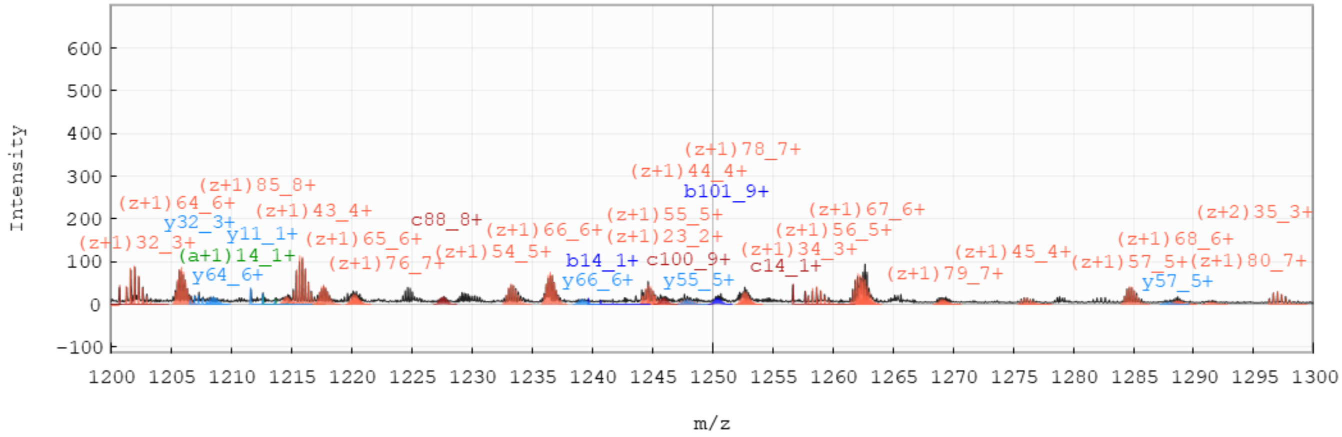

H4 N-acSK20me2 12+

1300-1400 *m/z*

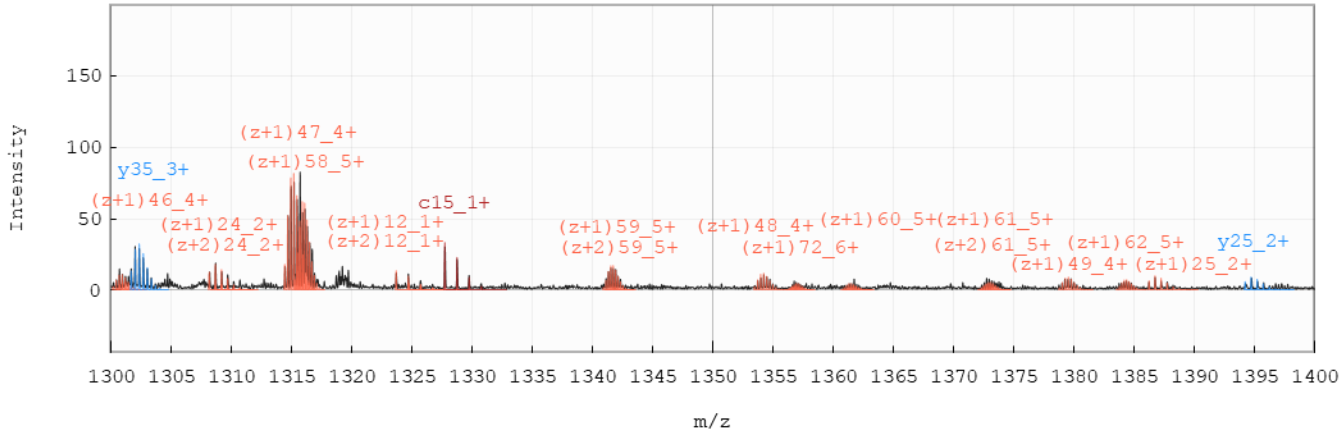

1400-1500 *m/z*

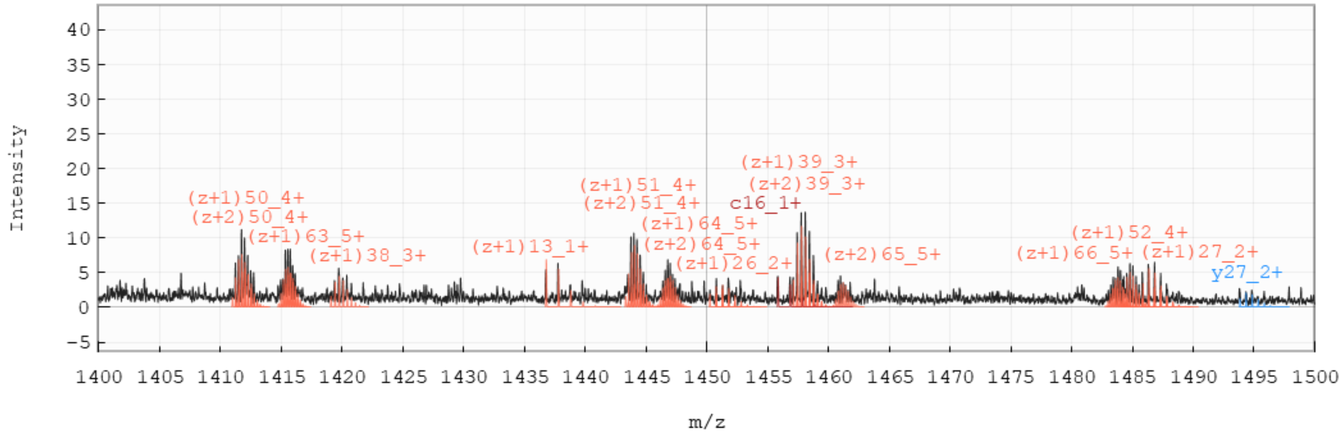

1500-1600 *m/z*

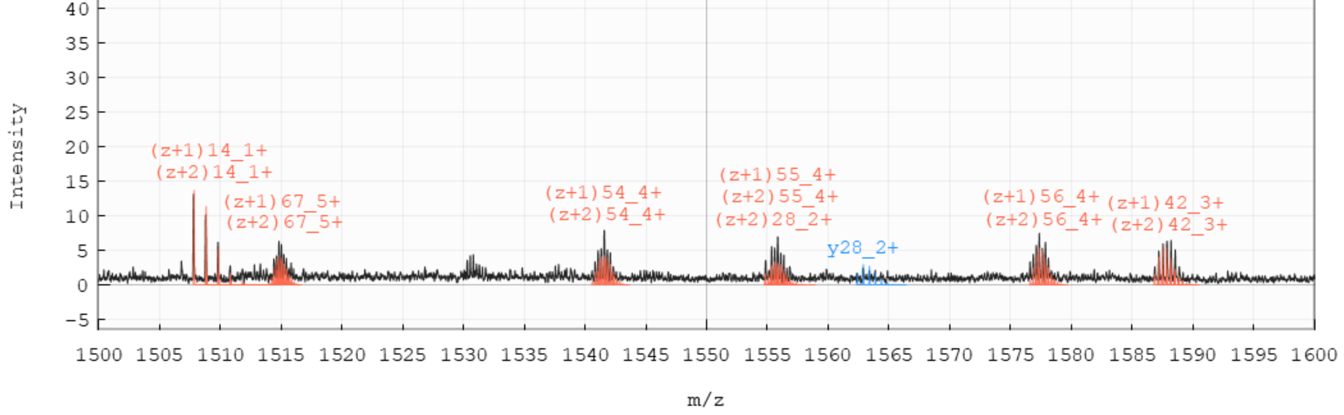

H4 N-acSK20me2 12+

1600-1800 *m/z*

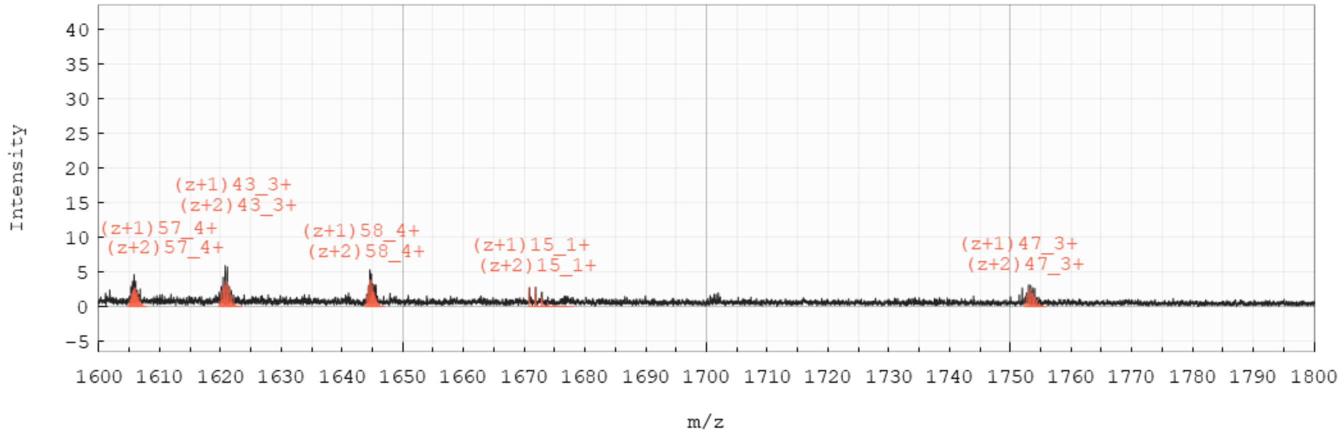

Figure S51. Ion annotation of MS/MS spectrum for H4.S N-acSK20me2 [M+13H]$^{13+}$.

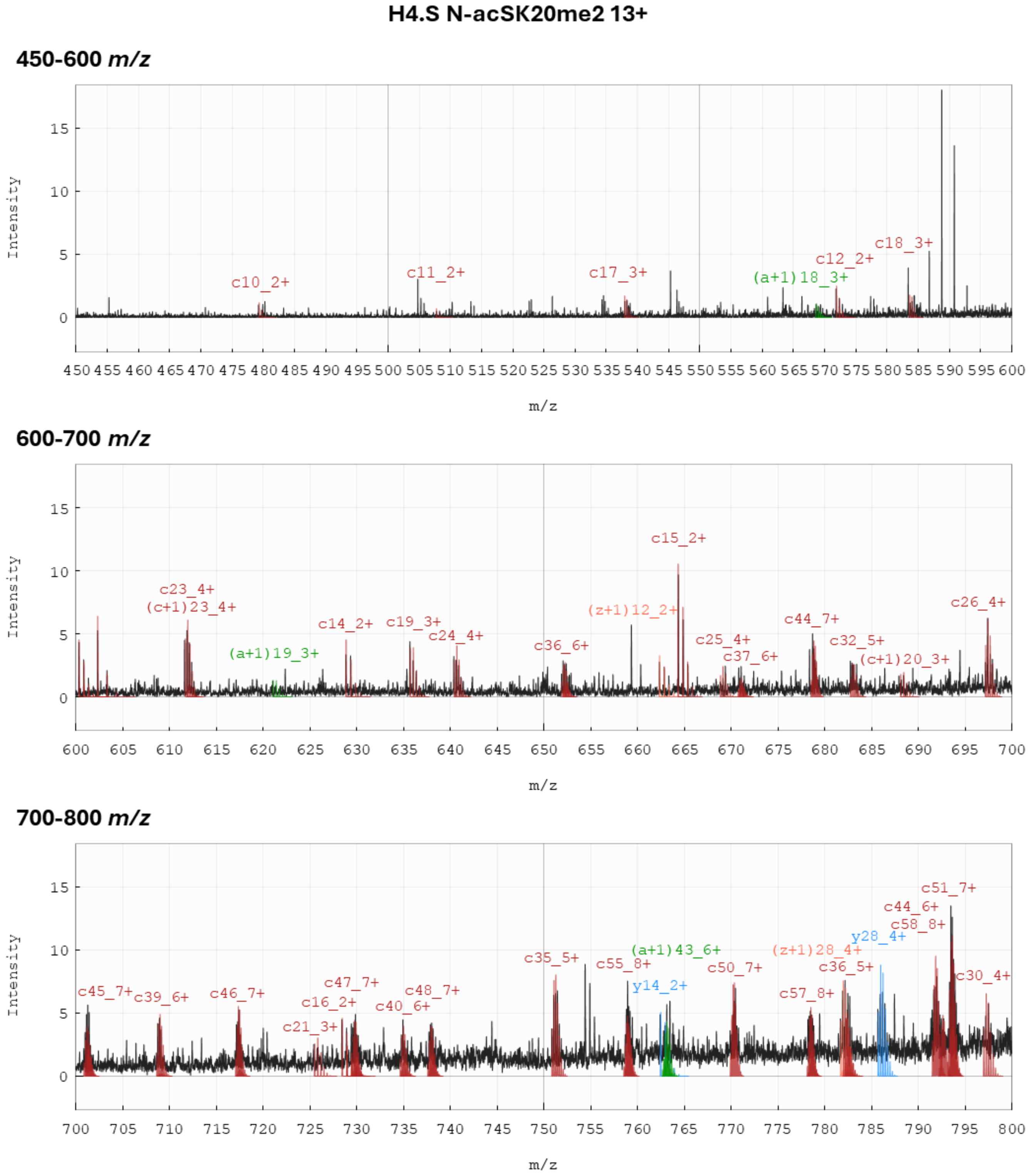

H4.S N-acSK20me2 13+

800-850 *m/z*

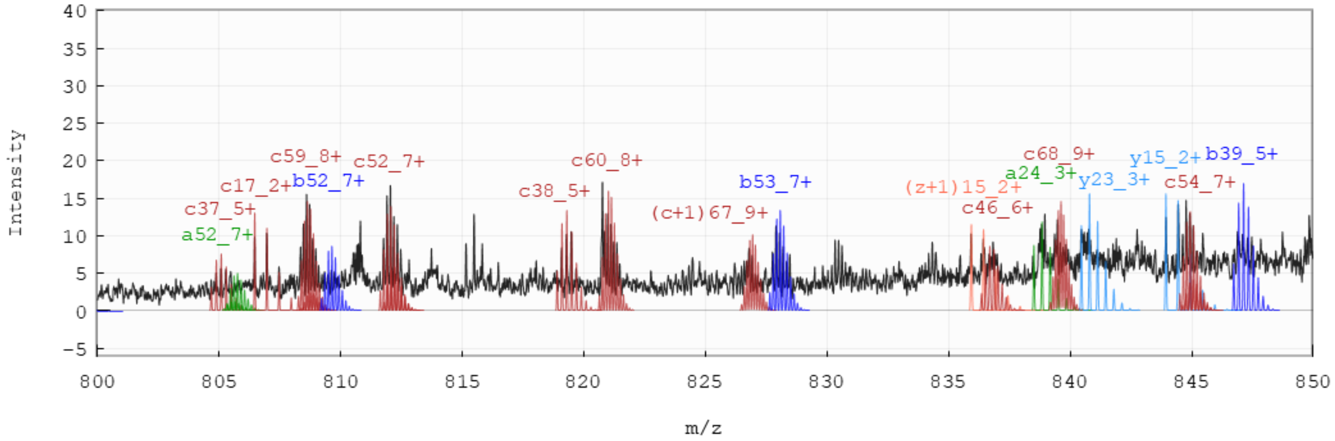

850-900 *m/z*

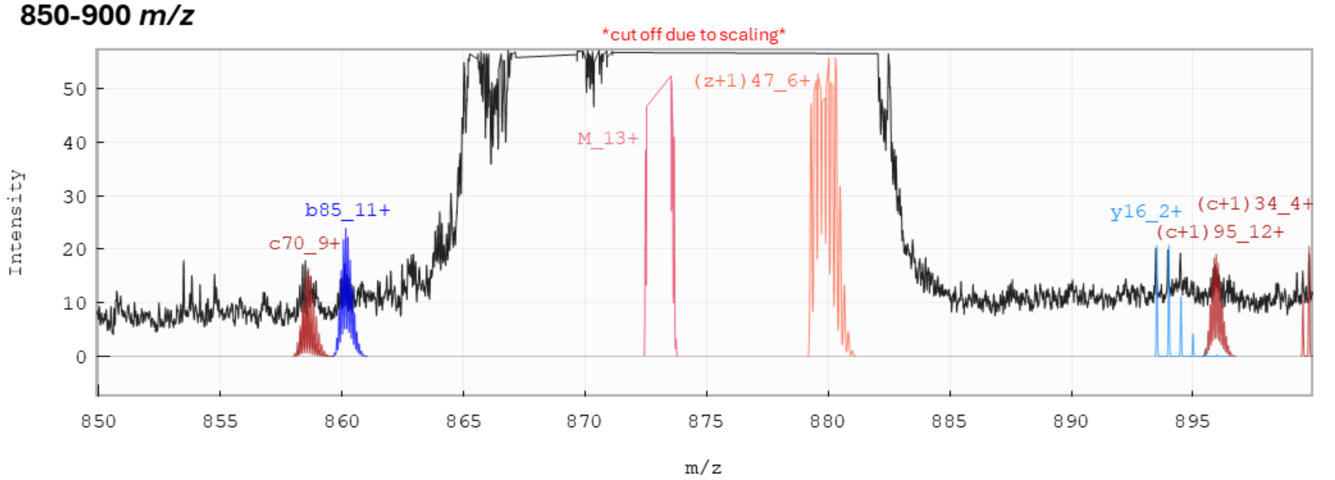

900-950 *m/z*

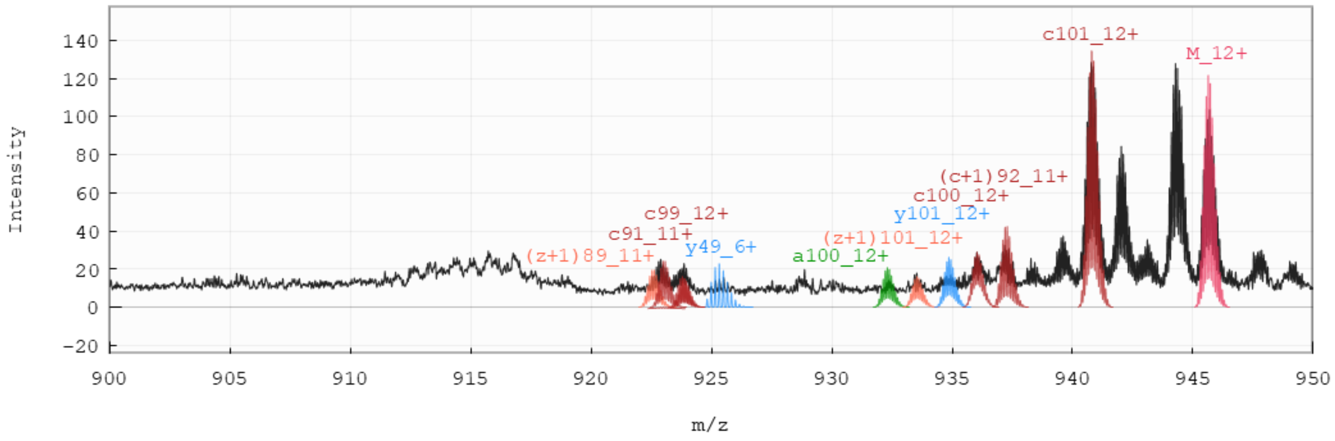

H4.S N-acSK20me2 13+

950-1000 *m/z*

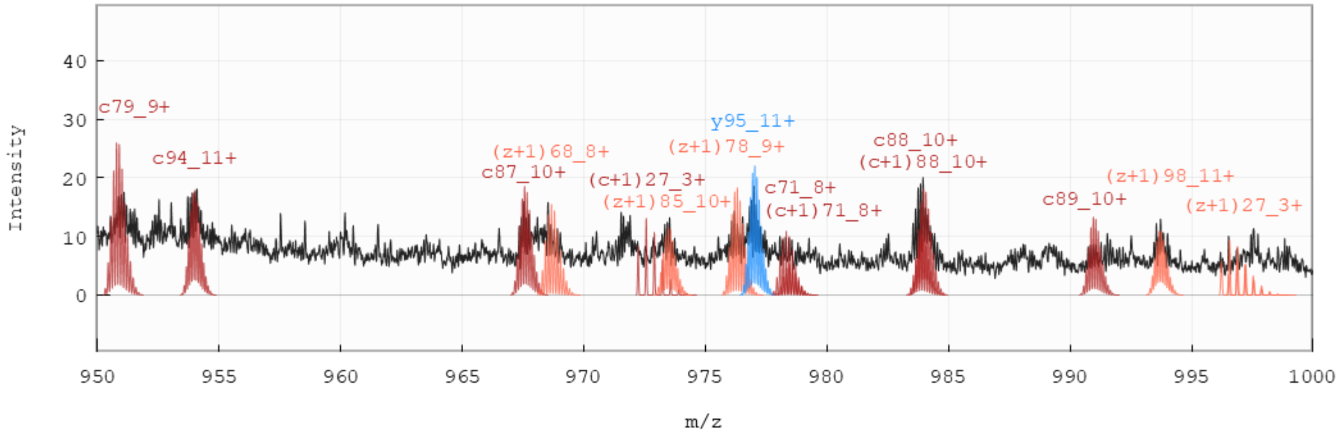

1000-1050 *m/z*

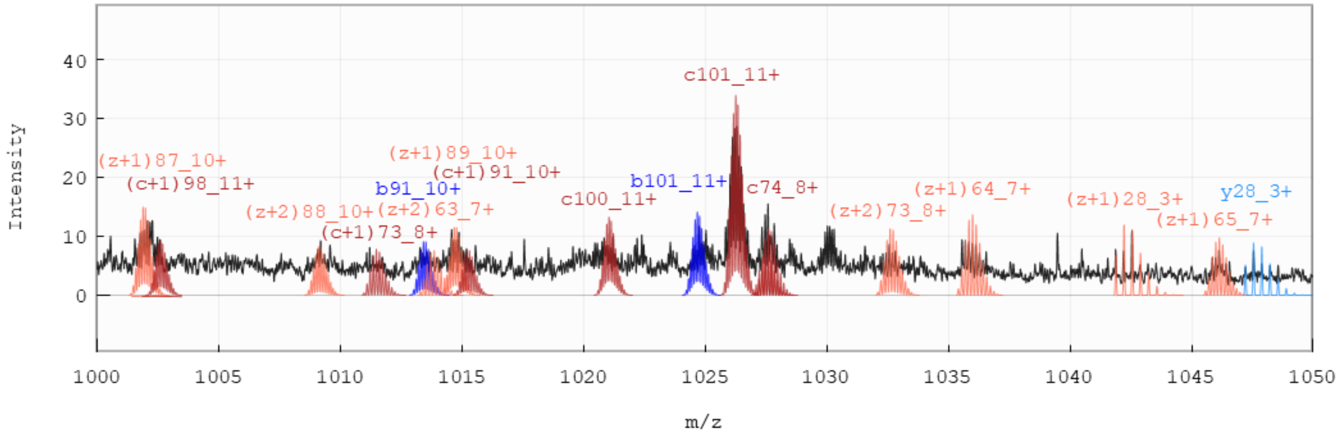

1050-1100 *m/z*

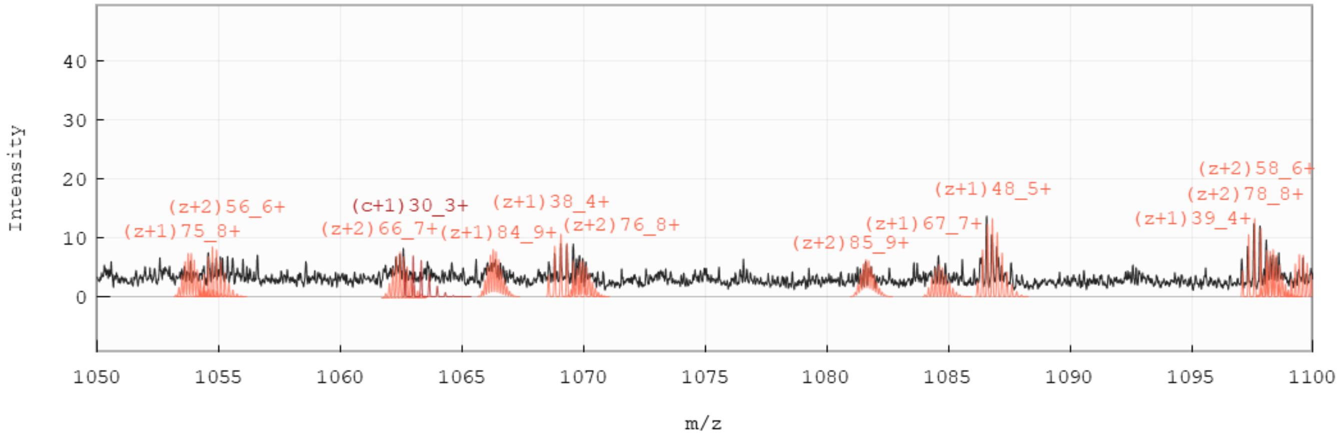

H4.S N-acSK20me2 13+

1100-1200 *m/z*

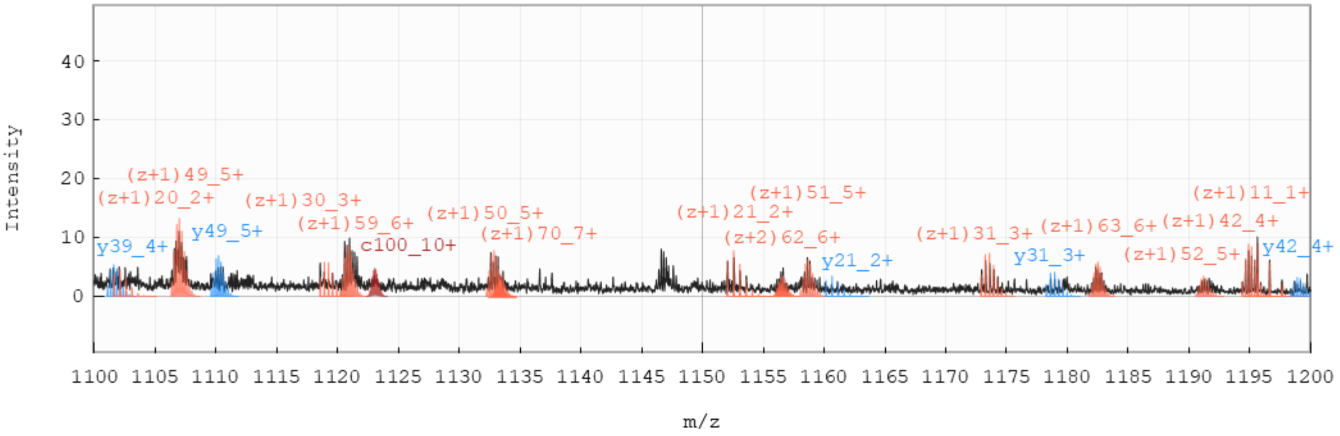

1200-1300 *m/z*

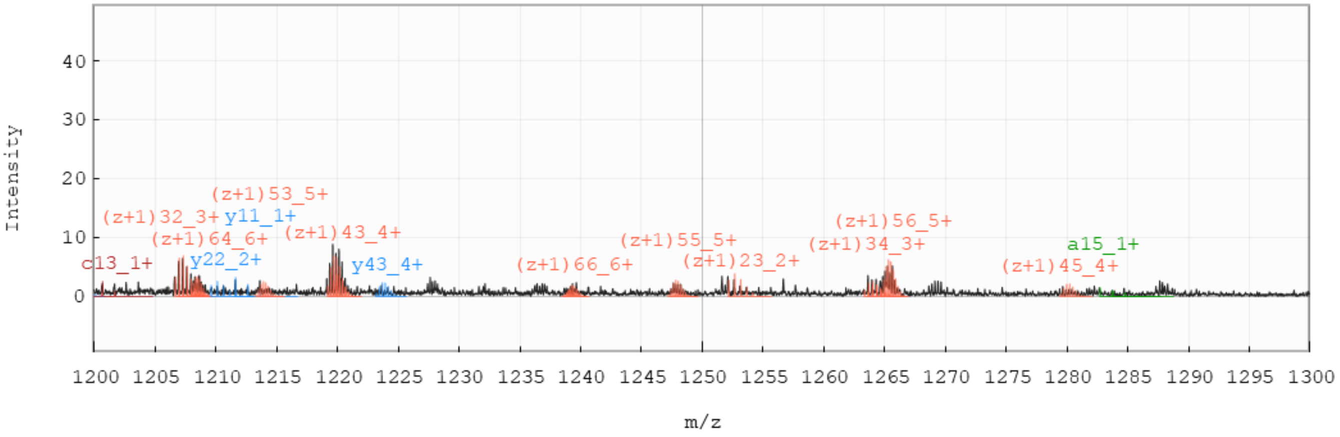

1300-1400 *m/z*

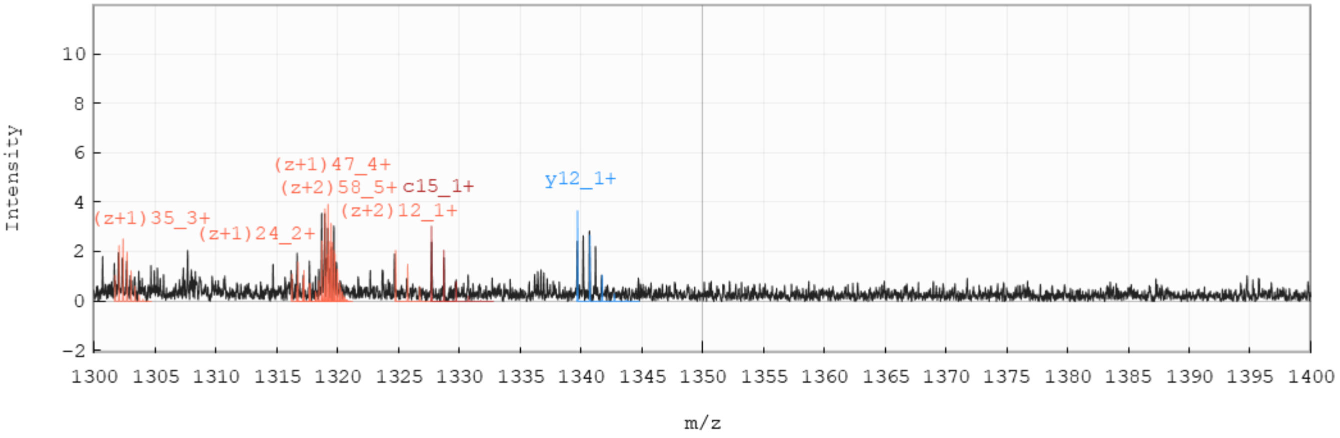

H4.S N-acSK20me2 13+

1500-1700 *m/z*

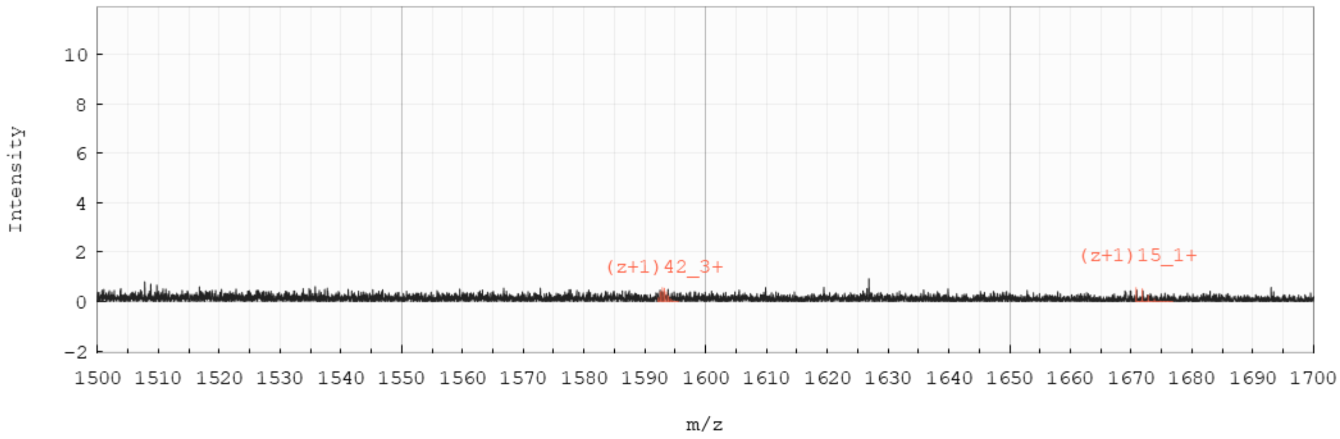

Figure S52. Ion annotation of MS/MS spectrum for H2A N-acS [M+14H]$^{14+}$.

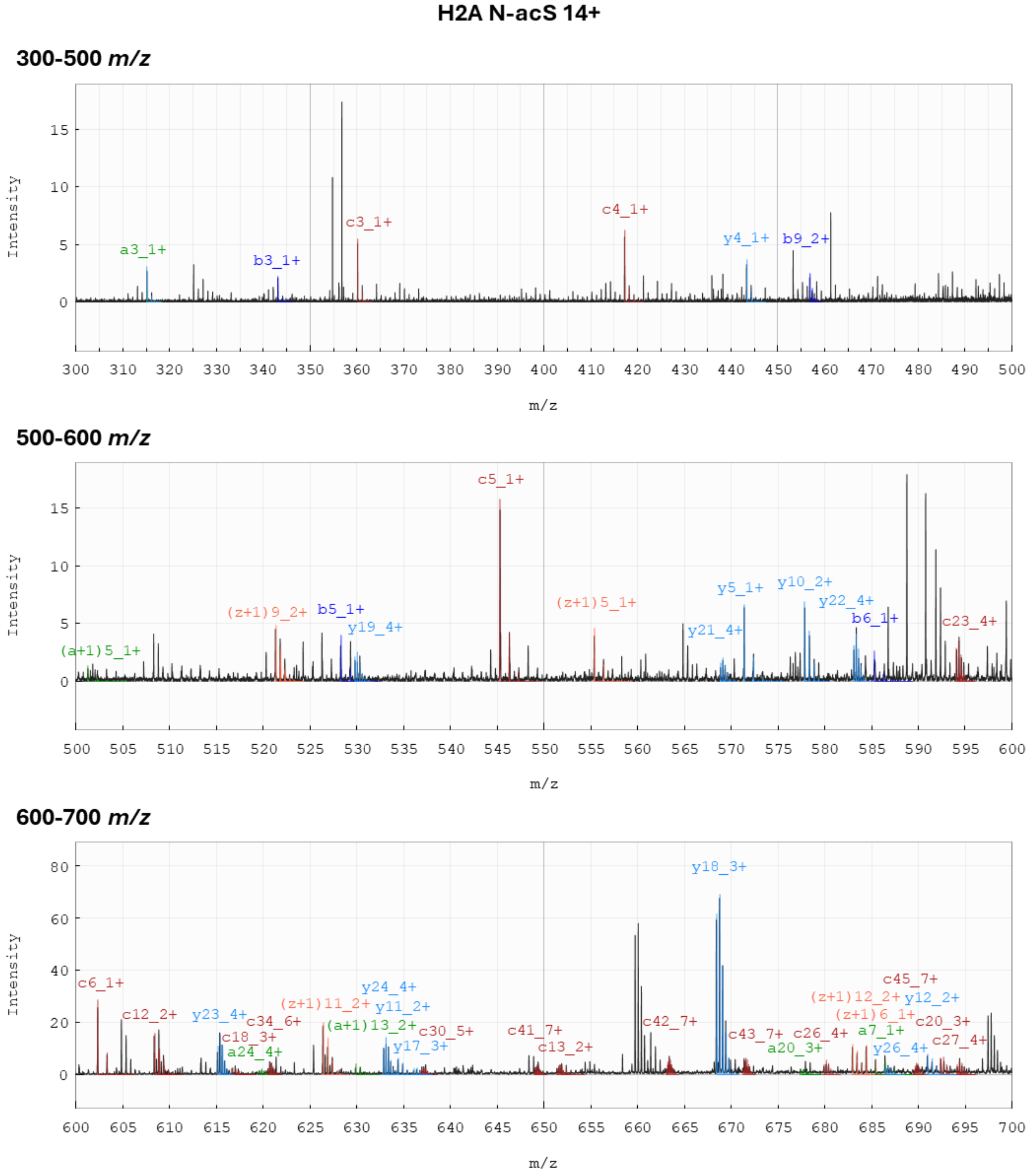

H2A N-acS 14+

700-800 *m/z*

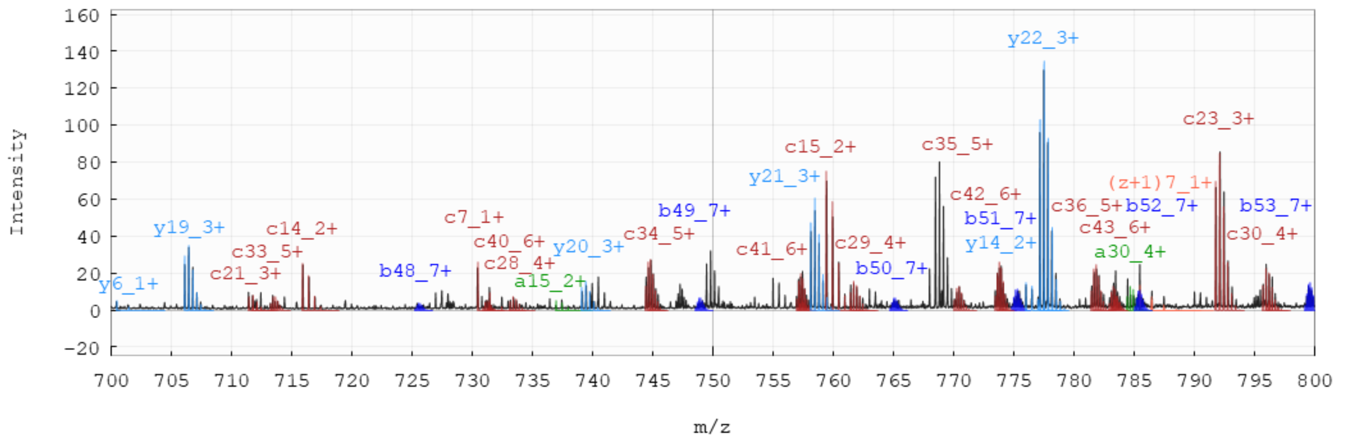

800-900 *m/z*

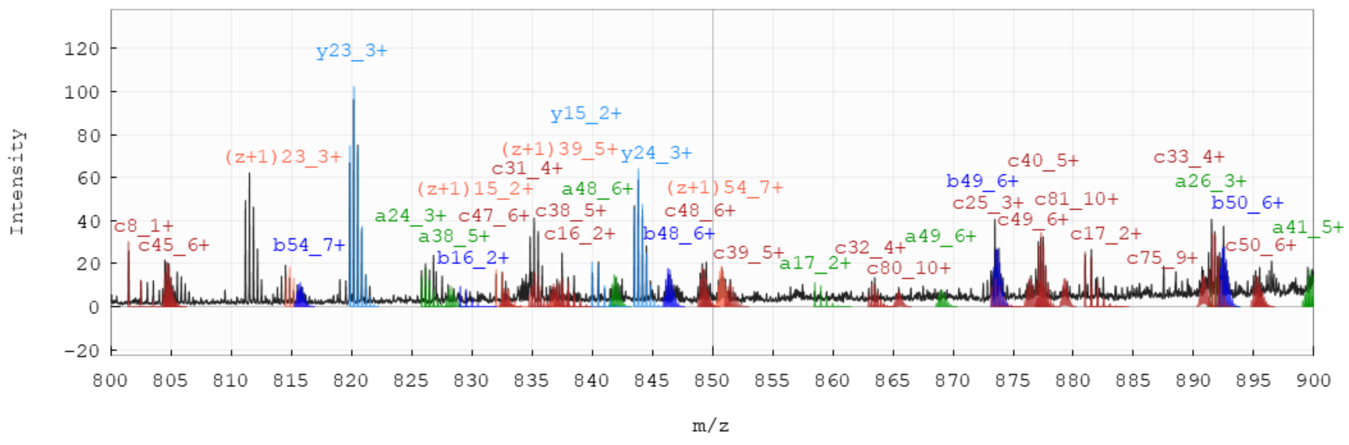

900-950 *m/z*

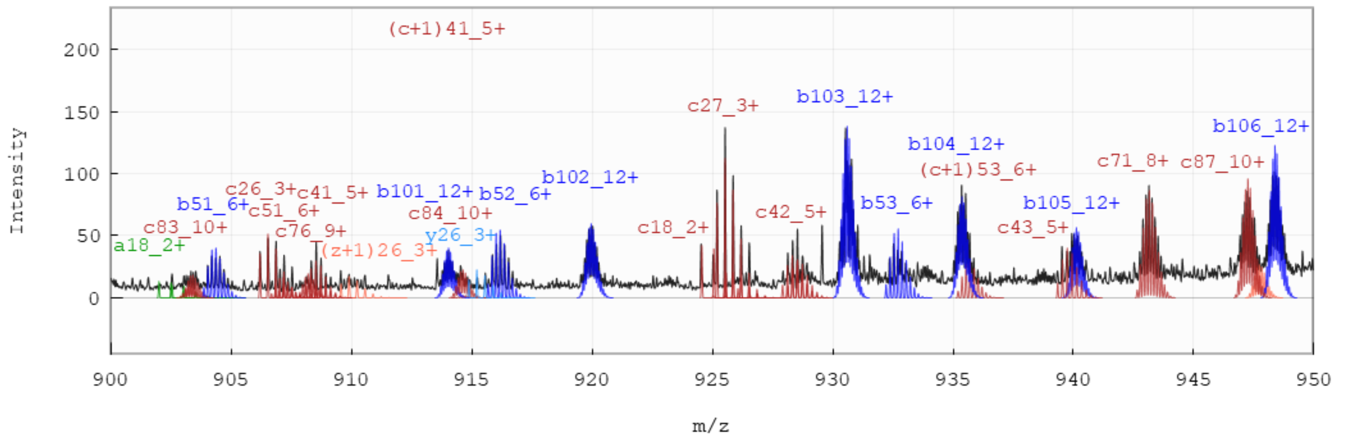

H2A N-acS 14+

950-1000 *m/z*

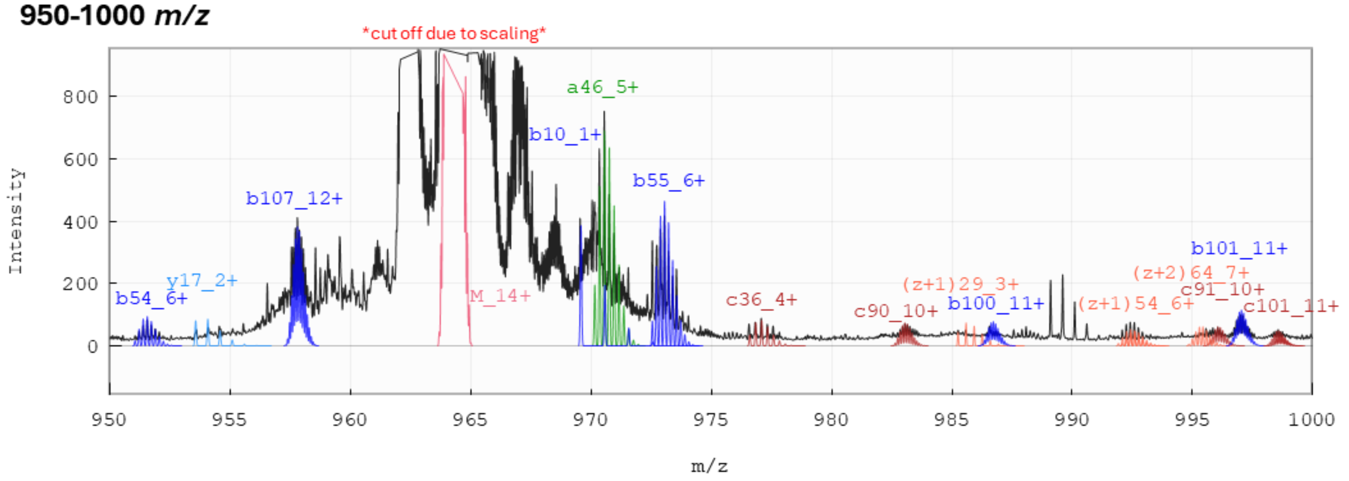

1000-1050 *m/z*

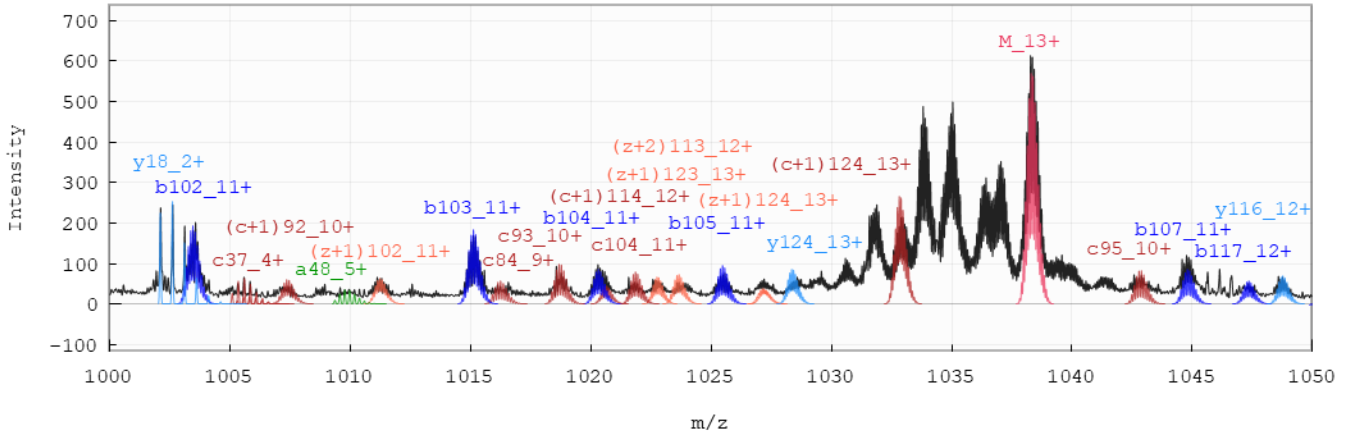

1050-1100 *m/z*

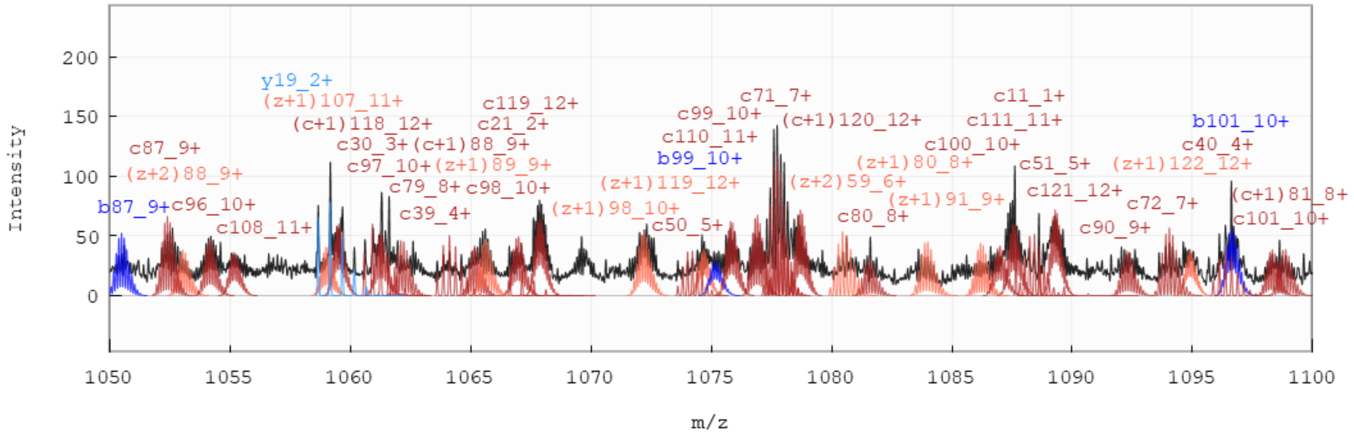

H2A N-acS 14+

1100-1200 *m/z*

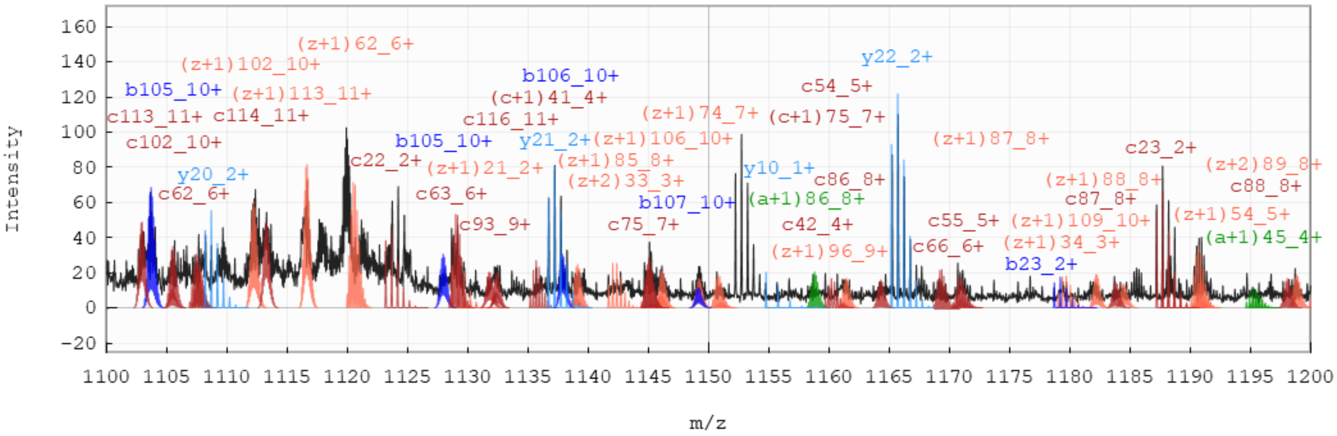

1200-1300 *m/z*

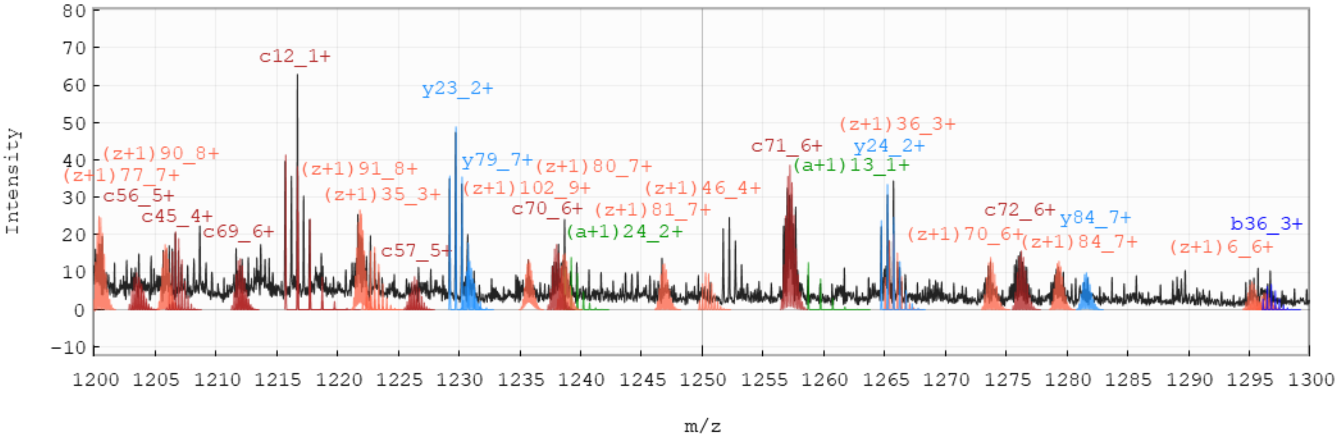

1300-1400 *m/z*

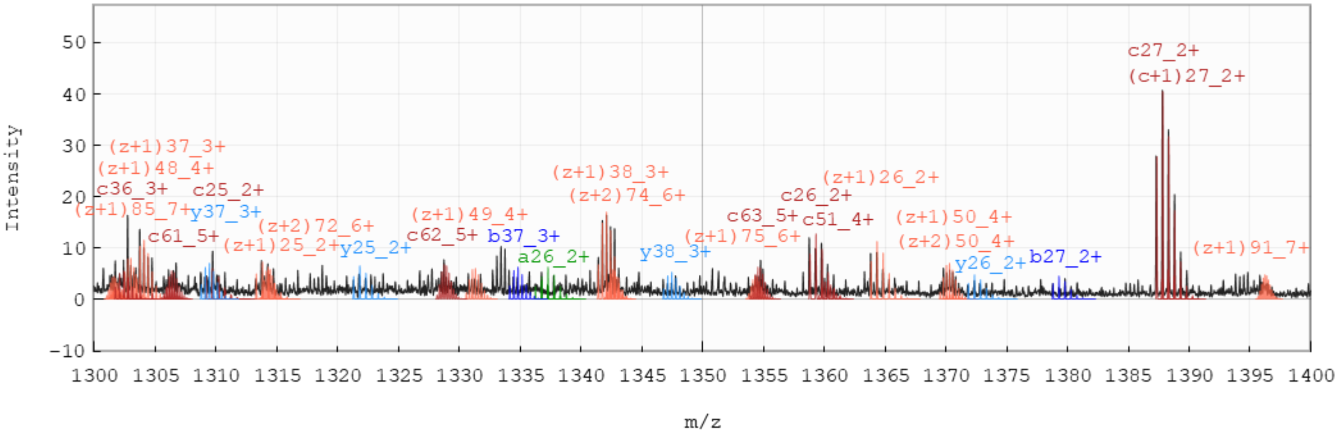

H2A N-acS 14+

1400-1500 *m/z*

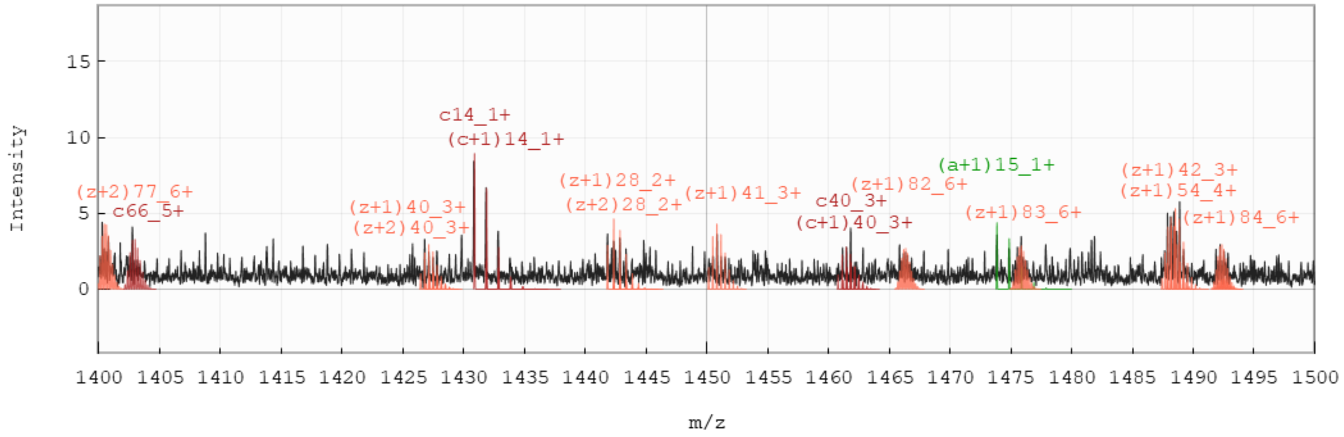

1500-1700 *m/z*

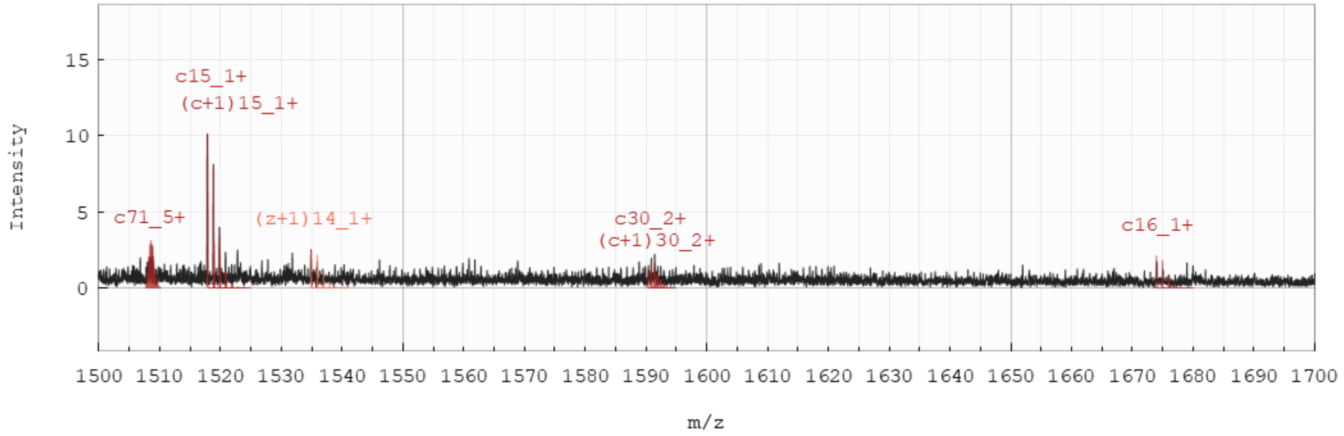

Figure S53. Ion annotation of MS/MS spectrum for H2B-1 N-me3A [M+16H]$^{16+}$.

H2B-1 N-me3A 16+

400-500 *m/z*

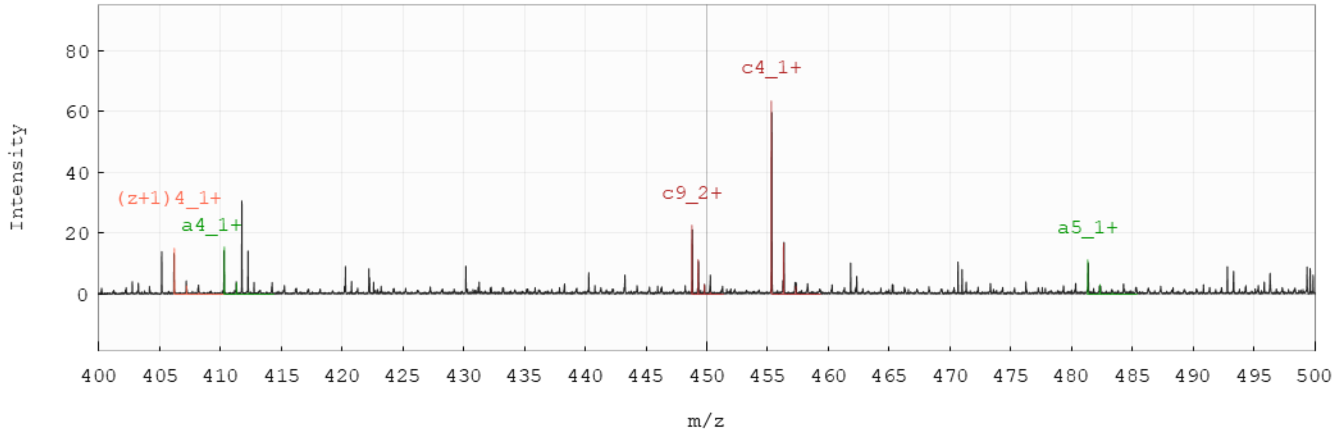

500-600 *m/z*

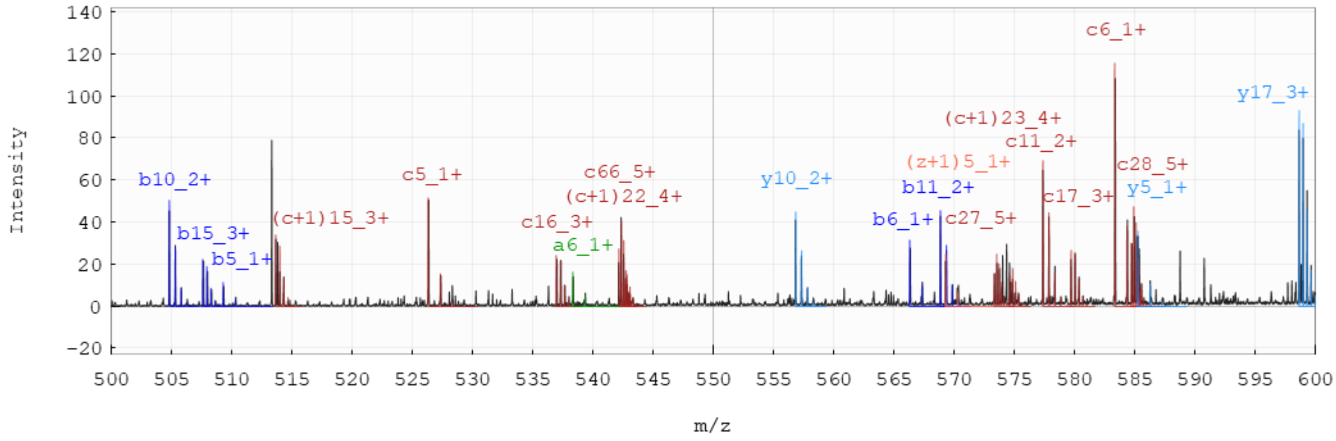

600-700 *m/z*

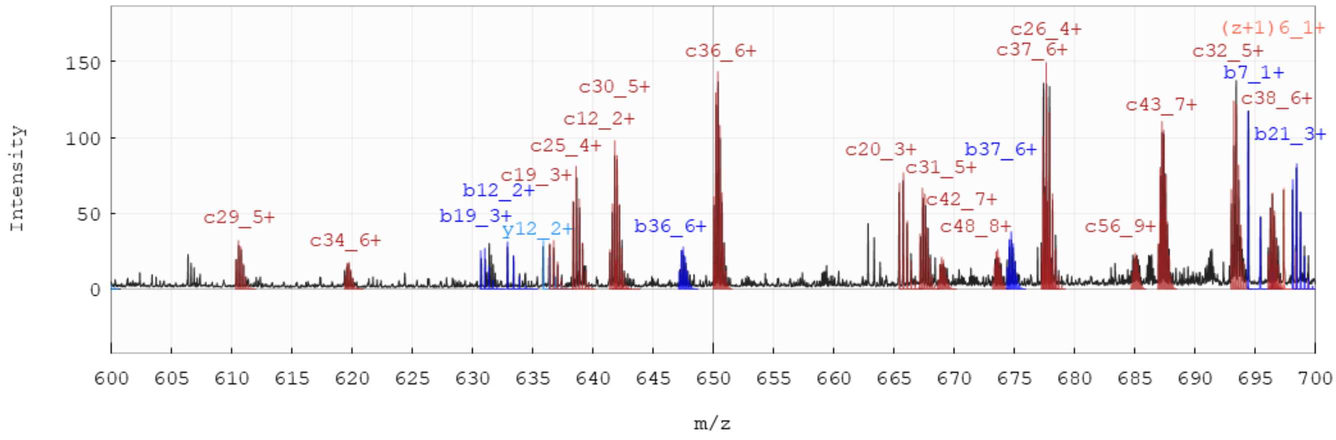

H2B-1 N-me3A 16+

700-800 *m/z*

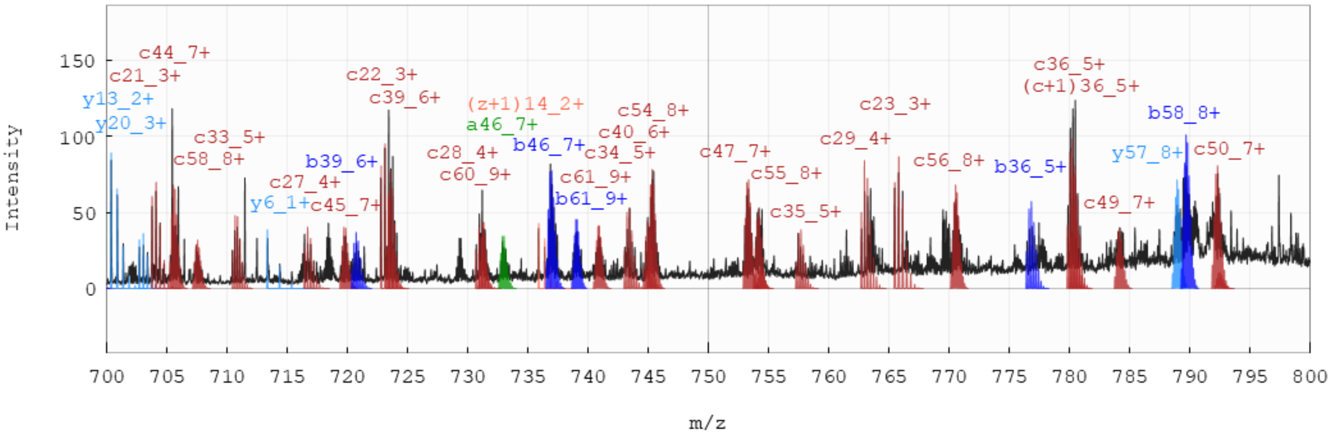

800-850 *m/z*

cut off due to scaling

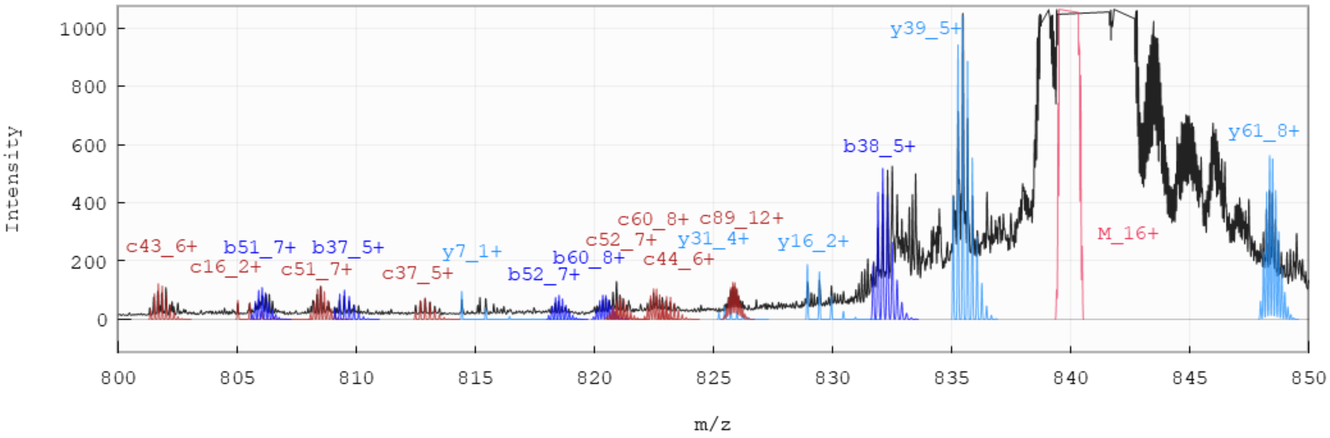

850-900 *m/z*

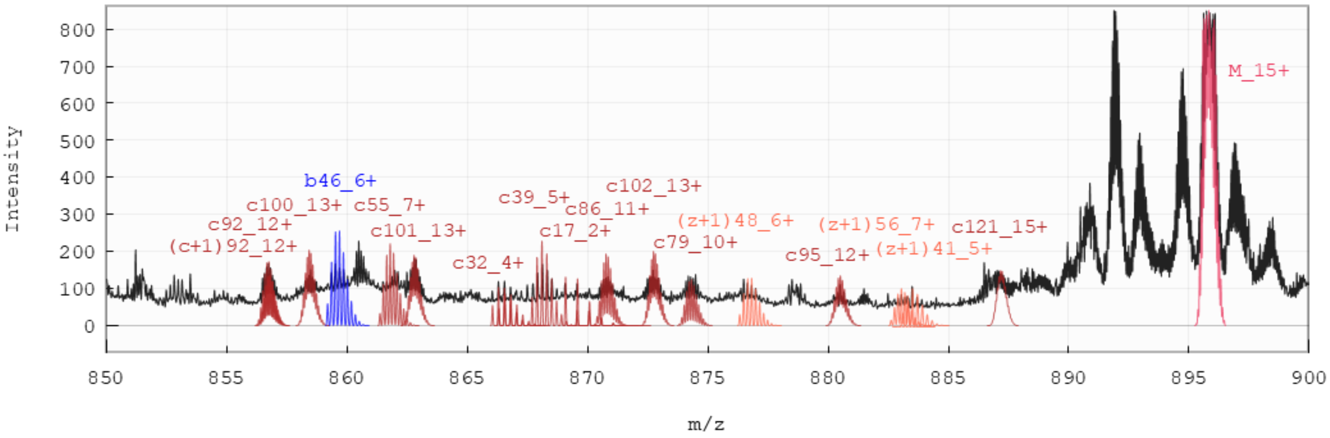

H2B-1 N-me3A 16+

900-950 *m/z*

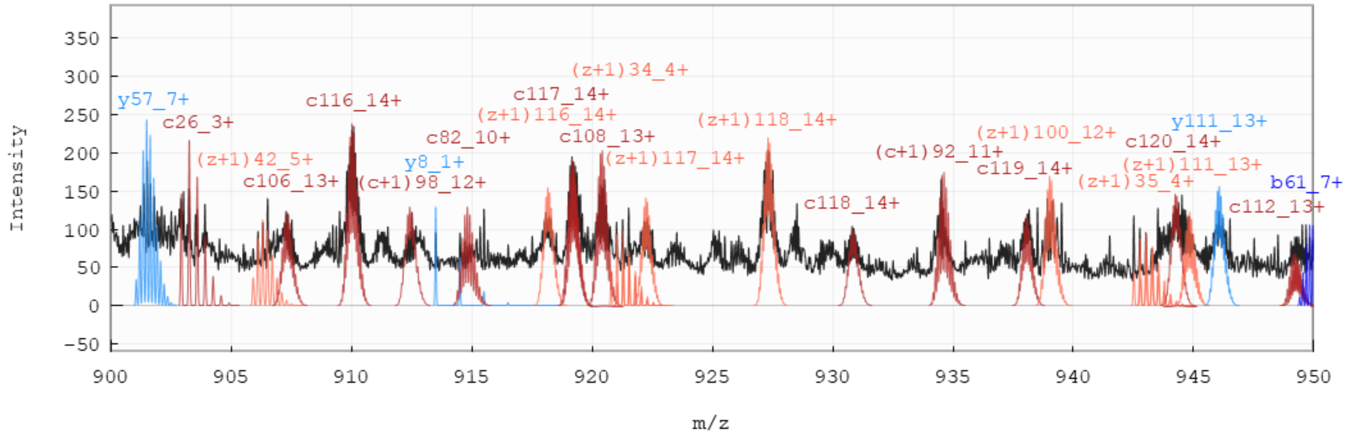

950-1000 *m/z*

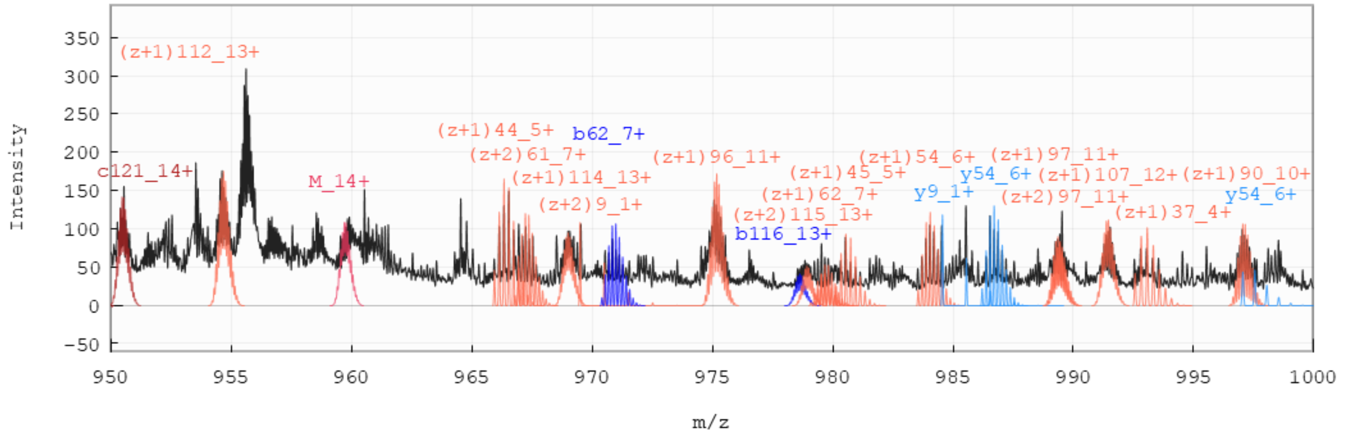

1000-1100 *m/z*

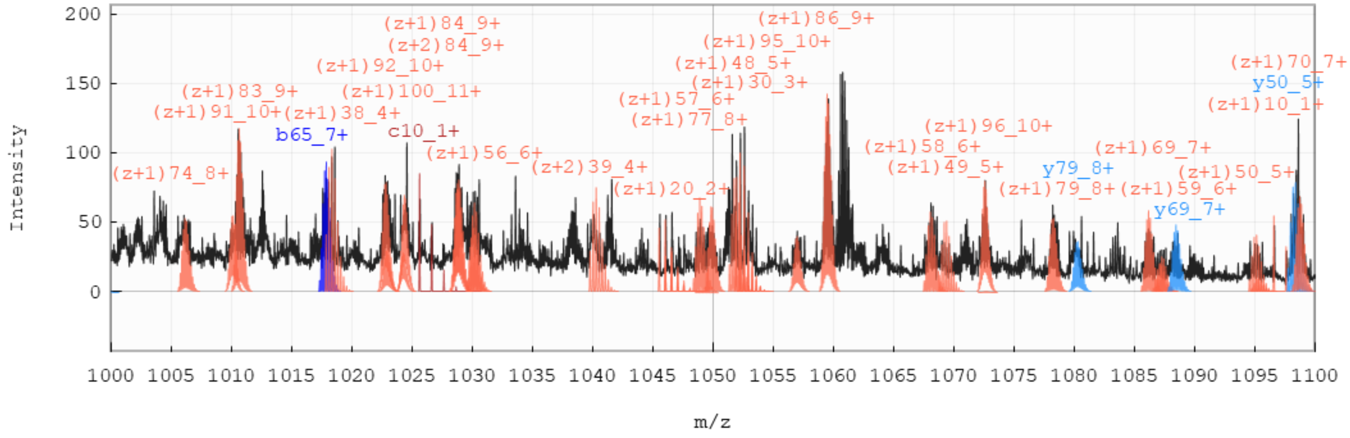

H2B-1 N-me3A 16+

1100-1200 *m/z*

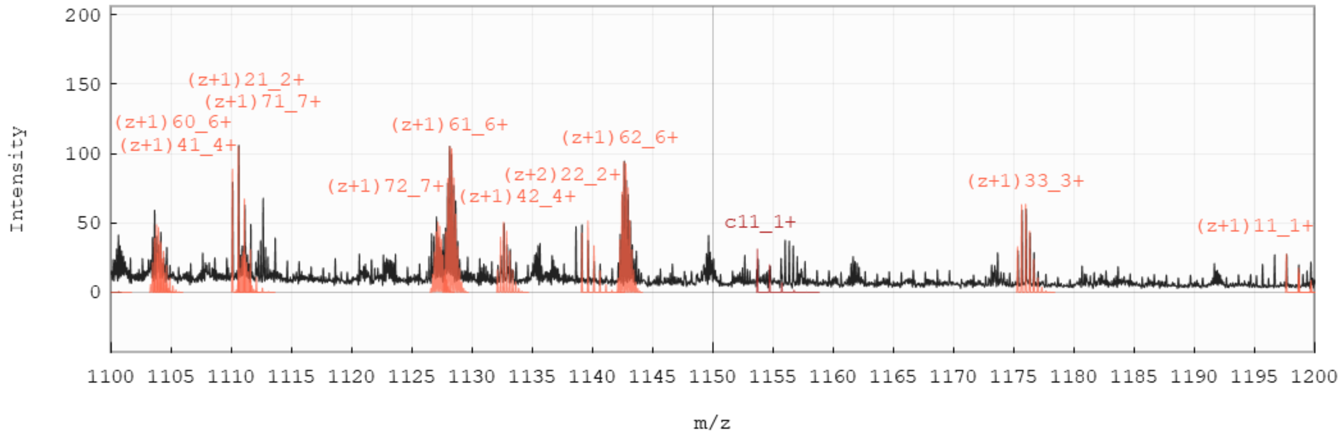

1200-1300 *m/z*

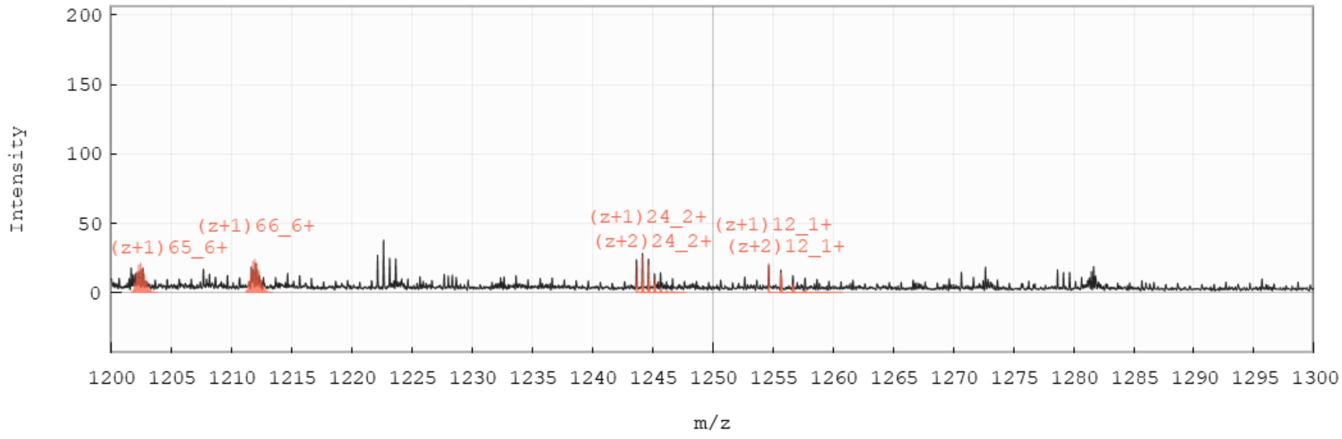

Figure S54. Ion annotation of MS/MS spectrum for H2B-2 N-me3A $[M+15H]^{15+}$.

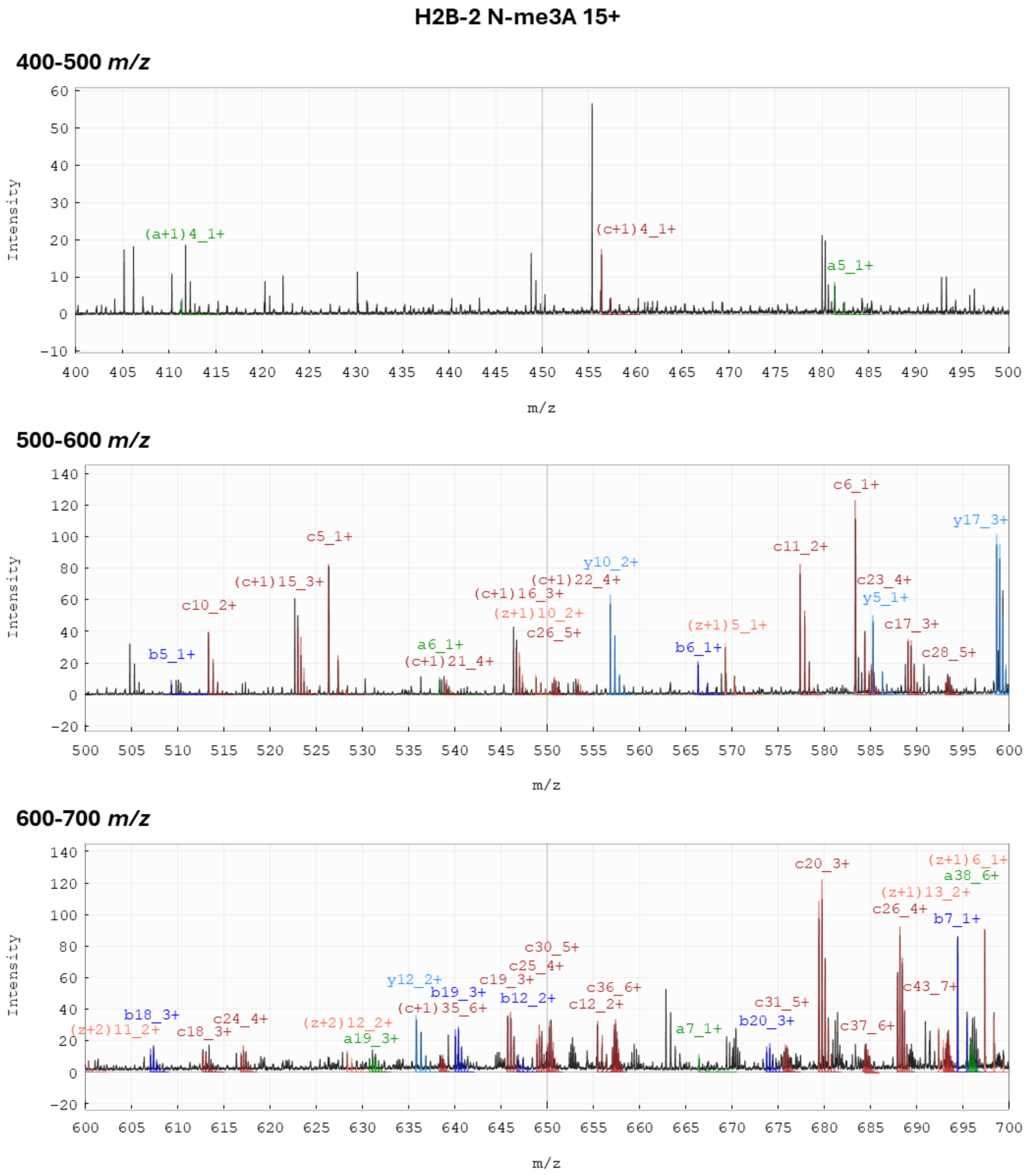

H2B-2 N-me3A 15+

700-800 *m/z*

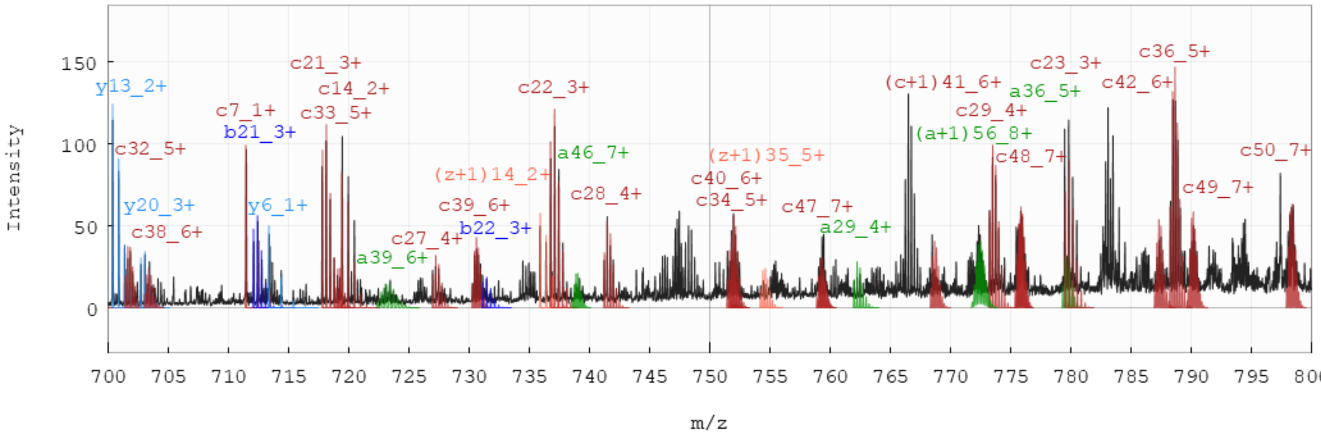

800-850 *m/z*

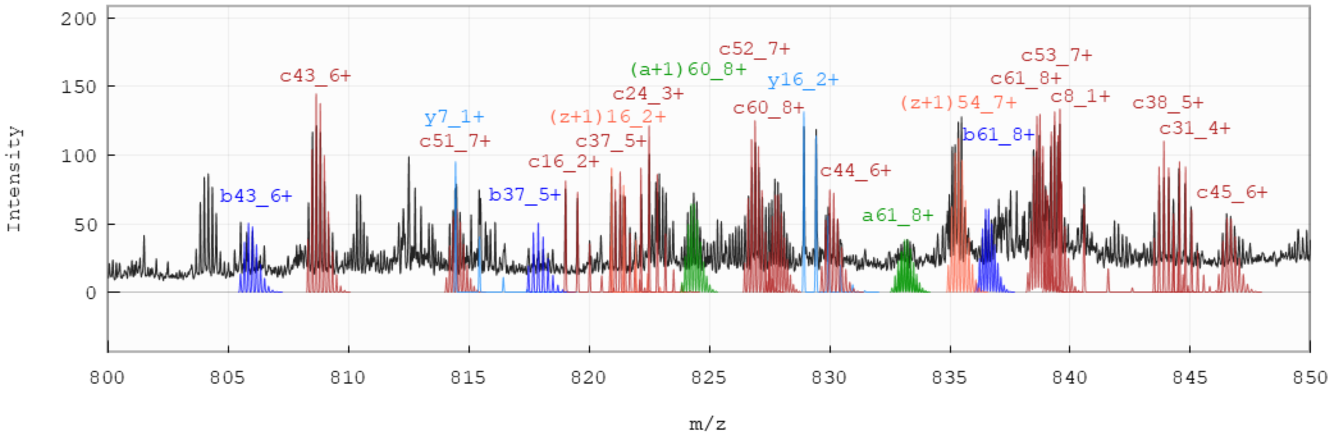

850-900 *m/z*

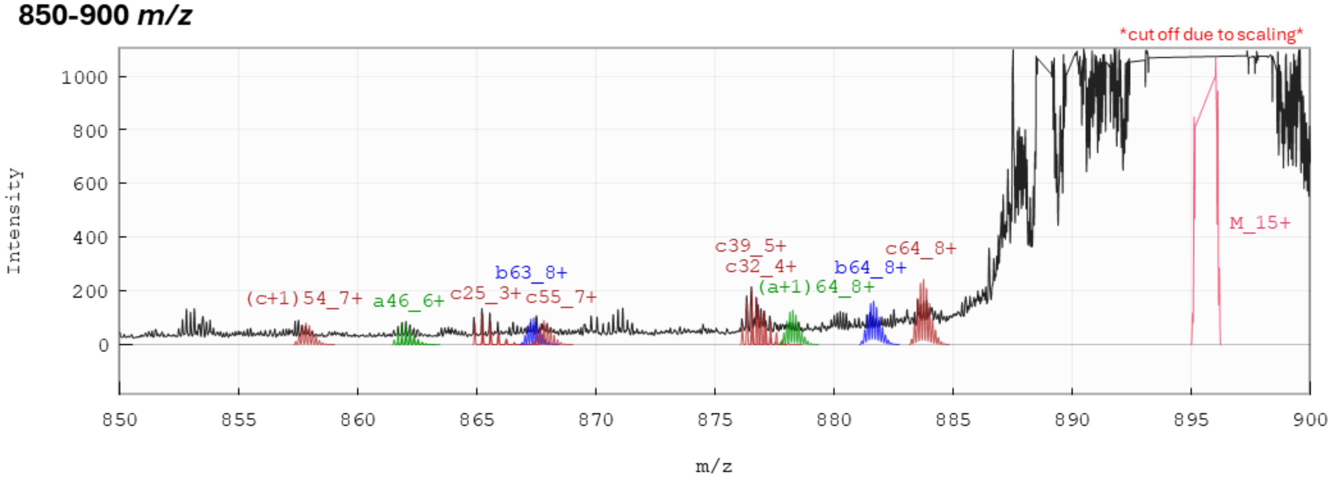

H2B-2 N-me3A 15+

900-950 *m/z*

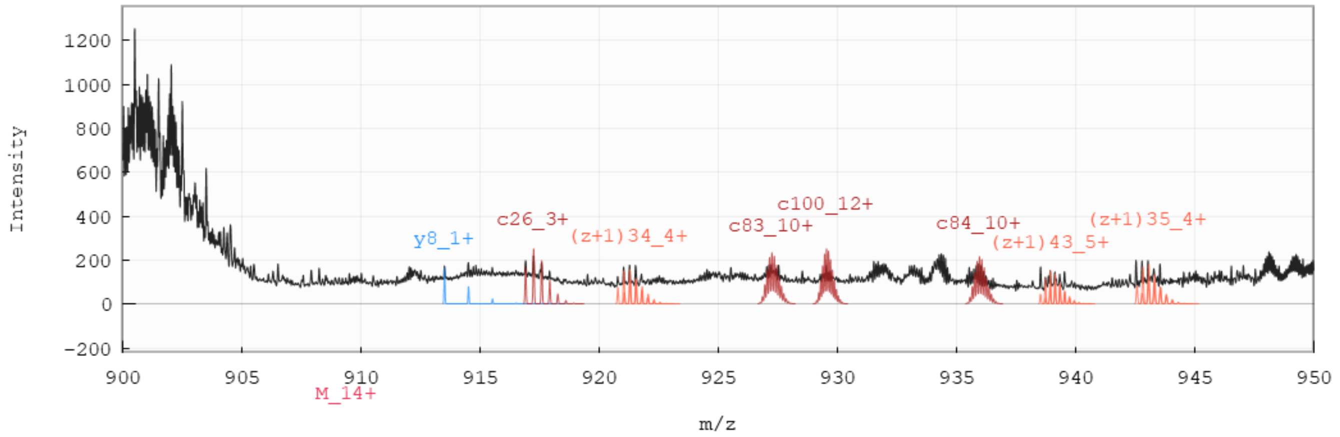

950-1000 *m/z*

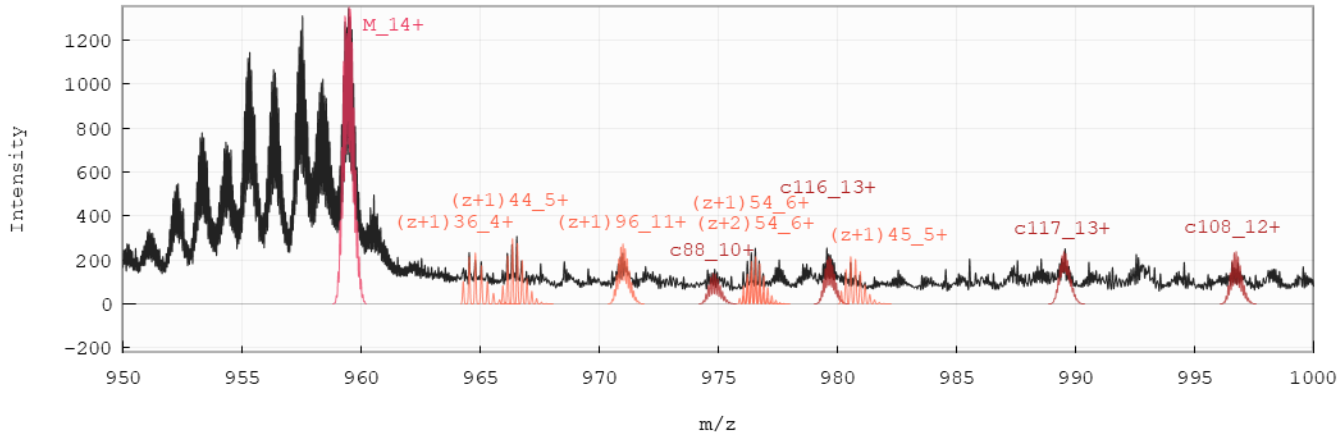

1000-1100 *m/z*

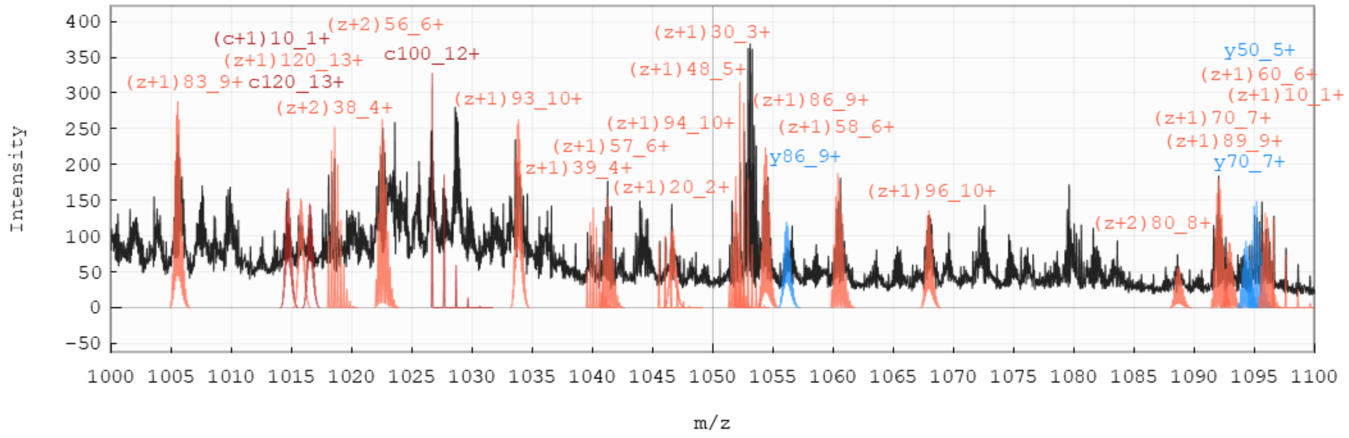

H2B-2 N-me3A 15+

1100-1200 *m/z*

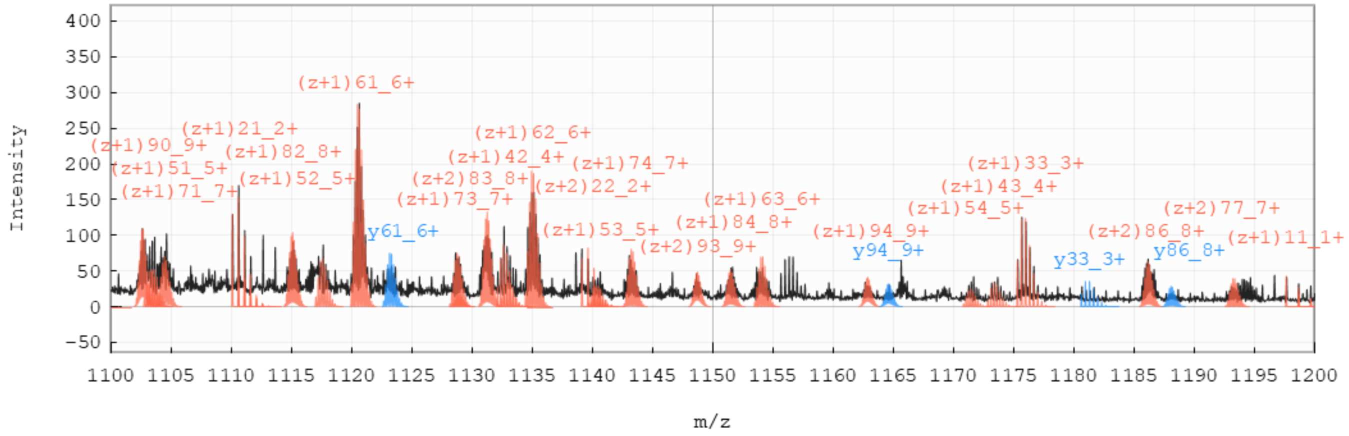

1200-1300 *m/z*

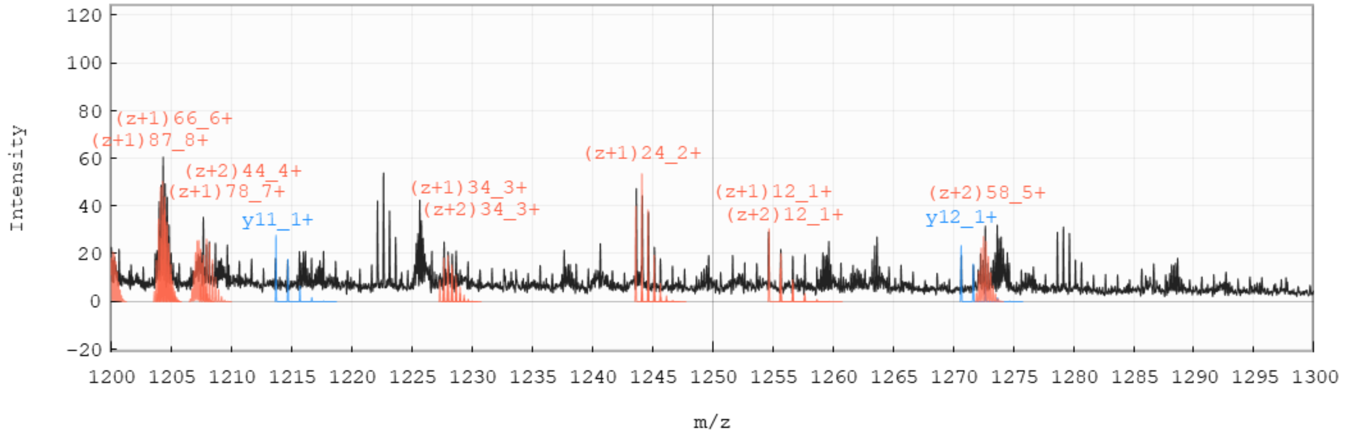

1300-1450 *m/z*

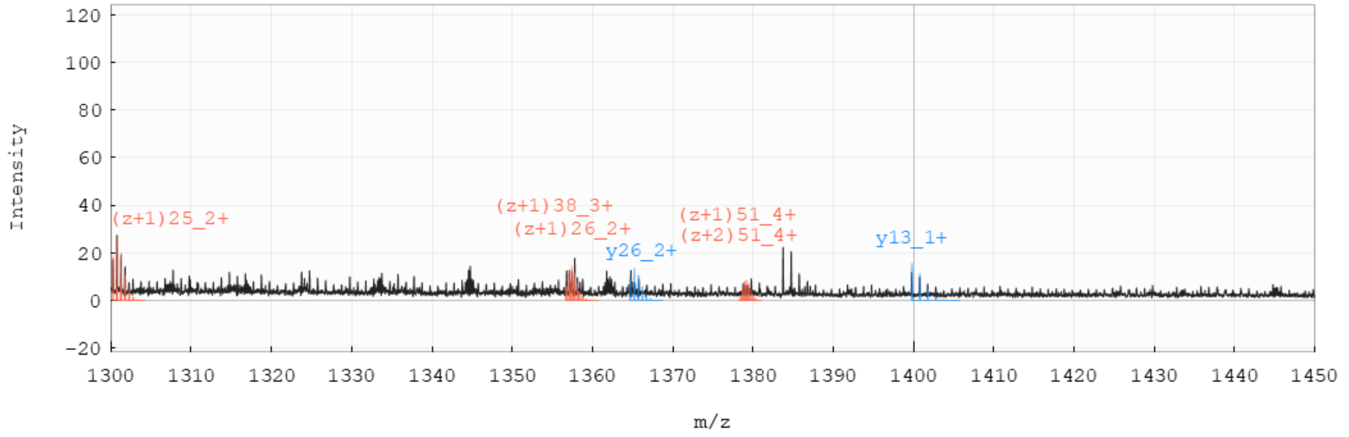

Figure S55. Ion annotation of MS/MS spectrum for H2B-3 N-me3A [M+14H]$^{14+}$.

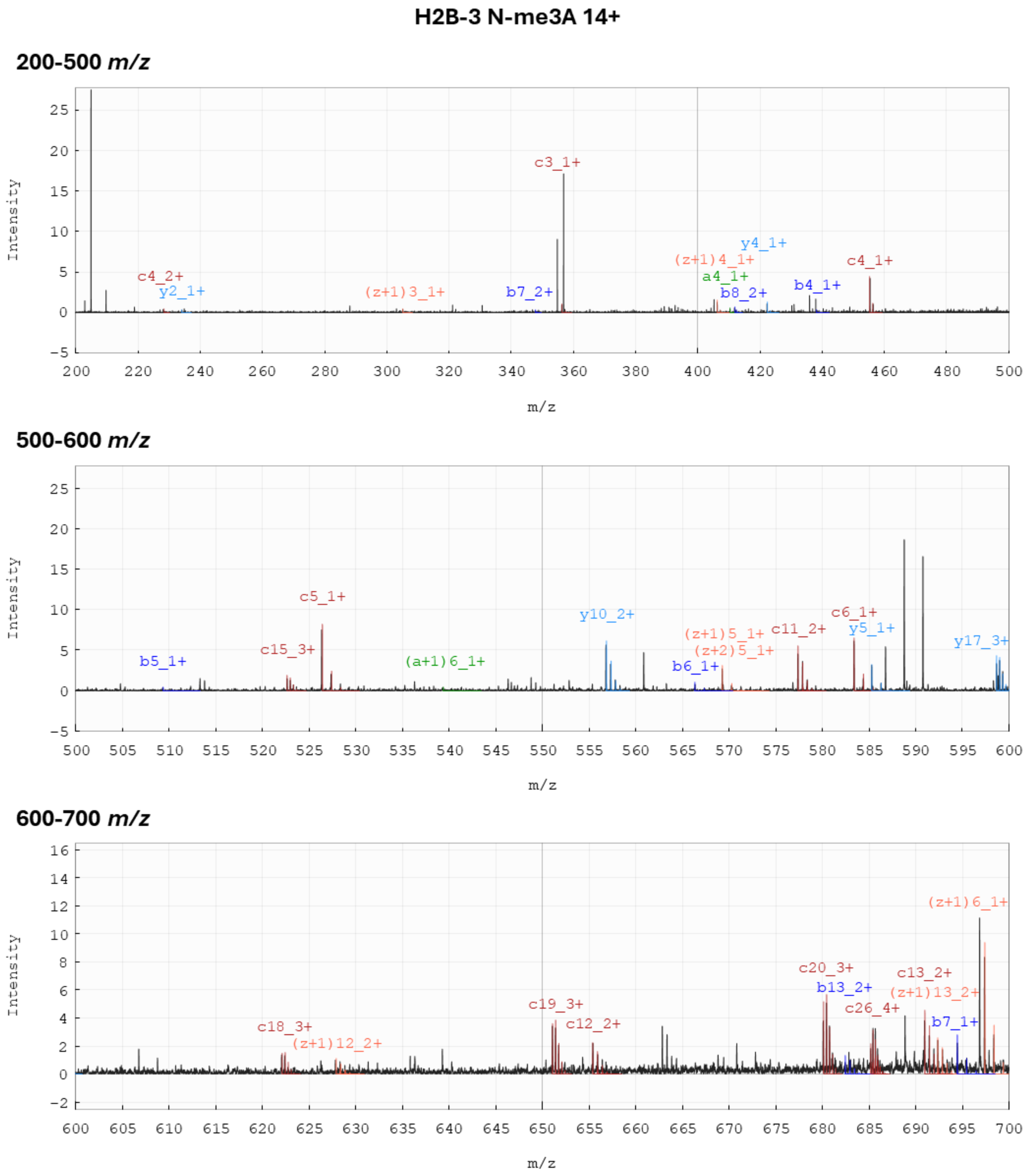

H2B-3 N-me3A 14+

700-800 *m/z*

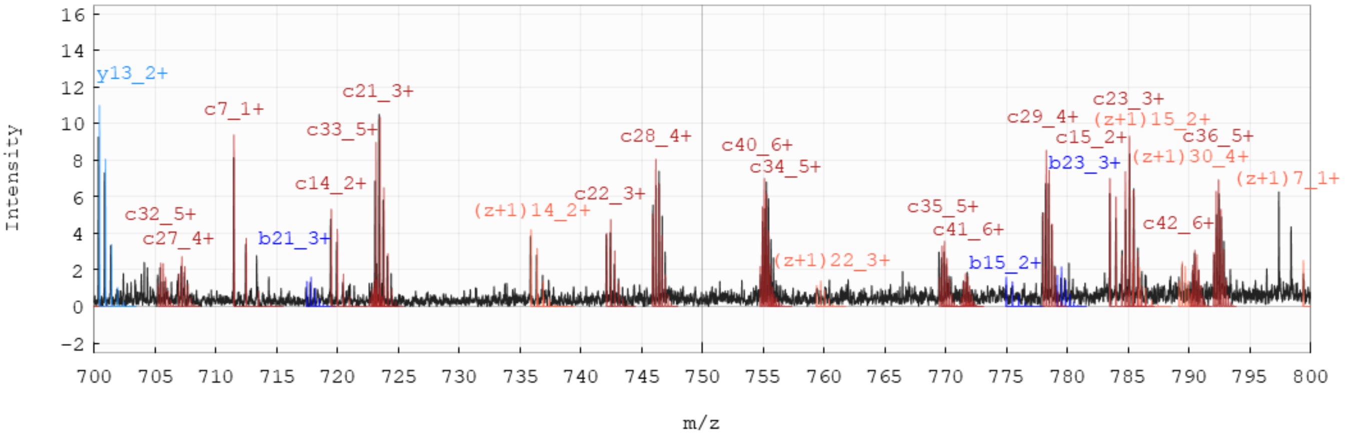

800-900 *m/z*

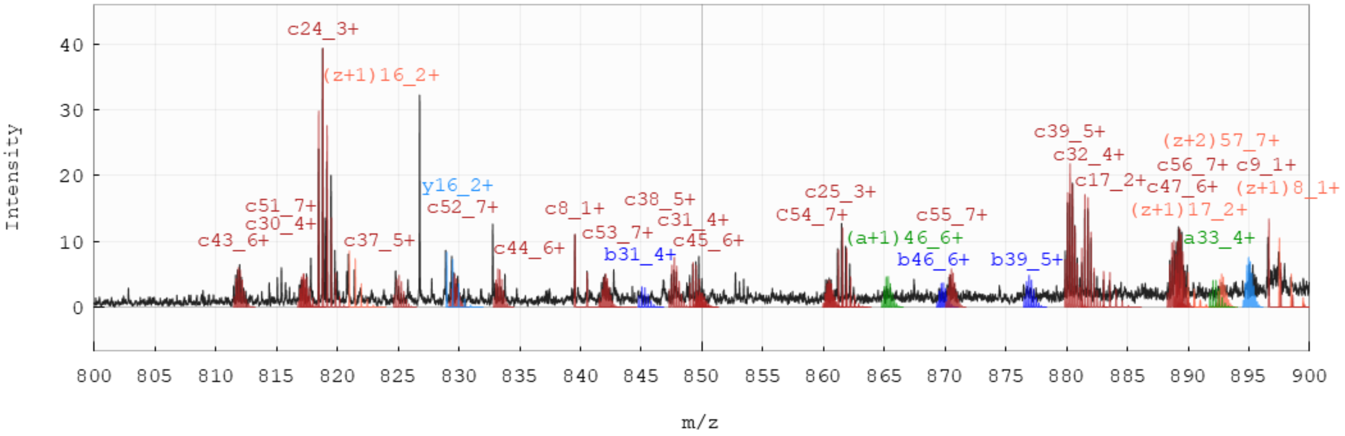

900-950 *m/z*

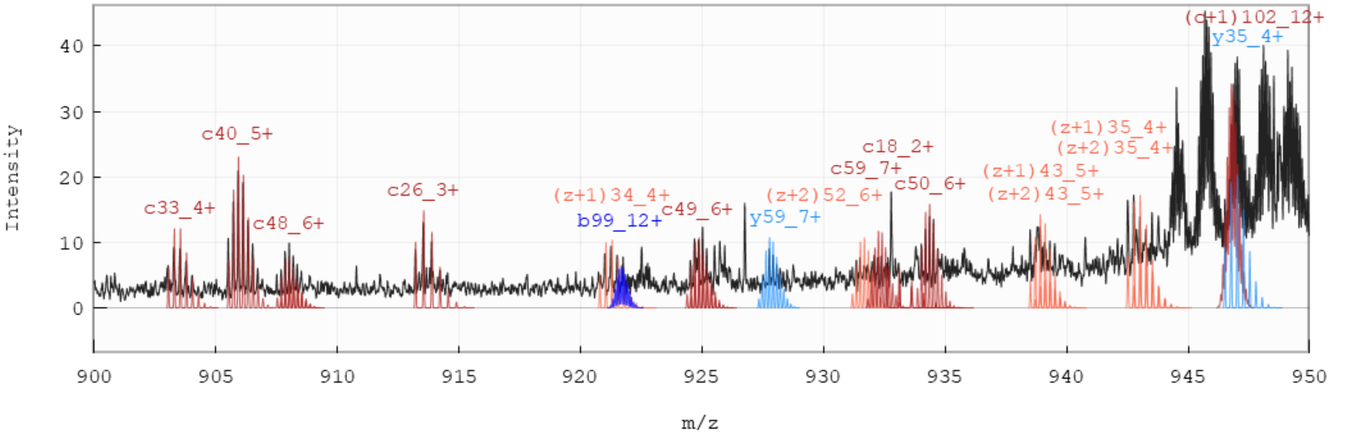

H2B-3 N-me3A 14+

950-1000 *m/z*

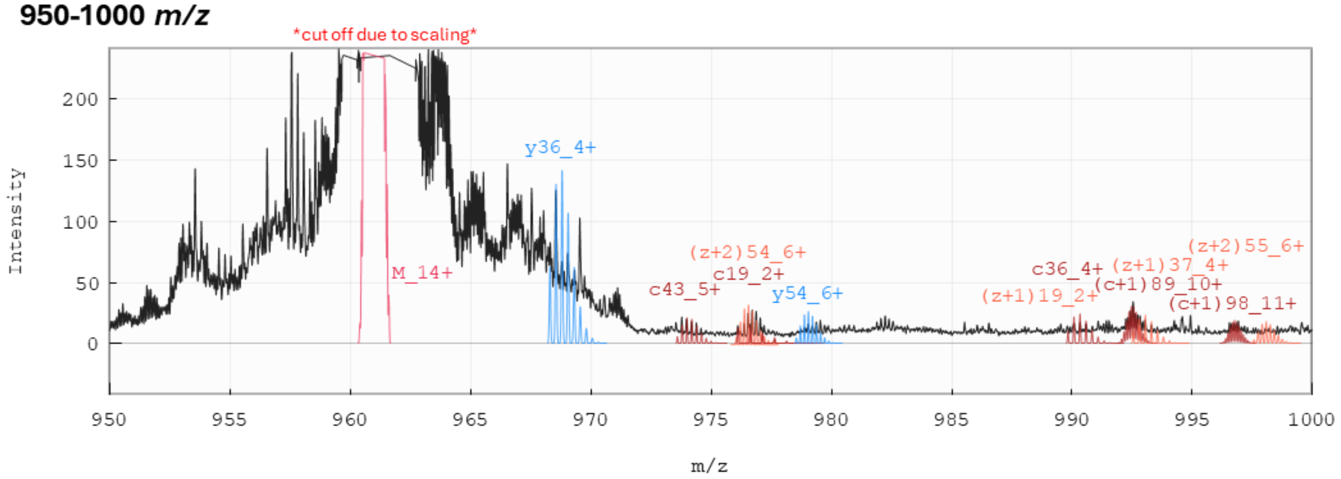

1000-1050 *m/z*

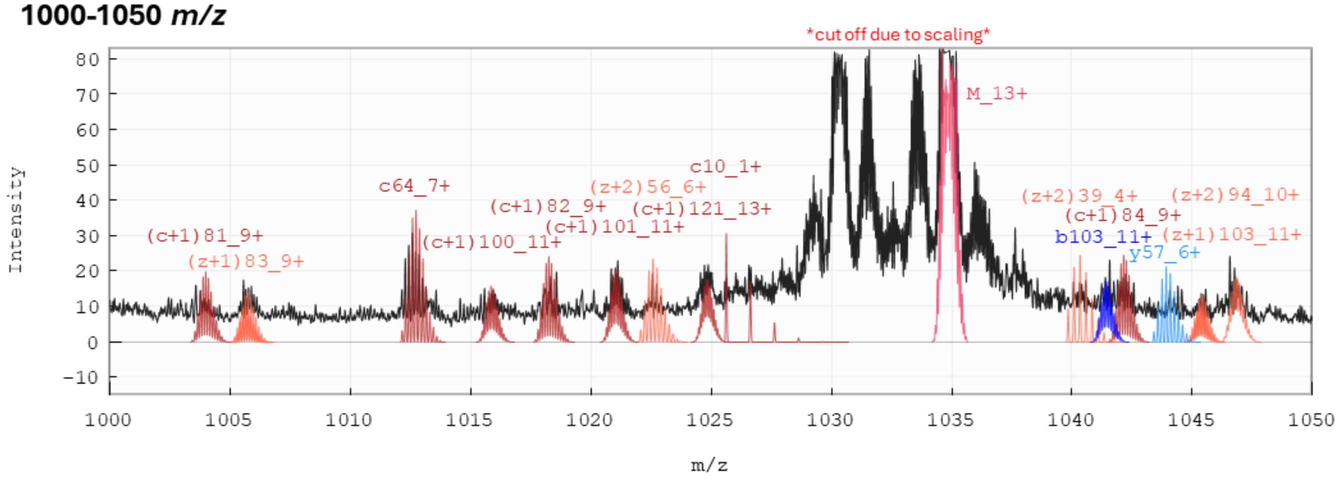

1050-1100 *m/z*

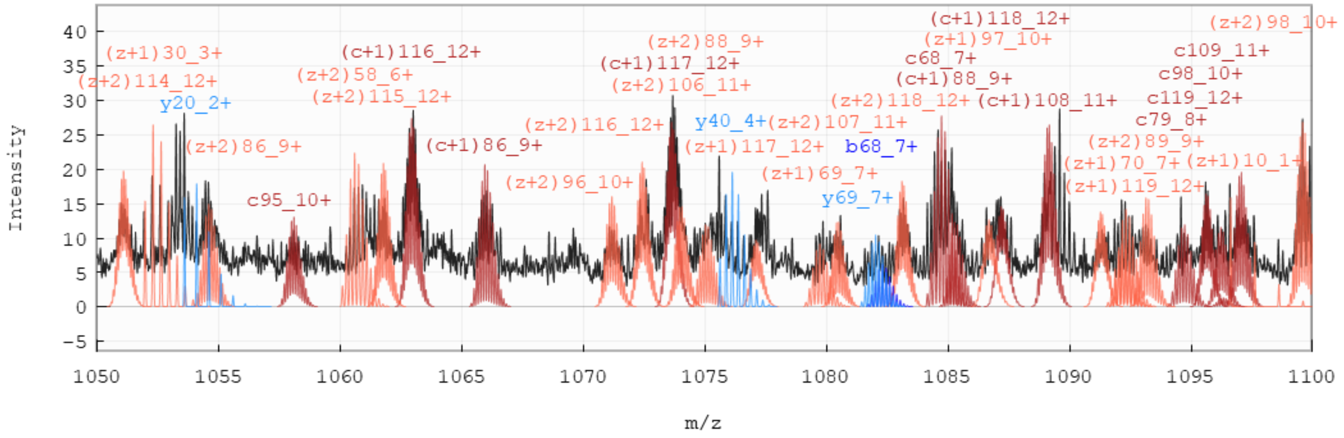

H2B-3 N-me3A 14+

1100-1150 *m/z*

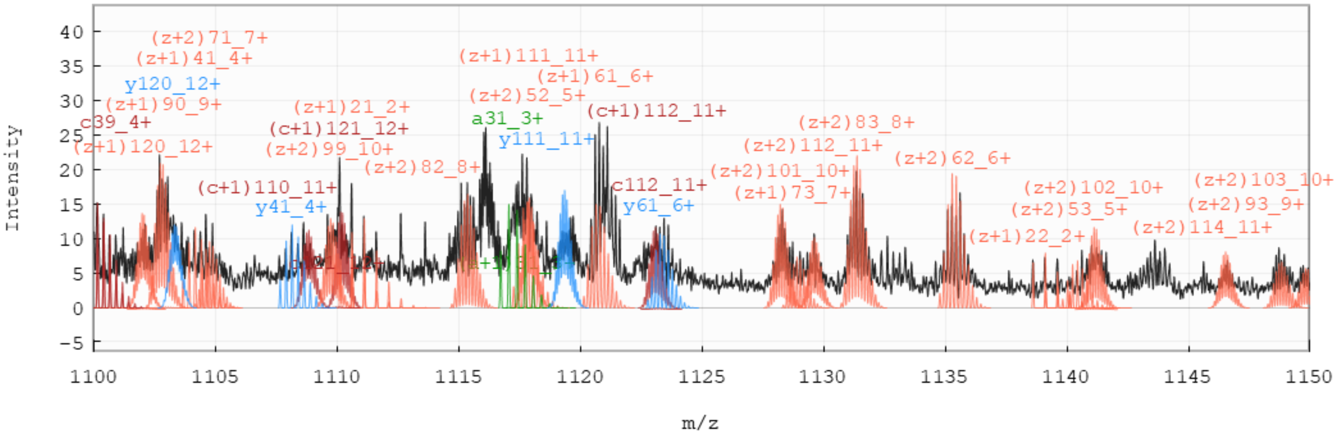

1150-1200 *m/z*

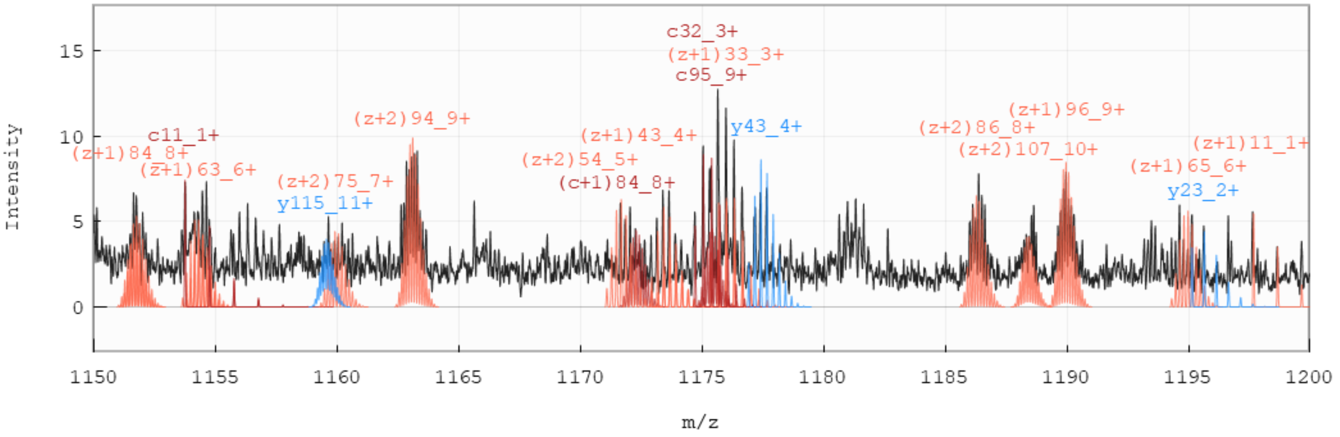

1200-1300 *m/z*

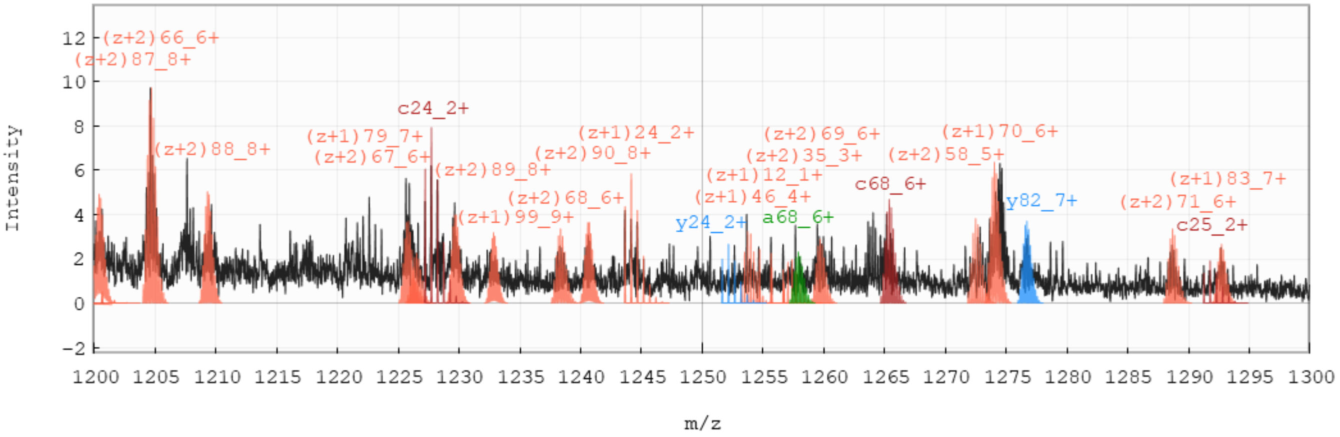

H2B-3 N-me3A 14+

1300-1500 *m/z*

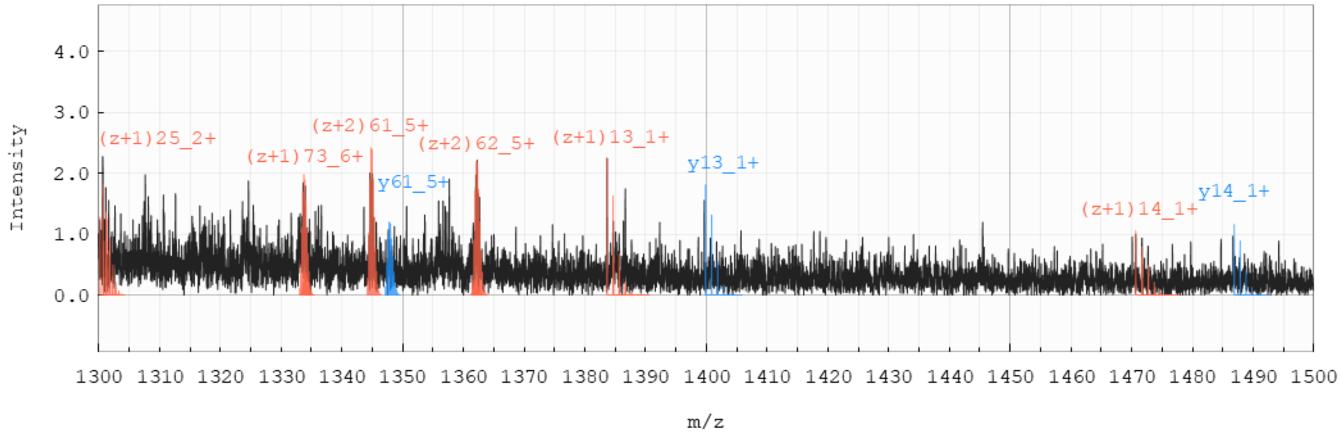

Figure S56. Bottom-up DataAnalysis script and example script input.

Script

```
'Extracts EIC based on m/z and mobRANGE PT 2
option explicit
Dim K, num, Nchrom, counter, listlength, charge
Dim inpFil
Dim fso, EIC, Width, Size
Dim Formul(),Charg(),Mz(),Mobrange(),RTrang1(),RTrang2()
Dim TextLine, columns
Dim MyChromatogram

Set fso = CreateObject("Scripting.FileSystemObject")

Const ForReading = 1
'Read m/z; IM; adduct list
Set inpFil = fso.OpenTextFile("Input file path", ForReading)
listlength = 0
Do While Not inpFil.AtEndOfStream
       TextLine = inpFil.ReadLine
       If TextLine <> "END" Then
           listlength = listlength + 1
       End If
Loop
inpFil.Close

ReDim Formul(listlength)
ReDim Charg(listlength)
ReDim Mz(listlength)
ReDim Mobrange(listlength)
ReDim RTrang1(listlength)
ReDim RTrang2(listlength)

' Read the file again and split into columns
counter = 0
Set inpFil = fso.OpenTextFile("Input file path", ForReading)
Do While Not inpFil.AtEndOfStream
       TextLine = inpFil.ReadLine
       If TextLine <> "END" Then
           columns = Split(TextLine, ";")
           If UBound(columns) = 6 Then
               Formul(counter) = columns(0)
               Charg(counter) = columns(1)
               Mz(counter) = columns(2)
               Mobrange(counter) = columns(3)
               RTrang1(counter) = columns(4)
               RTrang2(counter) = columns(5)
               counter = counter + 1
           End If
       End If
Loop
inpFil.Close

Size = counter
Width = 0.02 'will take m/z +/- width/2
'Charge = 2 'Fixed charge state for lipids > 1
```

```
Analysis.Compounds.Clear
'Analysis.Chromatograms.DeleteChromatogram(k)


Nchrom = Analysis.Chromatograms.count

For K = 1 To Size - 1
    Set EIC = CreateObject("DataAnalysis.EICChromatogramDefinition")
    EIC.MSFilter.Type = daMSFilterMS
    EIC.Polarity = daPositive
    EIC.Range = CStr(Mz(K))             '    EIC.Formula = Mass(K)
    EIC.WidthRight = CStr(Width / 2)
    EIC.WidthLeft = CStr(Width / 2)
'    EIC.Adducts = Adduct(K)
'    EIC.Charges = CStr(charge)
    EIC.MobilityRange = Mobrange(K)
    EIC.Color = daRed
    Analysis.Chromatograms.AddChromatogram EIC
    num = Nchrom + K

    'Extracting the compound spectra from the identified peaks in the
chromatogram.
    'set MyChromatogram = Analysis.Chromatograms(num)
    'MyChromatogram.FindCompounds
Next

Analysis.Save

MsgBox "M/Z + MOB RANGE -> LC extraction done."

'K=0
for K=1 to listlength-1
'MsgBox RTrang(k-1)
Analysis.Chromatograms(K).AddRangeSelection RTrang1(K),RTrang2(K)
'MSgBox Cstr(RTrang1(K))+ Cstr (RTrang2(K))
  ' given as range in min 10, 15
next

Analysis.Chromatograms.IntegrateOnly

form.close
```

Example Input

```
#formula ; charge ; M/Z ; mobrange ; RT start ; RT end ;
QC1 ; 2 ; 471.7538 ; 0.760-0.850 ; 12 ; 18 ;
QC2 ; 1 ; 739.3833 ; 0.980-1.120 ; 23 ; 30 ;
pr-GK(pr)GGK(pr)GLGK(pr)GGAK(pr)R ; 2 ; 775.9543 ; 1.004-1.165 ; 32 ; 38 ;
```

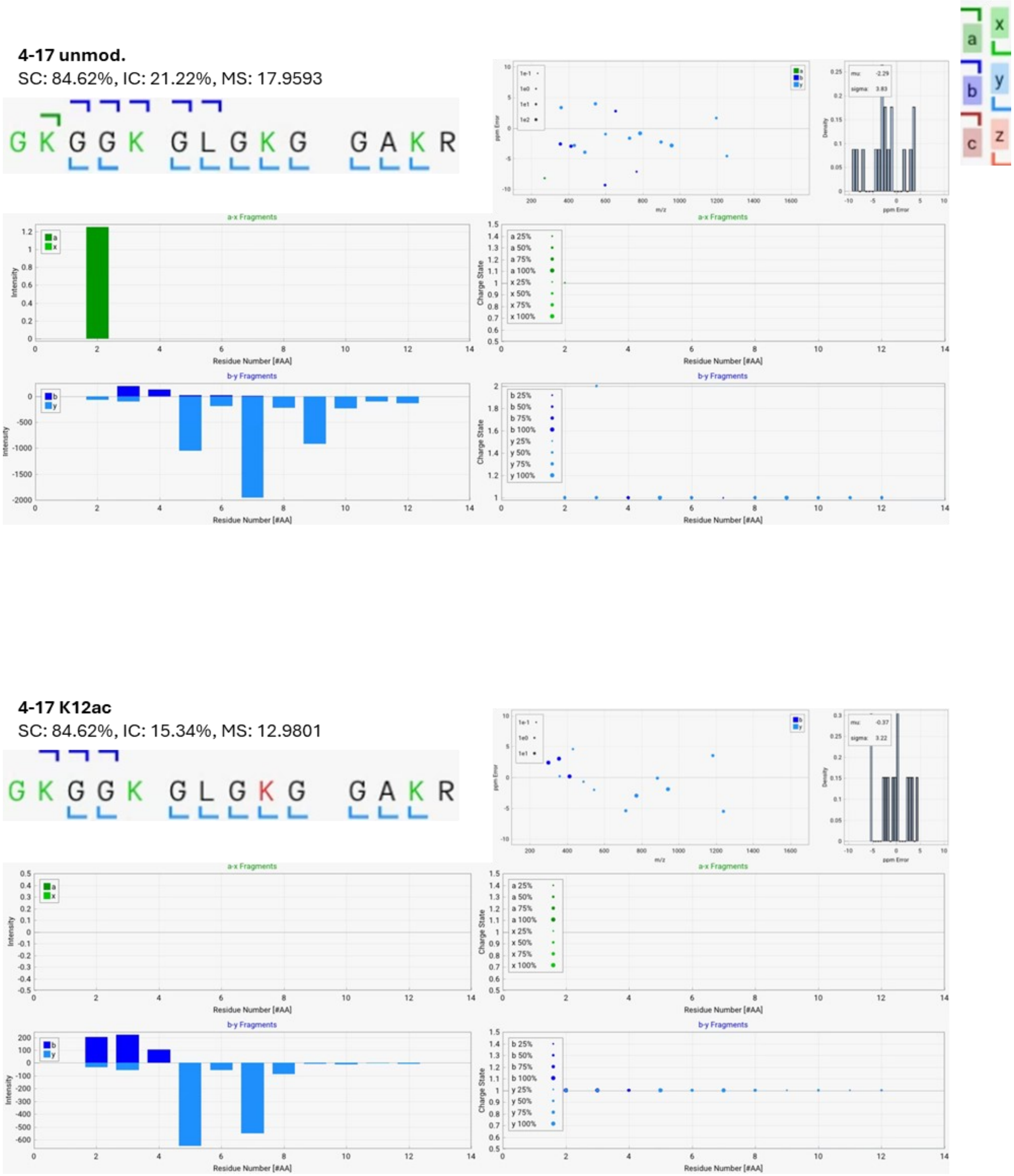

Figure S57. Replicate 1 bottom-up fragmentation of *A. cervicornis* H4 4-17 and 20-23 PTM patterns with ±10 ppm error: 4-17 unmodified and 4-17 K12ac.

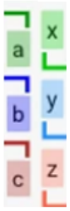

4-17 K16ac

SC: 76.92%, IC: 16.88%, MS: 12.9877

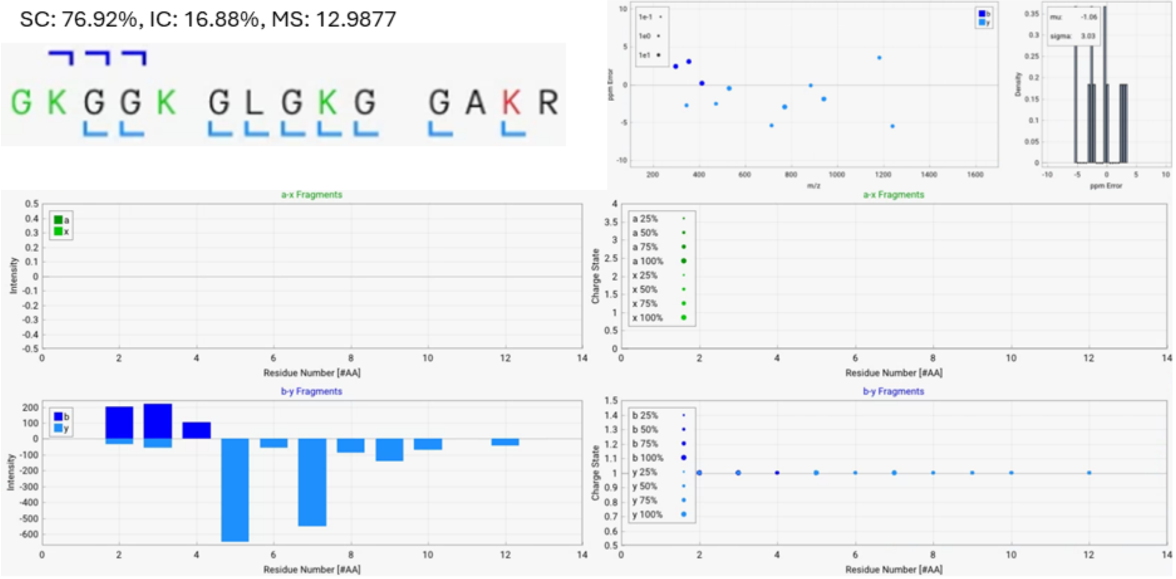

4-17 K5acK16ac

SC: 46.15%, IC: 6.30%, MS: 2.9085

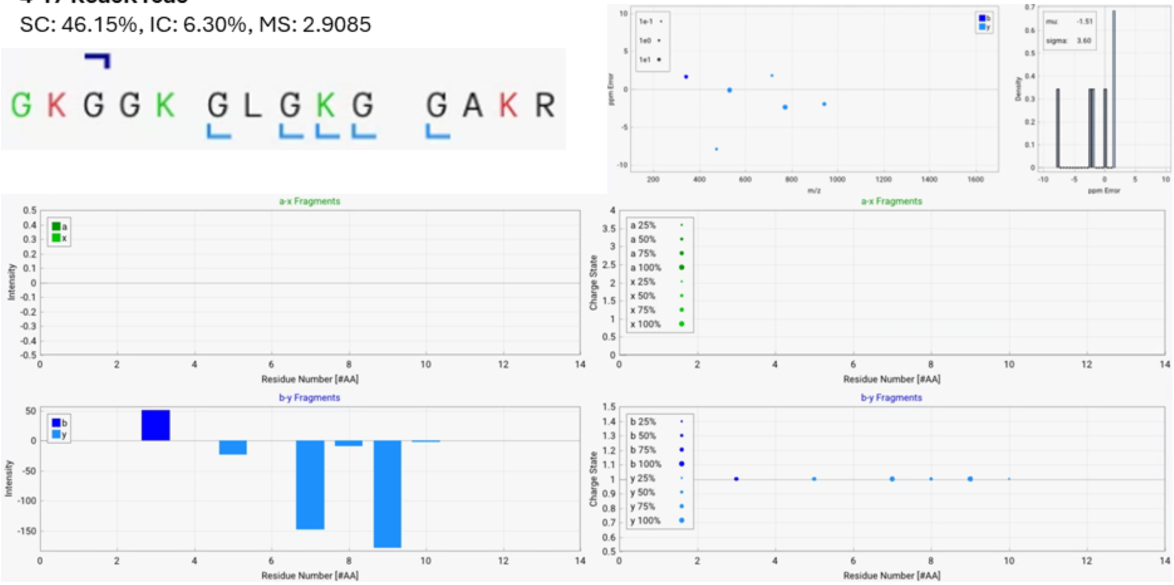

Figure S58. Replicate 1 bottom-up fragmentation of *A. cervicornis* H4 4-17 and 20-23 PTM patterns with ±10 ppm error: 4-17 K16ac and 4-17 K5acK16ac.

4-17 K8acK16ac

SC: 61.54%, IC: 9.06%, MS: 5.5728

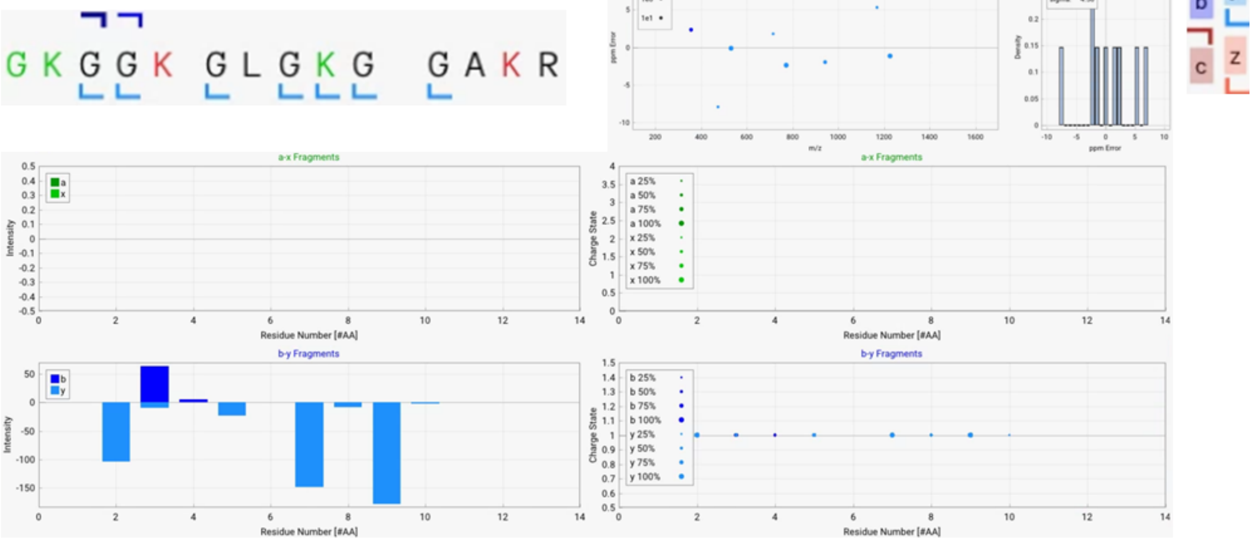

4-17 K12acK16ac

SC: 61.54%, IC: 8.28%, MS: 5.0930

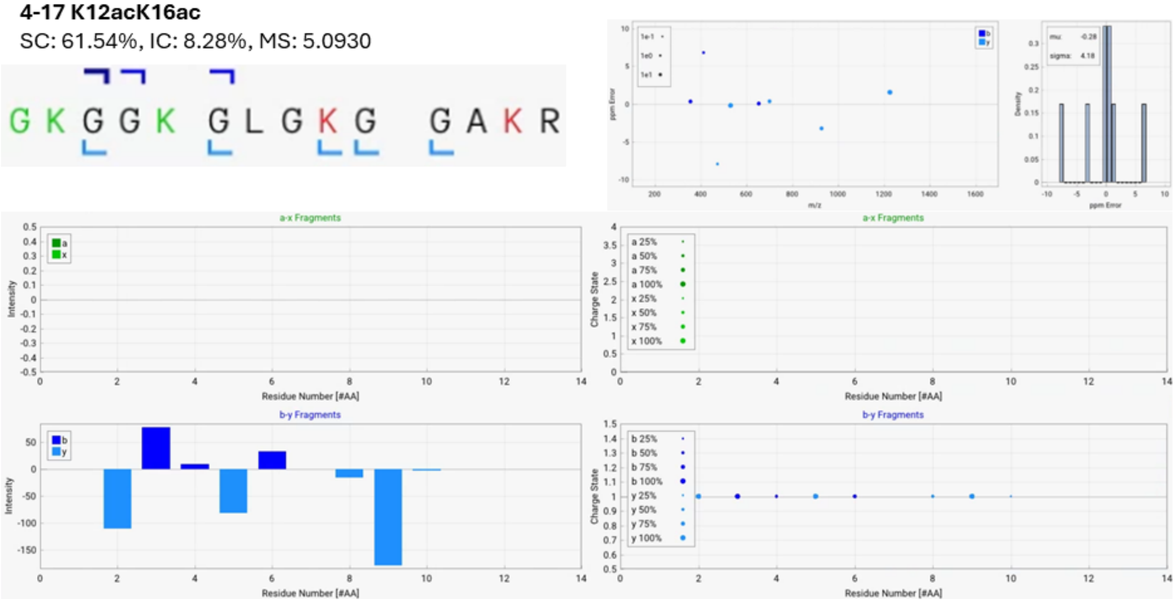

Figure S59. Replicate 1 bottom-up fragmentation of *A. cervicornis* H4 4-17 and 20-23 PTM patterns with ±10 ppm error: 4-17 K8acK16ac and 4-17 K12acK16ac.

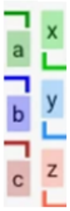

4-17 K5acK12acK16ac

SC: 69.23%, IC: 8.12%, MS: 5.6185

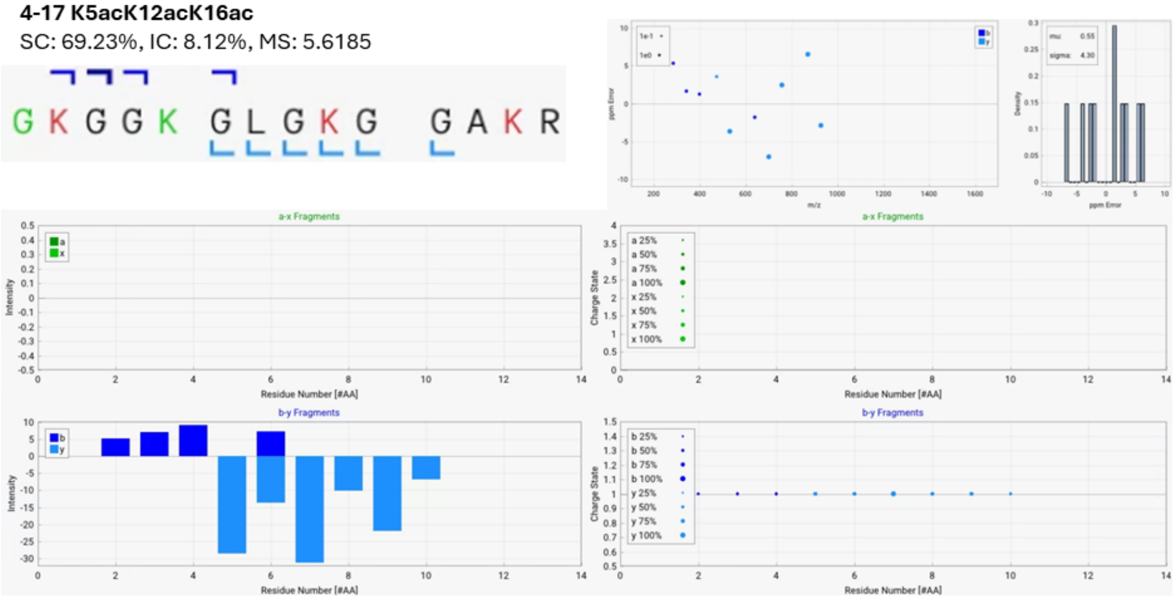

4-17 K8acK12acK16ac

SC: 69.23%, IC: 9.14%, MS: 6.3248

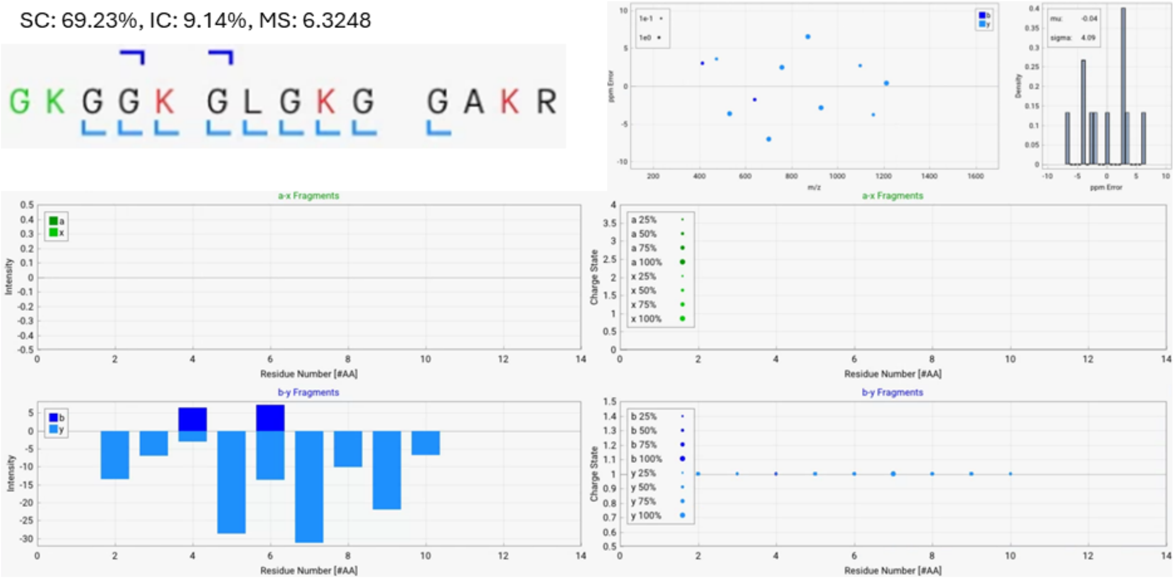

Figure S60. Replicate 1 bottom-up fragmentation of *A. cervicornis* H4 4-17 and 20-23 PTM patterns with ±10 ppm error: 4-17 K5acK12acK16ac and 4-17 K8acK12acK16ac.

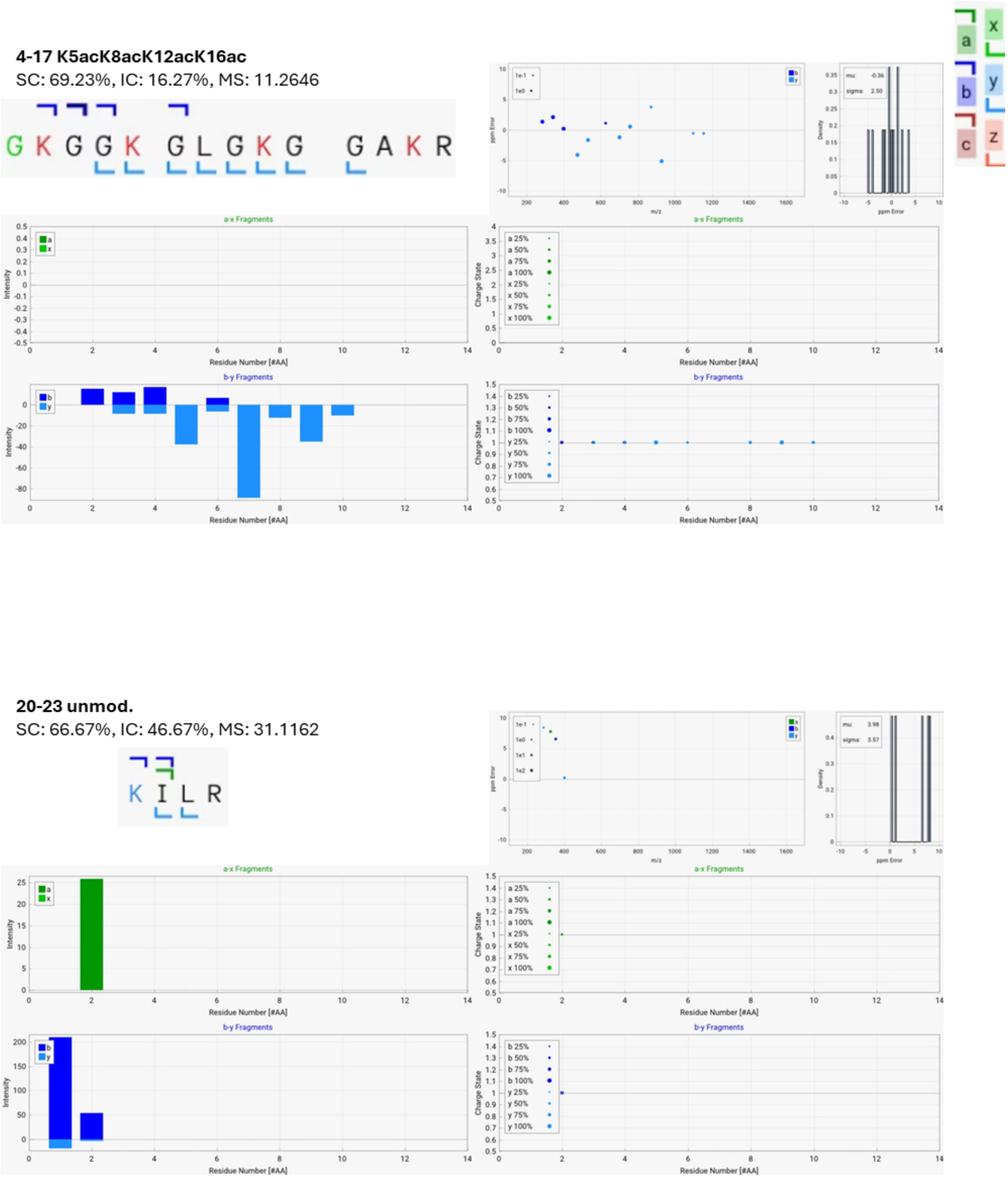

Figure S61. Replicate 1 bottom-up fragmentation of *A. cervicornis* H4 4-17 and 20-23 PTM patterns with ±10 ppm error: 4-17 K5acK8acK12acK16ac and 20-23 unmodified.

20-23 K20me1
SC: 100.00%, IC: 2.20%, MS: 2.1959

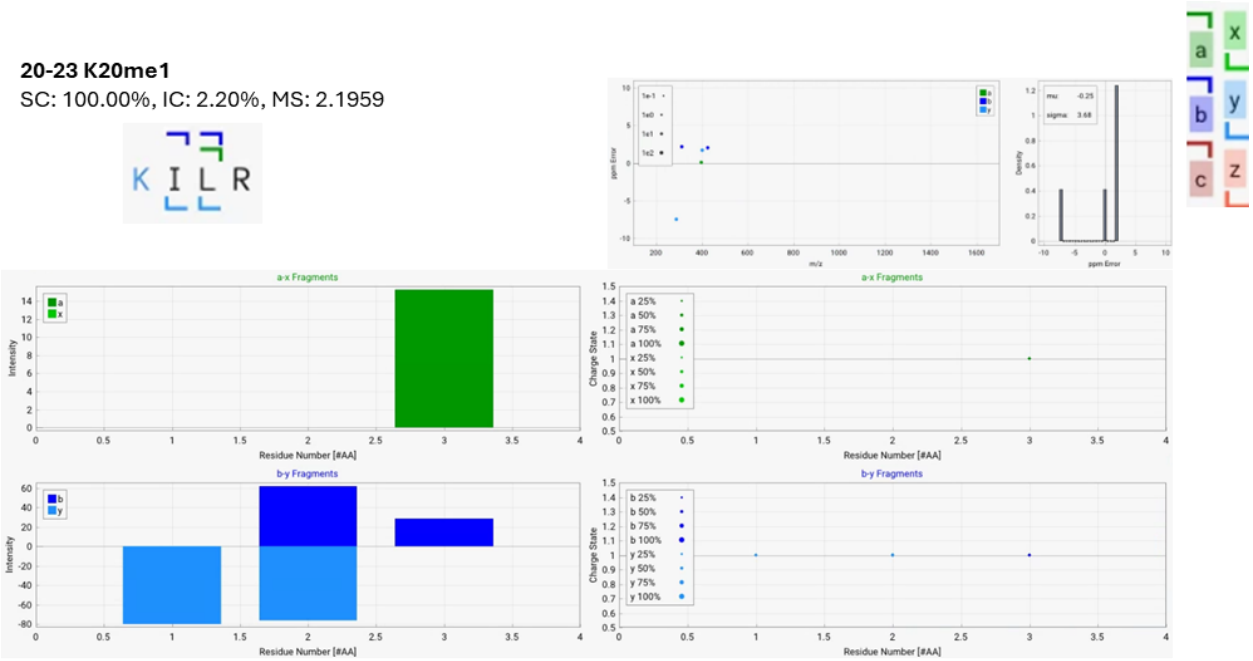

20-23 K20me2
SC: 66.67%, IC: 18.04%, MS: 12.0262

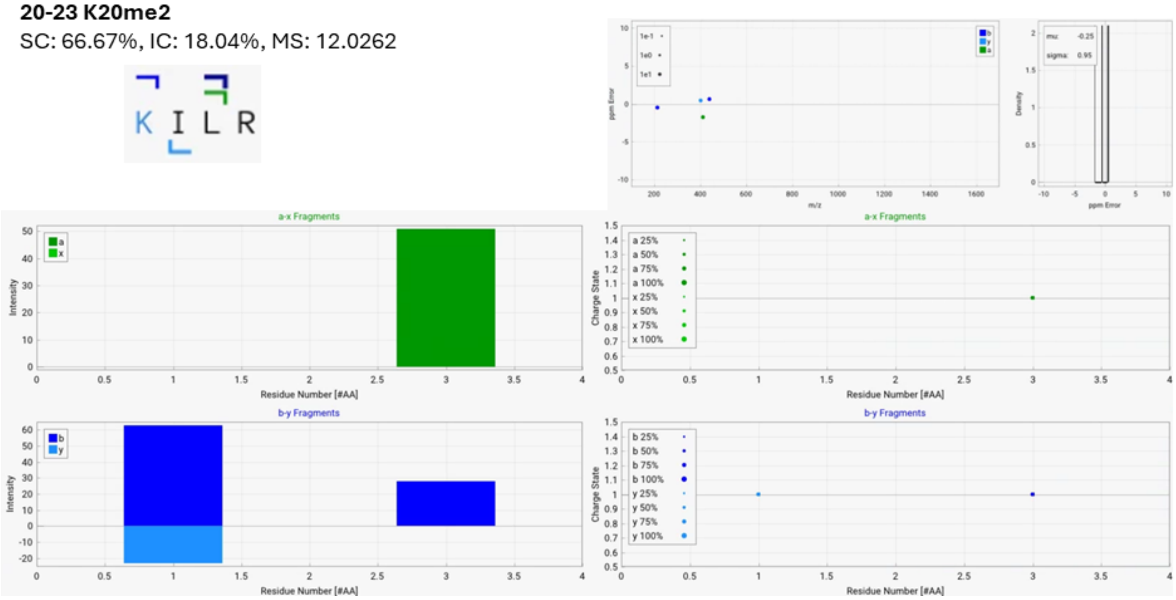

Figure S62. Replicate 1 bottom-up fragmentation of *A. cervicornis* H4 4-17 and 20-23 PTM patterns with ±10 ppm error: 20-23 K20me$_1$ and 20-23 K20me$_2$.

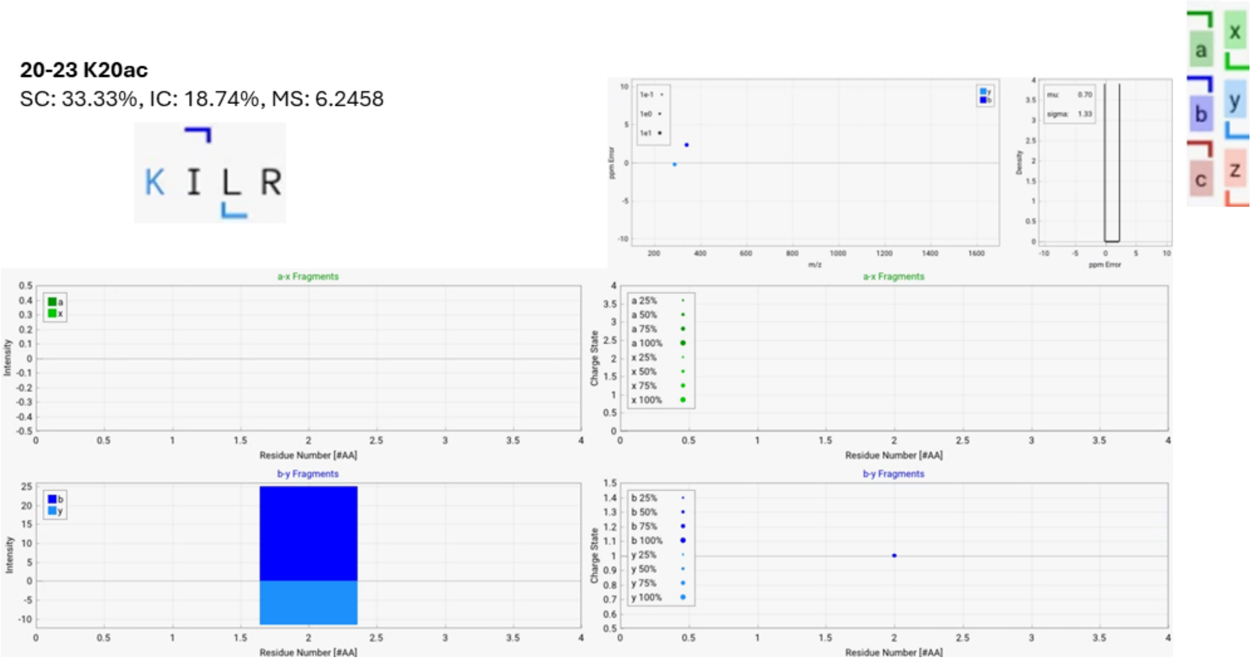

Figure S63. Replicate 1 bottom-up fragmentation of *A. cervicornis* H4 4-17 and 20-23 PTM patterns with ±10 ppm error: 20-23 K20ac.

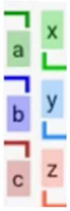

4-17 unmod.

SC: 92.31% IC: 27.15% MS: 25.0625

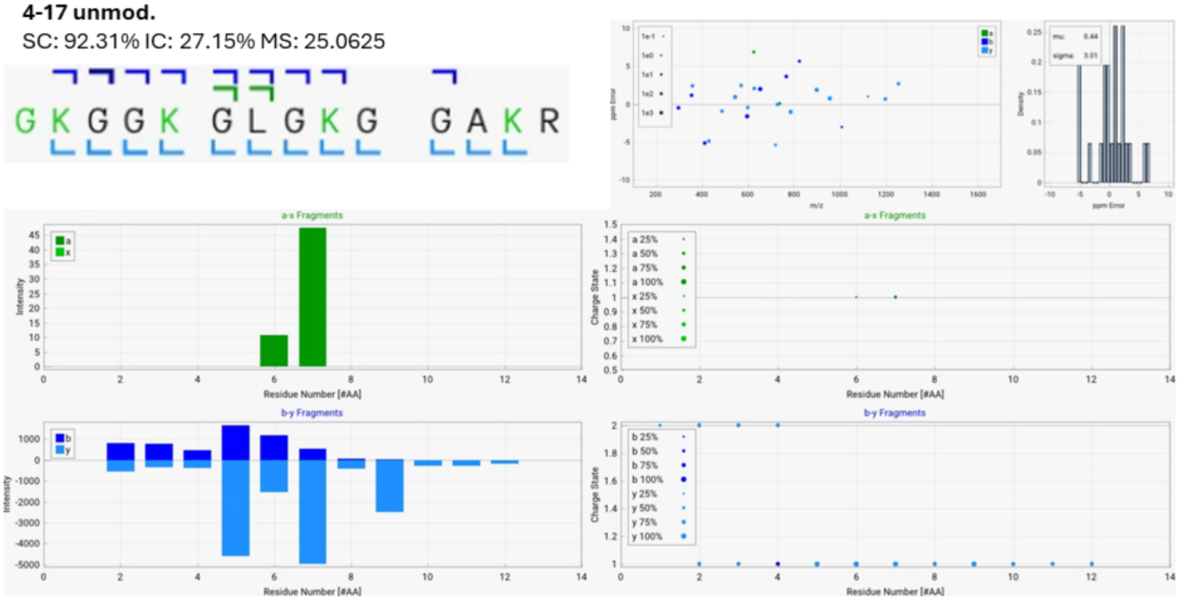

4-17 K12ac

SC: 92.31% IC: 24.37% MS: 22.4943

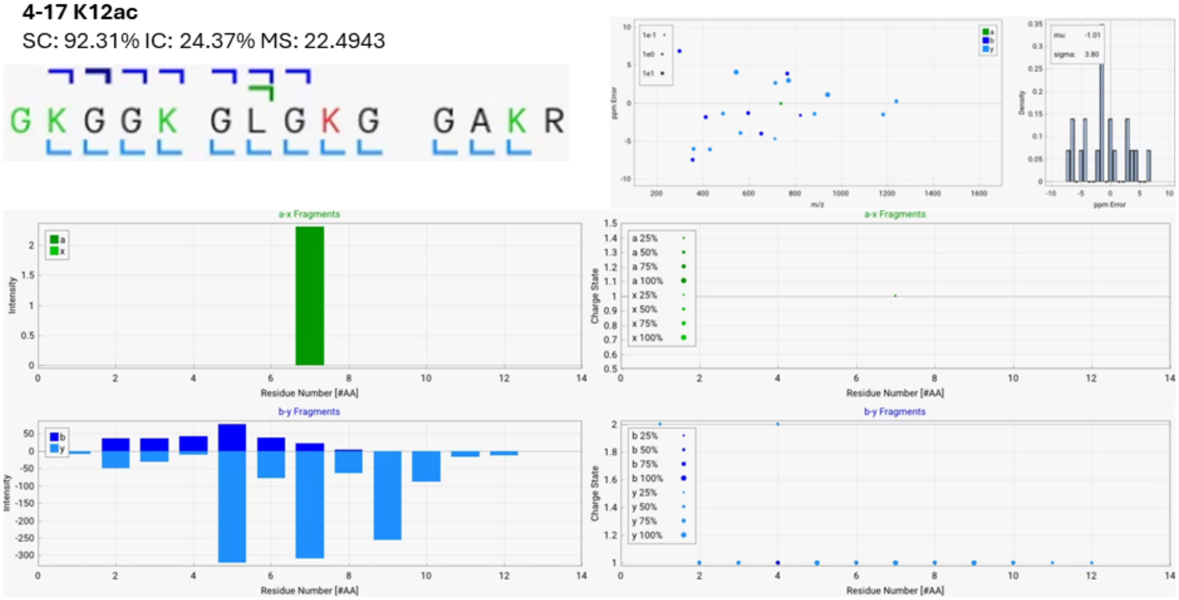

Figure S64. Replicate 2 bottom-up fragmentation of *A. cervicornis* H4 4-17 and 20-23 PTM patterns with ±10 ppm error: 4-17 unmodified and 4-17 K12ac.

4-17 K16ac

SC: 84.62% IC: 24.10% MS: 20.3944

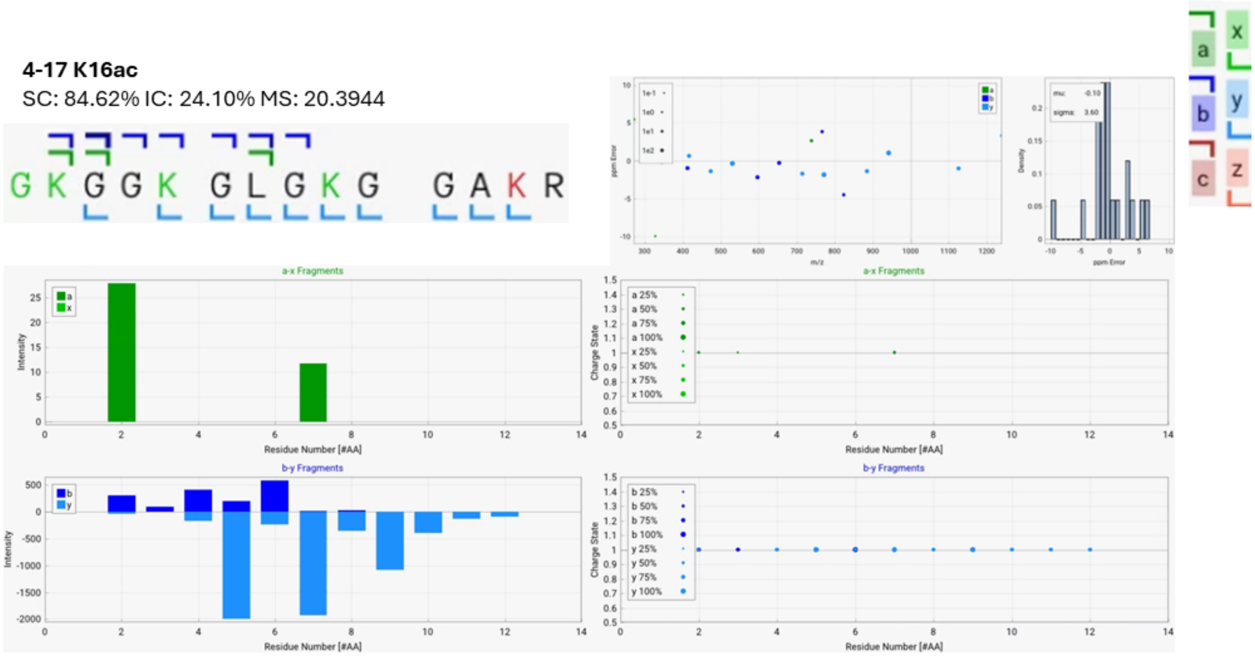

4-17 K5acK16ac

SC: 64.54%, IC: 7.46%, MS: 4.5934

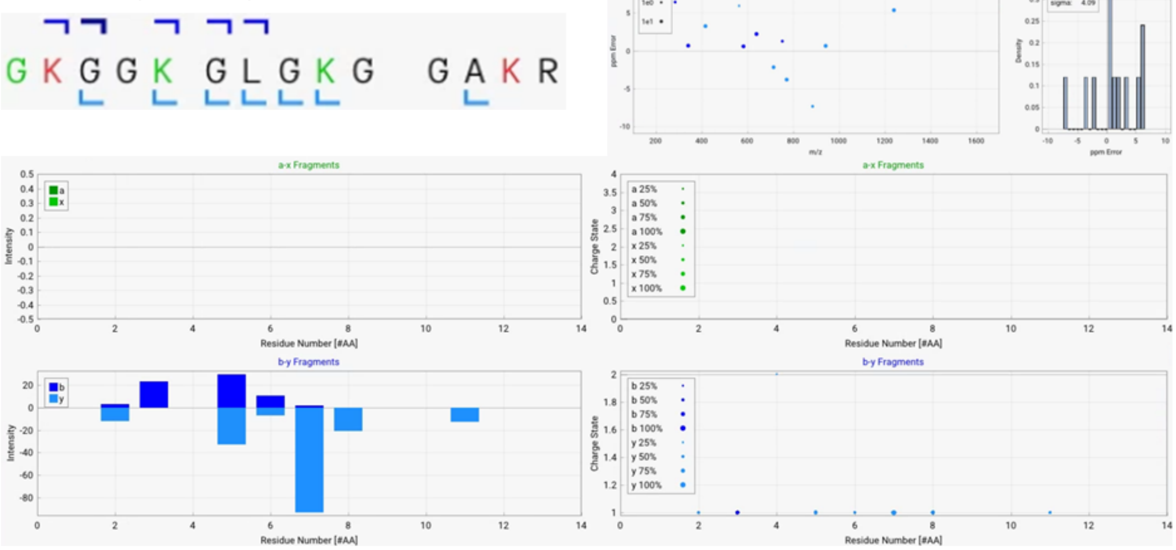

Figure S65. Replicate 2 bottom-up fragmentation of *A. cervicornis* H4 4-17 and 20-23 PTM patterns with ±10 ppm error: 4-17 K16ac and 4-17 K5acK16ac.

4-17 K8acK16ac

SC: 61.54%, IC: 8.11%, MS: 4.9934

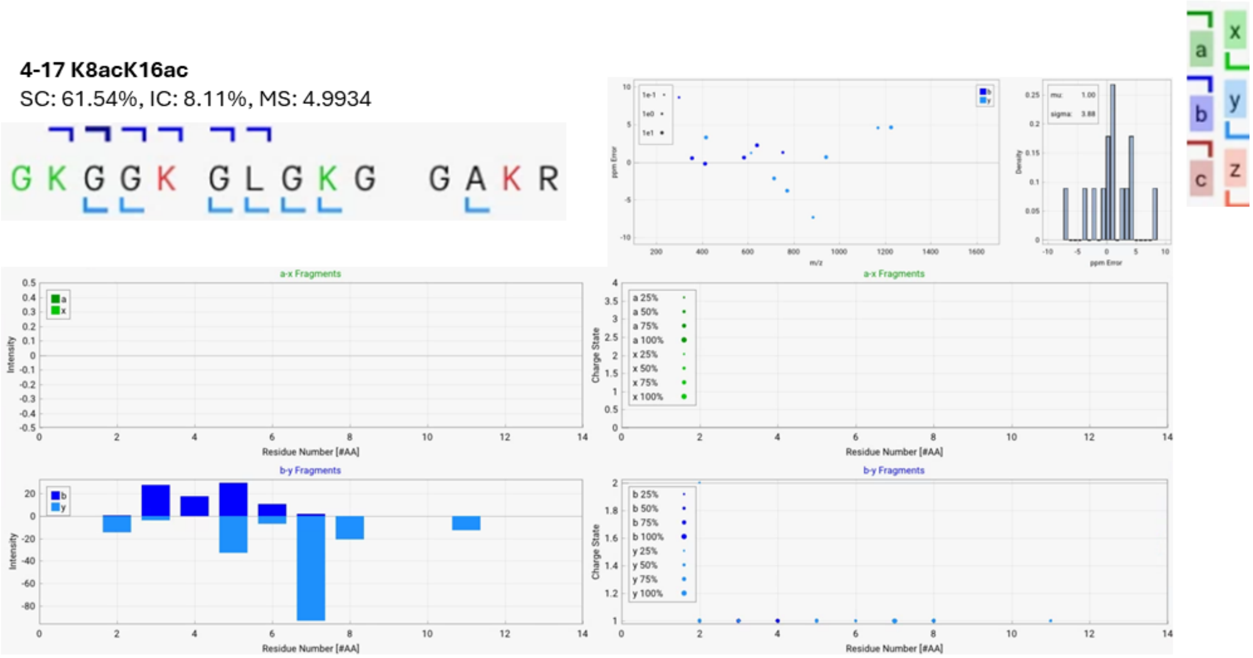

4-17 K12acK16ac

SC: 46.15%, IC: 5.56%, MS: 2.5666

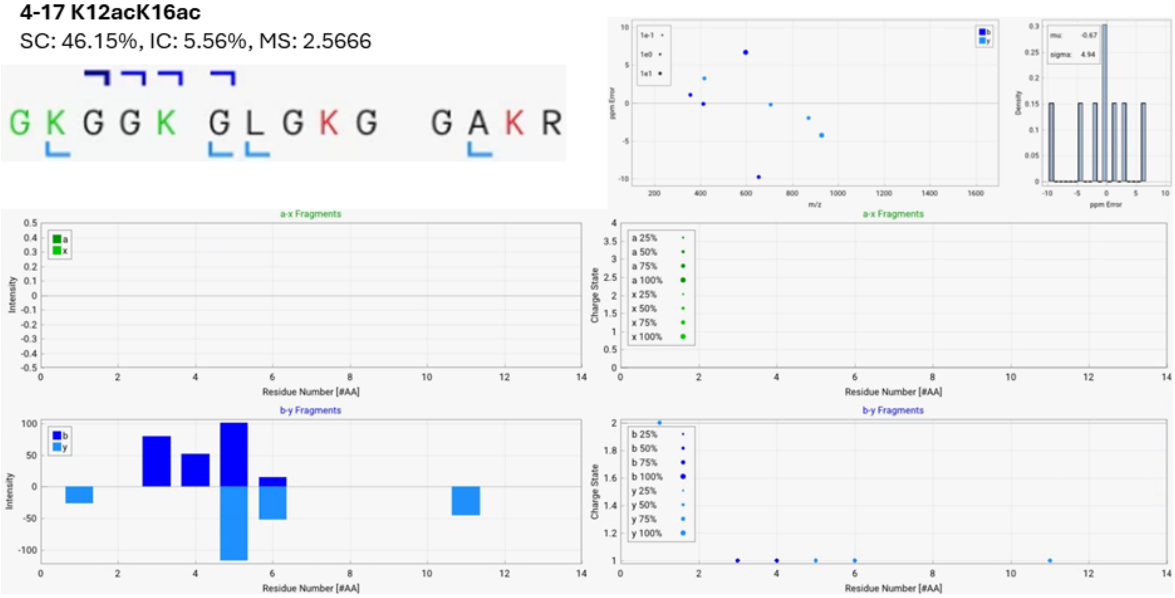

Figure S66. Replicate 2 bottom-up fragmentation of *A. cervicornis* H4 4-17 and 20-23 PTM patterns with ±10 ppm error: 4-17 K8acK16ac and 4-17 K12acK16ac.

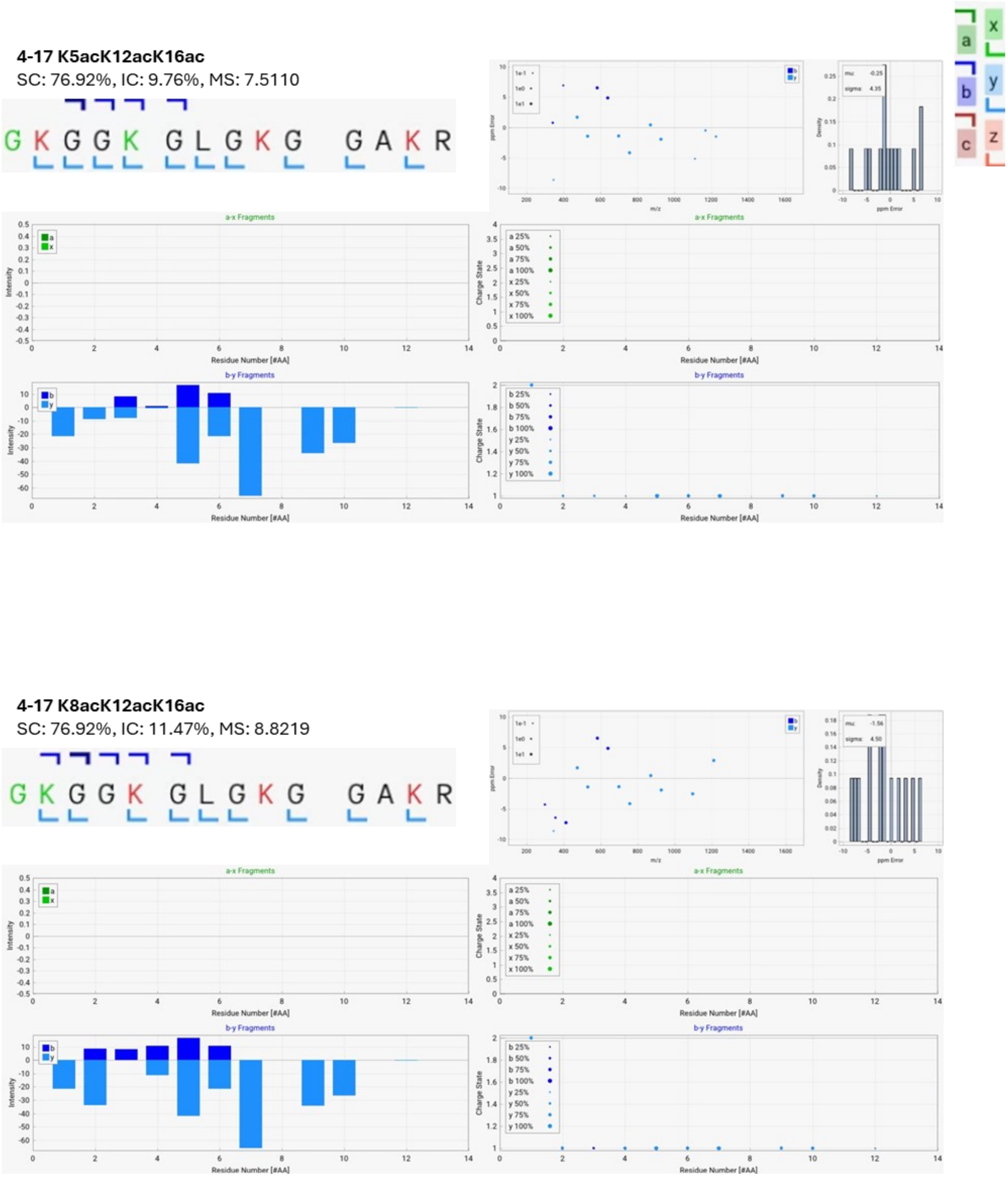

Figure S67. Replicate 2 bottom-up fragmentation of *A. cervicornis* H4 4-17 and 20-23 PTM patterns with ±10 ppm error: 4-17 K5acK12acK16ac and 4-17 K8acK12acK16ac.

4-17 K5acK8acK12acK16ac
SC: 84.62%, IC: 6.06%, MS: 5.1247

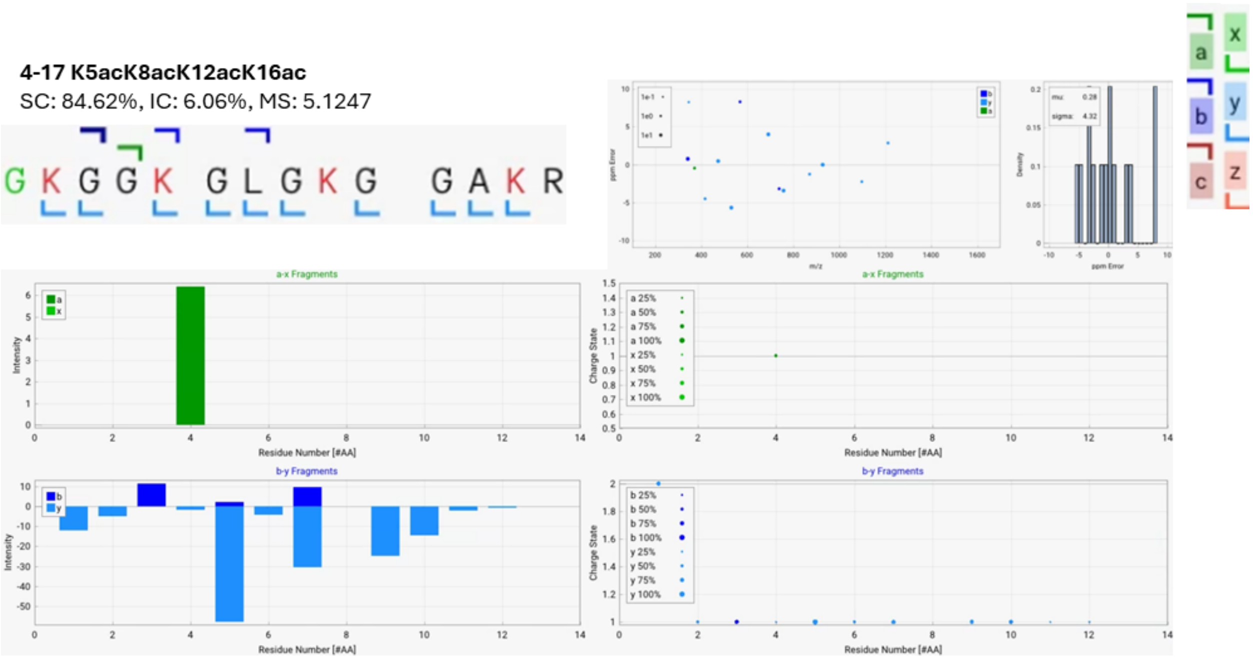

20-23 unmod.
SC: 100.00%, IC: 47.67%, MS: 47.6743

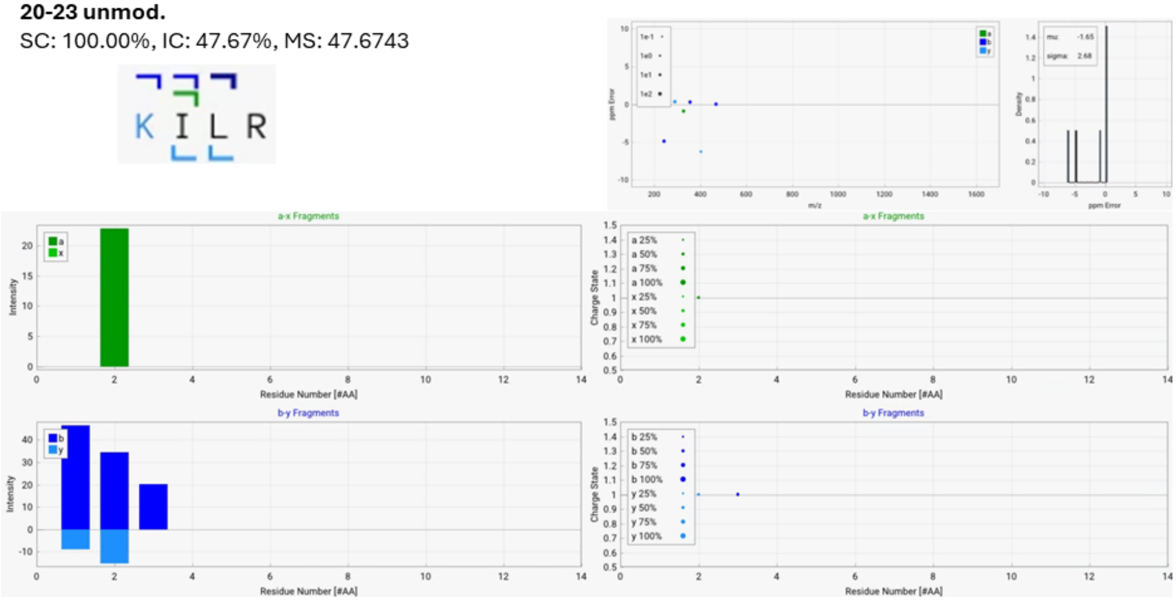

Figure S68. Replicate 2 bottom-up fragmentation of *A. cervicornis* H4 4-17 and 20-23 PTM patterns with ±10 ppm error: 4-17 K5acK8acK12acK16ac and 20-23 unmodified.

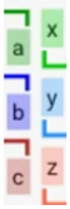

20-23 K20me1

SC: 100.00%, IC: 0.63%, MS: 0.6348

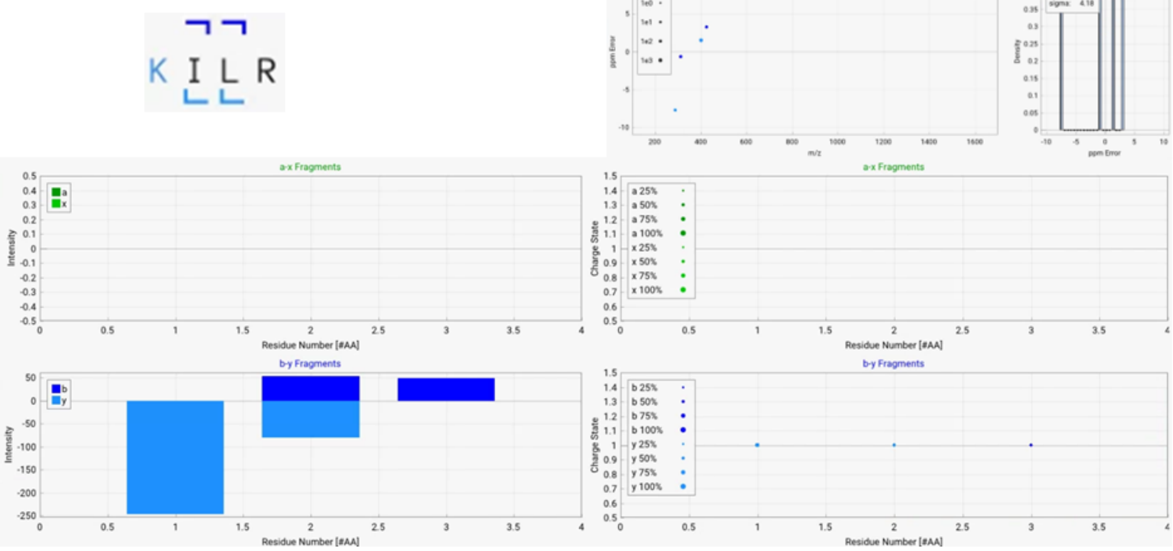

20-23 K20me2

SC: 100.00%, IC: 27.12%, MS: 27.1231

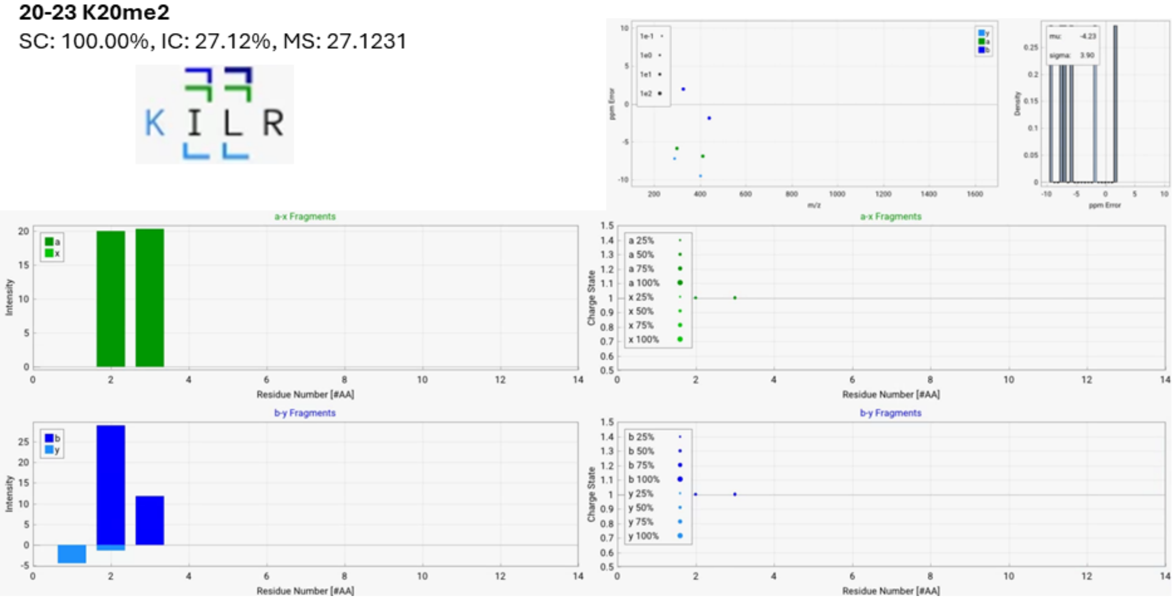

Figure S69. Replicate 2 bottom-up fragmentation of *A. cervicornis* H4 4-17 and 20-23 PTM patterns with ±10 ppm error: 20-23 K20me$_1$ and 20-23 K20me$_2$.

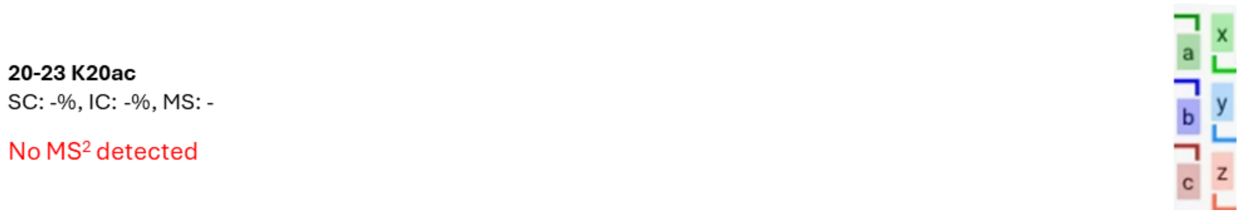

Figure S70. Replicate 2 bottom-up fragmentation of *A. cervicornis* H4 4-17 and 20-23 PTM patterns with ±10 ppm error: 20-23 K20ac.

4-17 unmod.

SC: 92.31% IC: 32.04% MS: 29.5798

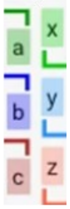

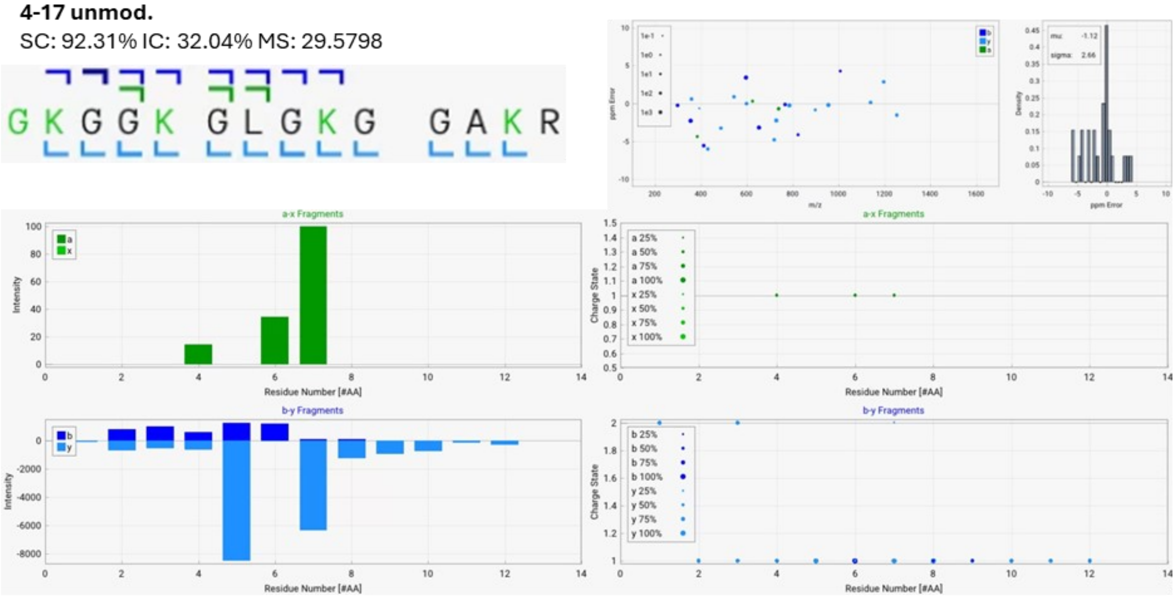

4-17 K12ac

SC: 92.31% IC: 20.87% MS: 19.2645

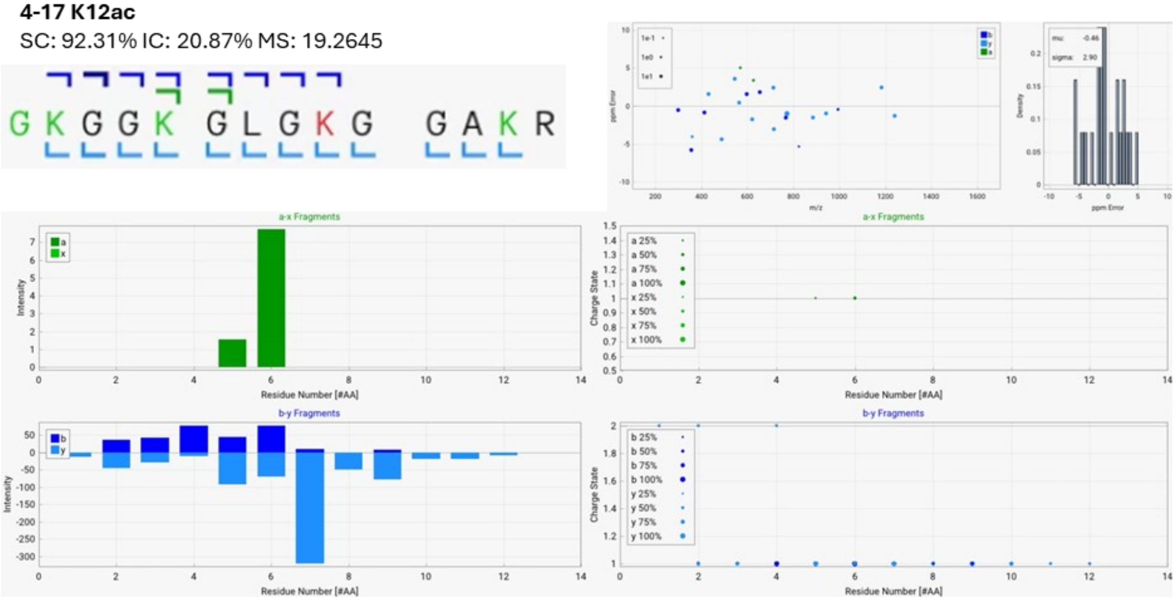

Figure S71. Replicate 3 bottom-up fragmentation of *A. cervicornis* H4 4-17 and 20-23 PTM patterns with ±10 ppm error: 4-17 unmodified and 4-17 K12ac.

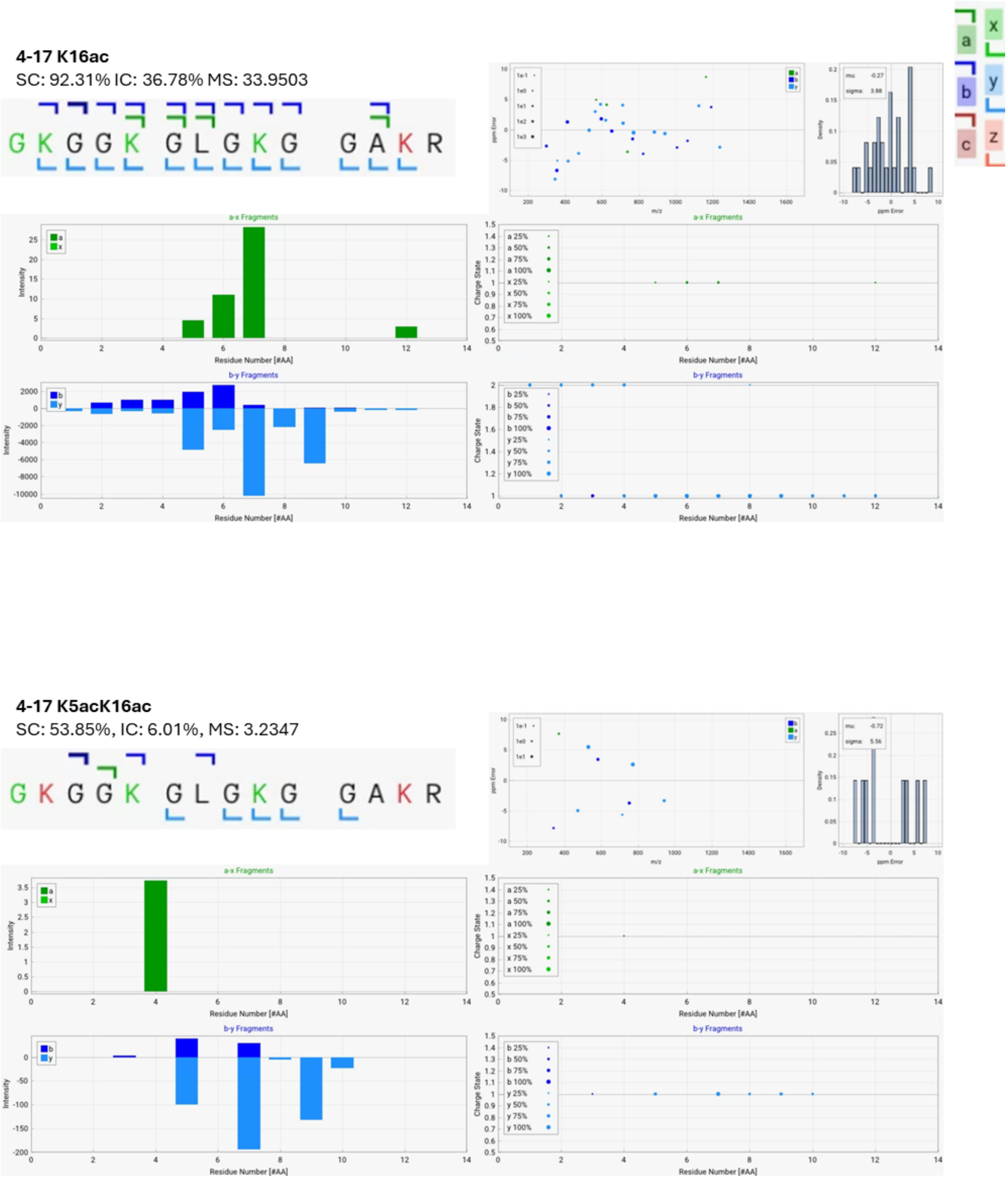

Figure S72. Replicate 3 bottom-up fragmentation of *A. cervicornis* H4 4-17 and 20-23 PTM patterns with ±10 ppm error: 4-17 K16ac and 4-17 K5acK16ac.

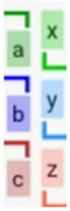

4-17 K8acK16ac

SC: 61.54%, IC: 7.77%, MS: 4.7824

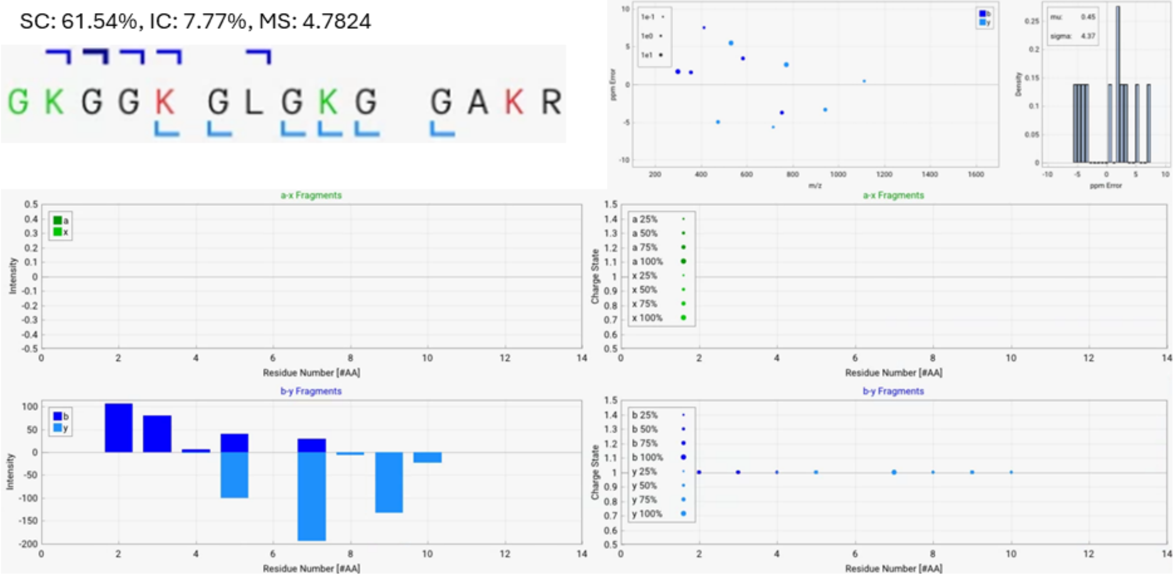

4-17 K12acK16ac

SC: 61.54%, IC: 9.29%, MS: 5.7168

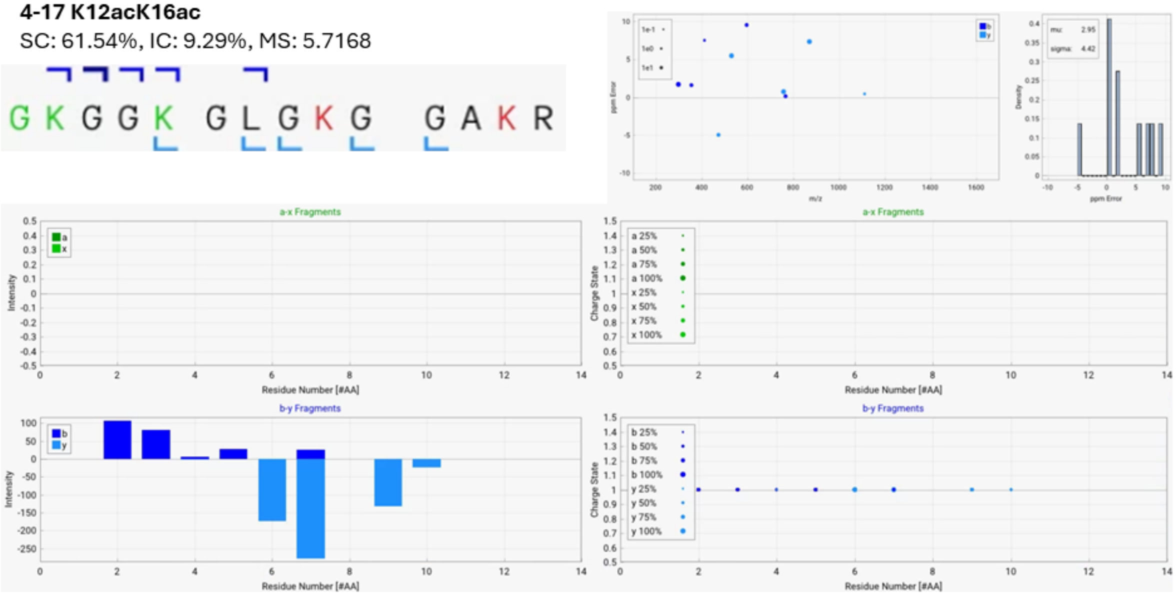

Figure S73. Replicate 3 bottom-up fragmentation of *A. cervicornis* H4 4-17 and 20-23 PTM patterns with ±10 ppm error: 4-17 K8acK16ac and 4-17 K12acK16ac.

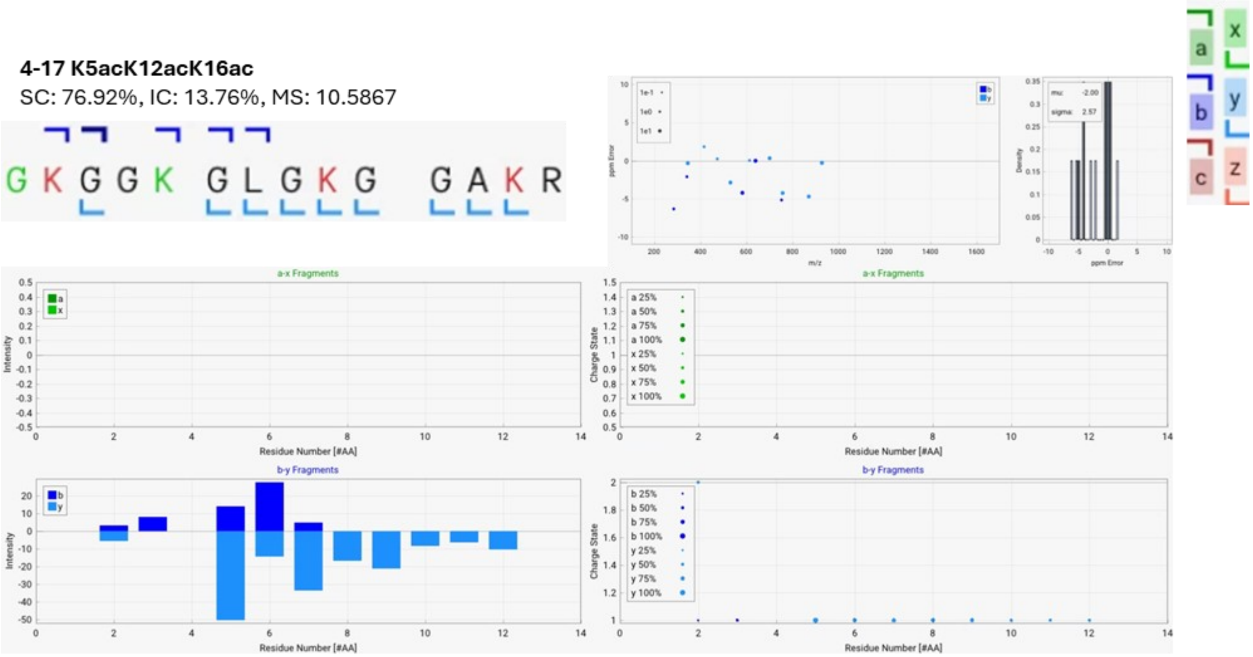

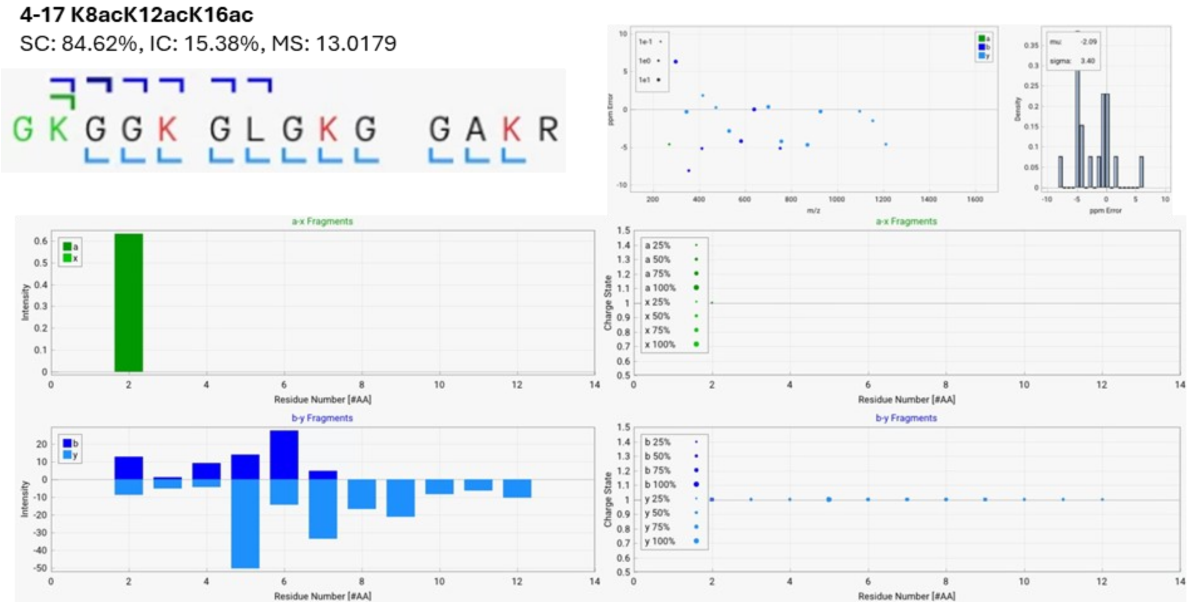

Figure S74. Replicate 3 bottom-up fragmentation of *A. cervicornis* H4 4-17 and 20-23 PTM patterns with ±10 ppm error: 4-17 K5acK12acK16ac and 4-17 K8acK12acK16ac.

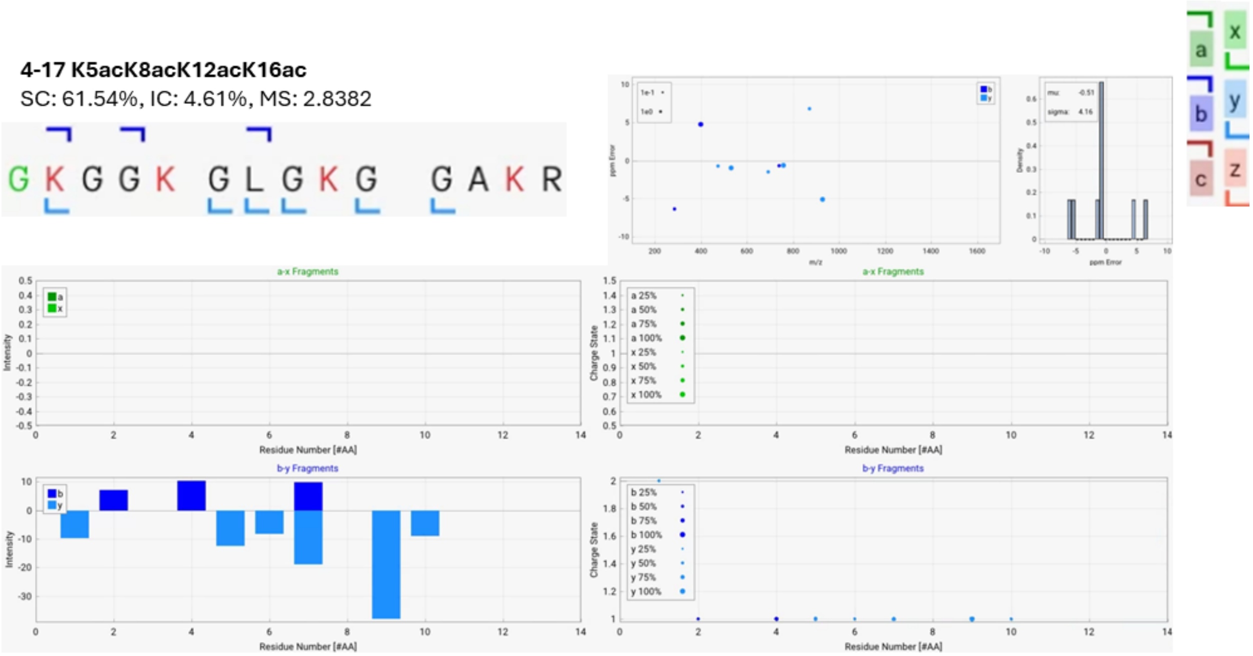

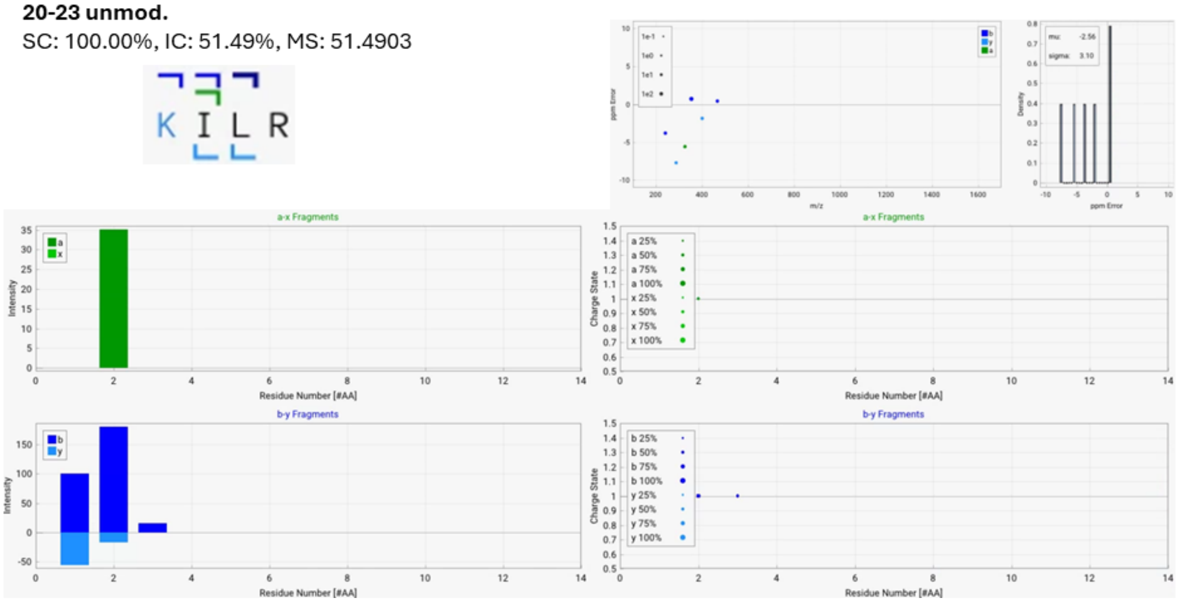

Figure S75. Replicate 3 bottom-up fragmentation of *A. cervicornis* H4 4-17 and 20-23 PTM patterns with ±10 ppm error: 4-17 K5acK8acK12acK16ac and 20-23 unmodified.

20-23 K20me1

SC: 100.00%, IC: 1.05%, MS: 1.0503

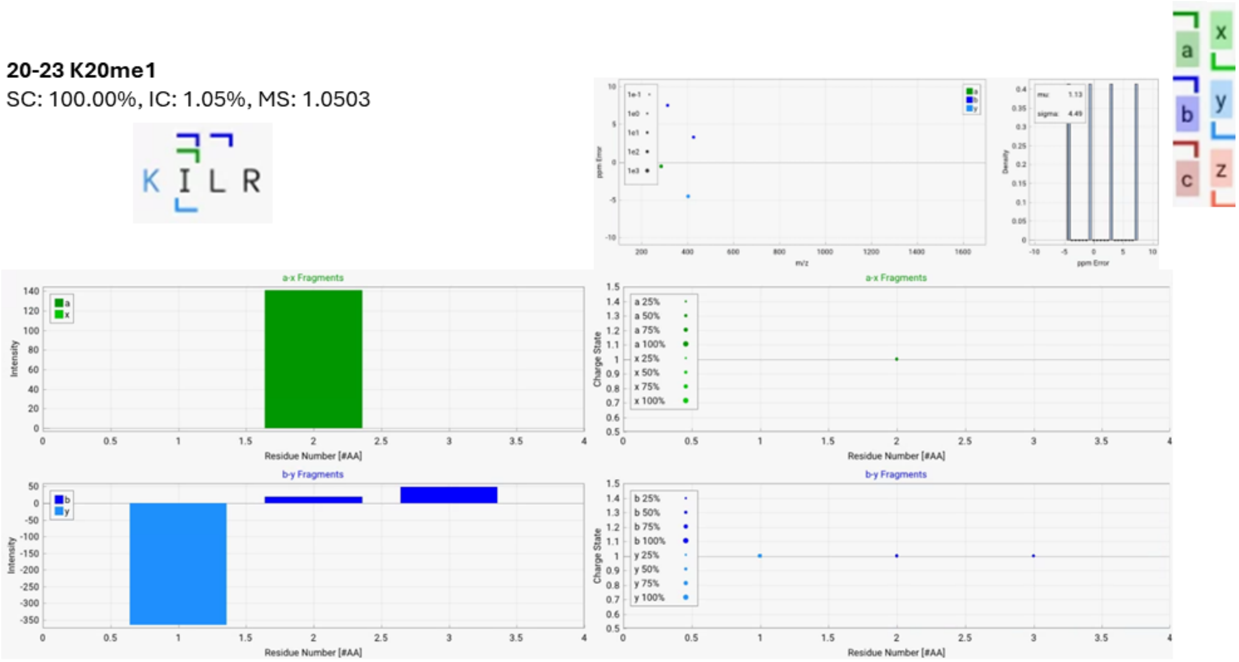

20-23 K20me2

SC: 100.00%, IC: 21.31%, MS: 21.3102

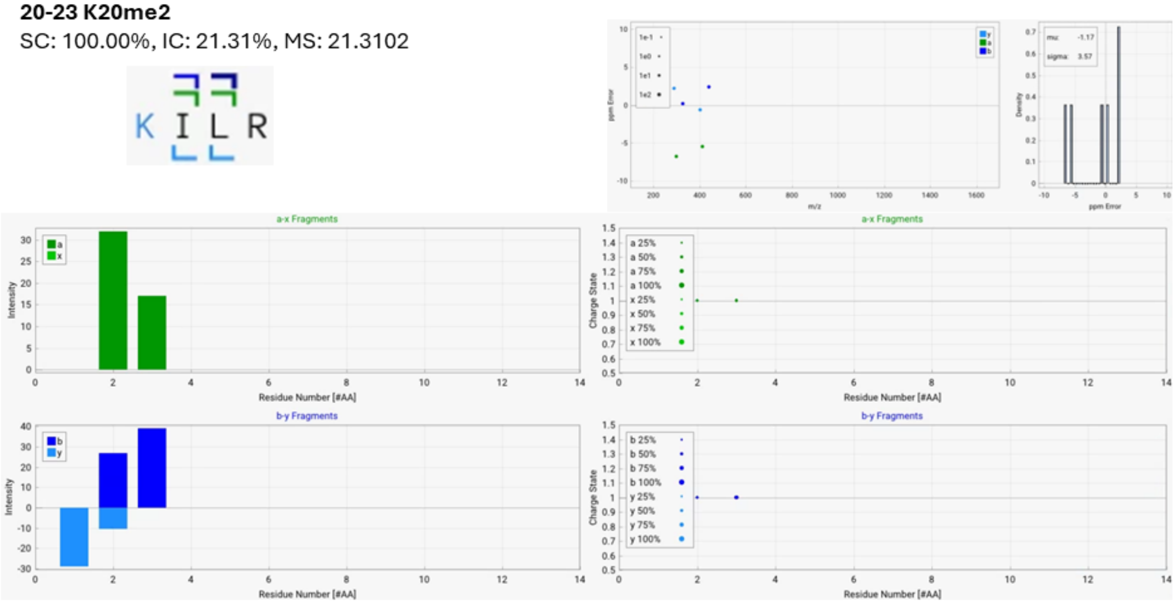

Figure S76. Replicate 3 bottom-up fragmentation of *A. cervicornis* H4 4-17 and 20-23 PTM patterns with ±10 ppm error: 20-23 K20me$_1$ and 20-23 K20me$_2$.

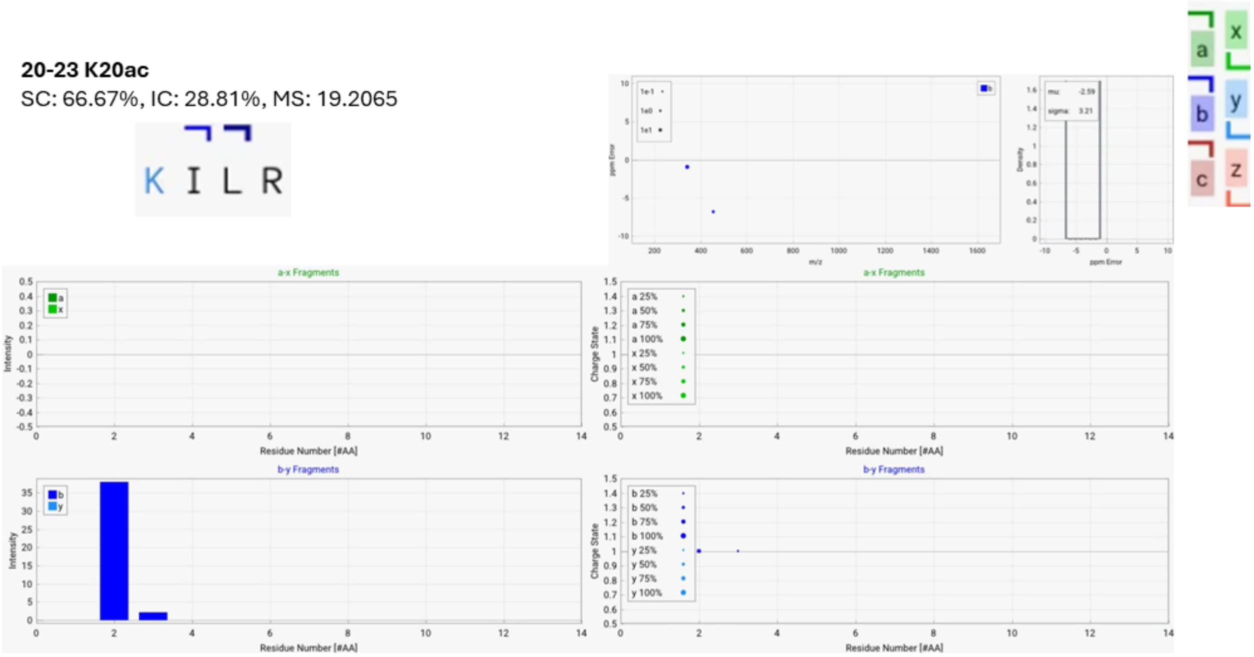

Figure S77. Replicate 3 bottom-up fragmentation of *A. cervicornis* H4 4-17 and 20-23 PTM patterns with ±10 ppm error: 20-23 K20ac.

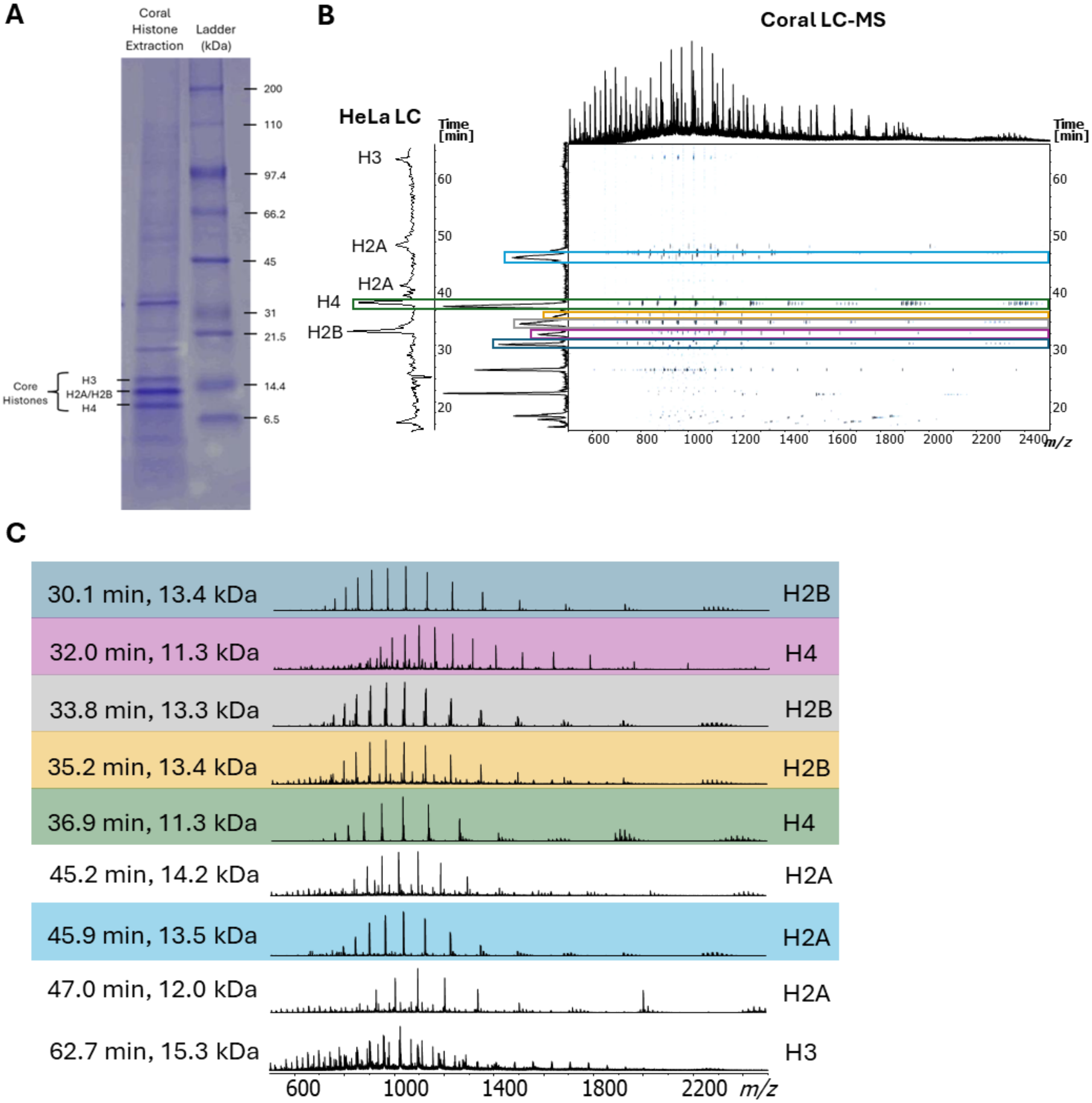

Figure S78. A. cervicornis histone extraction and online LC-MS. (A) SDS-PAGE, (B) 2D LC-MS with RT and *m/z* projections and HeLa (*H. sapiens*) chromatogram for comparison, and (C) extracted LC-MS for each observed histone-like mass with retention time, approximate masses, and histone families.

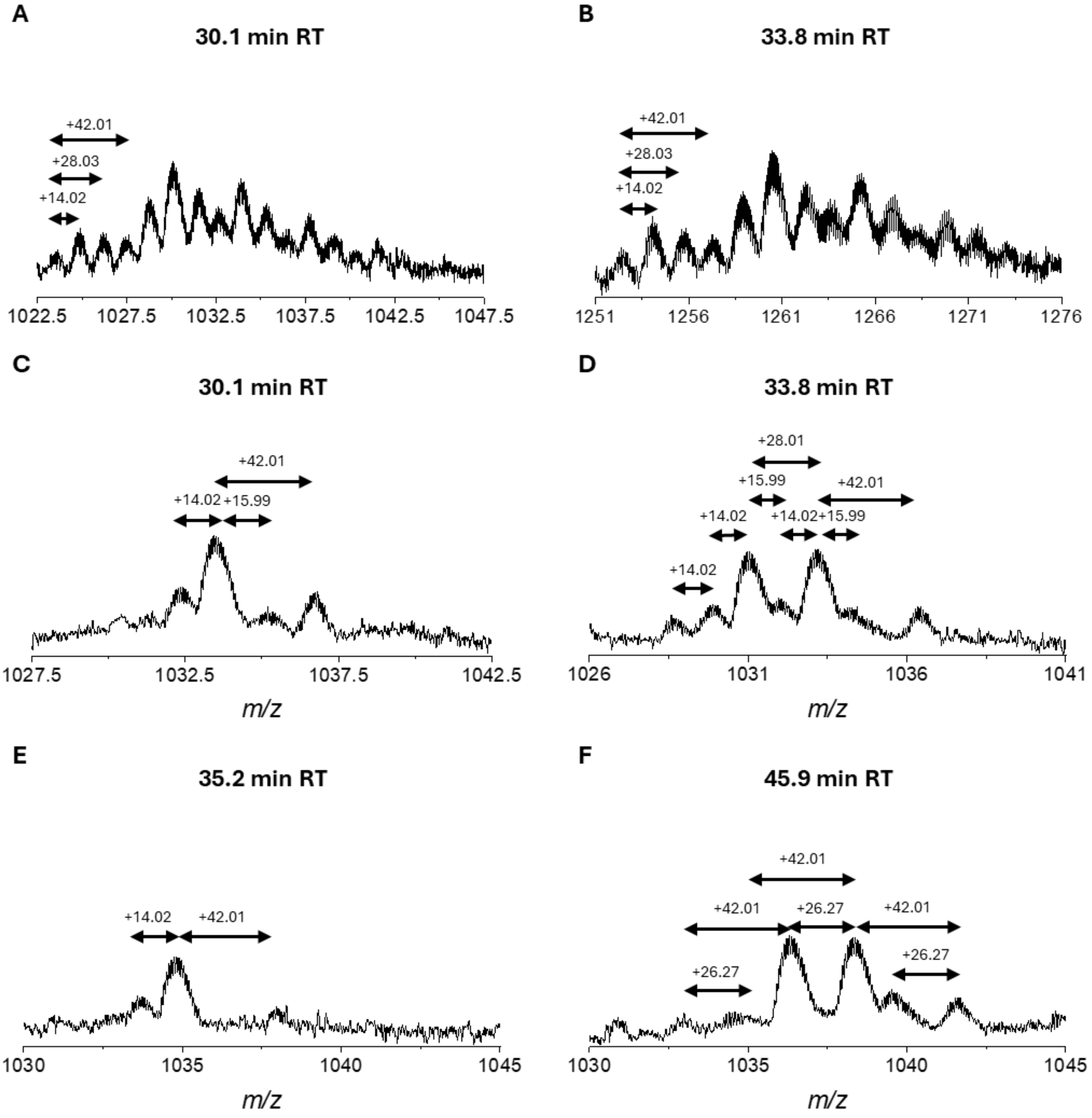

Figure S79. Observed mass shifts in H4 and H2A/H2B A. cervicornis histone fractions. (A) 32.0 min, (B) 36.9 min, (C) 30.1 min, (D) 33.8 min, (E) 35.2 min, and (F) 45.9 min RT fractions.

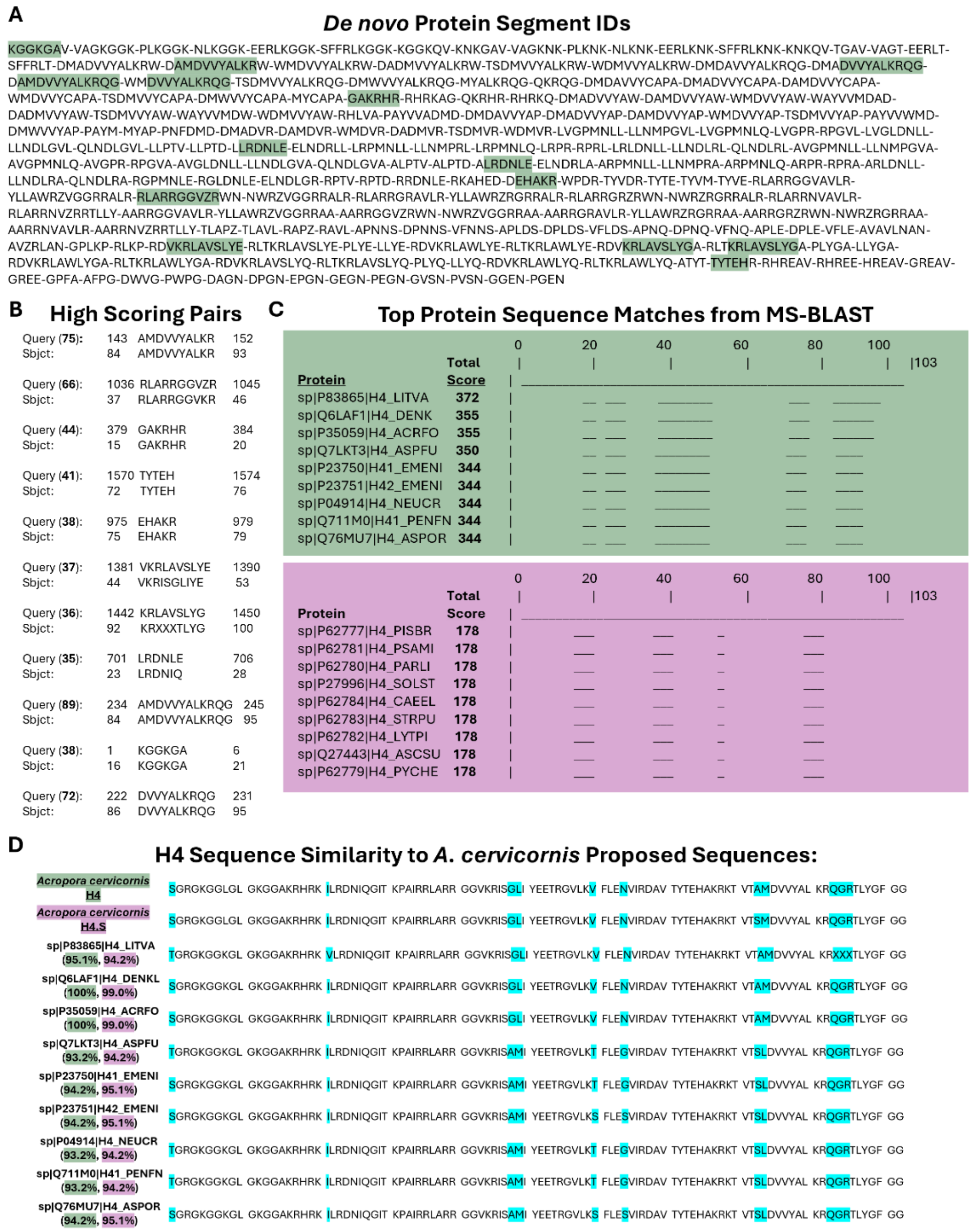

Figure S80. MS-BLAST results for A. cervicornis 32.0 and 36.9 min (H4 & H4.S) fractions. (A) Detected *de novo* protein sequence segments from OmniScape with high-scoring pairs highlighted in green. (B) High-scoring pairs from top 9 protein matches. (C) Top 9 matching proteins found in the SwissProt database with total scores and mapped protein segments. (D) *A. cervicornis* H4 sequence compared to top 9 protein matches with sequence similarity (%) and all vs. all comparison of sequences with common mutation points highlighted in cyan.

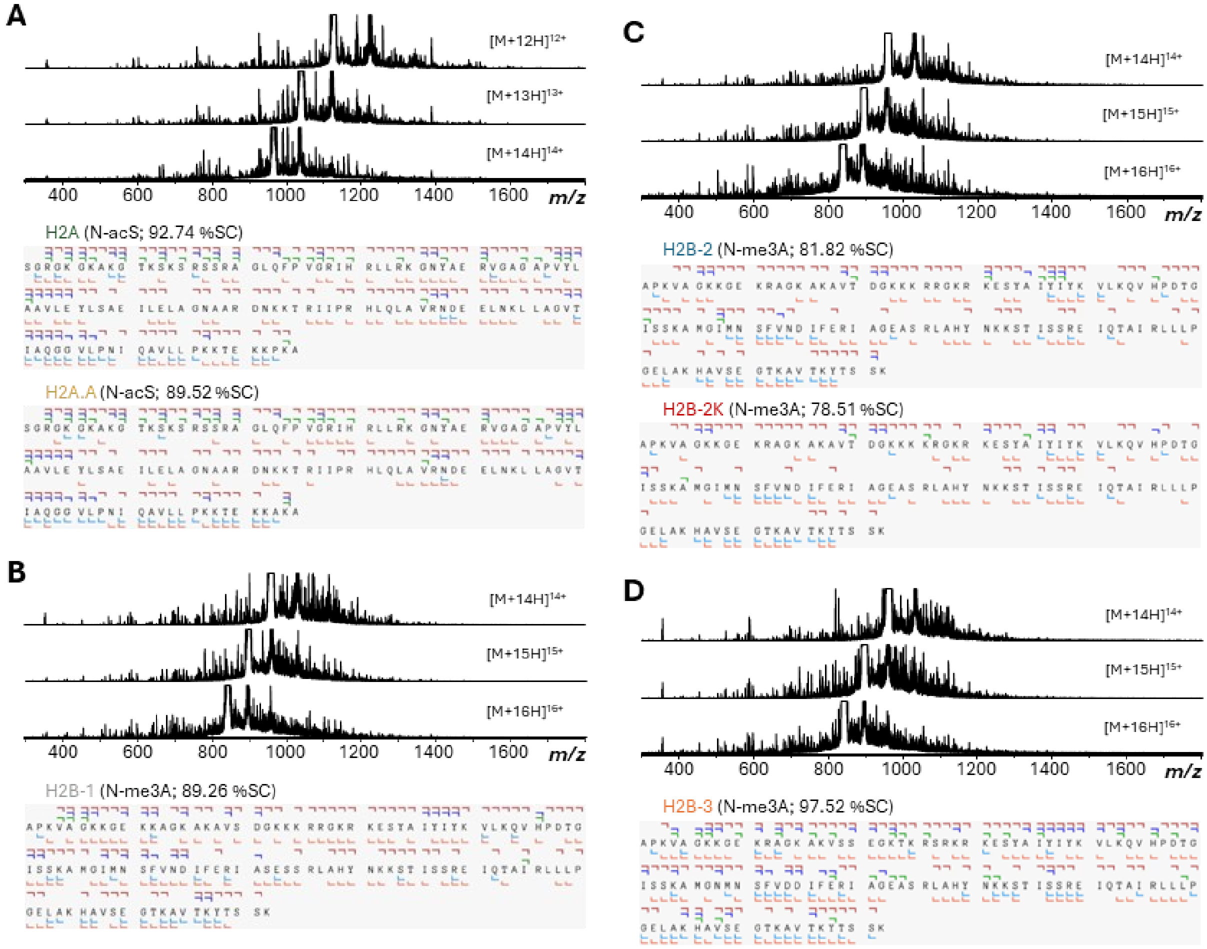

Figure S81. Highest intensity charge state ECD-MS/MS and highest intensity proteoforms sequenced for H2A/H2B fractions. (A) H2A 45.9 min RT band, (B) H2B 30.1 min RT band, (C) H2B 33.8 min RT band, (D) H2B 35.2 min RT band.

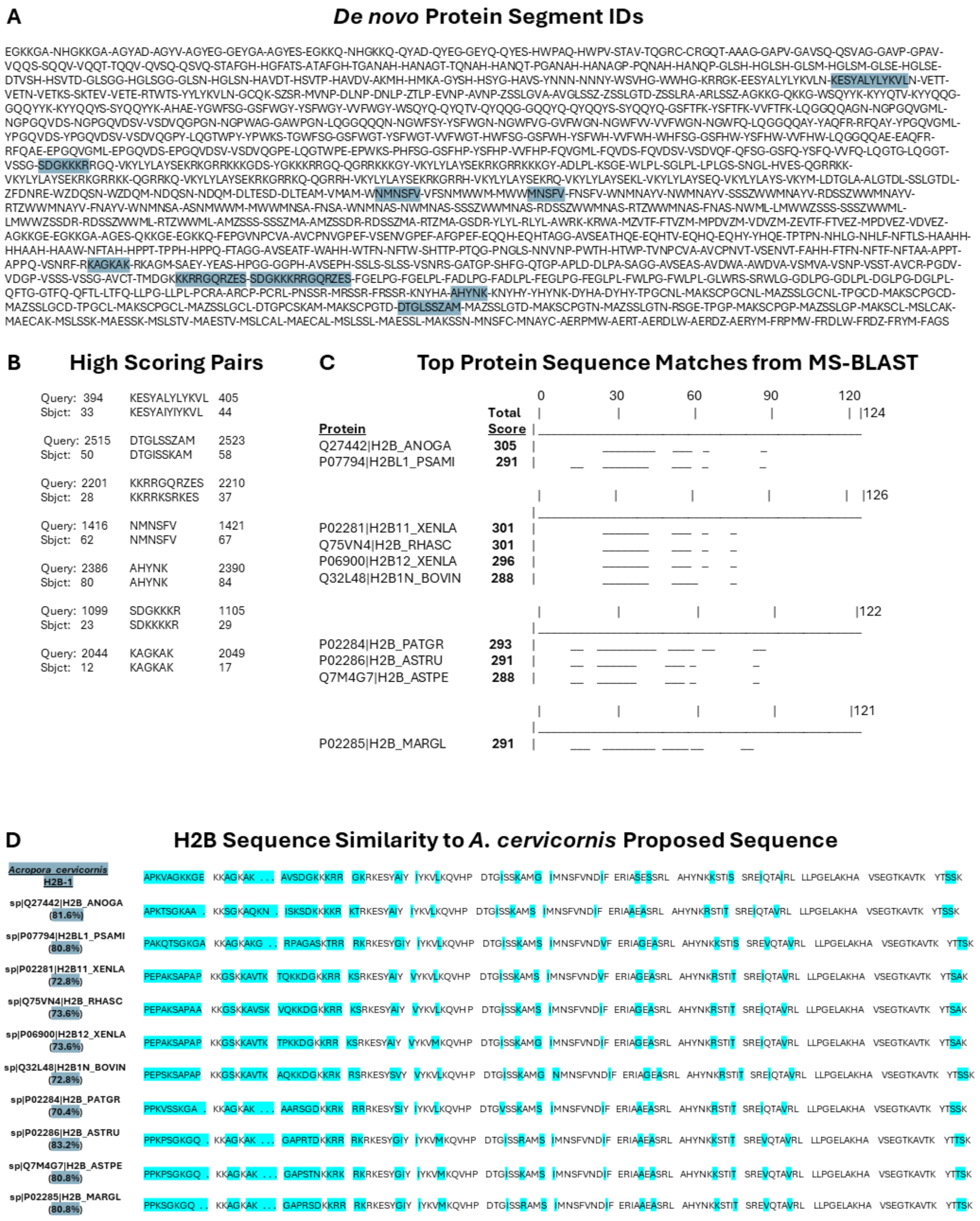

Figure S82. MS-BLAST results for A. cervicornis 30.1 min (H2B-1) fraction. (A) Detected *de novo* protein sequence segments from OmniScape with high scoring pairs highlighted in dark blue. (B) High scoring pairs from top 10 protein matches. (C) Top 10 matching proteins found in Swissprot database with total scores and mapped protein segments. (D) *A. cervicornis* H2B-1 sequence compared to top 10 protein matches with sequence similarity (%) and all vs. all comparison of sequences with common mutation points highlighted in cyan.

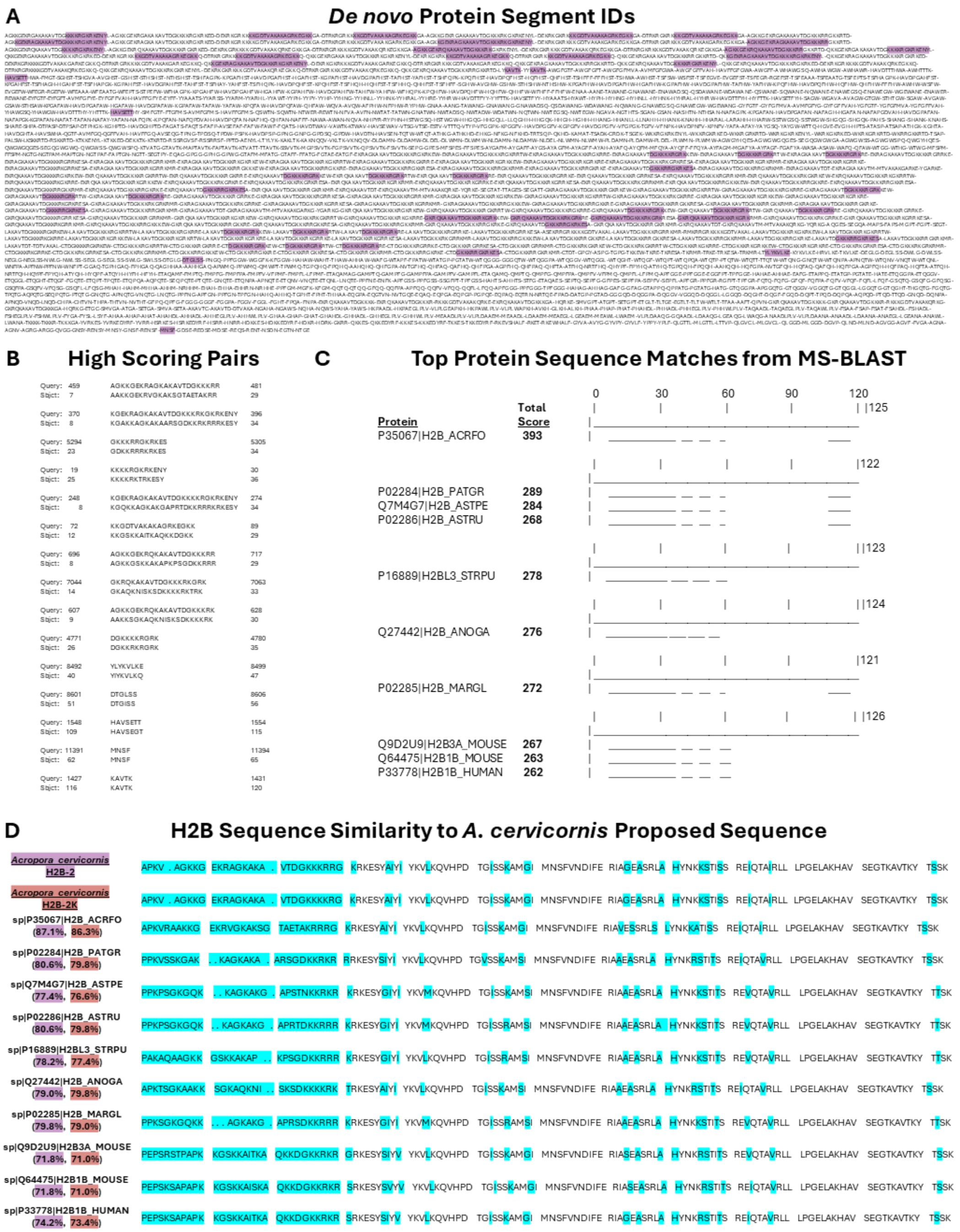

Figure S83. MS-BLAST results for A. cervicornis 33.8 min (H2B-2/H2B-2K) fraction. (A) Detected *de novo* protein sequence segments from OmniScape with high scoring pairs highlighted in purple. (B) High scoring pairs from top 10 protein matches. (C) Top 10 matching proteins found in Swissprot database with total scores and mapped protein segments. (D) *A. cervicornis* H2B-2 and H2B-2K sequences compared to top 10 protein matches with sequence similarity (%) and all vs. all comparison of sequences with common mutation points highlighted in cyan.

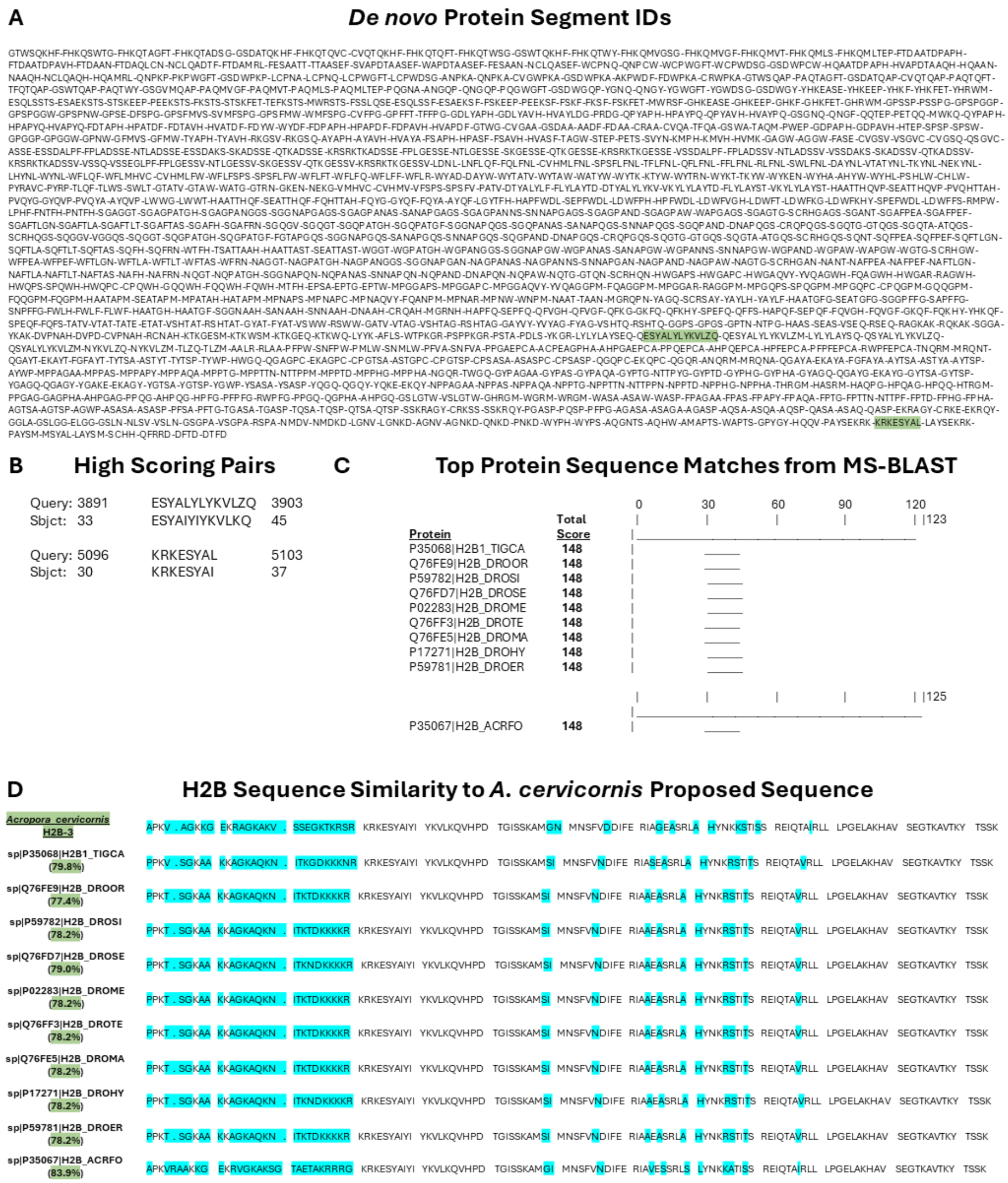

Figure S84. MS-BLAST results for A. cervicornis 35.2 min (H2B-3) fraction. (A) Detected *de novo* protein sequence segments from OmniScape with high scoring pairs highlighted in light green. (B) High scoring pairs from top 10 protein matches. (C) Top 10 matching proteins found in Swissprot database with total scores and mapped protein segments. (D) *A. cervicornis* H2B-3 sequence compared to top 10 protein matches with sequence similarity (%) and all vs. all comparison of sequences with common mutation points highlighted in cyan.

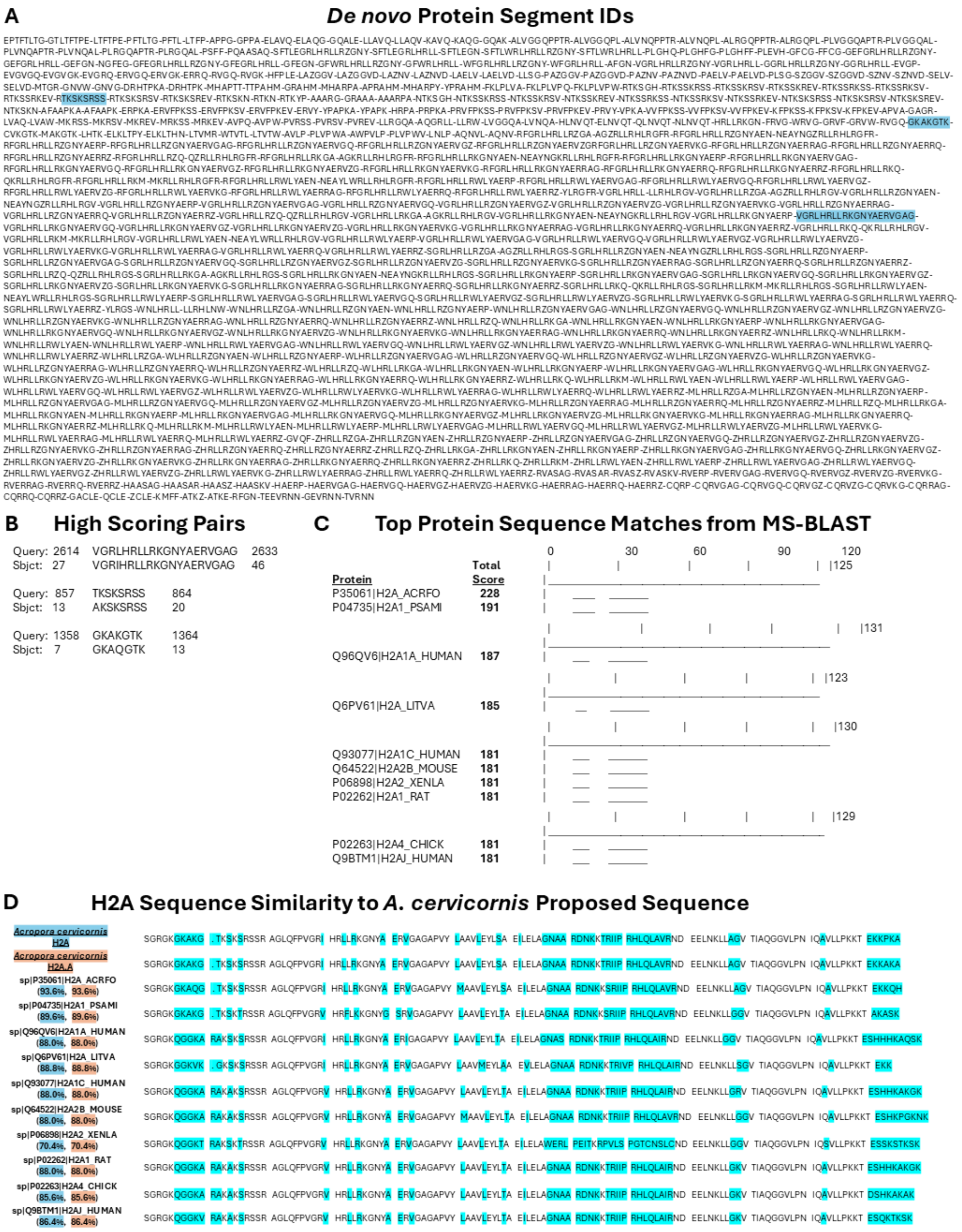

Figure S85. MS-BLAST results for A. cervicornis 45.9 min (H2A/H2A.A) fraction. (A) Detected *de novo* protein sequence segments from OmniScape with high scoring pairs highlighted in light blue. (B) High scoring pairs from top 10 protein matches. (C) Top 10 matching proteins found in Swissprot database with total scores and mapped protein segments. (D) *A. cervicornis* H2Aand H2A.A sequences compared to top 10 protein matches with sequence similarity (%) and all vs. all comparison of sequences with common mutation points highlighted in cyan.

Protein Existence Score Key:

1. Protein uncertain
2. Protein predicted
3. Protein inferred from homology
4. Experimental evidence at transcript level
5. Experimental evidence at protein level

A. cervicornis H4 proposed sequence

H4 Sequences - % similarity to proposed sequence — mutation

%	Sequence	H4 sequence	Prot. Score
	**Acropora cervicornis Proposed Sequence**	SGRGKGGKGL GKGGAKRHRK ILRDNIQGIT KPAIRRLARR GGVKRISGLI YEETRGVLKV FLENVIRDAV TYTEHAKRKT VTAMDVVYAL KRQGRTLYGF GG	
100%	*Acropora formosa* P35059	SGRGKGGKGL GKGGAKRHRK ILRDNIQGIT KPAIRRLARR GGVKRISGLI YEETRGVLKV FLENVIRDAV TYTEHAKRKT VTAMDVVYAL KRQGRTLYGF GG	3
100 %	*Dendro. klunz.* Q6LAF1	SGRGKGGKGL GKGGAKRHRK ILRDNIQGIT KPAIRRLARR GGVKRISGLI YEETRGVLKV FLENVIRDAV TYTEHAKRKT VTAMDVVYAL KRQGRTLYGF GG	3
95.1 %	*Penaeus vann.* P83865	TGRGKGGKGL GKGGAKRHRK VLRDNIQGIT KPAIRRLARR GGVKRISGLI YEETRGVLKV FLENVIRDAV TYTEHAKRKT VTAMDVVYAL KRXXXTLYGF GG	5
99.0 %	*Homo sapiens* P62805	SGRGKGGKGL GKGGAKRHRK VLRDNIQGIT KPAIRRLARR GGVKRISGLI YEETRGVLKV FLENVIRDAV TYTEHAKRKT VTAMDVVYAL KRQGRTLYGF GG	5
92.2 %	*Asper. fumigatus* Q7LKT3	TGRGKGGKGL GKGGAKRHRK ILRDNIQGIT KPAIRRLARR GGVKRISAMI YEETRGVLKT FLEGVIRDAV TYTEHAKRKT VTSLDVVYAL KRQGRTLYGF GG	3
93.2 %	*E. nidulans H4.1* P23750	SGRGKGGKGL GKGGAKRHRK ILRDNIQGIT KPAIRRLARR GGVKRISAMI YEETRGVLKT FLEGVIRDAV TYTEHAKRKT VTSLDVVYAL KRQGRTLYGF GG	3
93.2 %	*E. nidulans H4.2* P23751	SGRGKGGKGL GKGGAKRHRK ILRDNIQGIT KPAIRRLARR GGVKRISAMI YEETRGVLKS FLESVIRDAV TYTEHAKRKT VTSLDVVYAL KRQGRTLYGF GG	3
93.2 %	*N. crassa* P04914	TGRGKGGKGL GKGGAKRHRK ILRDNIQGIT KPAIRRLARR GGVKRISAMI YEETRGVLKT FLEGVIRDAV TYTEHAKRKT VTSLDVVYAL KRQGRTLYGF GG	3
92.2 %	*T. funiculosus* Q711M0	TGRGKGGKGL GKGGAKRHRK ILRDNIQGIT KPAIRRLARR GGVKRISAMI YEETRGVLKT FLEGVIRDAV TYTEHAKRKT VTSLDVVYAL KRQGRTLYGF GG	3
93.2 %	*A. oryzae* Q76MU7	SGRGKGGKGL GKGGAKRHRK ILRDNIQGIT KPAIRRLARR GGVKRISAMI YEETRGVLKS FLESVIRDAV TYTEHAKRKT VTSLDVVYAL KRQGRTLYGF GG	3

A. cervicornis H4.S proposed sequence

H4 Sequences - % similarity to proposed sequence — mutation

%	Sequence	H4 sequence	Prot. Score
	**Acropora cervicornis Proposed Sequence**	SGRGKGGKGL GKGGAKRHRK ILRDNIQGIT KPAIRRLARR GGVKRISGLI YEETRGVLKV FLENVIRDAV TYTEHAKRKT VTSMDVVYAL KRQGRTLYGF GG	
99.0%	*Acropora formosa* P35059	SGRGKGGKGL GKGGAKRHRK ILRDNIQGIT KPAIRRLARR GGVKRISGLI YEETRGVLKV FLENVIRDAV TYTEHAKRKT VTAMDVVYAL KRQGRTLYGF GG	3
99.0 %	*Dendro. klunz.* Q6LAF1	SGRGKGGKGL GKGGAKRHRK ILRDNIQGIT KPAIRRLARR GGVKRISGLI YEETRGVLKV FLENVIRDAV TYTEHAKRKT VTAMDVVYAL KRQGRTLYGF GG	3
94.1 %	*Penaeus vann.* P83865	TGRGKGGKGL GKGGAKRHRK VLRDNIQGIT KPAIRRLARR GGVKRISGLI YEETRGVLKV FLENVIRDAV TYTEHAKRKT VTAMDVVYAL KRXXXTLYGF GG	5
98.0 %	*Homo sapiens* P62805	SGRGKGGKGL GKGGAKRHRK VLRDNIQGIT KPAIRRLARR GGVKRISGLI YEETRGVLKV FLENVIRDAV TYTEHAKRKT VTAMDVVYAL KRQGRTLYGF GG	5
93.1 %	*Asper. fumigatus* Q7LKT3	TGRGKGGKGL GKGGAKRHRK ILRDNIQGIT KPAIRRLARR GGVKRISAMI YEETRGVLKT FLEGVIRDAV TYTEHAKRKT VTSLDVVYAL KRQGRTLYGF GG	3
94.1 %	*E. nidulans H4.1* P23750	SGRGKGGKGL GKGGAKRHRK ILRDNIQGIT KPAIRRLARR GGVKRISAMI YEETRGVLKT FLEGVIRDAV TYTEHAKRKT VTSLDVVYAL KRQGRTLYGF GG	3
93.1 %	*E. nidulans H4.2* P23751	SGRGKGGKGL GKGGAKRHRK ILRDNIQGIT KPAIRRLARR GGVKRISAMI YEETRGVLKS FLESVIRDAV TYTEHAKRKT VTSLDVVYAL KRQGRTLYGF GG	3
93.1 %	*N. crassa* P04914	TGRGKGGKGL GKGGAKRHRK ILRDNIQGIT KPAIRRLARR GGVKRISAMI YEETRGVLKT FLEGVIRDAV TYTEHAKRKT VTSLDVVYAL KRQGRTLYGF GG	3
93.1 %	*T. funiculosus* Q711M0	TGRGKGGKGL GKGGAKRHRK ILRDNIQGIT KPAIRRLARR GGVKRISAMI YEETRGVLKT FLEGVIRDAV TYTEHAKRKT VTSLDVVYAL KRQGRTLYGF GG	3
94.1 %	*A. oryzae* Q76MU7	SGRGKGGKGL GKGGAKRHRK ILRDNIQGIT KPAIRRLARR GGVKRISAMI YEETRGVLKS FLESVIRDAV TYTEHAKRKT VTSLDVVYAL KRQGRTLYGF GG	3

Figure S86. A. cervicornis H4 variant proposed sequences compared to highest similarity sequences from UniProt. The % similarity to the proposed sequence and UniProt entry numbers are given for each comparison sequence. Protein existence score is given based on UniProt entry and common mutations between species are highlighted in green.

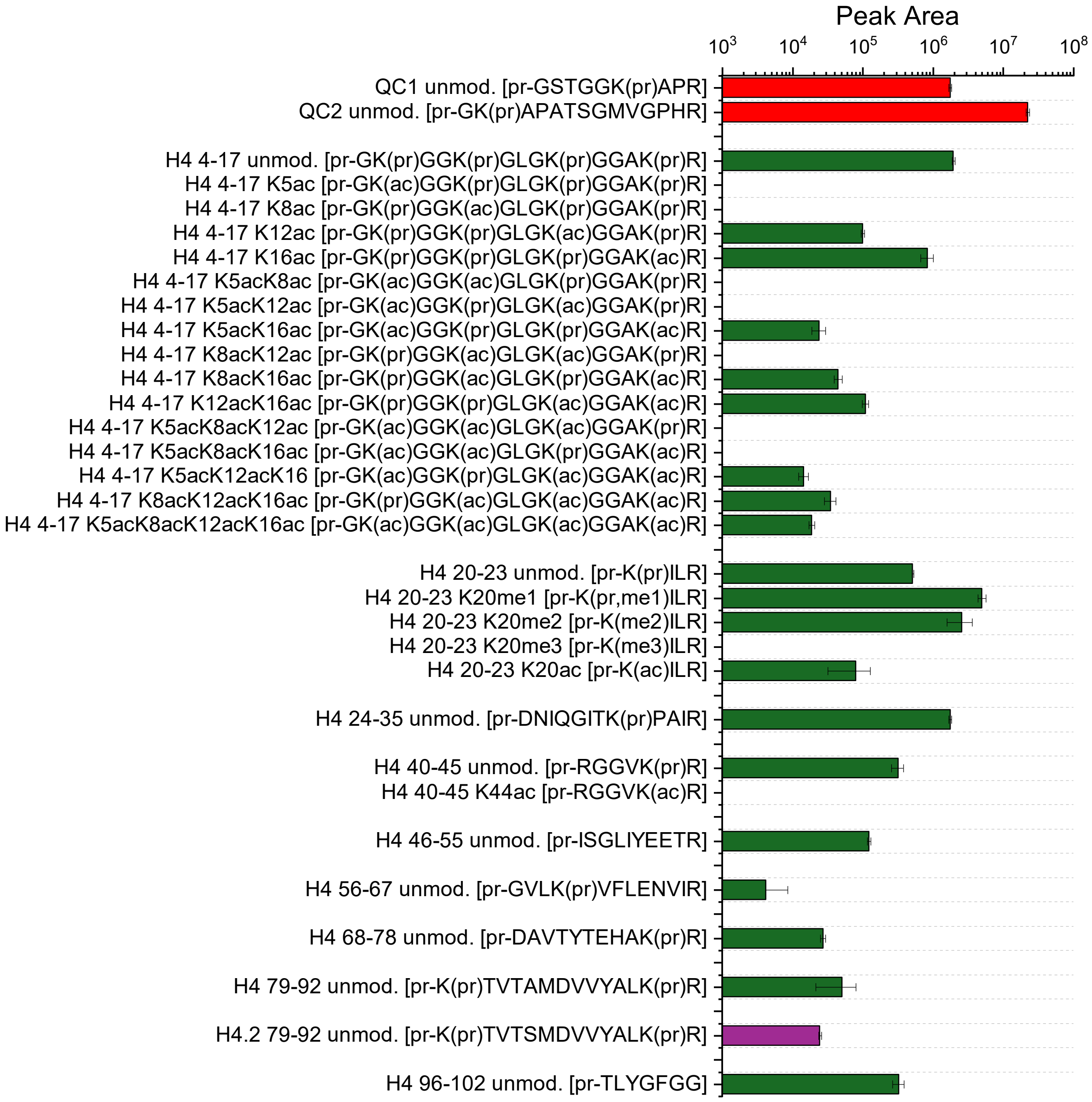

Figure S87. Bottom-up H4 target peptides with relative abundance of observed peptides. Relative abundances are reported as the mean of three analytical replicates with standard deviation error bars on a $\log_{10}$ scale.

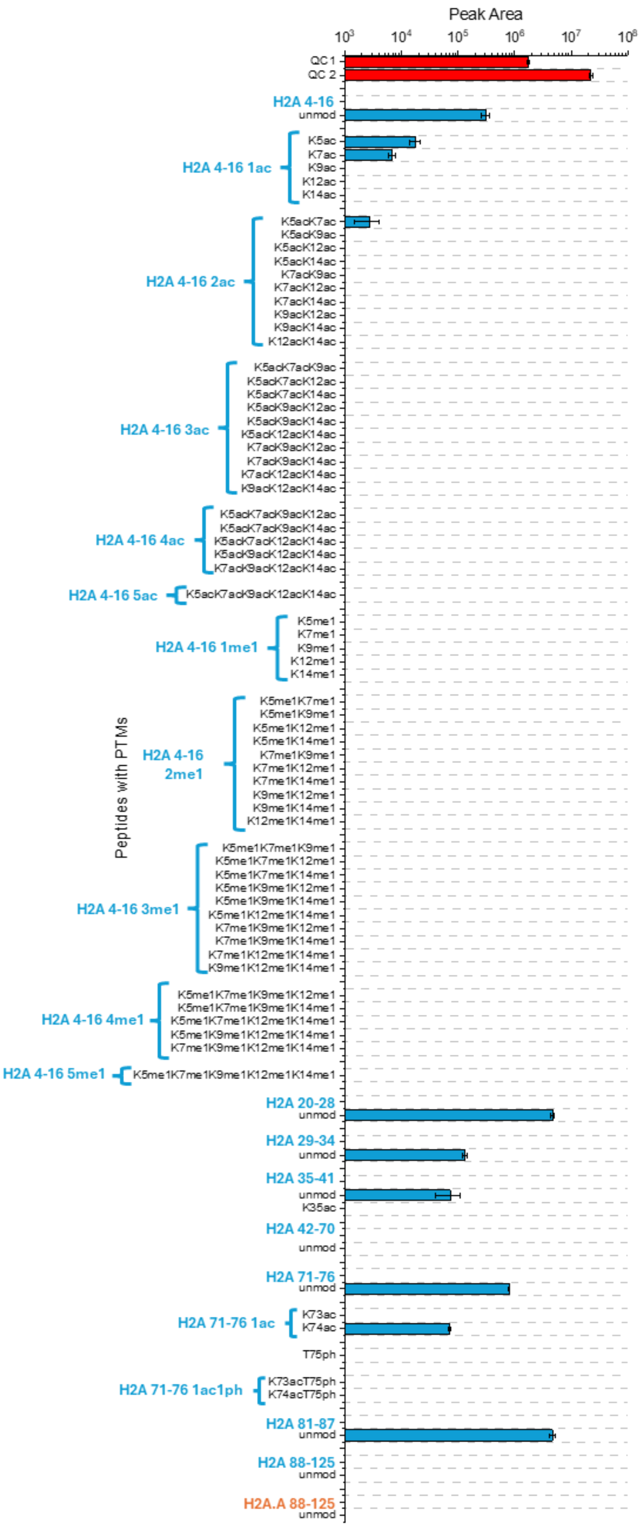

Figure S88. Bottom-up H2A target peptides with relative abundance of observed peptides. Relative abundances are reported as the mean of three analytical replicates with standard deviation error bars on a log_{10} scale.

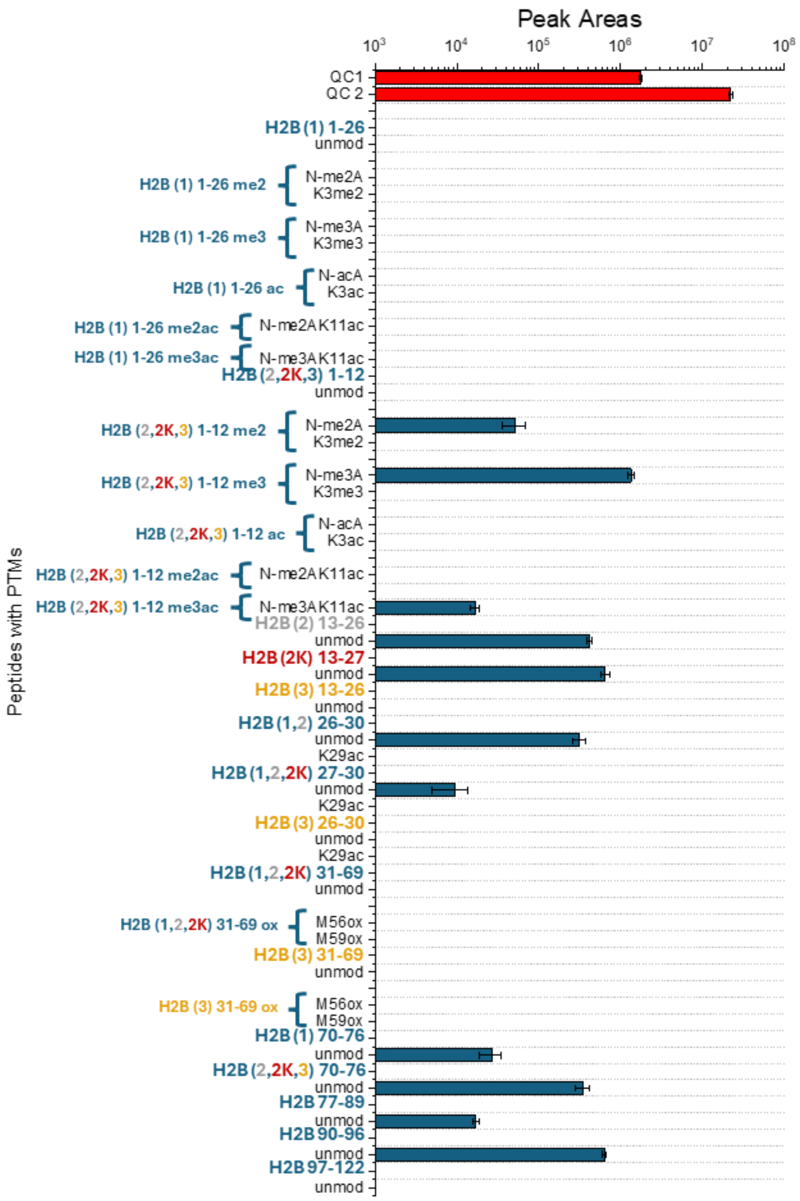

Figure S89. Bottom-up H2B target peptides with relative abundance of observed peptides. Relative abundances are reported as the mean of three analytical replicates with standard deviation error bars on a $\log_{10}$ scale.

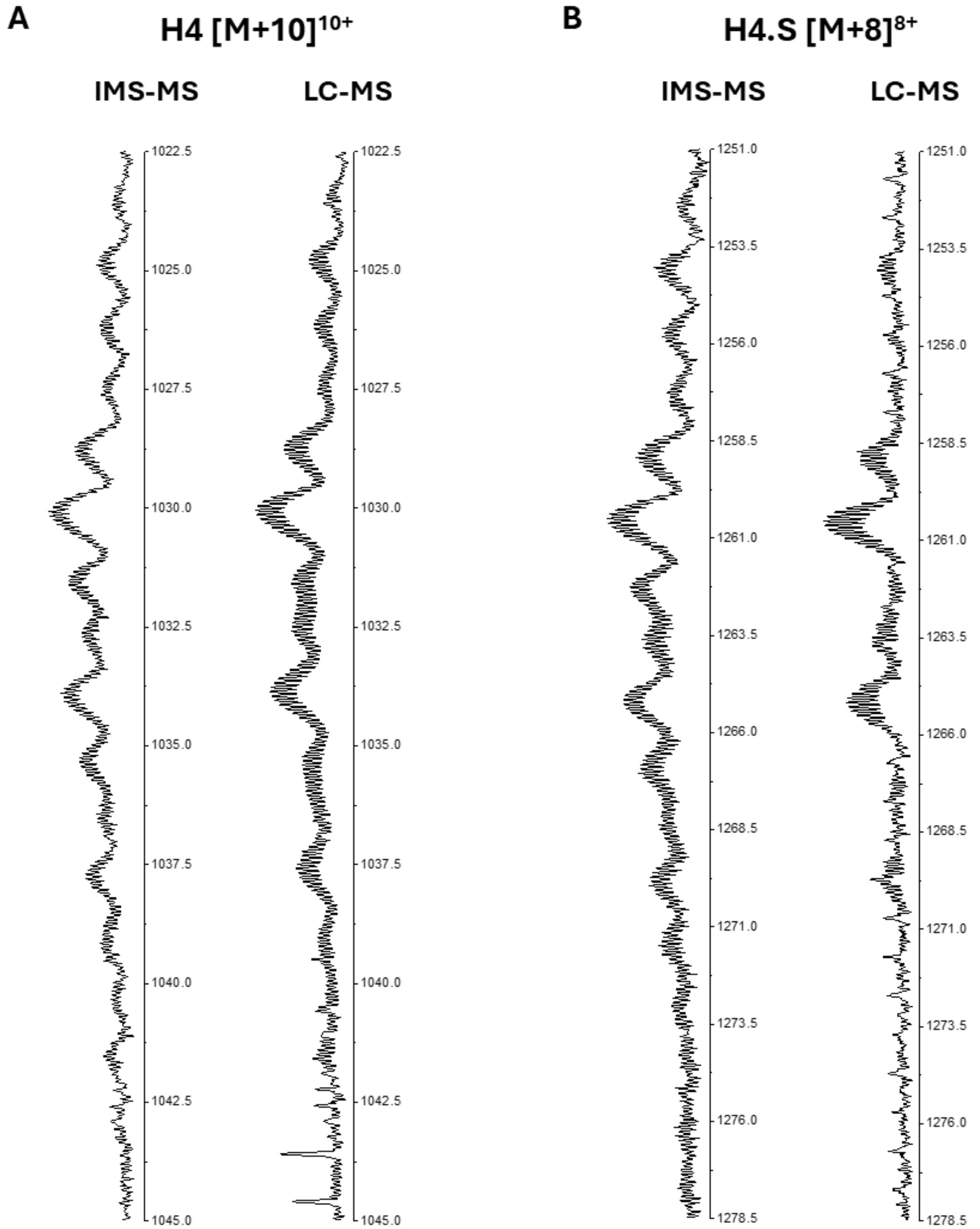

Figure S90. Comparison of S/N from IMS-MS and LC-MS. (A) H4 *A. cervicornis* $[M+10H]^{10+}$ proteoforms. (B) H4.S *A. cervicornis* $[M+8H]^{8+}$ proteoforms.
